# Safety of Repeated Open-Label Treatment Courses of Intravenous Ofatumumab, a Human Anti-CD20 Monoclonal Antibody, in Rheumatoid Arthritis: Results from Three Clinical Trials

**DOI:** 10.1371/journal.pone.0157961

**Published:** 2016-06-23

**Authors:** Emilia Quattrocchi, Mikkel Østergaard, Peter C. Taylor, Ronald F. van Vollenhoven, Myron Chu, Stephen Mallett, Hayley Perry, Regina Kurrasch

**Affiliations:** 1 R&D Immunoinflammation, GlaxoSmithKline, Stockley Park, Middlesex, United Kingdom; 2 Copenhagen Center for Arthritis Research, Center for Rheumatology and Spine Diseases, Glostrup Hospital, Copenhagen, Denmark and Department of Clinical Medicine, University of Copenhagen, Copenhagen, Denmark; 3 Kennedy Institute of Rheumatology, Nuffield Department of Orthopaedics, Rheumatology and Musculoskeletal Sciences, University of Oxford Botnar Research Centre, Oxford, United Kingdom; 4 Unit for Clinical Therapy Research, The Karolinska Institute, Stockholm, Sweden; 5 R&D Immunoinflammation, GlaxoSmithKline, Collegeville, Pennsylvania, United States of America; 6 Clinical Statistics, GlaxoSmithKline, Stockley Park, Middlesex, United Kingdom; Center for Rheumatic Diseases, INDIA

## Abstract

**Objectives:**

To investigate the safety of ofatumumab retreatment in rheumatoid arthritis.

**Methods:**

Patients with active rheumatoid arthritis participating in two phase III trials (OFA110635 and OFA110634) and a phase II extension trial (OFA111752) received individualised open-label ofatumumab retreatment (700 mg X 2 intravenous infusions two weeks apart) ≥24 weeks following the first course and ≥16 weeks following further courses. Retreatment required evidence of clinical response followed by disease relapse. These studies were prematurely terminated by the sponsor to refocus development on subcutaneous delivery. Due to differences in study designs and populations, data are summarised separately for each study.

**Results:**

483 patients (243, 148 and 92 in OFA110635, OFA110634 and OFA111752 respectively) received up to 7 treatment courses of intravenous ofatumumab; cumulative duration of exposure was 463, 182 and 175 patient-years, respectively. Mean time between courses was 17–47 weeks. Ofatumumab induced a profound depletion of peripheral B-lymphocytes. Retreated patients derived benefit based on improvement in DAS28. Adverse events were reported for 93% (226/243), 91% (134/148) and 76% (70/92), serious adverse events for 18% (44/243), 20% (30/148) and 12% (11/92) and serious infections for 3% (8/243), 5% (7/148) and 1% (1/92) of patients in OFA110635, OFA110634 and OFA111752, respectively. The most common adverse events were infusion-related reactions during the first infusion of the first course (48–79%); serious infusion-related reactions were rare (<1% [1/243], 5% [8/148], and 1% [1/92] of patients). Two deaths occurred (fulminant hepatitis B virus infection and interstitial lung disease).

**Conclusions:**

Ofatumumab was generally well tolerated with no evidence of increased safety risks with multiple retreatments. Serious infections were uncommon and did not increase over time.

**Trial Registration:**

ClinicalTrials.gov 110635

ClinicalTrials.gov 110634

ClinicalTrials.gov 111752

## Introduction

The development of B-lymphocyte depletion therapy marked a significant advance in the treatment of RA. In the late 1990s rituximab, a chimeric mouse-human monoclonal antibody (mAb) selectively targeting the B-cell surface CD20 antigen, was shown to be effective in patients with active rheumatoid arthritis (RA) [1–3]. Considerable variability in clinical response was observed despite effective peripheral B-cell depletion, and repeated treatment cycles were necessary to achieve sustained efficacy [4].

Ofatumumab is a human immunoglobulin G (IgG)1ĸ mAb that binds to a membrane-proximal epitope on the human CD20 molecule, distinct from the epitope recognised by rituximab [5,6] and by humanised anti-CD20 mAbs like ocrelizumab [7,8], veltuzumab [9] and obinutuzumab [10]. Ofatumumab induces potent B-cell lysis primarily through complement-dependent cytotoxicity and antibody-dependent cell-mediated cytotoxicity [6,11]. It is approved, as an intravenous infusion, for the treatment of chronic lymphocytic leukemia [12].

A randomised, placebo-controlled phase I/II study of ofatumumab at doses of 300, 700 and 1000 mg administered as two intravenous infusions two weeks apart showed significant clinical benefit over placebo in patients with active RA and an inadequate response to disease-modifying anti-rheumatic drugs (DMARDs) [13]. The 700 mg X 2 dose (one treatment course) was selected for further investigation in two confirmatory phase III trials in defined populations of RA patients. Study OFA110635 enrolled only active RA patients who had never been previously administered biologic therapies (biologic-naïve) and had demonstrated an inadequate response to methotrexate (MTX); study OFA110634 enrolled active RA patients who had failed one or more tumour necrosis factor (TNF) antagonists. OFA111752 was an open-label ofatumumab re-treatment extension study of the initial dose-ranging trial in active RA patients who were not responding to DMARDs. A key objective of these studies was to investigate the efficacy and safety of repeated treatment courses of ofatumumab administered on an individualised basis (dependent upon clinical need), to active RA patients despite previous RA treatments with either MTX, TNF-inhibitors or DMARDs.

Results of the initial dose-ranging study [13] and the 24-week, double-blind, placebo-controlled period of the Phase III study in biologic-naive MTX-refractory patients [14] indicated that the short-term efficacy and safety of intravenous ofatumumab in RA was similar overall to that observed with other anti-CD20 therapies [8,15]. Furthermore, consistent with the high potency of ofatumumab, a single-blind phase I/II trial in RA patients on background MTX demonstrated that even single subcutaneous formulation doses of ofatumumab, as low as 30 mg, were able to induce profound and persistent peripheral B-cell depletion [16]. Based on the encouraging results of the subcutaneous study, the clinical development of the intravenous formulation of ofatumumab in RA was discontinued and the three ongoing RA trials were prematurely terminated in favour of initiating a clinical development programme to evaluate the effects of subcutaneously administered ofatumumab in autoimmune diseases instead.

This article reports the long-term safety and efficacy data obtained following repeated treatment cycles of intravenous ofatumumab from the three terminated clinical trials in RA patients. Due to differences in study designs and patient populations, data are summarised separately for each study and have not been pooled.

## Material and Methods

### Study designs and patients

The protocols for the three clinical trials included in this article and the CONSORT checklist are available as supporting information (S1 Protocol; S2 Protocol; S3 Protocol and S1 CONSORT Checklist).

OFA110635 and OFA110634 (ClinicalTrials.gov identifiers NCT00611455 and NCT00603525, respectively) were Phase III trials with a 24-week randomised, placebo-controlled double-blind (DB) period, a 120-week open-label (OL) retreatment period and a safety follow-up (FU) of up to two years. OFA111752 (ClinicalTrials.gov identifier NCT00655824) was a Phase II extension trial with a 130-week OL period and safety FU, in patients who had received ofatumumab (300, 700 or 1000 mg) or placebo in a preceding Phase I/II dose-ranging trial [13] (Table 1). The trials were conducted between January 2008 and July 2013. Ethical approval was obtained from the independent ethics committee or institutional review board for each participating site and all patients provided written informed consent. Key inclusion/exclusion criteria for each trial are summarised in Table 1.

**Table 1:** Study Designs, Demographic and Baseline Characteristics.

	OFA110635	OFA110634	OFA111752
**Study design**			
Type of study	Phase III	Phase III	Phase II extension
Planned duration	24-week DB period; 120-week OL period^a^; Safety FU up to 2 yrs.	24-week DB period; 120-week OL period^a^; Safety FU up to 2 yrs.	130-week OL period^a^; Safety FU up to 2 yrs.
Number of sites	36	41	17
Geographic locations	Europe, South America, Australia	Europe, South America, East Asia	Europe, North America
**Key inclusion and exclusion criteria**			
Age	≥18 years	≥18 years	≥18 years
Gender	Male or non-pregnant female	Male or non-pregnant female	Male or non-pregnant female
RA disease characteristics	Active RA for ≥6 months; ≥8 swollen and ≥8 tender joints (of 66/68 assessed); CRP ≥1.0 mg/dl or ESR ≥22 mm/h; DAS28-ESR ≥3.2	Active RA for ≥6 months; ≥8 swollen and ≥8 tender joints (of 66/68 assessed); CRP ≥1.0 mg/dl or ESR ≥22 mm/h; DAS28-ESR ≥3.2	Active RA for ≥6 months; ≥3 swollen and ≥3 tender joints (of 28 assessed); DAS28-ESR ≥3.2
Previous RA therapy	Biologic naive and failed treatment with MTX	Failed treatment with TNF antagonist	Failed treatment with at least one DMARD
MTX therapy at study entry	Required (7.5–25 mg/week for ≥12 weeks and at stable dose for ≥4 weeks)	Required (7.5–25 mg/week for ≥12 weeks and at stable dose for ≥4 weeks)	Not required (if on MTX had to be on 7.5–25 mg/week for ≥12 weeks, and at stable dose for ≥4 weeks)
IgG	≥LLN	≥LLN	≥LLN
**Demographic and baseline characteristics**			
N	260	169	92
Mean (SD) age, years	52.7 (11.39)	53.5 (12.67)	52.9 (10.73)
Female, n (%)	214 (82)	140 (83)	84 (91)
Race, n (%)			
White	253 (97)	135 (80)	92 (100)
Asian	1 (<1)	32 (19)	0
African American/African	1 (<1)	0	0
Other	5 (2)	2 (1)	0
Mean (SD) disease duration, yrs	8.51 (8.174)	12.8 (9.08)	12.7 (8.95)
Concomitant MTX, n (%)	260 (100)	169 (100)	87 (95)
RF positive, n (%)	219 (84)	124 (73)	79 (86)
Anti-CCP positive, n (%)	224 (86)	137 (81)	80 (87)
Mean (SD) swollen joint count	15.9 (7.14)	16.4 (7.90)	11.2 (5.22)
Mean (SD) tender joint count	27.6 (13.00)	28.0 (13.29)	15.5 (6.45)
Mean (SD) DAS28-CRP	5.7 (0.80)	5.9 (0.96)	5.8 (0.95)

DB, double-blind; FU, follow-up; LLN, lower limit of normal; OL, open-label.

^a^In OFA110635 and OFA110634, ofatumumab treatment course 1 was the first course of ofatumumab the patient received, regardless of whether this occurred during the DB period (for those patients randomised to receive DB ofatumumab) or the OL period (for those patients randomised to receive DB placebo). In OFA111752, ofatumumab treatment course 1 was the first course of ofatumumab the patient received in OFA111752 (some patients had previously received a course of ofatumumab at a dose of 300, 700 or 1000 mg in the preceding Phase I/II trial).

Also excluded were patients with chronic or ongoing active infectious disease requiring systemic treatment such as, but not limited to, chronic renal infection, chronic chest infection with bronchiectasis, tuberculosis (TB) and active hepatitis B and C. In particular, patients with a screening chest X-ray suggestive of TB without documentation of adequate TB treatment were excluded. Screening for latent TB infection using intradermal injection of tuberculin (e.g. the Mantoux test or equivalent) was to be conducted in accordance with local guidelines. Patients with a positive skin tuberculin test were excluded if the investigator judged the patient to be at risk of latent TB infection.

In studies OFA110635 and OFA110634, ofatumumab treatment course 1 was the first course of ofatumumab the patient received, regardless of whether this occurred during the DB period (for those patients randomised to receive DB ofatumumab) or the OL period (for those patients randomised to receive DB placebo). In study OFA111752, ofatumumab treatment course 1 was the first course of ofatumumab the patient received in this OL study (as some patients had previously received a course of ofatumumab at a dose of 300, 700 or 1000 mg X 2 during the preceding Phase I/II trial). Active RA was defined according to the ACR 1987 classification criteria [17]. The swollen joint count was based on 66 joints for OFA110635 and OFA110634 and 28 joints for OFA111752; the tender joint count was based on 68 joints for OFA110635 and OFA110634 and 28 joints for OFA111752. Baseline DAS28 was calculated using CRP; if CRP was missing ESR was used instead. Previous RA treatment failure was defined as insufficient efficacy or intolerance. Patients underwent a washout period of ≥4 weeks for DMARDs (leflunomide ≥12 weeks or administration of cholestyramine according to manufacturer’s instructions) but maintained their concomitant stable MTX therapy.

In the OL period of each trial, eligible patients received repeated ofatumumab courses (two 700 mg intravenous infusions administered two weeks apart) at individualised intervals, following premedication with oral antihistamine, oral paracetamol and intravenous methylprednisolone [14]. In order to avoid retreating patients who may not be responsive to B-cell depleting therapy patients were eligible to receive further ofatumumab treatment, per protocol, only if they had demonstrated an efficacy response, defined as achieving at least a moderate European League Against Rheumatism [EULAR] response and/or ≥20% improvement in both swollen and tender joint counts compared to baseline, at least once within 24 weeks from the previous treatment course which was followed by a worsening in disease activity defined as achieving a Disease Activity Score based on erythrocyte sedimentation rate [DAS28-ESR] ≥3.2 plus a ≥0.6 increase in DAS28-ESR compared to the lowest DAS28-ESR after the previous treatment course and/or a ≥20% increase in both tender and swollen joint counts compared to lowest joint counts since the previous treatment course. Breakthrough pain management with analgesics, non-steroidal anti-inflammatory drugs and one intra-articular corticosteroid injection in one joint per 6-month period was allowed. The use of other DMARDs (except for MTX) was prohibited; however, patients in studies OFA110635 and OFA110634 who did not achieve a clinical response were allowed non-biologic DMARD rescue treatment from week 16 of the DB phase, but this precluded their entry into the OL retreatment period of the trials. In addition, patients with a circulating IgG level below the lower limit of normal (LLN), as measured by a central laboratory, at any time after the second ofatumumab treatment course were withdrawn from further retreatment. Patients were evaluated every 8 weeks and the minimum interval between retreatments was 16 weeks.

Patients who completed the OL period or who did not qualify for retreatment entered a safety FU and were monitored every 12 weeks until their B-cells and circulating IgG levels had returned to normal or baseline values, or for a maximum of 2 years. During the FU period RA therapy was prescribed at each investigator’s discretion based on the patient’s clinical need and local practice, but if treatment with another B-cell depleting agent was initiated the patient was to be withdrawn from the trial.

### Assessments

DAS28-ESR (measured at local laboratory) and DAS28 based on C-reactive protein (DAS28-CRP) (measured at central laboratory) were assessed at each visit during the DB and OL periods; DAS28-ESR was used by investigators to determine if patients met the criteria for retreatment. Adverse events (AEs) were recorded during DB and OL periods; serious adverse events (SAEs) only were to be recorded during FU. Infusion-related reactions (IRRs) occurring during and up to 24 h after completion of each infusion (and likely to represent clinical signs and symptoms characteristic of infusion reactions) were identified by a Safety Review Team; serious IRRs were those that met the definition of a SAE. Although progressive multifocal leukoencephalopathy (PML) occurs very rarely in RA patients treated with rituximab [18], patients were monitored throughout the trials by neurological questionnaire and plasma JC virus DNA testing.

Blood and urine samples were collected at regular intervals for haematology, clinical chemistry, immunoglobulins, biomarkers (OFA110635 and OFA111752 only), virology [plasma JC virus by PCR, hepatitis B virus (HBV) and hepatitis C virus (HCV) serology; positivity at screening was exclusionary] and urinalysis. In OFA110635 and OFA110634 CD19^+^, CD3^+^, CD4^+^ and CD8^+^ lymphocytes were measured by fluorescence-activated cell sorting. In OFA111752, anti-ofatumumab antibodies were measured by validated enzyme-linked immunosorbent assay; positive samples were further characterised (including testing for the presence of neutralising antibodies).

### Statistical methods

Data are summarised descriptively for each study, by ofatumumab treatment course. No statistical tests were conducted. The randomised population (OFA110635 and OFA110634 only) comprised all patients who received at least one infusion of study drug during the DB period. The ofatumumab population for each study comprised all patients who received at least one ofatumumab infusion; this is the primary population used for reporting safety and efficacy data. The FU population comprised all patients who underwent at least one safety assessment during the FU period.

For OFA110635 and OFA110634, baseline for the OL period was defined as the latest value recorded prior to the first infusion of study drug in the DB period. Ofatumumab treatment course 1 was the first course of ofatumumab the patient received, regardless of whether this occurred during the DB period (for those patients randomised to receive DB ofatumumab) or the OL period (for those patients randomised to receive DB placebo).

For OFA111752, baseline was defined as the latest value recorded on or before the date of the first infusion of ofatumumab in this extension study. Ofatumumab treatment course 1 was defined as the first course of ofatumumab the patient received in OFA111752 even though some patients had received a course of ofatumumab at a dose of 300, 700 or 1000 mg in the preceding dose-ranging trial [13]. For FU safety monitoring, baseline values for B-cells and IgG were the latest value recorded on or before the date of the first infusion of study drug in the preceding Phase I/II trial [13].

Time to retreatment was defined as the interval between the first infusion (infusion A) of treatment course n and infusion A of treatment course n+1. Disease remission was defined as DAS28 score <2.6 at any time during the treatment course and low disease activity was defined as DAS28 score ≥2.6 and <3.2 at any time during the treatment course. The overall duration of exposure for a patient to ofatumumab was defined as the time between the first infusion and completion/withdrawal from their final treatment course. The duration of exposure within a treatment course was defined as the time from start of the first infusion for that treatment course and the time of retreatment or withdrawal/completion.

AEs were coded using the Medical Dictionary for Regulatory Activities (MedDRA) (version 16 in OFA110635 and OFA110634; version 13 in OFA111752) and categorised by system organ class (SOC) and preferred term. Infections and neoplasms were those events reported within the SOCs of “Infections and infestations” and “Neoplasms (benign, malignant and unspecified, including cysts and polyps)”, respectively.

## Results

### Patient population

**Fig 1:**
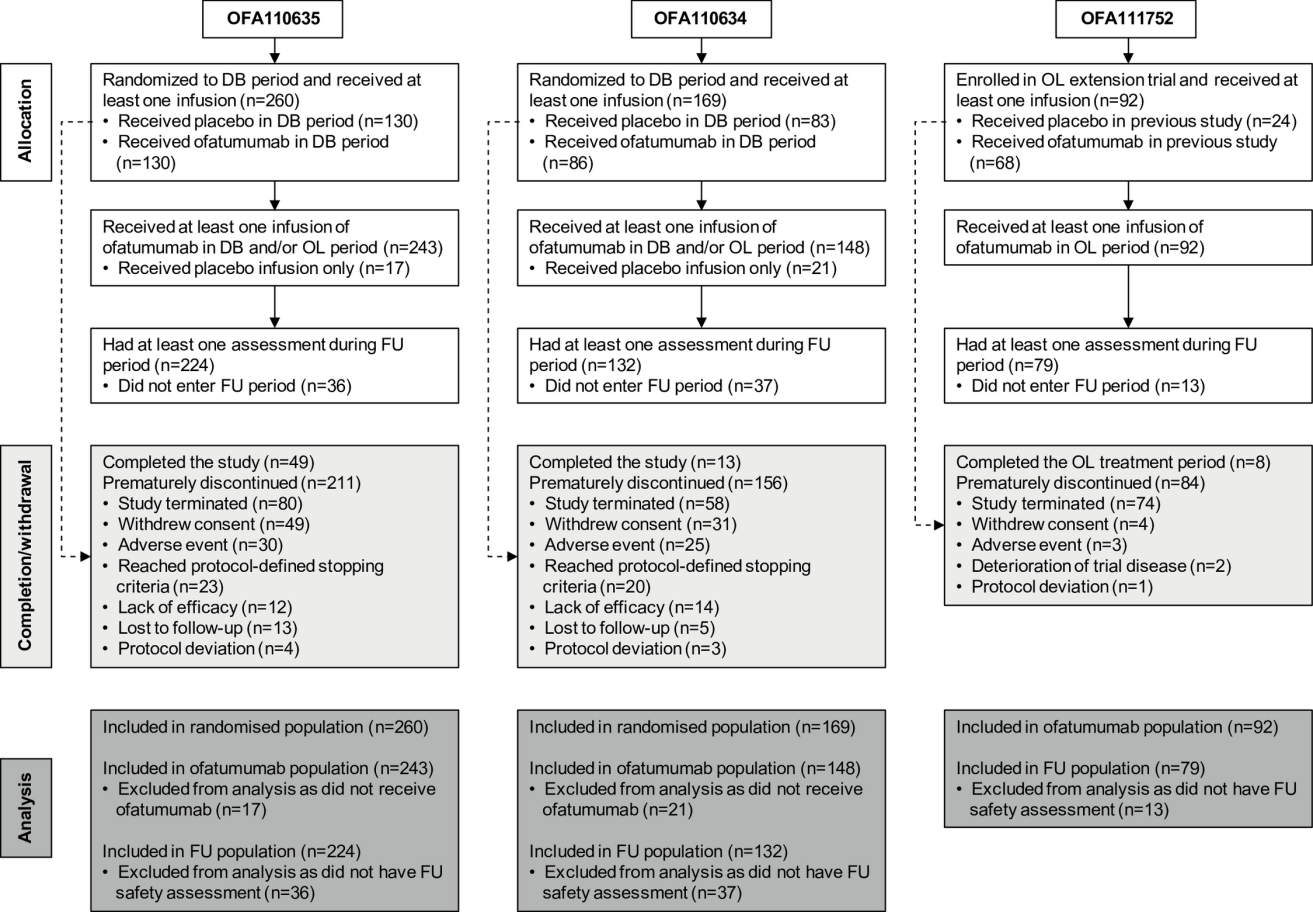
Flow Diagram of Patient Disposition in Each Study. The diagram shows the numbers of patients who were enrolled/randomized, received at least one infusion of ofatumumab, completed/discontinued, and included in analysis populations in each of the three studies. DB, double-blind treatment period; OL, open-label treatment period; FU, safety follow-up.

Overall, 483 RA patients received at least one infusion of ofatumumab across the three studies (243, 148 and 92 in OFA110635, OFA110634 and OFA111752, respectively) (Fig 1) and 364 patients (198, 93 and 73 patients, respectively) received more than one individualised treatment course, up to a maximum of seven courses. The cumulative duration of exposure to ofatumumab in each study was 463, 182 and 175 patient-years, respectively. The most common reason for patient withdrawal was premature study termination by the sponsor (Fig 1).

Demographic and baseline characteristics were generally similar across the studies (Table 1), except that OFA110634 was the only trial to enrol Asian (Korean) patients (24 patients, 19%). Mean disease duration was lower, as expected, in the biological-naive MTX-refractory trial OFA110635 compared to OFA110634 and OFA111752 (8.5, 12.8 and 12.7 years, respectively). Most patients were women (83–91%), Rheumatoid Factor (RF) positive (73–86%) and anti-Cyclic Citrullinated Peptide antibody (anti-CCP) positive (81–87%), with a mean age of 53 years. All patients in OFA110635 and OFA110634 and 95% of patients in OFA111752 received concomitant MTX therapy.

### Time to ofatumumab retreatment

Following the initial course of ofatumumab a decreasing number of patients needed to be retreated (Table 2), as retreatment depended on each patient’s personal clinical need as defined in the Methods’ section. It is important to note, however, that due to the early termination of these studies by the sponsor, patients recruited later in the trials were withdrawn prematurely, possibly resulting in an underestimation of treatment cycles required for disease control until study end. Table 2 shows the mean time which occurred between treatment courses, this ranged from 17 to 47 weeks. There seemed to be a tendency to shorter intervals between later ofatumumab retreatments as B-lymphocytes seemed to repopulate earlier in patients requiring several treatment courses.

When considering safety and efficacy data obtained from later treatment courses the small number of patients should also be noted and the corresponding data interpreted with caution.

**Table 2:** Time to Retreatment in Weeks by Ofatumumab Treatment Course in Each Study.

Ofatumumab Treatment Course							
	1	2	3	4	5	6	7
**OFA110635**	**N = 243**	**N = 198**	**N = 136**	**N = 72**	**N = 31**	**N = 11**	**N = 2**
Number retreated	198	136	72	31	11	2	0
Mean (SD)	34.3 (15.8)	34.5 (15.2)	28.8 (10.7)	20.8 (7.0)	19.9 (5.4)	17.9 (0.4)	NA
Median (min, max)	30.2 (16.0, 97.3)	31.9 (16.0, 85.4)	28.0 (16.0, 58.1)	16.9 (16.0, 40.0)	17.0 (16.0, 31.9)	17.9 (17.6, 18.1)	NA
**OFA110634**	**N = 148**	**N = 93**	**N = 63**	**N = 30**	**N = 13**	**N = 6**	**-**
Number retreated	93	63	30	13	6	0	
Mean (SD)	32.8 (12.0)	28.6 (11.1)	24.6 (8.1)	23.9 (6.1)	19.1 (2.8)	NA	-
Median (min, max)	28.0 (16.0, 65.9)	25.0 (16.0, 65.0)	23.4 (16.0, 39.7)	23.7 (16.1, 32.9)	17.9 (16.3, 23.1)	NA	-
**OFA111752**	**N = 92**	**N = 73**	**N = 53**	**N = 20**	**N = 8**	**N = 6**	**N = 2**
Number retreated	73	53	20	8	4	2	0
Mean (SD)	41.6 (18.2)	47.0 (17.5)	33.7 (13.4)	22.1 (2.7)	23.1 (3.2)	17.1 (0.0)	NA
Median (min, max)	36.0 (18.3, 98.3)	49.4 (18.6, 81.4)	30.1 (17.9, 60.1)	22.9 (17.9, 25.9)	22.3 (20.4, 27.4)	17.1 (17.1, 17.1)	NA

Time to retreatment was defined as the interval between infusion A of treatment course n and infusion A of treatment course n+1. For each patient’s final treatment course, the time was defined as the interval from infusion A to the date of withdrawal or completion. The minimum period allowed per protocol before retreatment was 16 weeks, except for the first course of ofatumumab in the double-blind period of OFA110635 and OFA110634 for which the minimum period allowed before retreatment was 24 weeks. Data from the final treatment course when patients withdrew due to early termination of the study were not included in this analysis.

### Overall safety profile

The majority of patients experienced at least one AE across the three ofatumumab studies: 93% (226/243) in OFA110635, 91% (134/148) in OFA110634, and 76% (70/92) in OFA111752; these AEs were mainly due to IRRs occurring during the first ofatumumab infusion (see Table 3, Table 4 and Table 5 for OFA110635, OFA110634 and OFA111752 respectively). AEs leading to trial discontinuation were reported overall for 12% (29/243), 16% (24/148) and 3% (3/92) of patients. SAEs were reported overall for 18% (44/243), 20% (30/148) and 12% (11/92) of patients in study OFA110635, OFA110634 and OFA111752; SAEs leading to trial discontinuation were reported for 4% (9/243), 6% (9/148) and 1% (1/92) of patients, respectively. Most of the SAEs leading to discontinuation in studies OFA110634 (6/9 patients) and OFA111752 (1/1 patient) were serious IRRs, whereas only one of the SAEs leading to discontinuation in study OFA110635 was a serious IRR (1/9 patients).

The frequencies of adverse events reported in the placebo and ofatumumab groups during the double-blind periods of studies OFA110635 and OFA110634 are provided as supporting information (S1 and S2 Tables).

**Table 3:** Incidence of Adverse Events by Ofatumumab Treatment Course in OFA110635.

	Ofatumumab Treatment Course							
	1	2	3	4	5	6	7	Overall
**OFA110635**	**N = 243**	**N = 198**	**N = 136**	**N = 72**	**N = 31**	**N = 11**	**N = 2**	**N = 243**
**Any AE**	**212 (87)**	**151 (76)**	**92 (68)**	**43 (60)**	**14 (45)**	**5 (45)**	**0**	**226 (93)**
IRR on day of first infusion	168 (69)	110 (56)	64 (47)	22 (31)	2 (6)	2 (18)	0	191 (79)
IRR on day of second infusion	7 (3)	2 (1)	1 (<1)	2 (3)	0	1 (9)	0	11 (5)
AE leading to discontinuation of investigational product/withdrawal	15 (6)	4 (2)	6 (4)	4 (6)	0	0	0	29 (12)
**Any SAE (fatal or non-fatal)**	**14 (6)**	**19 (10)**	**12 (9)**	**4 (6)**	**1 (3)**	**0**	**0**	**44 (18)**
Serious IRR	1 (<1)	0	0	0	0	0	0	1 (<1)
SAE leading to discontinuation of investigational product/withdrawal	2 (<1)	3 (2)	3 (2)	1 (1)	0	0	0	9 (4)
Death	1 (<1)	0	1 (<1)	0	0	0	0	2 (<1)
**Any infection**	**75 (31)**	**68 (34)**	**31 (23)**	**13 (18)**	**6 (19)**	**2 (18)**	**0**	**130 (53)**
Most common infections (≥5% overall)								
Nasopharyngitis	15 (6)	13 (7)	4 (3)	2 (3)	2 (6)	1 (9)	0	34 (14)
Urinary tract infection	15 (6)	10 (5)	3 (2)	4 (6)	1 (3)	1 (9)	0	27 (11)
Upper respiratory tract infection	11 (5)	7 (4)	2 (1)	0	0	0	0	20 (8)
Bronchitis	4 (2)	11 (6)	5 (4)	0	0	0	0	18 (7)
Pharyngitis	6 (2)	4 (2)	5 (4)	0	1 (3)	0	0	14 (6)
**Any serious infection**	**3 (1)**	**2 (1)**	**2 (1)**	**1 (1)**	**0**	**0**	**0**	**8 (3)**
Diverticulitis	0	0	0	1 (1)	0	0	0	1 (<1)
Endometritis	0	1 (<1)	0	0	0	0	0	1 (<1)
Hepatitis B	0	0	1 (<1)	0	0	0	0	1 (<1)
Lower respiratory tract infection	1 (<1)	0	0	0	0	0	0	1 (<1)
Pertussis	0	0	1 (<1)	0	0	0	0	1 (<1)
Pneumonia	1 (<1)	0	0	0	0	0	0	1 (<1)
Pyelonephritis acute	0	1 (<1)	0	0	0	0	0	1 (<1)
Tooth infection	1 (<1)	0	0	0	0	0	0	1 (<1)
**Any neoplasm**	**0**	**2 (1)**	**1 (<1)**	**2 (3)**	**0**	**0**	**0**	**5 (2)**
**Any serious malignant neoplasm**	**0**	**1 (<1)**	**1 (<1)**	**1 (1)**	**0**	**0**	**0**	**3 (1)**

AE, adverse event; IRR, infusion-related reaction; SAE, serious adverse event. Incidences are expressed as number (%) of patients with each type of event. Percentages are based on the number of patients who received at least one infusion of ofatumumab for the respective treatment course. Ofatumumab treatment course is the course that the patient was receiving at the onset of the event. Infections were those events reported in the MedDRA System Organ Class of “Infections and infestations”. Neoplasms were those events reported in the MedDRA System Organ Class of “Neoplasms (benign, malignant and unspecified, including cysts and polyps)”.

**Table 4:** Incidence of Adverse Events by Ofatumumab Treatment Course in OFA110634.

	Ofatumumab Treatment Course							
	1	2	3	4	5	6	7	Overall
**OFA110634**	**N = 148**	**N = 93**	**N = 63**	**N = 30**	**N = 13**	**N = 6**	**N = 0**	**N = 148**
**Any AE**	**127 (86)**	**60 (65)**	**37 (59)**	**17 (57)**	**8 (62)**	**5 (83)**	**-**	**134 (91)**
IRR on day of first infusion	101 (68)	48 (52)	18 (29)	10 (33)	2 (15)	1 (17)	-	110 (74)
IRR on day of second infusion	1 (<1)	2 (2)	0	3 (10)	0	0	-	6 (4)
AE leading to discontinuation of investigational product/withdrawal	21 (14)	1 (1)	1 (2)	0	1 (8)	0	-	24 (16)
**Any SAE (fatal or non-fatal)**	**20 (14)**	**10 (11)**	**2 (3)**	**1 (3)**	**0**	**0**	**-**	**30 (20)**
Serious IRR	8 (5)	1 (1)	0	0	0	0	0	8 (5)
SAE leading to discontinuation of investigational product/withdrawal	8 (5)	0	1 (1)	0	0	0	-	9 (6)
Death	0	0	0	0	0	0	-	0
**Any infection**	**30 (20)**	**20 (22)**	**12 (19)**	**4 (13)**	**3 (23)**	**3 (50)**	**-**	**48 (32)**
Most common infections (≥5% overall)								
Gastroenteritis	4 (3)	3 (3)	1 (1)	0	1 (8)	0	-	9 (6)
Urinary tract infection	4 (3)	4 (4)	2 (3)	0	0	0	-	9 (6)
Bronchitis	3 (2)	1 (1)	0	3 (10)	0	0	-	7 (5)
Nasopharyngitis	6 (4)	2 (2)	1 (1)	0	0	0	-	7 (5)
**Any serious infection**	**2 (1)**	**5 (5)**	**0**	**0**	**0**	**0**	**-**	**7 (5)**
Urinary tract infection	1 (<1)	1 (1)	0	0	0	0	-	2 (1)
Cellulitis	0	1 (1)	0	0	0	0	-	1 (<1)
Gastroenteritis	0	1 (1)	0	0	0	0	-	1 (<1)
Herpes oesophagitis	0	1 (1)	0	0	0	0	-	1 (<1)
Oesophageal candidiasis	0	1 (1)	0	0	0	0	-	1 (<1)
Oral candidiasis	1 (<1)	0	0	0	0	0	-	1 (<1)
Respiratory tract infection	0	1 (1)	0	0	0	0	-	1 (<1)
**Any neoplasm**	**3 (2)**	**1 (1)**	**0**	**0**	**0**	**0**	**-**	**3 (2)**
**Any serious malignant neoplasm**	**0**	**0**	**0**	**0**	**0**	**0**	**0**	**0**

AE, adverse event; IRR, infusion-related reaction; SAE, serious adverse event. Incidences are expressed as number (%) of patients with each type of event. Percentages are based on the number of patients who received at least one infusion of ofatumumab for the respective treatment course. Ofatumumab treatment course is the course that the patient was receiving at the onset of the event. Infections were those events reported in the MedDRA System Organ Class of “Infections and infestations”. Neoplasms were those events reported in the MedDRA System Organ Class of “Neoplasms (benign, malignant and unspecified, including cysts and polyps)”.

**Table 5:** Incidence of Adverse Events by Ofatumumab Treatment Course in OFA111752.

	Ofatumumab Treatment Course							
	1	2	3	4	5	6	7	Overall
**OFA111752**	**N = 92**	**N = 73**	**N = 53**	**N = 20**	**N = 8**	**N = 6**	**N = 2**	**N = 92**
**Any AE**	**49 (53)**	**45 (62)**	**26 (49)**	**8 (40)**	**3 (38)**	**1 (25)**	**0**	**70 (76)**
IRR on day of first infusion	29 (32)	20 (27)	13 (25)	4 (20)	1 (13)	0	0	42 (46)
IRR on day of second infusion	2 (2)	0	2 (4)	0	0	0	0	4 (4)
AE leading to discontinuation of investigational product/ withdrawal	3 (3)	0	0	0	0	0	0	3 (3)
**Any SAE (fatal or non-fatal)**	**7 (8)**	**4 (5)**	**0**	**0**	**0**	**0**	**0**	**11 (12)**
Serious IRR	1 (1)	0	0	0	0	0	0	1 (1)
SAE leading to discontinuation of investigational product/withdrawal	1 (1)	0	0	0	0	0	0	1 (1)
Death	0	0	0	0	0	0	0	0
**Any infection**	**18 (20)**	**24 (33)**	**10 (19)**	**5 (25)**	**2 (25)**	**0**	**0**	**40 (43)**
Most common infections (≥5% overall)								
Upper respiratory tract infection	5 (5)	6 (8)	5 (9)	1 (5)	0	0	0	14 (15)
Rhinitis	4 (4)	1 (1)	0	1 (5)	1 (13)	0	0	5 (5)
Urinary tract infection	1 (1)	3 (4)	1 (2)	1 (5)	0	0	0	5 (5)
**Any serious infection**	**0**	**1 (1)**	**0**	**0**	**0**	**0**	**0**	**1 (1)**
Pneumonia	0	1 (1)	0	0	0	0	0	1 (1)
**Any neoplasm**	**0**	**0**	**0**	**0**	**0**	**0**	**0**	**1 (1)**^a^
**Any serious malignant neoplasm**	**0**	**0**	**0**	**0**	**0**	**0**	**0**	**0**

AE, adverse event; IRR, infusion-related reaction; SAE, serious adverse event. Incidences are expressed as number (%) of patients with each type of event. Percentages are based on the number of patients who received at least one infusion of ofatumumab for the respective treatment course. Ofatumumab treatment course is the course that the patient was receiving at the onset of the event. Infections were those events reported in the MedDRA System Organ Class of “Infections and infestations”. Neoplasms were those events reported in the MedDRA System Organ Class of “Neoplasms (benign, malignant and unspecified, including cysts and polyps)”.

^a^One neoplasm in OFA111752 could not be assigned to a treatment course as the start date was not recorded.

### Infusion-related reactions

IRRs represented the most common AEs overall, occurring with higher incidence on the day of the first infusion of the first ofatumumab treatment course, consistently across the three trials (Tables 3–5). IRRs reported on the day of the second infusion decreased considerably and became less frequent with subsequent treatment courses. The most common symptoms were rash, urticaria, throat irritation, cough and pruritus. IRRs were mostly of mild-to-moderate intensity. SAEs due to IRRs were reported more frequently in study OFA110634 which enrolled patients with a previous exposure to biologic therapy (5% [8/148] in OFA110634 vs. <1% [1/243] in OFA110635 and 1% [1/92] in OFA111752), and generally occurred during the first infusion of the first treatment course (Tables 3–5).

### Infections

Overall, 53% (130/243), 32% (48/148) and 43% (40/92) of patients experienced an infection at any time in study OFA110635, OFA110634 and OFA111752, respectively; the percentage of patients experiencing infections generally remained stable over time and over repeated ofatumumab treatment courses (Tables 3–5). The commonly reported non-serious infections were nasopharyngitis, upper respiratory tract infections, bronchitis, urinary tract infections and, in study OFA110634, gastroenteritis. Overall, 3% (8/243), 5% (7/148) and 1% (1/92) of patients experienced serious infections in OFA110635, OFA110634 and OFA111752, respectively. No cases of tuberculosis were reported even from sites in countries with high prevalence of tuberculosis (at the time these trials recruited patients) such as Peru, Argentina, Republic of Korea, Russian Federation, Poland and Romania. No PML case was reported and opportunistic infections were very rare.

No cases of HBV reactivation occurred. A single case of *de novo* fulminant hepatitis B, which was fatal and assessed as related to study drug by the investigator, occurred in OFA110635 after the third ofatumumab retreatment. This 54-year-old Hungarian woman, biologic-naïve and concurrently treated with oral MTX (20 mg, weekly), tested consistently negative for HBsAg, HBV surface antibody, HBV core antibody and HCV at study entry and up to the fulminant onset of HBV infection. The genotype “D” of the HBV isolated from this patient had both precore (G1896A) and basal core promoter mutations (A1762T and G1764A) which are associated with a high incidence of fulminant outcome. Whether ofatumumab contributed to the fulminant course, through potentially diminishing the humoral immune response to acute HBV infection following sustained B-cell depletion in this patient remains unclear.

### Neoplasms

Overall, ≤2% of patients had a benign or malignant neoplasm across the studies (Tables 3–5). Serious malignant neoplasms were reported for 3 (1%) patients in OFA110635: a grade 3B-cell lymphoma developed in a 53 year old woman after ofatumumab treatment course 4; a gingival cancer in a 61 year old man after ofatumumab treatment course 2, and an ovarian cancer in a 56 year old woman after treatment course 3. No metastases or fatalities occurred. Other reported neoplasms were benign.

### Deaths

There were no deaths in studies OFA110634 and OFA111752. In study OFA110635 two patients had a fatal SAE while on ofatumumab treatment: interstitial lung disease (during the first treatment course, not considered by the investigator to be related to study drug) and the aforementioned fulminant HBV infection. In addition, three fatal SAEs occurred during the safety follow-up period of study OFA110635: acute pyelonephritis, cerebrovascular disorder and pancreatic necrosis; none of these SAEs were considered by the investigators to be related to ofatumumab.

### Pregnancies

One unplanned pregnancy was reported in OFA110635 and two were reported in OFA110634. The outcomes were a blighted ovum/spontaneous abortion (unrelated to ofatumumab) in OFA110635, an elective termination and a healthy infant, respectively, in OFA110634.

### Laboratory findings

**Fig 2:**
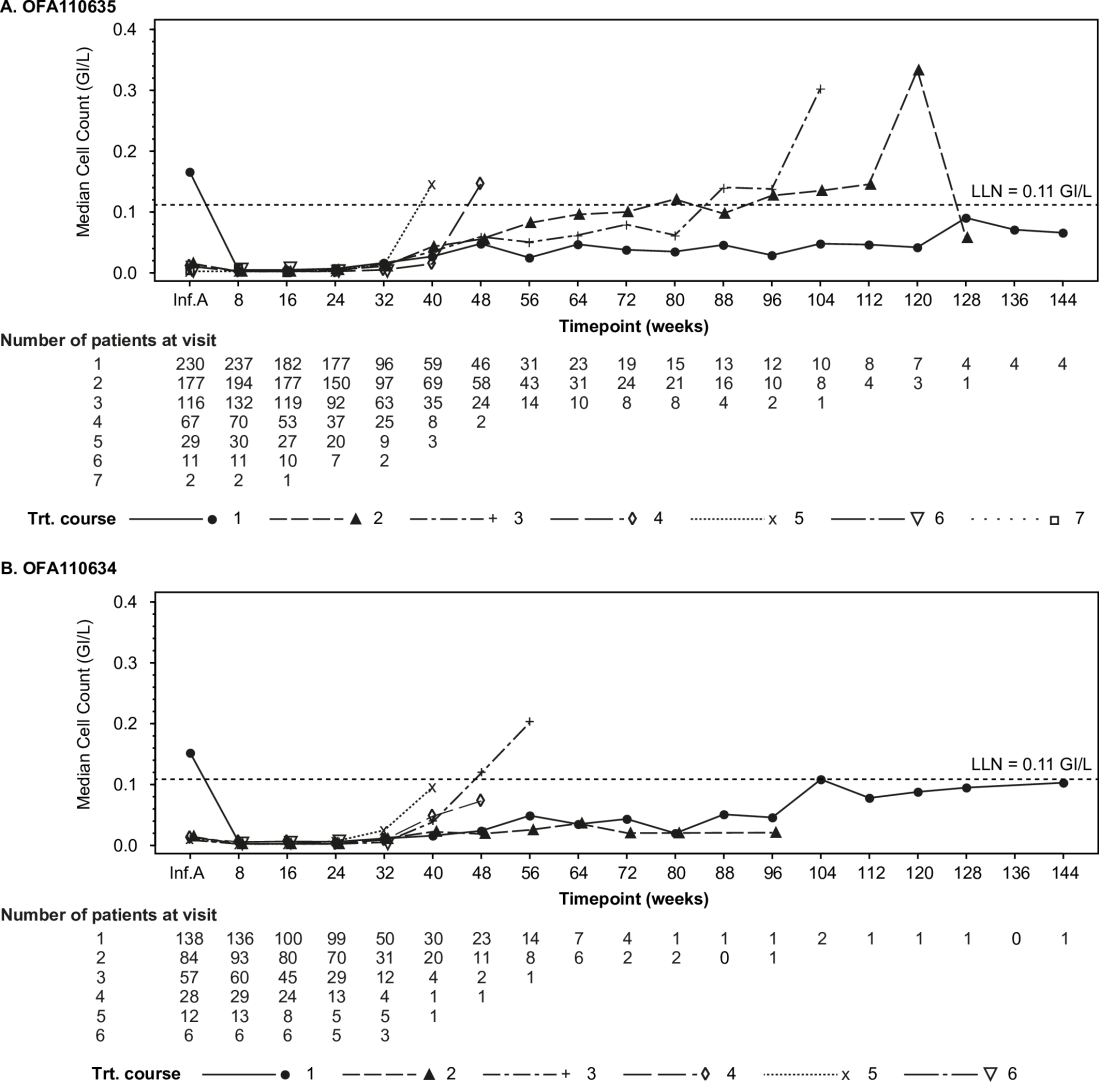
Median CD19+ B-cell Counts by Ofatumumab Treatment Course. Line plots show median B-cell CD19+ (GI/L) counts by week for each treatment course in OFA110635 (panel A) and OFA110634 (panel B). The data for OFA111752 were incomplete and have not been plotted. Inf. A, infusion A, was the first infusion of each treatment course; LLN, lower limit of normal = 0.11 GI/L; Trt, treatment.

Following the first infusion of ofatumumab, peripheral B-lymphocytes (CD19^+^) were greatly reduced relative to pre-study (Fig 2). Retreatment was based on clinical response rather than on B-cell counts, and peripheral B-lymphocytes were still depleted in most patients at the time they needed retreatment. However, there was a tendency for B-lymphocytes to start repopulating earlier with increasing number of treatment courses in those patients who required multiple retreatments (Fig 2). No changes in peripheral CD3^+^, CD4^+^ or CD8^+^ T-cell counts were noted.

In OFA110635, the proportion of patients with a B-lymphocyte stimulator (BLyS) value above the upper limit of normal increased from 6% (12/203) at baseline to 65% (84/130) at week 8 and 75% (114/152) at week 24 of treatment course one. Similar results were observed in OFA111752, with 13% (12/92) of patients having raised BLyS levels at baseline, 62% (54/87) at week 8 and 65% (48/74) at week 24 of treatment course 1.

IgG values <LLN at some time during treatment were reported for 9% (23/243), 4% (6/148) and 10% (9/92) of patients in OFA110635, OFA110634 and OFA111752, respectively. There did not appear to be a pattern for the association of infections and low IgG levels. However, per protocol patients with IgG levels falling below LLN at any time during the retreatment period could not receive further ofatumumab courses and proceeded to the safety FU phase.

Grade 3 or 4 neutropenia, i.e. neutrophils <1,000 to 500/mm^3^ or neutrophils <500/mm^3^ respectively based on the National Cancer Institute Common Terminology Criteria for Adverse Events (NCI-CTCAE), was reported for 3% (7/243), <1% (1/148) and 3% (3/92) of patients in OFA110635, OFA110634 and OFA111752, respectively; one of these patients (in OFA110635) had a temporally associated non-serious infection of nasopharyngitis. Grade 3 thrombocytopenia, i.e. platelets <50,000 to 25,000/mm^3^, was reported for 1 (<1%) patient in OFA110634.

In OFA111752 all 92 patients tested were negative for ofatumumab drug-induced antibodies.

### Clinical response

**Fig 3:**
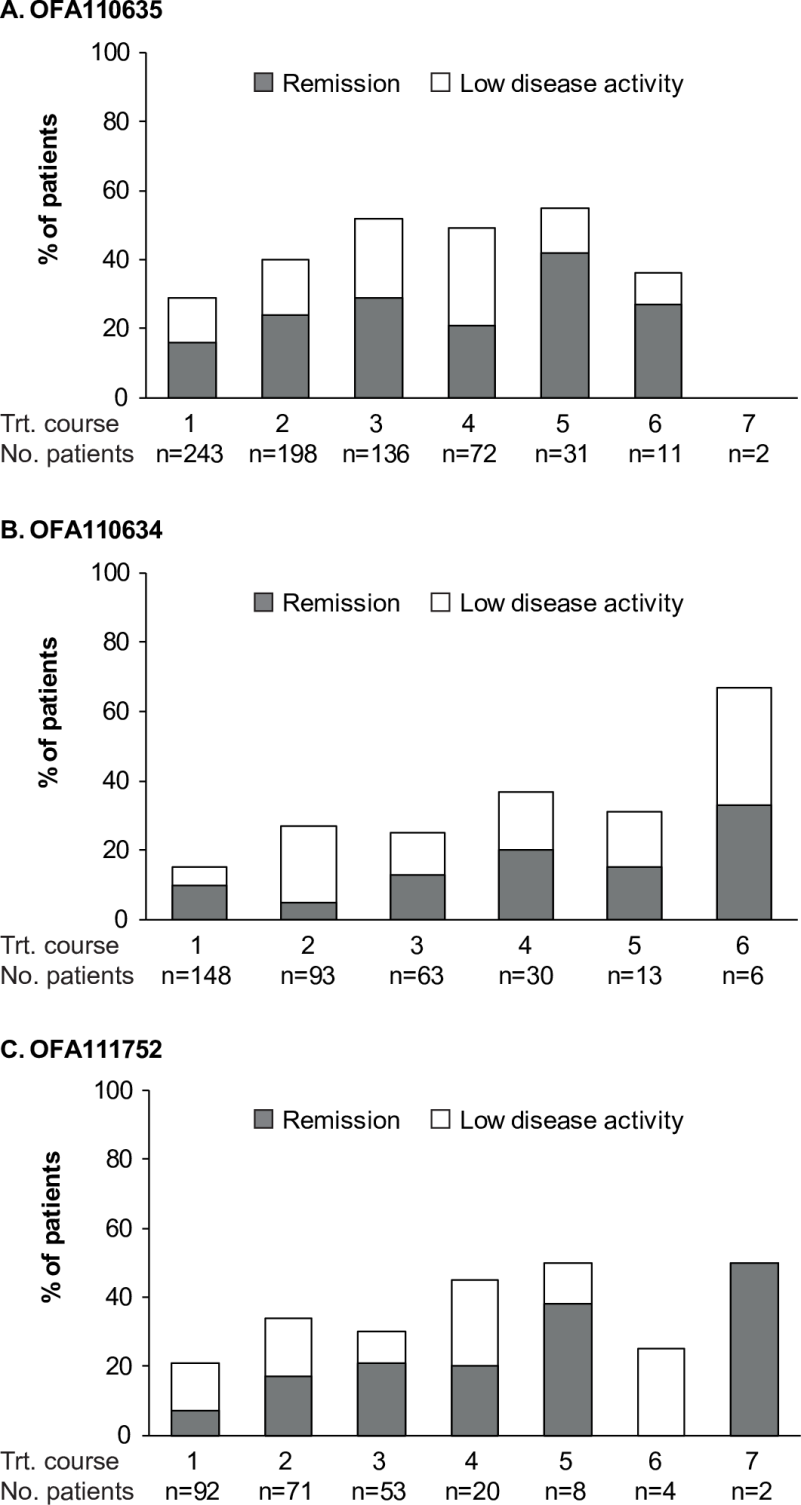
Summary of Disease Remission and Low Disease Activity Based on DAS28-ESR. Bars show the percentage of patients who achieved disease remission (DAS28-ESR <2.6; shaded portion of each bar) or low disease activity (DAS28-ESR ≥2.6 to <3.2; non-shaded portion of each bar) by ofatumumab treatment course in OFA110635 (panel A), OFA110634 (panel B) and OFA111752 (panel C).

Patients who were retreated derived benefit as shown by an improvement in DAS28-ESR. The percentage of patients who achieved disease remission or low disease activity (excluding data for treatment courses where the number of patients was <10) ranged from approximately 30–55% in OFA110635, 15–40% in OFA110634, and 20–45% in OFA111752, and remained relatively stable across treatment courses (Fig 3 and S3 Table).

## Discussion

The main goal of the clinical development of ofatumumab in RA was to optimise B-cell depletion therapy by administering a human anti-CD20 mAb in order to achieve sustained efficacy with higher disease remission rates over time and potentially improve patient safety. The success of this therapeutic approach relied on the ability to induce lasting efficacy without exposing patients to undue safety risks potentially caused by prolonged immunodeficiency. The question of greatest importance and uncertainty, when these trials were designed, was whether the persistent absence of circulating B-lymphocytes would lead to a clinically significant immunodeficiency with increased risk of developing serious infections or malignancies. Another issue was establishing the correct timing for retreatment, and whether a fixed retreatment regimen every 6 months or a more flexible approach based on clinical need would provide the optimal benefit/risk paradigm.

Data obtained from the open-label retreatment periods of the ofatumumab RA trials show that B-cell depletion therapy administered on clinical need is effective and generally safe in a clinical trial setting. Previously reported data from the double-blind and placebo-controlled phases of these trials highlighted higher rates of mild-to-moderate first dose IRRs observed with a single course of ofatumumab (despite steroid premedication) [13,14] compared to other intravenously administered anti-CD20s. In the current analysis of multiple treatment courses, first dose IRRs occurred at higher rate in patients previously exposed to a biologic DMARD in study OFA110634. However, the number of IRRs was low and the overall benefit/risk profile of ofatumumab remained generally favourable.

Opportunistic infections occurred rarely. Patients were closely monitored for signs of PML, a fatal opportunistic infection caused by the JC virus in immunocompromised patients, and no cases were reported. A study examining the aggregate experience of PML reported in association with autoimmune rheumatic diseases (ARDs) in the FDA Adverse Event Reporting System database [18] revealed 14 confirmed cases of PML in ARDs patients treated with rituximab, six of whom received rituximab for the treatment of RA.

Overall, serious infections were reported in ≤5% of patients across multiple ofatumumab retreatments. This was consistent with previous analyses on the long-term safety of rituximab retreatment across multiple RA populations [19,20]. The incidence of infections of any kind reported with rituximab in the international literature is 40% and it is similar to placebo in clinical trials of at least 6 months duration; serious infections in rituximab clinical trials have ranged between 2 and 7%, similar to placebo groups [21]. In our three clinical trials of ofatumumab in RA, serious infections were similar, ranging between 1 and 5%. In contrast, clinical trials of ocrelizumab, a humanised anti-CD20 mAb, in RA were prematurely terminated by the sponsor due to an increased incidence of serious infections which occurred at the highest, 500 mg X 2, dose tested [22]; this prompted termination of the clinical development of ocrelizumab in RA. In particular, patients recruited in Asia (Japan) showed a higher risk of developing serious infections [23] compared to patients recruited outside of Asia. This tendency was not observed in the ofatumumab RA programme where duration of drug exposure was longer than in the ocrelizumab programme but recruited a minority of Asian patients from Korea (24 patients,19%) only in study OFA110634. Apart from one serious case of gastroenteritis, during ofatumumab treatment course two, no other serious infections occurred in Asian patients. No cases of HBV reactivation were reported; however, the role played by repeated ofatumumab treatments in the *de novo* fatal case of fulminant hepatitis B in a Hungarian patient, the only case reported across the entire RA programme, remains unclear. Prevalence of HBV infection (HBsAg) in Hungary is reportedly low at <0.5% [24]; however, this may be an under-estimate as high-risk groups and migrants are often under-represented. In the rituximab REFLEX trial [25] one SAE of *de novo* HBV infection was reported after a single treatment course which resolved without sequelae. Isolated cases of reactivation of hepatitis B (including occult hepatitis B infection) are reported in RA patients receiving rituximab in the literature [26–28]. None of these cases came from high prevalence areas such as Asian countries. In the Autoimmunity and Rituximab (AIR) Registry, which provides to our knowledge the largest registry of infections in RA patients treated with rituximab, 1,303 patients were treated with 1000 mg X 2 IV infusions of rituximab in French clinical practice settings [21]. Of these, 712 patients had received retreatment at the time of the analysis: 466 with 2 cycles, 176 with 3 cycles, 45 with 4 cycles and 25 with ≥ 5 cycles. Data from the AIR registry showed a slightly higher percentage of serious infections than what previously reported in clinical trials, with 78 out of the 712 patients who were retreated developing 82 severe infections (11%). A slightly higher rate of serious infections in a “real life” clinical practice setting is to be expected, as in clinical trials several restrictions to patients’ eligibility apply. However, it is noteworthy that overall there seems to be little evidence to date that RA patients whose B cells are persistently depleted are at a significantly higher risk of developing serious infections [29–31].

Ofatumumab treatment was efficacious across distinct RA patient populations, i.e., patients with an inadequate response to DMARDs (study OFA111752), biologic-naïve patients with an inadequate response to MTX (study OFA110635), and patients refractory to TNF antagonists (study OFA110634), and was able to induce low disease activity and disease remission (based on DAS28-ESR), as also observed previously with repeated rituximab treatment [29, 30]. Intervals between treatments based on patients’ clinical need tended to be longer than 6 months in the earlier cycles of the retreatment phase. It is important to note, however, that due to the early termination of these studies by the sponsor, patients recruited later in the trials were withdrawn prematurely, limiting information available on longer term treatment with ofatumumab.

Increasingly elevated BLyS plasma levels were detected in patients administered additional courses of ofatumumab, consistent with its function as B-cell survival factor, also in line with rituximab data [32]. B-lymphocytes seemed to repopulate earlier in patients requiring several retreatments. This could be explained by the increased BLyS plasma levels inducing peripheral B-cells survival following initial depletion, by the selection of a more difficult-to-treat subpopulation of RA patients, or by the development of anti-drug antibodies which may reduce ofatumumab’s effectiveness.

Some important limitations of the present analysis need to be considered. Because of premature termination, OFA110634 in anti-TNF refractory patients did not enrol the planned number of patients and none of the pre-specified statistical analyses for the double-blind or open-label periods were performed. Additionally, as the open-label period of OFA110635 and study OFA111752 were also prematurely terminated, data have been reported using descriptive statistics only. The majority of patient withdrawals occurred because of premature trial termination by the sponsor, so it is not possible to know if those patients would have experienced safety issues with further ofatumumab treatment courses over time. There was only limited testing of anti-drug antibodies, therefore we are unable to confirm if repeated treatment cycles of ofatumumab were immunogenic or not in RA patients (although the samples which were tested were negative for anti-ofatumumab antibodies).

## Conclusions

In conclusion, individualised retreatment with intravenous ofatumumab, at a dose of 700 mg X 2 infusions, was efficacious and generally safe in active RA patients with no major differences observed across trials recruiting either biologic-naive, DMARDs-refractory, or patients previously exposed to TNF-inhibitors. Ofatumumab remains in clinical development for autoimmune diseases as a subcutaneous formulation.

## Supporting Information

S1 CONSORT Checklist (DOCX)

S1 Protocol Protocol for study OFA110635. (PDF)

S2 Protocol Protocol for study OFA110634. (PDF)

S3 Protocol Protocol for study OFA111752. (PDF)

S1 Table OFA110635—Safety of placebo and ofatumumab over the 24 weeks double-blind period (safety population). (DOCX)

S2 Table OFA110634 –Safety of placebo and ofatumumab over the 24 weeks double-blind period (safety population). (DOCX)

S3 Table Number (%) of patients who achieved Remission or Low Disease Activity based on DAS28 (using ESR) during the Double-Blind or Open-Label Periods, by Treatment Course (As Treated Population). (DOCX)
