# Supplementary material for: Safety of Repeated Open-Label Treatment Courses of Intravenous Ofatumumab, a Human Anti-CD20 Monoclonal Antibody, in Rheumatoid Arthritis: Results from Three Clinical Trials
Source: PLoS One. 2016 Jun 23;11(6):e0157961. doi: 10.1371/journal.pone.0157961 (PMC4919033; doi:10.1371/journal.pone.0157961)
Supplement: S2 Protocol — (PDF) [file pone.0157961.s003.pdf]

The GlaxoSmithKline group of companies

INTEGRATED CLINICAL TRIAL PROTOCOL

A double-blind, randomized, placebo controlled, parallel group, multi-center, phase III trial of ofatumumab investigating clinical efficacy in adult patients with active rheumatoid arthritis who had an inadequate response to TNF-α antagonist therapy

Investigating clinical efficacy of ofatumumab in adult RA patients who had an inadequate response to TNF-α antagonist therapy

Confidential

Final:	21-Jun-2007
Trial ID:	GEN411/OFA110634
Amendment 1:	23 October 2007
Amendment 2:	01 February 2008
Amendment 3:	13 May 2008
Amendment 4:	14 September 2010
Amendment 5:	22 October 2010

This Study Protocol is the property of GSK and is a confidential document. It is not to be copied or distributed to other parties without written approval from GSK.

Table of Contents

Table of Contents 2
List of Tables 5
List of Figures 5
1 Protocol Summary 7
1.1 Primary Objective 7
1.2 Secondary Objectives 7
1.3 Primary Endpoint 7
1.4 Secondary Endpoints 7
1.5 Trial population 8
1.6 Number of Patients 9
1.7 Trial Design 9
1.8 Investigational Medicinal Product 11
2 Flow Chart 12
3 Background and Rationale 18
3.1 Disease & Treatment 18
3.1.1 Rheumatoid Arthritis 18
3.1.2 Current Treatment of Rheumatoid Arthritis 19
3.2 Investigational Medicinal Product 20
3.2.1 Ofatumumab 20
3.2.2 Pre-clinical Pharmacology 21
3.2.3 Pre-clinical Safety 22
3.2.4 Previous Clinical Experience 23
3.2.5 Summary of known and potential risks to human subjects 24
3.3 Study rationale 24
3.3.1 Study population 25
3.3.2 Dose 25
3.3.3 Dose Schedule & Re-treatment 26
3.3.4 Pre-medication 28
4 Objectives 29
4.1 Primary Objective 29
4.2 Secondary Objectives 29
4.3 Primary Endpoint 29
4.4 Secondary Endpoints 29
5 Patient Selection and Withdrawal 30
5.1 Inclusion Criteria 30
5.2 Exclusion Criteria 31
5.3 Withdrawal Criteria 34
5.3.1 Withdrawal from treatment 34
5.3.2 Withdrawal from trial 36
5.3.3 Withdrawal from Safety Follow-up 36
6 Trial Design 37
6.1 Overall Design 37
6.2 Schedule of Events 39
6.3 Screening (Visit 1) 39
6.4 Treatment 40
6.4.1 Double-blind Period (Visit 2 to Visit 9) 40
6.4.2 Open-label Period (Visit 9 and forwards) 41
6.5 Follow-up Period 42
6.6 Treatment after the End of the Study 43

6.7 Recruitment period....43
6.8 Number of Patients 43
6.9 Randomization 43
6.10 Blinding 44
6.11 Emergency Unblinding procedure 44
7 Treatment 45
7.1 Investigational Medicinal Product (IMP)....45
7.1.1 Ofatumumab....45
7.1.2 Packaging and Labeling of ofatumumab/placebo 45
7.1.3 Storage of ofatumumab 45
7.1.4 Drug Accountability and Compliance Check....46
7.2 Dosage of ofatumumab/placebo....46
7.3 Handling of ofatumumab/placebo....47
7.3.1 Pre-Medication....47
7.3.2 Preparation of ofatumumab/placebo 47
7.3.3 Treatment Schedule....48
7.3.4 Administration of ofatumumab/placebo....48
7.4 Concomitant Therapy....50
7.4.1 Therapy Allowed during Trial....50
7.4.2 Prohibited therapy and procedures during the study 52
8 Trial Assessments 53
8.1 Clinical Assessments 53
8.1.1 Demographics 53
8.1.2 Medical History....53
8.1.3 Height and Body Weight....53
8.1.4 Physical Examination....53
8.1.5 Neurological Questionnaire....53
8.1.6 Electrocardiogram 54
8.1.7 Vital Signs....54
8.1.8 Adverse Events 54
8.1.9 Concomitant Medication....54
8.1.10 Administration of IMP 54
8.1.11 Chest X-Ray....54
8.1.12 Mantoux test....55
8.1.13 Clinical Efficacy Assessments 55
8.1.14 Patient Reported Outcomes 57
8.2 Laboratory Assessments 58
8.2.1 Biochemistry, Hematology and Urine test 58
8.2.2 Pregnancy Test....59
8.2.3 IgA, IgG, IgM 59
8.2.4 Flow Cytometry 59
8.2.5 JC Virus 60
8.2.6 Host Immune Response (HAHA) 60
8.2.7 Pharmacokinetics (PK)....60
8.2.8 Hepatitis B and C 60
8.2.9 Biomarkers....61
8.2.10 Transcriptomics....61
8.2.11 Pharmacogenetics....61
8.2.12 ACR/DAS28: Laboratory Efficacy Assessments (CRP and ESR)....62
9 Adverse Events....63
9.1 Characterization....63
9.1.1 Definition of Adverse Events....63
9.1.2 Pre-existing Conditions....63
9.1.3 B cell depletion and hypogammaglobulinemia 63

9.1.4 Study Disease 63
9.1.5 Procedures 64
9.1.6 Adverse Event Reporting 64
9.1.7 Pregnancy 64
9.2 Reporting Instructions 64
9.2.1 Recording 64
9.2.2 Diagnosis 65
9.2.3 Intensity 65
9.2.4 Relationship to Study Drug 65
9.2.5 Outcome 65
9.3 Serious Adverse Events (SAE) 66
9.3.1 Definition of a SAE 66
9.3.2 Reporting of SAEs 66
9.3.3 Suspected Unexpected Serious Adverse Reactions (SUSARs) 67
9.4 Follow-Up on Adverse Events 67
9.5 Safety Monitoring 68
9.5.1 Safety Surveillance set up 68
9.5.2 Liver Chemistry Abnormalities 68
9.5.3 Infections and malignancies 70
9.5.4 Critical Adverse Event 71
9.5.5 Stopping Rules 71
10 Statistics 73
10.1 Analysis Populations 73
10.1.1 Intent-to-treat (ITT) 73
10.1.2 Per Protocol (PP) 73
10.1.3 Safety 74
10.2 Statistical Analysis of Primary Endpoint 74
10.2.1 ACR20 74
10.2.2 Handling of missing values and use of rescue medication 75
10.3 Statistical Analysis of Secondary Endpoints 75
10.3.1 ACR50 and ACR70 75
10.3.2 ACRn 75
10.3.3 DAS28 76
10.3.4 EULAR 76
10.3.5 Clinical Remission 77
10.3.6 HAQ-DI Responders 77
10.4 Safety 77
10.4.1 Adverse Events 77
10.4.2 Vital Signs 78
10.4.3 Laboratory Safety Data 78
10.4.4 ECG 78
10.4.5 Immunoglobulin levels 78
10.4.6 Flow cytometry 78
10.4.7 Host Immune Response 78
10.5 Patient reported outcomes 78
10.5.1 HAQ-DI 78
10.5.2 SF-36 78
10.5.3 FACIT-F 79
10.6 Pharmacokinetics 79
10.7 Biomarkers / Transcriptomics 79
10.8 Handling of Missing Data, data recorded after possible treatment with rescue therapy or Outliers 79
10.9 Subgroups and Center Effects 79
10.10 Analysis of Open-Label data 80
10.11 Determination of Sample Size 80

11 Ethics.....81
11.1 Independent Ethics Committee (IEC) or Institutional Review Board (IRB).....81
11.2 Patient Information and Informed Consent.....81
11.3 Ethical and Risk/Benefit Considerations.....81
12 Monitoring and Quality Assurance (QA).....83
12.1 Compliance with Good Clinical Practice.....83
12.2 Monitoring.....83
12.3 Source Data Verification.....83
12.4 Study and Site Closure.....84
13 Data Handling and Record Keeping.....85
13.1 Data Management.....85
13.2 Archiving of Trial Documents at Site.....85
14 Reporting and Communication of Results.....87
14.1 Use of Information.....87
14.2 Provision of Study Results to Investigators, Posting to the Clinical Trials Register and Publication.....87
15 Insurance/Liability/Indemnity.....88
16 Changes to the Final Protocol.....89
17 Premature Termination of the Trial.....90
17.1 Premature Termination of a Trial Site.....90
18 List of abbreviations and definitions of terms.....91
18.1 List of abbreviations.....91
18.2 Definition of Terms.....94
19 References.....95
Appendices.....99
Appendix 1: Rheumatoid Arthritis Functional Class.....99
Appendix 2: Pharmacogenetic assessment.....100

List of Tables

Table 1 : Ofatumumab/placebo dosages.....47
Table 2: Pre-medication requirements before each infusion of ofatumumab/placebo.....47
Table 3: Ofatumumab infusion preparation.....47
Table 4 : Infusion rate.....48
Table 5: Measurements and PK sampling during infusion.....49
Table 6: Maximum doses for i.a. corticosteroids.....52
Table 7: Definition of rescue medication.....52
Table 8 : Timelines for reporting SAEs, pregnancies and liver function abnormalities.....67
Table 9 : Limits of CAE with error and detection rates.....72
Table 10: EULAR Response Conversion Table.....77

List of Figures

Figure 1: Trial Design.....10
Figure 2: Trial Design.....37

1 Protocol Summary

Trial ID: GEN411/OFA110634

Title: A double-blind, randomized, placebo controlled, parallel group, multi-center, phase III trial of ofatumumab investigating clinical efficacy in adult patients with active rheumatoid arthritis who had an inadequate response to TNF-α antagonist therapy

Short Title: Investigating clinical efficacy of ofatumumab in adult RA patients who had an inadequate response to TNF-α antagonist therapy

1.1 Primary Objective

- To demonstrate the efficacy of ofatumumab in reducing clinical signs and symptoms in adult RA patients after a single course of ofatumumab

1.2 Secondary Objectives

- To evaluate long-term efficacy of repeated courses of ofatumumab
- To evaluate ofatumumab with respect to impact on patient reported outcomes after single and repeated courses of ofatumumab
- To evaluate the risk of host immune response against ofatumumab after single and repeated courses of ofatumumab
- To evaluate the safety of ofatumumab after single and repeated courses of ofatumumab

1.3 Primary Endpoint

- ACR20 at 24 weeks

1.4 Secondary Endpoints

Key Secondary Endpoints:

- ACR20, ACR50, ACR70 and ACRn
- DAS28
- EULAR response

Other Secondary Endpoints:

- Biomarkers of disease activity, immune status, and whole blood transcriptional profiles
- Short-Form health survey (SF-36) and Functional Assessment of Chronic Illness Therapy in Fatigue (FACIT-F)
- Human anti-human antibodies
- Adverse events, clinical laboratory parameters, vital signs, ECG
- Tender joint count (TJC)
- Swollen joint count (SJC)
- Patient VAS of pain
- Patient VAS of global disease
- Physician VAS of global disease
- Health Assessment Questionnaire (HAQ-DI)
- C-reactive protein (CRP) and Erythrocyte sedimentation rate (ESR)

1.5 Trial population

1) Age ≥ 18 years

2) A diagnosis of rheumatoid arthritis according to the American College of Rheumatology (ACR1987 classification) of at least six months' duration from diagnosis at screening

3) Active disease at the time of screening as defined by:
   - ≥ 8 swollen joints (of 66 joints assessed)
   and
   - ≥ 8 tender joints (of 68 joints assessed)
   and
   - C-Reactive Protein (CRP) ≥ 1.0 mg/dL or Erythrocyte Sedimentation Rate (ESR) ≥ 22 mm/hour
   and
   - DAS28≥3.2 (based on ESR)
   The joints will be reassessed at baseline (Visit 2) to confirm eligibility criteria are fulfilled. Where possible joint reassessment must be performed at the baseline visit (Visit 2); if this is not feasible, it can be performed ≤3 days prior to Visit 2

4) RA functional class I, II or III (For definition of RA functional class please refer to Appendix 1)

5) Inadequate response to previous or current TNF-α antagonist treatment after infliximab therapy (≥3 mg/kg; at least 4 infusions), or adalimumab therapy (40 mg at a minimum of every other week for ≥3 months), or etanercept therapy (25 mg twice weekly or 50 mg once a week for ≥3 months) defined as:

- Inadequate efficacy according to the investigator's judgment

  and/or

- Intolerance defined as one or more side effects following at least one administration of one of the TNF-α antagonists at the dose listed above that reasonably results in the discontinuation of treatment with the TNF-α antagonist treatment

6) Treatment with methotrexate (MTX), 7.5-25 mg/week, for at least 12 weeks and at a stable dose for at least 4 weeks prior to Visit 2. Doses of MTX as low as 7.5 mg per week are permitted for patients who could not tolerate higher doses

1.6 Number of Patients

A total of 236 patients to be randomized and treated in this study, with a target of 118 patients per treatment group. This sample size provides 90% power at a 5% level of significance, based on ACR20 response rates of 25% and 45% at week 24 in the MTX and MTX+ofatumumab treatment groups respectively. This is based on a Chi-square test comparing two binomial proportions. The response rates are based on the assumption that patients with missing ACR20 at week 24 will be considered as non-responders.

1.7 Trial Design

This is a phase III, double-blind, randomized, multicenter, and parallel group trial with a duration of 24 weeks, followed by a 120 week Open-label Period.

Eligible patients will be randomized in a 1:1 ratio to receive ofatumumab 700mg x 2 or placebo x 2 in addition to their background methotrexate treatment. The randomized treatment will be administered as two infusions, one at day 0 and one at day 14 (one treatment course) after randomization. Randomization will be stratified by rheumatoid factor seropositivity / -negativity, and region. Patients will not receive additional treatment courses with ofatumumab in the Double-blind Period. Rescue treatment will be allowed from week 16 but the use of rescue treatment will preclude subsequent entry into the Open-label period. Breakthrough pain management in the form of analgesics, NSAIDs and intra-articular corticosteroid injections will be allowed during the Double-blind and Open-label Periods. Use of intra-articular corticosteroid injections will be prohibited 4 weeks prior to the primary endpoint (i.e. Visit 9, week 24). One injection in one single joint per 6 month period will be permitted throughout the Double-blind and Open-label periods.

All patients (i.e., those who have received ofatumumab or placebo) who complete the 24 week Double-blind Period without receiving rescue DMARD treatment will be eligible to proceed into the 120 week Open-label Period to receive treatment courses with ofatumumab (700mg x 2 two weeks apart). In the Open-label Period ofatumumab treatment courses will be given at

individualized time intervals only if a clinical response (see Section 6.4.2 for definition of clinical response) has been achieved following the previous treatment course, and followed by a subsequent worsening in disease activity.

The interval between initiation of a new treatment course in the Open-label Period should be at least 16 weeks irrespective of progression in disease activity. Sixteen weeks is counted from infusion A from the preceding infusion course. The last treatment course should be planned to occur no later than the scheduled visit at week 120 from baseline (Visit 2).

In addition, any patient with a circulating IgG level <lower limit of normal (as assessed by the central laboratory) at any time after the second treatment course will be withdrawn from further ofatumumab treatment. Patients who have completed the Open-label Period or have been withdrawn will enter the Follow-up Period; it is anticipated the Follow-up period will be in the order of a 2 year period.

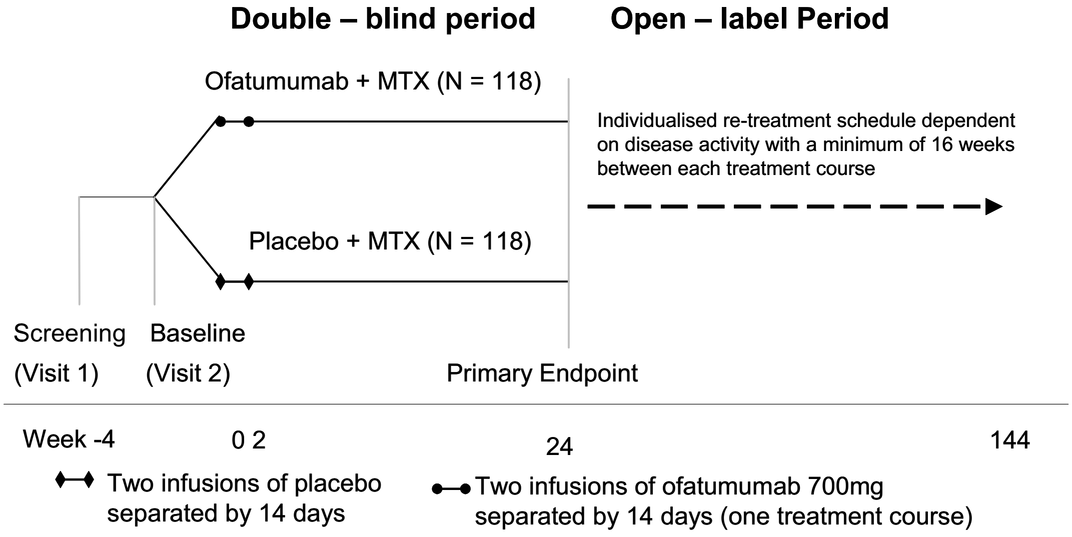

Figure 1: Trial Design

In the event the trial is prematurely terminated, all subjects in Double-blind or Open-label Periods will enter the Follow-Up Period at the next scheduled study visit.

1.8 Investigational Medicinal Product

Ofatumumab is a clear colorless liquid. Ofatumumab is a concentrate for solution intended for intravenous administration. It is intended for administration after dilution in 1000 mL sterile, pyrogen free 0.9% NaCl.

Ofatumumab will be filtered using an in-line filter (0.2 µm) during infusion.

The initial rate of the infusion will be 12 mL/hour for the first administration and 25 mL/hour for the second administration during each treatment course. During infusion, the rate will be doubled every 30 minutes to a maximum of 400 mL/hour. Thereafter, the rate will be increased by 200 mL/hour every 30 minutes until 800 mL/hour is reached. Duration of the infusion will be approximately 4 and 3½ hours respectively if this schedule is adhered to.

2 Flow Chart

	Screening[1]	Double-Blind Period								Open-Label Period[2]	Follow-Up
**CLINICAL ASSESSMENT[3]** (Assessments should be done prior to blood sampling and infusion unless stated otherwise)											
Period	28 days	24 weeks								120 weeks	Individualized
Visit Number	1	2	3	4	5	6	7	8	9 / early withdrawal	10+	FU+
Day/Week[4]	≤4w	0d	14d	4w	8w	12w	16w	20w	24w	Every 8 w	Every 12 w
Visit Window (days)	≤28 prior to v2	+1	±2	±3	±3	±3	±3	±3	±3	±14	±14
Informed Consent	X[5]										
Eligibility Criteria	X	X[6]									
Demographics	X										
Medical History	X										
Physical Examination	X	X				X			X	X	
Height and Weight		X				X[7]			X[6]	X[6,8]	

[1] Screen assessment to be completed within a maximum of 28 days prior to Baseline (Visit 2)
[2] The assessments should be done every 8 weeks (± 14 days) throughout the Open-label Period of the trial. If disease activity progresses between scheduled visits an unscheduled visit should be performed (same assessments to be performed as for a scheduled visit). If disease activity worsens and re-treatment criteria are fulfilled an infusion visit should be planned no later than 14 days from worsening of disease activity (please refer to separate Flow Chart). If possible, the infusion visit should be combined with a scheduled assessment visit.

[3] Details of assessment can be found in Section 8
[4] Visit dates are relative to the Baseline date (Visit 2) which, if required for practical reasons, may be conducted over two consecutive days, Day 0 and Day 1, with the infusion of ofatumumab / placebo being given on Day 1. All other visit dates should be relative to the day of the first infusion in the study.
[5] Informed consent can be obtained outside the screening visit window i.e. may be prior to screening date. Informed consent should be obtained prior to any study related activity (including DMARD wash-out)
[6] Swollen and tender joint counts should be re-assessed at baseline to confirm patients meet requirements for inclusion criterion 3. Patients can not be randomised before inclusion criterion 3 is fulfilled. Where possible joint count reassessment should be performed at the baseline (Visit 2); if this is not feasible it can be done ≤3 days prior to Visit 2.
[7] Body weight only
[8] Should only be assessed every 24 weeks i.e. at week 48, 72, 96 120 and 144

	Screening[1]	Double-Blind Period								Open-Label Period[2]	Follow-Up
**CLINICAL ASSESSMENT[3]** (Assessments should be done prior to blood sampling and infusion unless stated otherwise)											
Period	28 days	24 weeks								120 weeks	Individualized
Visit Number	1	2	3	4	5	6	7	8	9 / early withdrawal	10+	FU+
Day/Week[4]	≤4w	0d	14d	4w	8w	12w	16w	20w	24w	Every 8 w	Every 12 w
Visit Window (days)	≤28 prior to v2	+1	±2	±3	±3	±3	±3	±3	±3	±14	±14
Neurological Questionnaire	X			X	X	X	X	X	X	X	X
ECG	X								X	X[8]	
Vital Signs	X	X[9]	X[8]	X	X	X	X	X	X	X	
Adverse Events[10]	X	X	X	X	X	X	X	X	X	X	X[11]
Conc. Medication	X	X	X	X	X	X	X	X	X	X	X[12]
Administration of IMP		X	X								
X-ray (chest)	X[13]										
Mantoux test	X[14]										
Efficacy Assessments<br>- joint assessments[15]<br>- VAS Pain<br>- VAS Disease (patient)	X[16]	X		X	X	X	X	X	X	X	

[9] Vital signs should be assessed prior to infusion, every hour during infusion and every hour during the observation period see Table 5:
[10] SAEs related to study participation are reported from time of informed consent. All other SAEs are reported from Visit 2. AEs occurring between Visit 1 and Visit 2 should be recorded as Medical History
[11] Only Serious Adverse Events will be collected in the follow up period
[12] Only concomitant RA medication will be collected
[13] X-ray obtained ≤ 12 weeks prior to screening as part of routine practice can replace the screening assessment. However, if this previous x-ray is inconclusive and if clinically warranted the x-ray should be repeated at screening
[14] The Mantoux test is to be performed during the screening period. Patients who have documented BCG vaccination will be exempt.
[15] The joint assessment should be performed by an independent joint evaluator. Joints receiving an i.a. corticosteroid injection should be counted as swollen and tender for 12 weeks following the injection
[16] Joint assessments and patient VAS of global disease assessment only

	Screening[1]	Double-Blind Period								Open-Label Period[2]	Follow-Up
**CLINICAL ASSESSMENT[3]** (Assessments should be done prior to blood sampling and infusion unless stated otherwise)											
Period	28 days	24 weeks								120 weeks	Individualized
Visit Number	1	2	3	4	5	6	7	8	9 / early withdrawal	10+	FU+
Day/Week[4]	≤4w	0d	14d	4w	8w	12w	16w	20w	24w	Every 8 w	Every 12 w
Visit Window (days)	≤28 prior to v2	+1	±2	±3	±3	±3	±3	±3	±3	±14	±14
- VAS Disease (physician)<br>- HAQ											
SF-36		X					X		X	X[8]	
FACIT-F		X					X		X	X[8]	

	Screening[1]	Double-Blind Period								Open-Label Period[2]	Follow-Up
**LABORATORY ASSESSMENT[17]** (Samples to be drawn prior to infusion unless stated otherwise)											
Period	28 days	24 weeks								120 weeks	Individualized[18]
Visit Number	1	2	3	4	5	6	7	8	9 / early withdrawal	10+	FU+
Day/Week[19]	≤4w	0d	14d	4w	8w	12w	16w	20w	24w	Every 8 w	Every 12 w
Visit Window (days)	≤28 prior to v2	+1	±2	±3	±3	±3	±3	±3	±3	±14	±14
Biochemistry/hematology	X	X	X	X	X	X	X	X	X	X	X[20]
Urine test	X	X	X	X	X	X	X	X	X	X	
Pregnancy Test[21]	X	X	X	X	X	X	X	X	X	X[22]	X[23]
IgA, IgG, IgM	X	X				X			X	X	X[24]
Flow Cytometry		X	X	X		X			X	X	X[25]
Plasma/white cell JCV PCR	X					X			X	X[28]	X
ESR[26]	X	X		X	X	X	X	X	X	X	
CRP	X	X		X	X	X	X	X	X	X	
HAHA	X	X	X						X	X[28]	

[17] All analyses will be performed by a central laboratory unless stated otherwise. Details of parameters can be found in Section 8
[18] Patients will be followed until B-cells and circulating IgGs have returned to normal or baseline levels, or a maximum of 2 years from the last scheduled visit in the Double-blind or Open-label Period, whichever occurs earlier.
[19] Visit dates are relative to baseline date (Visit 2)
[20] During Follow-up biochemistry/hematology tests are ONLY to be performed at week 12 and 24 from the last scheduled visit in the Double-blind or Open-label Period.
[21] Pregnancy tests at screening, end of Double-Blind Period/early withdrawal and at end of Open-Label Period are based on serum-HCG. All other urine-pregnancy tests are done locally, if positive a confirmatory serum-HCG should be done
[22] In the Open-label period monthly urine pregnancy testing should be performed. If a visit is not scheduled, home urine testing must be performed
[23]. A urine pregnancy test is ONLY to be performed at week 12 and 24 from the last scheduled visit in the Double-blind or Open-label Period.
[24] All patients will be followed with regards to IgG levels.
[25] $CD19^+$ only
[26] ESR is measured locally utilizing the Westergren method

	Screening[1]	Double-Blind Period								Open-Label Period[2]	Follow-Up
**LABORATORY ASSESSMENT[17]** (Samples to be drawn prior to infusion unless stated otherwise)											
Period	28 days	24 weeks								120 weeks	Individualized[18]
Visit Number	1	2	3	4	5	6	7	8	9 / early withdrawal	10+	FU+
Day/Week[19]	≤4w	0d	14d	4w	8w	12w	16w	20w	24w	Every 8 w	Every 12 w
Visit Window (days)	≤28 prior to v2	+1	±2	±3	±3	±3	±3	±3	±3	±14	±14
PK		X[27]	X[27]	X		X		X	X		
Hepatitis B	X								X	X[28]	X[29]
Hepatitis C[30]	X										X[31]
Rheumatoid Factor (RF)	X[32]	X									
Transcriptomics		X									
Anti-Cyclic Citrullinated Peptide (anti-CCP)		X									
Other biomarkers (incl. BLyS)		X		X		X			X		
Pharmacogenetics (PGx)[33]		X									

[27] PK-sampling should be done prior to infusion and immediately after the end of infusion
[28] Should only be taken every 24 weeks, i.e. at week 48, 72, 96 120 and 144.
[29] During Follow-up Hep B test is to be performed every 24 weeks f or a maximum of 2 years from the last scheduled visit in the Double-blind or Open-label Period.
[30] If Hepatitis C antibody positive, a Hepatitis C RIBA® immunoblot assay should be reflexively performed on the same sample to confirm the results (See Section 5.2).
[31] During Follow-up Hep C test is to be performed every 24 weeks f or a maximum of 2 years from the last scheduled visit in the Double-blind or Open-label Period.
[32] Only rheumatoid factor (RF) seropositivity / - negativity is measured at screening. RF quantitative will be measured at all visits indicated in the Flow Chart
[33] The PGx sample may be collected at any visit during the trial. The PGx sample is not mandatory

**Individualized Infusion Visits**[34,35]		
**ASSESSMENT** [36] **(**Assessments and samples should be done/drawn prior to infusion unless stated otherwise)		
Visit Number	Infusion A	Infusion B
Week	Individualized	+ 2 weeks from Infusion A
Visit Window (days)	-	±2
Body weight	X	
Physical examination	X	
Vital signs[37]	X	X
Adverse Events	X	X
Concomitant Medication	X	X
Urine Pregnancy Test[38]	X	X
Efficacy Assessments<br>- Joint assessment[39]<br>- VAS Pain<br>- VAS Disease (patient)<br>- VAS Disease (physician)<br>- HAQ	X	
ESR[40]	X	
Administration of IMP	X	X
CRP	X	
HAHA	X	
PK[41]	X	X

[34] One treatment course equals Infusion A and Infusion B 14 days apart. When re-treatment criteria are fulfilled a treatment course should be planned no later than 14 days from worsening of disease activity. If possible the infusion visit can be combined with a scheduled assessment visit.
[35] There must be a minimum of 16 weeks between initiation of a new treatment course. The last treatment should be planned to occur no later than the scheduled visit 120 weeks after baseline (Visit 2)
[36] All analyses will be performed by a central laboratory unless stated otherwise. Details of assessments can be found in Section 8
[37] Vital signs should be assessed prior to infusion , every hour during infusion and every hour during the observation period
[38] Urine-pregnancy testing is done locally, if positive a serum-HCG should be done confirmatory
[39]The joint assessment should be performed by an independent joint evaluator. Joints receiving an i.a. corticosteroid injection should be counted as swollen and tender for 12 weeks following the injection
[40] ESR is measured locally utilizing the Westergren method
[41] PK-sampling should be done prior to infusion and immediately after end of infusion at the first, third and fifth re-treatment courses in the Open-label Period

3 Background and Rationale

3.1 Disease & Treatment

3.1.1 Rheumatoid Arthritis

Rheumatoid arthritis (RA) is a chronic systemic inflammatory disease which affects 0.8-1.0% of all populations. The etiology of RA remains unknown.

RA primarily affects diarthrodial joints in a symmetrical distribution with an additive disease evolution over years (1). The hallmark of the disease is damage and disruption of joint integrity, starting early in the course of RA. In most cases, bone erosions progress leading to irreversible joint deformities. It is estimated that around one third of patients have radiographic evidence of bone erosions at the time of diagnosis, and this increases to almost 60% two years after diagnosis (2). Unless the inflammatory processes are halted or controlled, the disease leads to substantial disability, co-morbidities, and in many cases also premature death (3).

About 80% of RA patients present with rheumatoid factor (RF) (i.e., anti-IgG-Fc antibodies) in their serum, and this is associated with a poor prognosis (4). Along with more recent discoveries of other autoantibodies, such as anti-cyclic citrullinated peptide (anti-CCP), the presence of autoantibodies strongly suggests that B-lymphocytes (B-cells) are involved in the pathogenesis of RA. Also, the observation, more than 20 years ago, of the presence of considerable numbers of cells of the B-cell lineage in the rheumatoid synovium, including RF-producing B-cells and plasma cells supports this notion (5). Previous research in animal models furthermore demonstrated the critical role of B-cells in disease development as animals which lack functional B-cells or receive B-cell inhibitory signals are less prone to develop experimental arthritis (6).

Recent research established a clear role of cytokines, protein factors, and interaction of various cell populations including T-lymphocytes (T-cells), B-cells, and fibroblasts in the pathogenesis of the inflammatory lesion in the synovial membrane with consequent destruction of joint cartilage and bone. A recently discovered protein, B-lymphocyte stimulatory protein (BLys), is believed to contribute to B cell recruitment and activation in RA. Elevated concentrations of BLyS can be detected in the synovial fluid and serum of many patients with RA and in serum of patients with other inflammatory diseases (7).

An intimate relationship has also been established among dendritic cells, macrophages, B-cells, and T-cells within the RA synovium. In particular, dendritic cells and macrophages are clearly dependent on stimuli released by B lymphocytes, and this may explain why dendritic cell function may remain impaired beyond the recovery of B lymphocytes following B cell depletion (8;9) (31). Cellular interactions such as antigen processing and presentation occur upstream of the pro-inflammatory cytokine response involving tumor necrosis factor-α (TNF-α), interleukin-1, and

interleukin-6. In addition, production of IgM-RF and IgG-RF by plasma cells in the rheumatoid synovium may act as a self-perpetuating stimulus for local B-cell proliferation and differentiation (10).

Therefore, it appears increasingly evident that B-lymphocytes play a key pathogenetic role in the induction and maintenance of RA.

As B-lymphocytes are involved in various cellular interactions with other cells of the immune system in RA, B-cell depletion following ofatumumab treatment can be expected to affect disease activity within a few weeks after initiation of therapy. In addition, a prolonged beneficial effect can be anticipated with this treatment due to the upstream position of the B-cell system in relation to the classical pro-inflammatory cytokine pathways and to the slow recovery of this cell population as well. Remarkably, the clinical efficacy in RA achieved by depleting B-cells in the anti-TNF-α naïve RA patients occurs at a magnitude that is at least as high as obtained with TNF-α blockade (11) or with downregulation of T-cell function with CTL4A-Ig (abatacept) (12).

3.1.2 Current Treatment of Rheumatoid Arthritis

Medications traditionally used in the treatment of RA belong to three categories: Non-steroidal anti-inflammatory drugs (NSAIDs), corticosteroids, and disease-modifying anti-rheumatic drugs (DMARDs). These are defined as medications that retard the radiographic progression of disease (joint destruction), Nowadays, DMARDs encompass both synthetic agents (small molecules) and the more potent biological agents.

Initiation of therapy with DMARDs within three months after the diagnosis of RA is crucial, because delayed therapy results in substantially more radiographic joint damage (13). Methotrexate is the synthetic DMARD which demonstrated in clinical trials to induce a long-term response and is therefore most often selected for initial therapy. Methotrexate has thus become the current standard of care in RA (14). Concomitant administration of folic acid significantly decreases methotrexate-induced toxic effects (15).

Methotrexate however is effective (defined as obtaining an ACR20 (ACR=American College of Rheumatology) response) in about 50% of RA patients, and the effect tends to taper off four to six years after initial treatment by yet unknown mechanisms (16). Today, most patients not responding to methotrexate therapy will receive either therapy with new biologic agents (see below) or enter trials with combination therapy with other DMARDs (32), although the benefits of this combination therapy needs further evaluation (17).

Three biological products that inhibit the actions of TNF-α (infliximab, etanercept, and adalimumab) have been marketed for the treatment of RA in recent years. In clinical trials these agents induce ACR20 responses in about two thirds of patients and this efficacy was confirmed in several post-marketing studies.

Rituximab, a B-cell depleting chimeric anti-CD20 monoclonal antibody, was recently approved for the treatment of RA patients with an inadequate response to TNF-α blocking agents, as a statistically significant ACR20 response was achieved with this novel treatment compared to controls in clinical trials. Also in trials on anti-TNF-α naïve RA patients who failed methotrexate therapy, 2/3 of patients obtained an ACR20 response (18) lasting in some cases up to 2 years (19).

Recent data in RA patients relapsing TNF- α blocking treatments indicate that rituximab may be superior to alternative TNF- α blocking treatments now available (20). However, rituximab is immunogenic and human anti-chimeric antibodies (HACA) have been observed in up to 25% of patients (21) thus a fully human anti-CD20 antibody is warranted.

Abatacept, a novel biologic treatment for RA based on a CTLA4-IgG construct (which acts by inhibiting co-stimulation, thus activation, of T-lymphocytes), was recently approved for treating RA patients with an inadequate response to MTX (12) and to TNF-α blocking agents (22). Antibodies binding to the entire abatacept molecule or to the CTLA-4 portion of abatacept have been seen in 1.7% of patients (see FDA prescribing information, March 2007[*]). Moreover, abatacept treatment was also evaluated in combination with other DMARDs in a recent RA trial and when used in combination with TNF-α blocking agents high infection rates of 22.3% were observed (23).

Thus, due to limitations of clinical efficacy and adverse reactions profile of synthetic and biologic DMARDs, alternative new therapies for RA are still needed.

3.2 Investigational Medicinal Product

3.2.1 Ofatumumab

Ofatumumab is a fully human IgG1κ lytic monoclonal antibody (mAb) that specifically binds to the human CD20 antigen whose expression is restricted to B lymphocytes from the pre-B to the plasmacytoid immunoblast stage only. The antibody is generated via transgenic mouse and hybridoma technology and produced in a recombinant murine cell line (NS0) using standard mammalian cell cultivation and purification techniques.

Ofatumumab recognizes an epitope (AA 143-164) on the human CD20 molecule which is different from the epitope recognized by the other anti-CD20 B-cell depleting agent rituximab (AA 165-172), and this property likely accounts for the higher efficiency of B-cell killing observed with ofatumumab in both *in vitro* and *in vivo* pre-clinical studies comparing these two agents.

Ofatumumab is currently being developed for the treatment of relapsed or refractory Non-Hodgkin B-cell follicular lymphoma (FL), for the treatment of relapsed or refractory chronic B-lymphocytic leukemia (CLL), and for the treatment of active rheumatoid arthritis (RA).

[*] http://www.fda.gov/cder/foi/label/2007/125118s0016lbl.pdf

3.2.2 Pre-clinical Pharmacology

To characterize its features, ofatumumab has been tested in a series of *in vitro* and *in vivo* experiments, and in some instances compared to the reference antibody rituximab. A summary of data from the pharmacology and toxicology investigations, including important comparisons to rituximab, are provided below.

Ofatumumab represents a novel anti-CD20 mAb that recognizes a CD20 epitope localized in the second extracellular loop distinct from the site recognized by rituximab. On human tissue sections ofatumumab demonstrated tissue reactivity that is consistent with its target antigen specificity.

Following the binding of ofatumumab to tumor B-cells clustering of CD20 into lipid rafts, as seen with rituximab, was observed. Strong differences in antibody cell-surface off-rates were detected between ofatumumab and rituximab. Ofatumumab dissociated much more slowly from surface CD20 than rituximab, corresponding to a half life of 180 minutes for ofatumumab and 90 minutes for rituximab. In this respect, both efficient clustering into rafts and a lower off-rate value for ofatumumab are important characteristics for an effective activation of complement and may explain the superiority of ofatumumab over rituximab in Complement Dependent Cytotoxicity (CDC) of B-cells.

Ofatumumab was able to induce complement-mediated lysis of freshly isolated human B-CLL tumor cells, and rituximab-resistant cells lines expressing low levels of CD20 and high levels of CD55/CD59, which is known to diminish the lytic effect of complement. Ofatumumab- and rituximab-mediated Antibody Dependent Cell-mediated Cytotoxicity (ADCC) were equivalent. Ofatumumab, as opposed to rituximab, did not induce cell death of tumor B-cell lines by apoptosis. Though ofatumumab mechanism of action is similar to that of rituximab, differences in the effector activity potentials exist between both mAbs.

In an *in vivo* study employing a SCID mouse tumor model, ofatumumab-treated animals showed a dose-dependent prolongation of survival that was superior to rituximab. A pilot pharmacokinetic study in cynomolgus monkeys demonstrated that depletion of CD20 expressing B lymphocytes occurred rapidly after antibody administration. Depletion of B cells with ofatumumab lasted four times longer than depletion observed after administration of a similar dose of rituximab. There were no adverse effects considered to be related to the ofatumumab treatment. In addition, a second pilot study in cynomolgus monkeys showed that a dose up to 150 mg/kg of ofatumumab did not induce any adverse effects and therefore, was considered a safe dose in these animals.

For further information see the Investigator's Brochure.

3.2.3 Pre-clinical Safety

In a multiple dose toxicology study in cynomolgus monkeys, the toxicity of ofatumumab was investigated following four weekly doses by intravenous administration of 0, 20 or 100 mg/kg of ofatumumab. In the first set of animals, sacrificed two weeks after administration of the last dose, no changes in laboratory parameters, and no abnormal necropsy or histology findings (except for an expected germinal center atrophy after antibody treatment) were seen. Accordingly, analysis of $CD20^+$ cells in peripheral blood and lymph node biopsies of all animals showed a rapid depletion of these cells after administration of ofatumumab. In the second set of animals sacrificed five months after administration of last dose, the B-cell counts in the peripheral blood in four out of six animals receiving 20 mg/kg of ofatumumab recovered to baseline levels at Days 133 to 149. Percentages of $CD20^+$ cells in the lymph nodes of all treated animals reached baseline levels at Days 112 to 182. In animals receiving 100 mg/kg of ofatumumab, four out of six animals showed full B-cell count recovery in the peripheral blood and lymph nodes at Days 112 to 182.

Subsequently, a multiple dose chronic toxicology study entitled “Multiple Dose Intravenous Toxicity Study in Cynomolgus Monkeys followed by a 6 Month Recovery Period” was initiated in 2005. The objectives of this study were to assess the toxicity of ofatumumab in cynomolgus monkeys following 13 intravenous administrations over a 7 month period (*i.e.* Days 1, 8, 15, 22, 29, 36, 43, 50, 78, 106, 134, 162 and 190) and then, during a 6 month recovery period, to monitor B-cell recovery in the blood and lymph nodes of designated animals. For this purpose, 21 male and 21 female cynomolgus monkeys were assigned to 3 dose groups in which ofatumumab was administered at dose levels of 0, 20 and 100 mg/kg/dose (Groups 1, 2 and 3, respectively). Two days after completion of the last dose administration, the Main study animals were sacrificed and sent for necropsy, and the remaining Recovery study animals were retained for an approximate 6 month recovery period. The chronic treatment of cynomolgus monkeys receiving 13 intravenous administrations of ofatumumab over a period of 7 months led to a depletion of B cells, which recovered to baseline levels in the majority of animals upon completion of a 6 month recovery period. No influence on cell numbers or immune system physiology, other than the target cell population, could be detected. In total 5 monkeys died or were humanely sacrificed due to deteriorating health during the course of the study. These deaths occurred in both treatment groups. Post-mortem investigation confirmed that the deaths or deterioration in health were attributable to infections and were not attributable to direct toxicity of the test item. The reasons for animal withdrawals can be characterized into those which had a probable *C. jejuni* infection and those which showed signs of hemolytic anemia. A review of hemolytic events indicated that the majority of treated animals were experiencing a slowly developing hemolytic anemia during both the dosing and the recovery periods, which were associated with a progressive, dose related increase in lactate dehydrogenase which was reversible. Direct Coomb’s test results suggested that non-human primates probably developed a humoral response to ofatumumab which subsequently induced the formation of immune complexes capable of binding to the red cell surface. Preliminary results from the ongoing clinical studies indicate that ofatumumab does not induce hemolytic anemia in humans.

For further information see the Investigator's Brochure.

3.2.4 Previous Clinical Experience

Ofatumumab has been administered to patients in several phase I/II & III clinical trials, in B-cell Chronic Lymphocytic Leukemia (B-CLL), non-Hodgkin Follicular Lymphoma (FL), and Rheumatoid Arthritis (RA). Preliminary efficacy and safety data are available from one B-CLL and one FL trial. The remaining trials are still ongoing, and only limited safety data are available. Interim efficacy and safety data (24 weeks) are available for the rheumatoid arthritis study.

For further information see the Investigator's Brochure.

Follicular Lymphoma

A phase I/II trial investigating patients with relapsed or refractory FL grade I-II (Hx-CD20-001) has completed the treatment phase. It is an open-label, multi-center, and dose escalating trial testing doses in the ranges of 300 - 1000 mg (FL) administered as intravenous infusions once weekly for four weeks.

A phase III trial in patients with FL has been initiated (Hx-CD20-405). It is a double-blind, randomized, two-dose-arm trial testing doses in the range 300 - 1000 mg, administered as intravenous infusions once weekly for eight weeks.

A phase II open-label, randomized, parallel group trial in 56 patients with previously untreated Follicular Lymphoma (Hx-CD20-409) has been initiated. Patients will receive an infusion of ofatumumab 3000 mg i.v. on day 0 in combination with CHOP on days 1-5. Thereafter, patients will receive either ofatumumab 500 mg or 1000 mg (1:1) at weeks 3, 6, 9, 12, and 15.

B-cell Chronic Lymphocytic Leukemia

For the phase I/II trials B-CLL in patients with relapsed or refractory B-CLL (Hx-CD20-402), recruitment has been completed. It is an open-label, multi-center, and dose escalating trial testing doses in the ranges 100 – 2000 mg administered as intravenous infusions once weekly for four weeks.

A phase III trial in patients with B-CLL (Hx-CD20-406) has been initiated. It is an open-label, multi-center trial testing doses in the range 300 – 2000 mg administered as intravenous infusions in a combination of weekly and monthly infusions.

A phase II two-arm trial (Hx-CD20-407) in which ofatumumab is investigated in previously untreated patients with B-CLL has been initiated. In this study ofatumumab is administered as 6 monthly infusions (1x300mg + 5x500) / (1x300mg + 5x1000) in combination with fludarabine and

cyclophosphamide on days 1-3.

Rheumatoid Arthritis

A phase I/II trial investigating ofatumumab in patients with active RA (Hx-CD20-403) has been conducted. The first part (Part A) of the trial, which is the initial placebo-controlled dose-escalation part of the study, is completed. The second part (Part B), which has a placebo-controlled parallel group design with randomization into one of four treatment arms, is of 48 weeks duration, and the primary endpoint is ACR20 response at 24 weeks. This study is completed up to 24 weeks to date, with follow-up 48 weeks ongoing. In Part A, 39 patients distributed across three cohorts were randomized (4:1 in each cohort) to receive two infusions of ofatumumab 300 mg (n=12), 700 mg (n=10), 1000 mg (n=10) or placebo (n=7) given two weeks apart. In Part B, 225 patients were randomized to receive two infusions of ofatumumab 300 mg (n=58), 700 mg (n=57), 1000 mg (n=54) or placebo (n=56) two weeks apart. In total, 201 patients with rheumatoid arthritis received ofatumumab.

Results from Part A and interim results (to 24 weeks) from Part B are described in the Investigator's Brochure.

3.2.5 Summary of known and potential risks to human subjects

The following reactions have been observed in patients exposed to ofatumumab and should be regarded as expected infusion-related adverse reactions associated with administration of this antibody: pruritus, dyspnoea, throat irritation, rigors/chills, headache, flushing, nausea, hypotension, urticaria, fatigue, fever, and rash. However, experience to date shows that these infusion-related reactions can be prevented or reduced in frequency and severity when patients are administered pre-medication prior to ofatumumab infusion as described in Section 3.3.4. Profound and prolonged depletion of peripheral $CD20^+$ B lymphocytes has been observed during treatment with ofatumumab, as expected with the administration of an anti-CD20 monoclonal antibody. To date, an increased risk of serious infectious complications associated with ofatumumab has not been observed.

Further information can be found in the Investigator's Brochure.

3.3 Study rationale

Data from the ongoing RA phase I/II trial, Hx-CD20-403 (i.e. final data from Part A and interim data from Part B) have shown ofatumumab to be generally well tolerated in patients with rheumatoid arthritis , with the majority of adverse events being reported on the day of ofatumumab infusion. In Part B significantly more patients achieved the primary endpoint, ACR20 response at week 24, in the 300 mg (40%), 700mg (49%) and 1000mg (44%) groups compared to placebo (11%). A similar benefit over placebo was demonstrated with other efficacy measures investigated in the trials including the Disease Activity Score with 28 joints (DAS28) and the EULAR response.

Overall, these data support investigation of the efficacy and safety of ofatumumab in phase III clinical trials for rheumatoid arthritis.

A recent study in RA patients with a previous inadequate response to TNF-blocking agents who received at least three courses of rituximab demonstrated in a study of 96 patients a marked reduction in DAS 28 from baseline with 69%, 76% and 77% of patients achieving an ACR20 relative to the original baseline during the first, second and third courses of therapy, respectively (37). Due to the chronic nature of rheumatoid arthritis, this therefore supports the objective of administering ofatumumab as repeated treatment courses in this study.

3.3.1 Study population

Patients eligible for this study are adults with active RA of at least six months' duration who have had an inadequate response to previous or current TNF-α antagonist treatment.

Previous exposure to biologic cell-depleting RA therapies will not be allowed for this trial.

3.3.2 Dose

Based on review of the efficacy and safety interim data from the phase I/II trial, Hx-CD20-403, the dose of 700mg ofatumumab (as 2 infusions administered two weeks apart) for investigation in the phase III clinical programme in patients with rheumatoid arthritis has been selected. This is supported by the following data from study Hx-CD20-403:

- In the intention-to-treat study population, comprising 224 patients, ACR20 was achieved by 44% of all patients receiving ofatumumab, ACR50 achieved by 22% and ACR70 achieved by 6% of ofatumumab patients compared to 11%, 4% and 0% in the placebo group. Evaluated by dose group, an ACR20 response was obtained by 40% ($p<0.001$), 49% ($p<0.001$) and 44% ($p<0.001$) of patients receiving 300mg, 700mg and 1000mg of ofatumumab respectively compared to 11% in the placebo group.

- In the subgroup of patients receiving concomitant stable doses of methotrexate, comprising 174 patients, results across the three dose levels of ofatumumab studied showed that an ACR20 response was obtained by 41%, 55% and 50% of patients in the 300 mg, 700 mg and 1000 mg dose groups, respectively compared to 14% in the placebo group. Furthermore, for the EULAR and DAS28 efficacy outcomes all ofatumumab dose groups were superior to placebo with a numerically greater benefit demonstrated in the 700mg and 1000mg compared to the 300mg group in the subgroup of patients receiving concomitant methotrexate.

- Ofatumumab was generally well tolerated with a total of 81%, 84%, 83% and 57% of subjects in the 300mg, 700mg, 1000mg and placebo groups reporting at least one adverse event respectively. Approximately half of the adverse events noted in the ofatumumab

groups occurred on infusion days (51%) with the most frequently reported being mild or moderate (CTC grade 1-2 events), including throat irritation, dyspnoea and rash NOS. The proportion of patients who reported an AE on the day of 1st infusion was 28/58 (48%), 39/57 (68%) and 40/54 (74%) in the 300mg, 700mg and 1000mg groups respectively. The following AEs of grade 3 or greater (severe) were observed on the day of infusion as follows: first day: throat tightness (300mg); bronchospasm NOS (2 events; both 700mg); fatigue (700mg); infusion related reaction (4 events; 1 in 700mg; 3 in 1000mg); hypersensitivity (1000mg); rash NOS (1000mg) and on the second infusion day: IDDM (300mg); blood potassium increased (1000mg). Serious Adverse Events were reported in 3 (5%) subjects in the placebo group, 4 (7%), 5 (9%), 8 (15%) in the 300mg, 700mg and 1000mg groups respectively. Although there was a higher frequency of infusion-related adverse events reported in the 700mg and 1000mg ofatumumab groups compared to the 300mg group, following the modifications to the premedication regimen the majority were mild to moderate in severity and did not preclude treatment with ofatumumab.

- There is a demonstrated correlation between exposure (AUC, Cmax and Cmin) and ACR20 response such that responders had a significantly higher AUC, C_{min} and C_{max} than non-responders. This therefore indicates that the 700mg ofatumumab dose, by providing greater exposure may have the potential to provide improved efficacy compared to a 300mg dose.

Thus in summary, these data show 700 mg ofatumumab to be an effective and generally well tolerated dose. Taken together these data support selection of 700 mg ofatumumab (as 2 infusions 2 weeks apart) for investigation in phase III rheumatoid arthritis trials.

3.3.3 Dose Schedule & Re-treatment

Dose Schedule
Two i.v. infusions of ofatumumab separated by 14 days has been chosen based on the following considerations:

Few studies have addressed the dosing schedule of rituximab. In the early paper of Leandro et al. (24), a total of 5 patients, who received only one infusion did not achieve ACR responses whereas in the remaining patients receiving at least 2 infusions (a total of 17 patients), ACR responses were obtained. All subsequent studies with rituximab have used 2 infusions 2 weeks apart. A 2-compartment model based on such studies, used to analyze the population pharmacokinetic profiles, fits reasonably well with the PK data (25). Both the half life and C_{max} increase considerably comparing the first and second dose. Likewise, the clearance is markedly reduced comparing the first and second dose. Similar data have been obtained with ofatumumab in rheumatoid arthritis (see Investigator’s Brochure).Therefore, 2 doses are selected to ensure maximal depletion of B cells in lymphoid tissues and bone marrow from which B cells are recruited following B cell depletion (26;27).

Re-treatment

The clinical efficacy of the 2-infusions cycle of rituximab therapy in rheumatoid arthritis who respond to the first cycle ("responders") usually lasts on average between 6 and 15 months in patients achieving an ACR20 (26) response, and in most cases B cells have returned to baseline levels before loss of clinical efficacy (26) is observed. Current scientific evidence on the pathophysiology of RA shows that a prolonged and tight control of inflammation is needed to secure clinical benefit and minimize joint structural damage (28), and this is why new biologic therapies such as TNF-α blocking agents and abatacept are indeed administered on a continuous basis.

After B cell depletion, B cells are still present in synovial tissue in patients responding to rituximab at 8 weeks whereas B cells are absent from peripheral blood (38). Furthermore, the reduction in numbers of inflammatory cells other than B cells in the synovial tissue supports the view that B cells facilitate the local cellular infiltration, possibly by depletion of precursors of short-lived plasma cells associated with autoantibody production (38).

Findings from an open-label study of patients with rheumatoid arthritis who were allowed re-treatment with rituximab if tender and swollen joint counts had improved ≥ 20%, and not necessarily meeting an ACR20 response, indicated that re-treatment may yield further depletion of synovial B-cells. In this study, the proportion of patients achieving ACR20 responses with the second course of treatment increased compared to the first course; thus the ACR20 response comparing the first and second treatment courses in the same 99 patients increased from 59% to 73%, the ACR50 from 27% to 37% and the ACR70 from 9% to 19%, with all of these differences reaching statistical significance (36). Re-treatment with an anti-CD20 depleting antibody may therefore achieve a clinical response in patients who did not meet an ACR20 response after the first treatment.

Recent studies demonstrated long-term efficacy (29) without significant changes in the safety profile following re-treatment with rituximab when compared to single cycle therapy (29), (33), (34). A rational approach to minimize the inflammatory and joint-destructive process in RA patients who respond to B-cell depletion is to provide retreatment with B-cell depleting therapy, which is not expected to alter the safety profile according to current knowledge.

Cambridge and coworkers (26) demonstrated a minimum relapse time of 6 months in a population of active RA patients treated with rituximab. Furthermore, it is known from studies with rituximab that saturation of CD20 receptors occurs with concentrations around 10 µg/ml (39). The PK profile from the Phase I/II trial with ofatumumab in RA (Hx-CD20-403) indicated a terminal T½ of 15.6 days after the 2nd infusion with Cmax of 373.6 µg/ml. Assuming similar CD20 receptor occupancy for ofatumumab, 10 µg/ml will be reached after 5-6 T½'s after the 2nd dose. At 6 months (corresponding to 11-12 T½'s), the expected concentration of ofatumumab is low, around 0.2 µg/ml. Thus, it is predicted that re-dosing after 6 months will not cause accumulation of ofatumumab.

The phase I/II study of ofatumumab in RA patients (Hx-CD20-403) demonstrated minimal reduction of IgG and IgM following one treatment course. Therefore, assessments of IgG and IgM status to decide if patients can progress to the first re-treatment in the open-label period of this trial have been omitted. Patients who are IgM deficient and/or IgA deficient do not generally have an increased risk of infections as opposed to patients with low IgG (hypogammaglobulinemia or agammaglobulinemia). Therefore, the decision for re-treatment is based on IgG levels rather than IgM and IgA levels. Consequently, to assure the patients' safety, a minimum threshold level for IgG equivalent to the lower limit of normal has been set.

With this knowledge an individualized re-treatment schedule with 2x700 mg of ofatumumab has been selected.

3.3.4 Pre-medication

In Part A of the ofatumumab phase I/II trial (Hx-CD20-403), patients were initially pre-medicated only with paracetamol and anti-histamines prior to the 2 infusions of ofatumumab 2 weeks apart. With this treatment regimen occurrence of grade >3 infusion-related AEs were observed, thus, pretreatment with corticosteroids was subsequently implemented after issuing amendments to the protocol. In these amendments the dose of i.v. prednisolone (or similar) was increased up to 100 mg administered 1-2 hours prior to the ofatumumab infusions. An additional dose of prednisolone (100 mg orally the day before the first infusion and 30 mg the day before the second infusion) was also implemented. By these amendments, the incidences of grade 3 infusion-related AEs according to National Cancer Institute Common Terminology Criteria Adverse Events version 3.0 (NCI CTCAE) on the day of the first infusion was reduced from 14% to 6%; however, it was noted that pre-treatment with steroids the day before the infusions had little impact while a major reduction in the incidence of infusion-related grade 3 AEs occurred when the infusion volume was increased from 500 mL to1000 mL, allowing for infusion of less drug substance at the initiation of the infusion. Following the increase in infusion volume, the incidence of grade 3 infusion-related AEs among 169 patients was reduced from 6% to 3.5%. Hence, the major reduction of infusion-related grade 3 AEs was obtained by increasing the volume of the infusion to 1000 mL. Thus, treatment with steroids the day before the infusions in this study is not mandatory.

4 Objectives

4.1 Primary Objective

- To demonstrate efficacy of ofatumumab in reducing clinical signs and symptoms in adult RA patients after a single course of ofatumumab

4.2 Secondary Objectives

- To evaluate long-term efficacy of repeated courses of ofatumumab
- To evaluate the effect on established and novel biomarkers of clinical response after single and repeated courses of ofatumumab
- To evaluate ofatumumab with respect to impact on patient reported outcomes after single and repeated courses of ofatumumab
- To evaluate the risk of host immune response against ofatumumab after single and repeated courses of ofatumumab
- To evaluate the safety of ofatumumab after single and repeated courses of ofatumumab

4.3 Primary Endpoint

- ACR20 at 24 weeks

4.4 Secondary Endpoints

Key Secondary Endpoints:

- ACR20, ACR50, ACR70 and ACRn
- DAS28
- EULAR response

Other Secondary Endpoints:

- Biomarkers of disease activity, immune status, and whole blood transcriptional profiles
- Short-Form health survey (SF-36) and Functional Assessment of Chronic Illness Therapy in Fatigue (FACIT-F)
- Human anti-human antibodies
- Adverse events, clinical laboratory parameters, vital signs, ECG
- Tender joint count (TJC)
- Swollen joint count (SJC)
- Patient VAS of pain
- Patient VAS of global disease
- Physician VAS of global disease
- Health Assessment Questionnaire (HAQ-DI)
- C-reactive protein (CRP) and Erythrocyte sedimentation rate (ESR)

5 Patient Selection and Withdrawal

To be eligible the patient has to meet all inclusion criteria and must not violate any of the exclusion criteria.

5.1 Inclusion Criteria

1) Age ≥ 18 years

2) A diagnosis of rheumatoid arthritis according to the American College of Rheumatology (ACR1987 classification) of at least six months' duration from diagnosis at screening

3) Active disease at the time of screening as defined by:
- ≥ 8 swollen joints (of 66 joints assessed)
and
- ≥ 8 tender joints (of 68 joints assessed)
and
- C-Reactive Protein (CRP) ≥ 1.0 mg/dL or Erythrocyte Sedimentation Rate (ESR) ≥ 22 mm/hour
and
- DAS28≥3.2 (based on ESR)
Note: The swollen and tender joints must be reassessed at baseline (Visit 2) to ensure these eligibility criteria are fulfilled prior to randomization. Where possible joint count reassessment must be performed at the baseline visit (Visit 2); if this is not possible it can be performed ≤ 3 days prior to Visit 2.

4) RA functional class I, II or III*

5) Inadequate response to previous or current TNF-α antagonist treatment after infliximab therapy (≥3 mg/kg; at least 4 infusions), or adalimumab therapy (40 mg at a minimum of every other week for ≥3 months), or etanercept therapy (25 mg twice weekly or 50 mg once a week for ≥3 months) defined as:

- Inadequate efficacy according to the investigator's judgment

  and/or

- Intolerance defined as one or more side effects following at least one administration of one of the TNF-α antagonists at the dose listed above that reasonably results in the discontinuation of treatment with the TNF-α antagonist treatment

* For definition of RA functional class please refer to Appendix 1

6) Treatment with methotrexate (MTX), 7.5-25 mg/week, for at least 12 weeks and at a stable dose for at least 4 weeks prior to Visit 2. Doses of MTX as low as 7.5 mg per week are permitted for patients who can not tolerate higher doses

7) Following receipt of verbal and written information about the study, the patient must provide signed informed consent before any study related activity including wash-out of other drug products is carried out

   France: In France, a patient will be eligible for inclusion in this study only if either affiliated to or a beneficiary of a social security category.

5.2 Exclusion Criteria

1) Patients with a history of a rheumatic autoimmune disease other than RA (except secondary Sjögren's syndrome), or with significant systemic involvement secondary to RA (vasculitis, pulmonary fibrosis or Felty's syndrome)

2) Previous exposure to biologic anti-rheumatic therapies, (with the exception of those specified in Exclusion Criteria 3 below) including investigational compounds (e.g. anti-CD11a, anti-CD19, anti-CD20, anti-CD22, anti-BLyS/BAFF, anti-CD3, anti-CD4, anti-CD5, CAMPATH, anti-IL-6 receptor)

3) Exposure to etanercept ≤ 4 weeks, infliximab or adalimumab ≤ 8 weeks, or abatacept ≤ 12 weeks prior to Visit 2

4) Received any of the following treatments within 4 weeks prior to Visit 2:

   - Anti-cancer therapy (e.g. alkylating agents, anti-metabolites, purine analogues, monoclonal antibodies)

   - Glucocorticoid unless given in doses equivalent to ≤ 10 mg of prednisolone /day

   - Intra-articular, i.m. or i.v. corticosteorids

   - Live/attenuated vaccinations

   - Cyclosporine

   - Azathioprine

   - Penicillamine

- Bucillamine
- chloroquine
- hydroxychloroquine
- sulfasalazine

5) Exposure to leflunomide within 12 weeks prior to Visit 2 unless the patient has completed peroral cholestyramine treatment for washout according to the manufacturer's instructions and locally accepted clinical practice

6) Exposure to gold therapy ≤ 12 weeks prior to Visit 2

7) Exposure to i.v. immunogammaglobulins ≤ 24 weeks prior to Visit 2

8) Past or current malignant melanoma

9) Past or current malignancy, except for:

   - Cervical carcinoma Stage 1B or less
   - Non-invasive basal cell and squamous cell skin carcinoma
   - Other cancer with complete response duration of > 5 years

10) Chronic or ongoing active infectious disease requiring systemic treatment such as, but not limited to, chronic renal infection, chronic chest infection with bronchiectasis, tuberculosis (TB) and active hepatitis B and C:

    Note: i) Subjects with a screening chest X-ray suggestive of TB without documentation of adequate TB treatment (see Section 8.1.11) will be excluded; ii) Screening for latent TB infection using intradermal injection of tuberculin (e.g. the Mantoux test or equivalent) should be conducted in accordance with local guidelines (see Section 8.1.12). Subjects with a positive skin tuberculin test should be excluded if the investigator judges the patient to be at risk of latent TB infection.

11) Clinically significant cardiac disease including unstable angina, acute myocardial infarction within six months from screening, congestive heart failure, known QT abnormalities, and arrhythmia requiring therapy, with the exception of extra systoles or minor conduction abnormalities

12) Significant concurrent, uncontrolled medical condition including, but not limited to, renal, hepatic, hematological, gastrointestinal, endocrine, pulmonary, neurological, cerebral psychiatric disease, or evidence of demyelinating disease

13) History of significant cerebrovascular disease

14) Known HIV positive

15) Screening laboratory values (according to central laboratory):
- Hemoglobin < 5.6 mmol/L (9.0 g/dL)
- Neutrophils < 2 x 10^9/ L
- Platelets < 100 x 10^9/ L
- Serum IgG < lower limit of normal
- S-ALAT > 3.0 times the upper limit of normal
- S-AST > 1.5 times the upper limit of normal
- S-ALP > 2 times the upper limit of normal
- S-creatinine > 133 µmol/L (1.5 mg/dL)

16) Serologic evidence of Hepatitis B (HB) infection based on the results of testing for HBsAg, anti-HBc and anti-HBs antibodies as follows:

- Patients positive for HBsAg are excluded
- Patients negative for HBsAg but positive for both anti-HBc and anti-HBs antibodies are eligible to participate
- Patients negative for HBsAg and anti-HBc antibody but positive for anti-HBs antibody are eligible to participate
- Patients negative for HBsAg and anti-HBs antibody but positive for anti-HBc antibody will require clarification of their status by testing for HBV DNA which if positive will exclude the patient from participation.

Patients with documented vaccination against Hepatitis B (primary and secondary immunization and booster) will be considered negative.

17) Positive test for Hepatitis C antibody confirmed on the same sample with a Hepatitis C RIBA® immunoblot assay. Subjects who are positive for Hepatitis C antibody and negative when the Hepatitis C RIBA® immunoblot assay is performed on the same sample will be eligible to participate. Subjects who are positive for Hepatitis C antibody and have a positive or indeterminate result when the Hepatitis C RIBA® immunoblot assay is performed on the same sample will not be eligible to participate.

18) Positive plasma / white cell JC Virus (JCV) PCR (either compartment)

19) Known hypersensitivity to components of the investigational medicinal product

20) Patients who have received treatment with any non-marketed drug substance or experimental therapy within 4 weeks prior to screening

21) Current participation in any other interventional clinical study

22) Patients known or suspected of not being able to comply with a study protocol (e.g. due to alcoholism, drug dependency or psychological disorder)

23) Breast feeding women or women with a positive pregnancy test at screening

24) Women of childbearing potential not willing to use adequate contraception during study and one year after last dose of ofatumumab. Adequate contraception is defined as abstinence, oral hormonal birth control, implants of levonorgestrel, hormonal birth control injections, estrogenic vaginal ring, percutaneous contraceptive patches, intrauterine device, and male partner sterilization if male partner is sole partner for that patient.
For patients in the USA the use of a double barrier method is also considered adequate (condom or occlusive cap plus spermicidal agent).

25) Male patient, with a female partner of child bearing potential, not willing to use condoms or abstinence for the full duration of the study. Contraceptive measures should only be discontinued after completion of or withdrawal from the study following the advice of the physician prescribing methotrexate and in accordance with the local methotrexate label.

5.3 Withdrawal Criteria

Patients will not be substituted if withdrawn from the trial.

5.3.1 Withdrawal from treatment

A patient should be withdrawn from ofatumumab/placebo if at any time:

- It is the wish of the patient (or their legally acceptable representative) for any reason
- The investigator judges it necessary due to medical reasons
- The patient becomes pregnant
- The patient receives prohibited therapy as listed in Section 7.4.2
- An adjustment in concomitant MTX is initiated except for decreases in dose due to toxicity
- If the patient at any time during the trial (including the Follow-up Period) enters another interventional clinical study
- The patient has an abnormal ECG finding judged by the investigator to be clinically relevant
- The patient has an abnormal liver chemistry as defined in Section 9.5.2

- The patient becomes positive for hepatitis B infection*
- The patient develops symptoms of PML
- If, after the second treatment course and subsequent re-treatments with ofatumumab in the Open-label Period, if the patient has not at least once achieved a
  - moderate EULAR response compared to Visit 2 values (for definition of a EULAR response please refer to Section 10.3.4) **and/or**
  - 20% improvement in both swollen and tender joint counts (compared to baseline value from Double-blind Period, i.e. Visit 2) within 24 weeks since the last ofatumumab re-treatment course
- If circulating IgG levels are below lower limit of normal according to central laboratory range at any time during the Open-label Period from the first scheduled visit after the patient has received the second treatment course

Double-blind Period

If the patient is withdrawn from ofatumumab/placebo during the Double-blind Period the patient should continue protocol assessments until completion of the Double-blind Period at 24 weeks. Thereafter, the patient should proceed to the Follow-up Period as outlined in Section 6.5. Due to safety reasons the patient should only be offered the non-biological DMARDs which are described in Section 7.4.1.

Open-label Period

If the patient is withdrawn from ofatumumab during the open-label period the patient should proceed to the Follow-up Period according to Section 6.5.

Patients should, whenever possible, irrespective of the reason for withdrawal, be examined as soon as possible. Relevant samples should be obtained and all relevant assessments should be completed according to the week 24 / early withdrawal visit. The “Study Conclusion section” of the electronic case report form (eCRF) should be completed.

If a patient does not return for a scheduled visit, every effort should be made to contact the patient. In all circumstances, every effort should be made to document patient outcome. All study drug-related adverse events should be followed until resolved or until the Investigator assesses them as chronic or stable.

The Investigator, in consultation with the patient, will decide the future course of treatment.

* See exclusion criterion 17

5.3.2 Withdrawal from trial

A patient should be withdrawn from the trial if at any time:

- It is the wish of the patient (or their legally acceptable representative) for any reason
- The investigator judges it necessary due to medical reasons
- The patient initiates treatment with other B-cell suppressive treatment (e.g. other anti-CD20 antibodies, cyclophosphamide, azathioprine etc.)

5.3.3 Withdrawal from Safety Follow-up

A patient should be withdrawn from the Follow-up if at any time:

- It is the wish of the patient (or their legally acceptable representative) for any reason
- The investigator judges it necessary due to medical reasons
- The patient initiates treatment with other B-cell suppressive treatment (e.g. other anti-CD20 antibodies, cyclophosphamide, azathioprine etc.)
- The patient enters another interventional clinical trial and/ or receives treatment with any non-marketed drug substance or experimental therapy.

6 Trial Design

6.1 Overall Design

This is a phase III, double-blind, randomized and parallel group trial with a duration of 24 weeks (Double-blind Period), followed by a 120 week open label extension (Open-label Period). A schematic of the study is provided in Figure 2.

Eligible patients will be randomized in a 1:1 ratio to receive ofatumumab 700mg x 2 or placebo x 2 in addition to their background methotrexate treatment. The randomized treatment will be administered as two infusions 14 days apart, one at day 0 and one at day 14 (one treatment course) after randomization. Randomization will be stratified by rheumatoid factor seropositivity / -negativity, and region. For patients who are not responding, non-biological DMARD rescue treatment as listed in 7 will be allowed from week 16 but the use of rescue treatment will preclude subsequent entry into the Open-label Period. Breakthrough pain management in the form of analgesics, NSAIDs and intra-articular corticosteroid injections will be allowed during the Double-blind and Open-label Periods. Use of intra-articular corticosteroid injections will be prohibited 4 weeks prior to the primary endpoint (i.e. Visit 9, week 24). One injection in one single joint per 6 month period will be permitted throughout the Double-blind and Open-label Periods. The joint receiving this i.a. injection will be scored as swollen and tender in joint count assessments during the following 12 week period.

All patients (i.e., those who have received ofatumumab or placebo) who complete the 24 week Double-blind Period without receiving rescue DMARD treatment will be eligible to proceed into the 120 week Open-label Period to receive repeat treatment courses with ofatumumab (700mg x 2 two weeks apart). In the Open-label Period ofatumumab treatment courses will be given at individualized time intervals only if a clinical response has been achieved following the previous treatment course, and followed by a subsequent worsening in disease activity as follows:

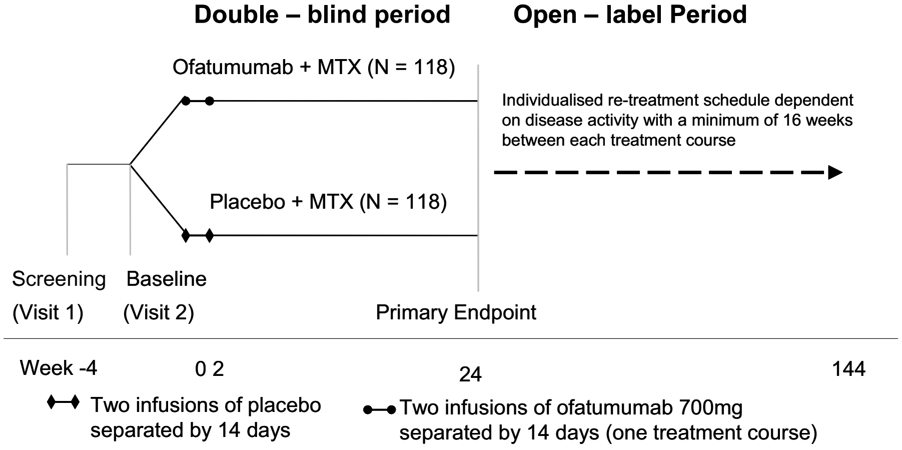

Figure 2: Trial Design

<u>First re-treatment course</u>:

If a patient has not achieved at least a moderate EULAR response **<u>and/or</u>** ≥20% improvement in both swollen <u>and</u> tender joint counts (compared to baseline values, i.e. Visit 2) at either week 16, 20, or 24 of the Double-blind Period the patient can be given the first re-treatment course at week 24 (i.e., dosing at weeks 24 and 26).

If a patient has achieved at least a moderate EULAR response or ≥20% improvement in swollen <u>and</u> tender joint counts (compared to baseline values, i.e., Visit 2) at either week 16, 20 or 24 of the Double-blind Period, the patient will receive the first re-treatment course when there is a worsening in disease activity defined as:

- DAS28 ≥3.2 **PLUS** a ≥0.6 increase in DAS28 compared to lowest DAS28 after the first treatment course at baseline (i.e. Visit 2) **<u>and/or</u>** a ≥20% increase in both tender <u>and</u> swollen joint counts compared to lowest counts after the first treatment course at baseline (i.e. Visit 2).

Note: A moderate EULAR response or a ≥20% improvement in both swollen and tender joint counts is only considered to be valid if it is recorded at least 6 weeks after date of last infusion to allow washout of any positive effects of corticosteroid treatment from the pre-infusion treatment. Similarly the response is only valid if recorded at least 4 weeks after any i.a injection of corticosteroid.

<u>Second and subsequent re-treatment courses:</u>

After the first re-treatment course, patients will be eligible to receive further ofatumumab re-treatment courses during the 120-week Open-label Period if she/he:

- Achieves an efficacy response to ofatumumab at least once within 24 weeks from the last ofatumumab re-treatment course, defined as at least a moderate EULAR response **<u>and/or</u>** ≥20% improvement in both swollen <u>and</u> tender joint counts (compared to baseline value from Double-blind Period, i.e., Visit 2).

  PLUS

Following this response, subsequently shows a worsening in disease activity at any time since the last ofatumumab re-treatment course defined as:

- DAS28 ≥ 3.2 **PLUS** a ≥0.6 increase in DAS28 compared to lowest DAS28 after the last re-treatment course **<u>and/or</u>** a ≥20% increase in both tender <u>and</u> swollen joint counts compared to lowest counts since the previous treatment course

Note, in the Open-label Period the interval between initiation of a new treatment course for patients should be at least 16 weeks (16 weeks is counted from infusion A from the preceding infusion course) irrespective of progression in disease activity. The last treatment course should be planned to occur no later than the scheduled visit at week 120 after baseline (Visit 2), i.e. Infusion A of the last treatment course must be given no later than at week 120.

Patients who do not obtain at least a moderate EULAR response **or** ≥20% improvement in both swollen and tender joint counts (compared to baseline values from Double-blind Period, i.e., Visit 2) within 24 weeks from the first day of infusion in the previous treatment course in the Open-label Period will be withdrawn from further ofatumumab treatment and proceed to the Follow-up Period.

In addition, any patient with a circulating IgG level <lower limit of normal (as assessed by the central laboratory) at any time after the second treatment course will be withdrawn from further ofatumumab treatment and enter the Follow-Up Period. In the Follow-up Period the patient will be followed until the number of B-cells, and circulating IgG have returned to normal (according to the central laboratory) or baseline (i.e. Visit 2) levels or for a maximum of 2 years from the last scheduled visit in the Double-Blind or Open-label Period, whichever occurs earlier.

Patients who have completed the Open-label Period or who do not qualify for re-treatment will be followed (i.e., in the Follow-up Period) until the number of B-cells and circulating IgG have returned to normal (according to the central laboratory) or baseline (i.e. Visit 2) levels or for a maximum of 2 years from the last scheduled visit in the Double-Blind or Open-label Period, whichever occurs earlier.

In the event the trial is prematurely terminated, all subjects in Double-blind or Open-label Period will enter the Follow-Up Period at the next scheduled study visit (see Section 6.5 for details on Follow-up activities).

6.2 Schedule of Events

Every effort should be made to adhere to all the protocol visit windows: in circumstances where this is not possible (for example patient on holiday or other unexpected circumstances), this should be documented in the patient notes with reasons. For more information about the visits please refer to the Flow Chart included in Section 2.

6.3 Screening (Visit 1)

When an investigator identifies a patient suitable for screening and the patient has provided informed consent, the screening visit (Visit 1) can be initiated. All screening examinations must be performed within 28 days prior to the baseline visit (Visit 2). The only exception is the chest X-ray which, if already obtained as part of routine treatment ≤12 weeks prior to the screening visit, is not

required. To accommodate for fluctuations in joint swelling and tenderness, CRP and ESR, these assessments may be repeated once during the 28 day period from the screening visit to the baseline visit (Visit 2), to meet requirements for inclusion criterion 3. No other assessment can be repeated to meet the eligibility criteria.

For patients requiring DMARD washout out prior to baseline (Visit 2), this can be initiated (dependent on the wash-out period specified in Section 5.2) ahead of the screening visit. However, patient informed consent must be obtained prior to commencing DMARD washout.

All patients subjected to screening will receive a Patient ID Number and should be listed on the Patient Screening and Allocation Log. The Patient Screening and Allocation Log will as a minimum contain, Date of Birth, Patient ID Number (if applicable) and reason for failing screening (if applicable). For patients failing screening, the reason for not entering the study should be provided in the screening and allocation log.

Each patient from the screening phase that is willing to participate and is found eligible according to the inclusion and exclusion criteria will enter the treatment phase.

6.4 Treatment

The investigator must confirm the patient's eligibility in the eCRF before the patient can be randomized and receive trial treatment.

The investigator must maintain an Identification List of all allocated patients at the site containing data to identify the patient and for traceability purposes. This list will contain full name, initials, date of birth, and other contact details. The identification list will not be disclosed to or collected by the sponsor but may be seen by the Clinical Research Associate (CRA).

6.4.1 Double-blind Period (Visit 2 to Visit 9)

The swollen and tender joint counts must be reassessed at baseline (Visit 2), or if this is not possible within $\leq$ 3 days of Visit 2, to ensure these eligibility criteria are fulfilled prior to randomization. If the number of swollen and tender joints are less than 8, the patient cannot be randomized into the study.

Following randomization into the study, each patient will receive 2 infusions, separated by 2 weeks, of either ofatumumab 700 mg or placebo. Treatment will be blinded to the treating staff as well as patients. Treatment allocation will only be known to dedicated pharmacy (or equivalent) staff.

If required for practical reasons, patients may have their Baseline Visit 2 procedures conducted over two consecutive days, Day 0 and Day 1, with the infusion of ofatumumab / placebo given on Day 1. Study Visit 3 should subsequently occur 14 days after the first infusion.

All patients will be followed for concomitant medication, adverse events, and other assessments

according to the Flow Chart shown in Section 2.

Efficacy assessments include evaluation of 66/68 joints for tenderness and swelling to be performed by an independent joint evaluator, VAS (pain), VAS (global disease) for the patient and treating physician, health assessment questionnaire (HAQ-DI), and laboratory assessments (CRP & ESR). Based on these assessments ACR, DAS28 (DAS28 is based on ESR) and the EULAR response will be calculated.

All patients will complete the SF-36 and FACIT-F according to the Flow Chart shown in Section 2.

6.4.2 Open-label Period (Visit 9 and forwards)

For the first re-treatment course:

If a patient has not achieved at least a moderate EULAR response **and/or** ≥20% improvement in both swollen and tender joint counts (compared to baseline values, i.e. Visit 2) at either week 16, 20 or 24 of the Double-blind Period the patient can be given the first re-treatment course at week 24 (i.e., dosing at weeks 24 and 26).

If a patient has achieved at least a moderate EULAR response or ≥20% improvement in swollen and tender joint counts (compared to baseline values, i.e., Visit 2) at either week 16, 20 or 24 of the Double-blind Period, the patient can enter directly into the Open-label Period and will receive the first re-treatment course when there is an worsening in disease activity defined as:

- DAS28 ≥3.2 **PLUS** a ≥0.6 increase in DAS28 compared to lowest DAS28 after the first treatment course at baseline (i.e Visit 2) **and/or** a ≥20% increase in both tender and swollen joint counts compared to lowest counts after the first treatment course at baseline (i.e Visit 2).

Note: A moderate EULAR response is only considered to be valid if it is recorded at least 6 weeks after date of last infusion to allow washout of any positive effects of corticosteroid treatment from the pre-infusion treatment. Similarly the response is only valid if recorded at least 4 weeks after any i.a injection of corticosteroid.

For the second and subsequent re-treatment courses the following criteria apply:
After the first re-treatment course, patients will be eligible to receive further ofatumumab re-treatment courses during the 120-week Open-label Period if she/he:

- Achieves an efficacy response to ofatumumab at least once within 24 weeks from the last ofatumumab re-treatment course, defined as at least a moderate EULAR response and/or ≥20% improvement in both swollen and tender joint counts (compared to baseline value from Double-blind Period, i.e., Visit 2).

  PLUS

Following this response, subsequently shows an worsening in disease activity at any time since the last ofatumumab re-treatment course defined as:

- DAS28 ≥ 3.2 **PLUS** a ≥0.6 increase in DAS28 compared to lowest DAS28 after the last re-treatment course **and/or** a ≥20% increase in both tender and swollen joint counts compared to lowest counts since the previous treatment course

Note: A moderate EULAR response or a ≥20% improvement in both swollen and tender joint counts is only considered to be valid if it is recorded at least 4 weeks after any i.a injection of corticosteroid. Additionally, the joint injected will be scored as swollen and tender in joint count evaluation if performed within 12 weeks following the i.a. joint injection.

The interval between initiation of a new treatment course should be at least 16 weeks irrespective of progression in disease activity. Sixteen weeks are counted from infusion A from the preceding infusion course. Assessments will be performed every 8 weeks (± 2 weeks) independent of disease activity. If disease activity progresses between scheduled visits, an unscheduled visit should be performed. Pain management in the form of i.a. corticosteroid injection, analgesics or NSAIDs can be given to patients. If re-treatment criteria are fulfilled, infusion visits (two visits separated by 14 days) should be planned as soon as possible, and no later than 14 days after worsening of disease activity, in parallel to the scheduled visits performed every 8 weeks (please refer to separate Flow Chart in Section 2 for assessments to be done at infusion visits). An infusion visit and a scheduled visit could be combined if within the visit windows of the scheduled visit. The last treatment course should be planned to occur no later than the scheduled visit at week 120 after baseline (Visit 2).

If a patient attends an unscheduled visit at any time during the treatment period of the study, it is highly recommended to do the assessments which are performed at open-label non-infusion visits.

Patients who do not obtain at least a moderate EULAR response or ≥20% improvement in both swollen and tender joint counts (compared to baseline values from Double-blind Period, i.e., Visit 2) within 24 weeks of their last infusion in the Open-label Period will be withdrawn from further ofatumumab treatment and proceed to the Follow-up Period.
In addition, any patient with a circulating IgG level <lower limit of normal (as assessed by the central laboratory) at any time after the second treatment course will be withdrawn from further ofatumumab treatment and enter the Follow-up Period.

6.5 Follow-up Period

Patients who have completed the Open-label Period or are withdrawn from the Double-blind or Open-label Period of the trial (Section 5.3 and Section 17) will be followed every 12 weeks until B-cells, and circulating IgG have returned to normal (according to central laboratory) or baseline levels (i.e. Visit 2) or for a maximum of 2 years from the last scheduled visit in the Double-blind or Open-label Period, whichever occurs earlier. Neurological examinations and plasma/white cell JCV PCR testing will also be performed during Follow-up. At these visits, patients will also be

followed for SAEs and concomitant RA medication. Patients who have been withdrawn due to low levels of IgG will in addition to the above also be followed until the level of circulating IgG has returned to normal (according to central laboratory) or baseline level (i.e. Visit 2), or for a maximum of 2 years from the last scheduled visit in the Double-blind or Open-label period.

If the patient initiates treatment with other B-cell suppressive treatment (e.g., other anti-CD20 antibodies, cyclophosphamide, or azathioprine), at any time during the trial including the Follow-Up Period or enters another interventional trial while in the Follow-Up Period, all patient related study activities according to this trial protocol should be terminated. Furthermore, the investigator should document the date of initiation of other B-cell suppressive treatment or date of initiation of other trial and complete the End of Study Conclusion Form.

6.6 Treatment after the End of the Study

The investigator is responsible for ensuring that consideration has been given to the post-study care of the patient's medical condition.

Note: GSK will not provide any post study treatment with ofatumumab, and no compassionate use of ofatumumab for autoimmune indications is currently available for rheumatoid arthritis patients

6.7 Recruitment period

The recruitment period is approximately 14 months. However, some trial centres may participate for a shorter recruitment period by arrangement.

6.8 Number of Patients

The total number of patients to be randomized is 236.

See Section 10.11.

6.9 Randomization

Patients will be allocated to treatment in a 1:1 ratio to ofatumumab 700mg x2 or placebo x2 in addition to their background methotrexate treatment. Randomization will be stratified by rheumatoid factor seropositivity / -negativity, and region. The randomization will be restricted to include no more than 20% rheumatoid factor seronegative (RF-) patients.

An Interactive Voice Response System (IVRS) will be used for randomization of patients. The randomization procedure will be described in the IVRS manual.

6.10 Blinding

The investigator/trial staff at site, patient, and sponsor/partner trial personnel will be blinded to the trial treatment allocated to each individual patient in the Double-blind Period of the trial. At each trial site, a pharmacist or dedicated unblinded staff will receive information revealing trial treatment for the individual patients at that particular site.

Unblinding of treatment allocation for any patient will not take place until all data have been completed and source data verified for all patients up to and including visit 9 and database release has taken place. This also applies if a patient is withdrawn in the Double-blind Period unless it is medically justified to unblind the patient. Although this implies that no laboratory results will be revealed until after database release even though the patient has proceeded to Follow-up visits, selected laboratory results will be revealed to the investigator during the study as described in Section 8.2.

6.11 Emergency Unblinding procedure

Emergency unblinding of patients will take place by using the IVRS.

Unblinding is restricted to emergency situations and should only be used under circumstances where knowledge of the treatment is necessary for the appropriate clinical management or welfare of the subject. Whenever possible, the investigator must first discuss options with the GSK Medical Monitor or appropriate GSK study personnel **before** unblinding the subject's treatment assignment. If this is impractical, the investigator must notify GSK as soon as possible, but without revealing the treatment assignment of the unblinded subject, unless that information is important for the safety of subjects currently in the study.

GSK's Global Clinical Safety and Pharmacovigilance (GCSP) staff may unblind the treatment assignment for any subject with an SAE. If the SAE requires that an expedited regulatory report be sent to one or more regulatory agencies, a copy of the report, identifying the subject's treatment assignment, may be sent to clinical investigators in accordance with local regulations and/or GSK policy.

All SAEs and Suspected Unexpected Serious Adverse Reactions (SUSARs) will be unblinded within safety in accordance with regulatory requirements.

If the treatment blind is broken, the investigator will;

1) Contact the CRA (preferably before unblinding)

2) Document date and reason for unblinding in the Patient's Medical Record

3) Store the confirmation of unblinding from IVRS in the Investigator Site File

7 Treatment

7.1 Investigational Medicinal Product (IMP)

7.1.1 Ofatumumab

Ofatumumab is a clear colorless liquid. Ofatumumab is a concentrate for solution intended for intravenous administration.

Ofatumumab is formulated at 20 mg/mL . Ofatumumab is supplied in 6 mL clear glass vials. Each vial contains 5 mL of ofatumumab (20 mg/mL), i.e. a total of 100 mg ofatumumab.

Ofatumumab/placebo will be filtered using an inline filter (0.2 µm) during infusion.

7.1.2 Packaging and Labeling of ofatumumab/placebo

Ofatumumab will be supplied to the site or pharmacy in cartons, each carton containing 10 vials. Labeling will be in accordance with all applicable regulatory requirements.

7.1.3 Storage of ofatumumab

Ofatumumab should be stored in a safe and secure place in a refrigerator at 2-8°C, protected from light and it must not be frozen.

After ofatumumab has been diluted in sterile, pyrogen free 0.9% NaCl it can be kept at room temperature and must be given to the patient within 24 hours. Exact time of dilution into 0.9% NaCl must be written on the label of the infusion bag.

Drug supplies must be kept in an appropriate restricted area, which may be accessed only by the pharmacist, or a duly designated person. A log to document the temperature with daily readings (working days only) must be kept.

If the temperature of the refrigerator is outside the limits of 2-8°C (35.6-46.4 °F) it should be noted in the temperature log. If the temperature is / has been ≤ 0 °C or ≥ 10°C (≤ 32 °F or ≥ 50 °F) for more than 8 hours the local CRA should be contacted. The following information should be available: the study number, amount of ofatumumab and data on the temperature in the refrigerator, and for how long the temperature was outside the temperature limits. If a break down of the

refrigerator occurs, ofatumumab should be transferred to another temperature controlled refrigerator immediately.

Ofatumumab must not be utilized after the expiry date printed on the carton label.

7.1.4 Drug Accountability and Compliance Check

The investigator must ensure that a designated person receives ofatumumab shipments from sponsor and that all such shipments are:

- Recorded
- Handled and stored safely and properly
- Only dispensed to study patients according to the protocol
- Returned to sponsor if unused or disposed according to locally accepted procedures.

The Pharmacist or designee must keep drug inventory and accountability logs. The inventory will include details of ofatumumab received and dispensed to the patient, batch and ID numbers. All unused vials must be kept and returned to sponsor or alternatively be disposed at site according to locally accepted procedures after the reconciliation of delivery records with accountability logs by the CRA. After accountability of empty used vials has been performed by the CRA, these can be destroyed at site. Accountability must be made of any drug deliberately or accidentally destroyed. Discrepancies between the amount of received and dispensed drug must be reconciled.

Disposal of hazardous material, e.g., syringes, needles, etc. must conform to applicable local laws and regulations.

7.2 Dosage of ofatumumab/placebo

Apart from the unblinded pharmacist (or delegated person) and the unblinded pharmacy CRA, all other trial staff (which include the investigator, other site trial staff, the patient, and sponsor or delegated trial personnel) will be blinded to the treatment allocated to each individual patient in the Double-blind Period. In the Double-blind Period each patient will receive 2 infusions of ofatumumab or placebo separated by 14 days (one treatment course). In the Open-label Period all further re-treatment courses will be administered according to Section 6.4.2 and will not exceed the number of re-treatment courses outlined in Table 1.

Table 1 : Ofatumumab/placebo dosages

1st treatment course	2nd treatment course	3rd treatment course	4th treatment course	5th treatment course	6th treatment course	7th treatment course	8th treatment course
2 x 700 mg / 2 x placebo	2 x 700 mg	2 x 700 mg	2 x 700 mg	2 x 700 mg	2 x 700 mg	2 x 700 mg	2 x 700 mg

7.3 Handling of ofatumumab/placebo

7.3.1 Pre-Medication

Before each infusion, all patients must receive the following pre-medication ½ - 2 hours prior to start of treatment with ofatumumab. Data regarding pre-medication should be recorded in the eCRF.

Table 2: Pre-medication requirements before each infusion of ofatumumab/placebo

Time prior to infusion	Medication	Dose	Administration
30 min – 2 h	Paracetamol (acetaminophen) or equivalent	1 g*	p.o.
30 min – 2 h (i.v.) alternatively 1 h – 2 h (p.o.)	Antihistamine (cetirizine or equivalent)	10 mg*	i.v. alternatively p.o.
30 min – 2 h	Glucocorticoid (methylprednisolone or equivalent**)	100 mg	i.v.

* If an equivalent drug is chosen, dose range should be according to locally accepted practice.
** Methylprednisolone 100 mg corresponds to prednisolone 125 mg and hydrocortisone 500 mg.

7.3.2 Preparation of ofatumumab/placebo

The pharmacy at each trial site will be informed of the treatment assignment (ofatumumab or placebo) via IVRS. Preparation of infusion bags should be done on the day of planned infusion. Active treatment will be prepared as a 1000 mL dilution of ofatumumab in sterile, pyrogen free 0.9% NaCl. Seven vials equivalent to 35 mL are required to prepare the solution for infusion. 35 mL of the sterile, pyrogen free 0.9% NaCl should be removed from the infusion bag prior to mixing with ofatumumab. A detailed description with instructions for the preparation of ofatumumab will be supplied to each center or pharmacy.

Table 3: Ofatumumab infusion preparation

Dose Ofatumumab	Number of vials	Volume of ofatumumab
700 mg	7	35 mL

Placebo will be 1000 mL sterile, pyrogen-free 0.9% NaCl solution. To prevent unblinding the injection membrane on the infusion bag should be punctured with an injection needle.

7.3.3 Treatment Schedule

Ofatumumab / placebo will be administered as an intravenous (i.v.) infusion. The initial rate of the infusion will be 12 mL/hour for the first administration and 25 mL/hour for the second administration during each treatment course. During infusion, the rate will be doubled every 30 minutes to a maximum of 400 mL/hour. Thereafter, the rate will be increased with 200 mL/hour every 30 minutes until 800 mL/hour is reached. Duration of the infusion will be approximately 4 and 3½ hours respectively if this schedule is adhered to.

Table 4 : Infusion rate

1st infusion in each treatment course		2nd infusion in each treatment course	
**Time**	**mL/hour**	**Time**	**mL/hour**
0 – 30 minutes	12	0 – 30 minutes	25
31 – 60 minutes	25	31 – 60 minutes	50
61 – 90 minutes	50	61 – 90 minutes	100
91 – 120 minutes	100	91 – 120 minutes	200
121- 150 minutes	200	121- 150 minutes	400
151 – 180 minutes	400	151 – 180 minutes	600
181 – 210 minutes	600	+ 180 minutes	800
+ 210 minutes	800		

The infusion rate may be adjusted if any study drug related AEs occur. A detailed description with instructions for the administration of ofatumumab/placebo will be supplied to each center.

The actual administration time should be recorded including any changes in the infusion rate.

7.3.4 Administration of ofatumumab/placebo

Ofatumumab/placebo must be administered by intravenous (i.v.) infusion through an in-line filter and through a well-functioning intravenous catheter (i.v. cannula) into a vein in the arm by an infusion pump. Please note that the injection site cannot be used for blood sampling.

The patient should be carefully observed (including blood pressure, heart rate, temperature, and adverse events) during infusion and 2 hours following the initial infusion in each treatment course and 1 hour for the second infusion in each treatment course. The study staff at the clinic must be prepared to intervene if an infusion reaction should occur. Special caution should be taken during the first infusion for each patient and during the infusion of a patient who did not tolerate a previous infusion well.

Following the infusion, the infusion line should be flushed with sterile, pyrogen free 0.9% NaCl. It

should be noted that all infusions must be completed or pre-maturely stopped within 24 hours from the preparation of ofatumumab. A detailed description with instructions for administration of ofatumumab/placebo will be supplied to each center.

7.3.4.1 Measurements during infusion

During infusion the patient should be monitored closely, and vital signs and PK sampling must be performed as per Table 5 at a minimum.

Table 5: Measurements and PK sampling during infusion

Time	Vital Signs: Temperature, blood pressure and heart rate	PK sampling[a)]
Before infusion	X	X
Every hour during infusion	X	
End of infusion	X	X [b)]
+1 hour	X	
+2 hours [c)]	X	
> 2 hours after end of infusion	X [d)]	

Table notes:
a) PK-sampling should be done prior to infusion and immediately after end of infusion at the first, second, fourth and sixth treatment courses
b) PK sampling at "end of infusion" is immediately after the infusion line has been flushed with sterile, pyrogen free 0.9% NaCl.
c) Only applicable after the first infusion in each treatment course. Otherwise the observation period is one hour.
d) If the patient remains at clinic after two hours from ended infusion, due to trial drug induced toxicity, vital signs will be measured every hour until the patient leaves the clinic.

The following data should be documented:

- Time for observations / measurements
- Infusion rate including all changes in infusion rate
- Pre-medication and medication given during infusion including information of dose
- All measurements during infusion
- Adverse events

7.3.4.2 Handling of Infusion Related Reactions

Previously observed infusion related AEs are described in the Investigator's Brochure, Investigator's Brochure Supplement 1, and Section 3.2.5. Emergency equipment should always be available at the clinic during the infusions. In case of adverse events during infusion the patients must be treated according to the investigator's judgment and best clinical practice. Guidance on the management of infusion related reactions may also be found in the Study Manual.

Infusion related Adverse Events may lead to a prolonged infusion time. Overnight stay at the hospital due to slow infusion rate shall not be considered a Serious Adverse Event (SAE).

Interruption, restart and increasing the rate of the infusion depending on the severity of the adverse event must be according to the description below and at the investigator's discretion. Increase of the infusion rate after an interruption must not exceed the schedule in Table 4 (i.e. not more than doubled rate and no earlier than every 30 minutes).

Please see description below:

Mild and moderate intensity

If the investigator judges the AE to be related to the infusion, the infusion must be paused.

When the patient's condition is stable, the infusion can be restarted according to the judgment of the investigator.

Upon restart, the infusion rate should be half of the infusion rate at the time the infusion was paused. If, however, the infusion rate was 12 mL/hour before the pause, the infusion should be restarted at 12 mL/hour.

Hereafter, the infusion rate may be increased according to the judgment of the investigator, in the manner described in Table 4 (i.e. not more than doubled and no earlier than every 30 minutes).

Severe intensity

If the investigator judges the AE to be related to the infusion, the infusion must be paused. The patient should be observed for 2 hours. If AE remains severe the patient should be withdrawn from further treatment. If the AE decreases to moderate the investigator must judge if the infusion should be restarted.

Upon restart, the infusion rate must be 12 mL/hour (the first infusion in each treatment course) or 25 mL/hour (second infusion in each treatment course), but may subsequently be increased according to the judgment of the investigator, in the manner described in Table 4 (i.e. not more than doubled and no earlier than every 30 minutes).

7.4 Concomitant Therapy

7.4.1 Therapy Allowed during Trial

Patients may receive their current medication for non-RA conditions.

With respect to current RA medication, the following will be allowed under the restrictions described below. Adjustments in concomitant RA medication is prohibited unless due to toxicity.

Methotrexate and Folic Acid

All patients will continue receiving stable doses of methotrexate (7.5 - 25 mg/week, p.o., i.m. or s.c.) throughout the trial. This dose should be identical to the dose received for the last four weeks prior to screening. During the study, increases in dose and change of dosage form compared to the

dosage form used at baseline of MTX are not allowed. Decreases in dose of MTX are permitted only if due to toxicity and must be kept within 7.5 - 25 mg/week.

Temporary interruption of MTX dosing for the management of intolerance will be permitted for up to 14 days (i.e., 2 missed weekly doses). If a longer period of interruption is required, the subject will be withdrawn from further ofatumumab treatment. Temporary interruption of MTX may be done no more than twice during the double-blind period up to and including Week 20. In the Open-label period, dose reduction/interruption of MTX is permitted as clinically indicated.

All patients will receive folic acid ≥5 mg/week starting 4 weeks prior to baseline (Visit 2) until the last visit in the Open-label Period administered according to locally accepted practice.

Oral Corticosteroids

Continued use of oral corticosteroids ≤ 10 mg/day prednisolone or equivalent are allowed if the dosage was stable for at least four weeks prior to screening. During the Open-label period, if indicated by clinical status, adjustment may be made to either decrease or increase the dose of oral corticosteroids (to a maximum dose of ≤ 10 mg/day prednisolone or equivalent).

NSAIDs

Continued use of NSAIDs is permitted (e.g. diclofenac, ibuprofen, ketoprofen, naproxen) in daily doses up to the maximum recommended according to locally accepted clinical practices if the dosage was stable for at least 2 weeks before screening. If the patient is not regularly using NSAIDs, he/she may take the NSAIDs mentioned above as breakthrough pain management. However, the patients should be advised not to take any NSAIDs within 12 hours prior to attending a trial visit.

During the Open-label period, if indicated by clinical status, adjustment may be made to either decrease or increase the dose of NSAIDs in accordance with locally accepted clinical practice.

Analgesics

Regular use of codeine, opium alkaloid, paracetamol/acetaminophen, propoxyphene, and tramadol are permitted in daily doses up to the maximum recommended according to locally accepted clinical practices. If the patient is not regularly using any analgesics, he/she may take the analgesics mentioned above as breakthrough pain management. However, the patients should be advised not to take any analgesics within 12 hours prior to attending a trial visit.

Intra-articular Corticosteroids

One intra-articular (i.a.) injection of corticosteroid (80 mg methyl prednisolone) or equivalent (see Table 6) in one joint is allowed every 6 months during the trial. To avoid confounding the primary endpoint i.a. injections will not be permitted from week 20 to week 24. Responses (defined as at least a moderate EULAR response **or** ≥20% improvement in swollen and ≥20% improvement in tender joint counts (compared to baseline values) obtained in the period from time of an i.a. corticosteroid injection and 4 weeks forwards are considered related to this injection and will not be regarded as a response to ofatumumab (will not trigger re-treatment).

Table 6: Maximum doses for i.a. corticosteroids

Generic Name	Brand Name (may be country specific)	Maximum Allowed Dose
Triamcinolone hexa-acetonide	Lederspan	20 mg
Triamcinolone acetonide	Kenalog	40 mg
Methyl prednisolone	Depo-Medrol	80 mg
Dexamethasone	Decadron	8 mg
Betamethasone-17-valerate, Betamethasone-21-acetate, Betamethasone phosphate	Celestona	12 mg
Betamethasone	Diprospan	14 mg

Rescue medication from week 16

If a moderate EULAR response based on the DAS28 score (please refer to Section 10.3.4) is not achieved at week 16 rescue medication may be considered. Rescue medication is defined in Table below:

Table 7: Definition of rescue medication

Allowed nonbiologic DMARDs	Approved dose interval
Sulphasalazine	1 g - 3 g/day
Hydroxychloroquine	200 mg – 400 mg/day
Methotrexate	7.5mg - 25mg/week
Chloroquine	250 mg – 500 mg/day
Corticosteroids	≤ 10 mg/day

If rescue medication is used the patient can not continue in the open-label extension period of the study.

7.4.2 Prohibited therapy and procedures during the study

The following medications and therapies will <u>not</u> be allowed as concomitant therapy from 4 weeks prior to visit 2 and until completion of the study:

- I.a., i.m. and i.v. corticosteroids (apart from i.a corticosteroid injections, as described in 7.4.1, and apart from i.v. glucocorticoid given as pre-medication, see 7.3.1)
- Anti-cancer therapy, immuno therapy, or chemo therapy
- Any non-marketed drug substance or experimental therapy
- Narcotics other than specified under analgesics in Section 7.4.1

Exposure to biologic DMARDs during the study will not be allowed. For drug product specific wash-out period prior to Visit 2 please refer to Section 5.2.

8 Trial Assessments

An overview of visits is given in a Flow Chart in Section 2. The trial consists of a 24 week Double-blind Period followed by a 120 week Open-label Period. After the last visit in the 120 week Open-label Period, patients will be followed every 12 weeks (Follow-up Visits) until normalization of circulating IgG and $CD19^+$ cells for a maximum of 2 years from the last scheduled visit in the Double-blind or Open-label Period.

8.1 Clinical Assessments

All trial assessments should be done prior to blood sampling and infusion unless stated otherwise.

8.1.1 Demographics

Date of birth, race and sex will be obtained by the site staff and recorded.

8.1.2 Medical History

Any relevant prior or current diseases will be obtained by the site staff. Furthermore, the following information regarding RA will be recorded:

- Date of diagnosis of RA
- RA functional class (I, II, or III)
- Previous RA treatment(s) including reason for discontinuation

8.1.3 Height and Body Weight

Height (without shoes) will be measured by the site staff and recorded rounded to nearest centimeter. Body weight (without overcoat and shoes) will be measured and recorded rounded to nearest kilogram.

8.1.4 Physical Examination

A physical examination will be performed by the physician. It will include but is not limited to: general appearance and the following body systems: Lymph nodes, mouth and throat, lungs, cardiovascular system, abdomen, extremities, musculo-skeletal system, neurological system and skin. All abnormal findings judged by the investigator to be clinically relevant, should be reported as AEs.

8.1.5 Neurological Questionnaire

A neurological questionnaire to detect any signs or symptoms consistent with the diagnosis of PML will be conducted as a part of the physical examination required by protocol. A questionnaire will be provided. If any question is answered 'yes' the Investigator should contact the medical monitor to discuss appropriate management of the patient. Any findings should be reported as an adverse event and the PML reporting procedures followed, Section 9.5.3.

8.1.6 Electrocardiogram

Electrocardiograms (ECG) taken according to local clinical practice will be performed.

An overall interpretation of the ECG will be performed by the investigator, or the investigator may delegate this task to a cardiologist, if applicable. The date of the ECG and the overall interpretation of the ECG will be recorded. The ECG recordings will be kept in the patient's record.

If an ECG taken during the trial is abnormal and judged by the investigator to be clinically relevant, the patient should be withdrawn from further trial treatment and proceed to Follow-up.

8.1.7 Vital Signs

Vital signs including temperature, blood pressure, and heart rate will be measured and recorded by the site staff. Further information about measurements during infusion is described in Section 7.3.4.1.

Wherever possible, body temperature must be measured using the same method (e.g. an ear thermometer) and the position (e.g. sitting or lying) for measuring blood pressure and heart rate must be consistent throughout the study for each individual patient.

8.1.8 Adverse Events

The reporting of adverse events is described in Section 9 (Adverse Events).

8.1.9 Concomitant Medication

All concomitant medication taken during the study will be recorded. Any medication other than the trial drug is considered concomitant medication (including methotrexate, and medication given as pre-medication prior to infusions of ofatumumab / placebo).

8.1.10 Administration of IMP

A description of the procedures for administration of IMP is described in Section 7 and in the Study Procedures Manual.

8.1.11 Chest X-Ray

A chest X-ray will be performed to evaluate for tuberculosis. Postero-anterior projections and, if indicated, lateral projections will be taken. A chest X-ray with the same projections, taken within 12 weeks prior to Visit 1, may also be used. However, if this previous x-ray is inconclusive and if clinically warranted the x-ray should be repeated at screening.

Reading of the chest X-ray will be done by a radiologist. A copy of the radiologist's evaluation reports should be kept in the patient's record. The date of the chest X-ray and the overall interpretation of evaluation will be recorded.

8.1.12 Mantoux test

An intradermal injection of tuberculin (e.g., Mantoux test or equivalent) should be performed according to locally accepted practice to evaluate for latent TB infection. This skin test should be done at any time between the screening visit and baseline; subjects with documented BCG vaccination are exempted. Subjects with a positive skin tuberculin test will be excluded if the investigator judges the patient to be at risk of latent TB infection.

8.1.13 Clinical Efficacy Assessments

The clinical efficacy assessments must be performed prior to blood sampling, and the administration of pre-medication and trial drug.

8.1.13.1 Joint Assessments

The 66/68 joints to be assessed for both the left and the right sides of the patient are:

Temporomandibular, sternoclavicular, acromioclavicular, shoulder, elbow, wrist, metacarpophalangeal (first (thumb), second, third, fourth, fifth), proximal interphalangeal (thumb (interphalangeal), index, middle, ring, little), distal interphalangeal (second, third, fourth, fifth), hip (assessed for tenderness only), knee, ankle, tarsus, metatarsophalangeal (first, second, third, fourth, fifth) and proximal interphalangeal (toe) (first, second, third, fourth, fifth).

Tender Joint Count:
A total of 68 joints should be assessed. Joints are classified as either tender or not tender.

Swollen Joint Count:
A total of 66 joints should be assessed. Joints are classified as either swollen or not swollen.

Replaced or Fused Joints
Replaced or fused joint will not be included in joint evaluations. The reason for absence of the evaluations of those joints must be recorded.

Intra-articular corticosteriod injections:

If a patient has received and intra-articular corticosteriod injection the affected joint should be counted as tender and swollen for 12 weeks following the injection.

Independent Joint Evaluator
One or more independent assessors, who have documented experience in performing joint assessments, will be designated at each trial site to perform joint assessments. Preferably the independent assessor will perform all joint assessment for the same patient throughout the trial. The Principal Investigator must ensure that the independent joint assessor has documented experience and he/she is adhering to locally accepted and implemented standards. This also applies if the independent joint assessor is replaced during the trial.

The independent joint assessor should have no other contact with the patient during the trial, must not be the treating physician (investigator), should not discuss the patient's clinical status with the patient during the joint assessment nor with other site personnel, and will not be permitted to review the patient's medical records, the eCRF, nor any of the previous joint assessments.

The independent joint assessor will perform only the joint assessment, and will not be involved in any other assessments in this trial.

8.1.13.2 Visual Analogue Scale (VAS) of Pain

A horizontal visual analogue scale (VAS) of 100 mm will be used to report the patient's level of joint pain. The scale ranges from ‘no pain’ to ‘unbearable pain’. The patient should be instructed to place a vertical mark on the line to indicate the severity of the pain.

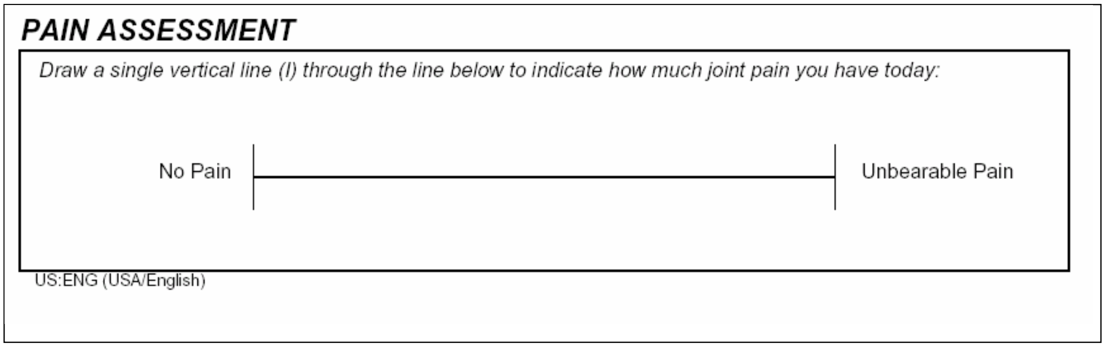
PAIN ASSESSMENT

Draw a single vertical line (I) through the line below to indicate how much joint pain you have today:

No Pain ⊢——⊣ Unbearable Pain

US:ENG (USA/English)

The distance from the *no pain* end to the vertical line drawn by the patient is his/her joint pain score. The site staff or designee will measure this distance with a standard ruler scored in mm starting from *no pain* to the line marked by the patient and record that measurement (in mm). Patients should be instructed not to place a mark outside of the end markers. If this occurs, the data will be non-evaluable.

8.1.13.3 Patient and Physician VAS of Global Disease Assessment

Both the patient and the physician will use the horizontal VAS for overall assessment of the disease. The scale ranges from ‘very well’ to ‘very poor’. The evaluator and patient must complete the global assessment independently from each other.

The evaluator performing the global assessment cannot be the independent joint assessor. The results of the joint assessment performed by the independent joint assessor will be available to the physician assessing the patient's global disease.

PATIENT'S GLOBAL ASSESSMENT OF ARTHRITIS CONDITION

Considering all the ways that your arthritis affects you, draw a single vertical line (|) through the line below to indicate how your arthritis is today.

Very Well |————————————| Very Poor

US:ENG (USA/English)

It will be anchored at the 0 mm with "very well" and anchored and at 100 mm end with "very poor". Using a standard ruler the site staff will measure and document the distance in mm from the left end marker to the point at which the patient's mark intersects the horizontal line. Patients should be instructed not to place a mark outside of the end markers. If this occurs, the data will be considered non-evaluable.

8.1.14 Patient Reported Outcomes

Questionnaires will be handed out to the patient for each assessment. If a patient is unable or refuses to complete a whole questionnaire; the reason for this should then be ascertained and recorded.

8.1.14.1 Disability Index of the Health Assessment Questionnaire (HAQ-DI)

The functional status of the patient will be assessed by means of the Disability Index of the Stanford Health Assessment Questionnaire (HAQ-DI). This 20-question instrument assesses the degree of difficulty a person has in accomplishing tasks in eight functional areas (dressing, arising, eating, walking, hygiene, reaching, gripping, and errands and chores). Responses in each functional area are scored from 0 indicating no difficulty, to 3, indicating inability to perform a task in that area.

The index is calculated by the addition of the scores, then dividing this score by the total number of components answered.

8.1.14.2 SF-36

The Short-Form 36 (SF-36) is a validated health survey that contains 36 questions which should be answered by the patient. The SF-36 yields an eight-scale profile of functional health and well-being scores as well as psychometrically-based physical and mental health summary measures. The standard 4-weeks recall questionnaire will be used.

8.1.14.3 FACIT-Fatigue

The Functional Assessment of Chronic Illness Therapy (FACIT) questionnaire is a validated tool designed to manage chronic illnesses. The subset determining fatigue contains 13 questions. Responses are scored from 0 indicating 'Not at all', to 4, indicating 'Very much'.

8.2 Laboratory Assessments

All samples should be drawn prior to infusion unless stated otherwise.

Except for urine pregnancy testing and ESR, which will be performed locally, all laboratory samples will be sent to a central laboratory (QUEST) for analysis. The central laboratory may utilize subcontractors.

A detailed description of the procedures for sampling, handling, storage, and shipment of the laboratory samples and all material such as test tubes and labels will be provided in the Study Procedures Manual. The Manual and the result reports will include all reference ranges.

The individual patient's laboratory results (apart from the Visit 1 measurements, biochemistry, hematology (not including lymphocyte levels) and the urine test) obtained during the Double-blind Period will be not revealed to the investigator. However, all plasma/ white cell JCV PCR results will be revealed to the investigator.

Treatment allocation will be revealed when all patients have completed week 24 (primary endpoint), all data are entered into the database, and all data have been declared clean in order to keep the blinding.

If the patient is withdrawn in the Double-blind Period of the trial the individual patient's blinded laboratory results levels of IgG, IgM, and $CD19^+$ cells will NOT be revealed to the investigator (or sponsor) unless medically justified.

Relevant previously blinded laboratory results obtained from the first scheduled visit after the patient has received the second treatment course and beyond in the Open Label Period will be revealed to the investigator and the sponsor.

8.2.1 Biochemistry, Hematology and Urine test

Blood and urine samples will be shipped to the central laboratory for immediate analysis of the following parameters:

- Biochemistry: Serum electrolytes (sodium, potassium, chloride, bicarbonate, calcium), creatinine, liver enzymes (AST/SGOT and ALT/SGPT), alkaline phosphatase (ALP), total protein, albumin, total bilirubin, uric acid, gamma glutaryl-transferase (GGT), alkaline phosphatase (ALP), blood urea nitrogen (BUN), lactic dehydrogenase (LDH), creatinine phosphokinase (CPK)
- Hematology: Hemoglobin, red blood cell (RBC) count, hematocrit, platelet count, and white blood cells (WBC) with differential count
- Urine test: Protein, glucose, ketones, blood and WBC.

Date of sampling will be recorded.

8.2.2 Pregnancy Test

For women of childbearing potential pregnancy testing will be performed. Blood samples will be drawn at screening, end of Double-Blind Period/early withdrawal, and at end of Open-Label Period. Serum samples are shipped to the central laboratory for immediate analysis of Human Chorionic Gonadotrophine (HCG).

If the patient is an early withdrawal from treatment in the Double-blind Period and continues with the remaining visits serum-HCG testing is not applicable at the early withdrawal from treatment visit. Instead, the serum-HCG testing will be done at the end of the double-blind period, i.e. when the patients attend the last visit prior to progressing to the follow-up period.

All remaining pregnancy tests (including a test at week 12 and 24 from the last scheduled visit in the Double-Blind or Open-label Period) will be based on urine sampling and will be analyzed locally. A negative urine pregnancy test should be obtained and documented. If the urine pregnancy test is positive a serum-HCG pregnancy test should be obtained. Protocol assessments can only be re-commenced if results of the serum pregnancy test are negative.

In the Open-label Period monthly urine pregnancy testing should be performed for women of childbearing potential. If a visit is not scheduled, home urine testing must be performed.

Women are considered of childbearing potential unless they have been hysterectomized, or have undergone tubal ligation at least one year prior to Visit 1 (Screening), or have been postmenopausal for at least one year.

The date of sampling and outcome will be noted. If sampling for pregnancy test is not applicable, the reason for this will be documented.

8.2.3 IgA, IgG, IgM

Blood samples for measurement of IgA, IgG and IgM will be drawn and shipped to the central laboratory.

The date of sampling will be documented.

8.2.4 Flow Cytometry

Blood samples will be drawn and shipped to the central laboratory for analysis of $CD19^{+,}$ $CD3^{+}$, $CD4^{+}$, and $CD8^{+}$ cells.

The date of sampling will be documented.

8.2.5 JC Virus

Blood samples will be drawn and shipped to the central laboratory for analysis of plasma / white cell JC Virus (JCV).

The date of sampling will be documented.

8.2.6 Host Immune Response (HAHA)

Blood samples will be drawn and shipped to the central laboratory for analysis of Human Anti-Human Antibodies (HAHA).

The date of sampling will be documented.

8.2.7 Pharmacokinetics (PK)

Blood samples will be drawn at the Visits marked in the Flow Chart in Section 2, and shipped to the central laboratory for analysis of concentrations of ofatumumab.

At some of the visits where infusions will take place a more extended PK sampling will be done (see Table 5).

The date and time of sampling will be recorded.

8.2.8 Hepatitis B and C

Patients will be evaluated for serologic evidence of Hepatitis B (HB) infection based on the results of testing for HBsAg, anti-HBc and anti-HBs antibodies as follows:

- Patients positive for HBsAg are excluded
- Patients negative for HBsAg but positive for both anti-HBc and anti-HBs antibodies are eligible to participate.
- Patients negative for HBsAg and anti-HBc antibody but positive for anti-HBs antibody are eligible to participate.
- Patients negative for HBsAg and anti-HBs antibody but positive for anti-HBc antibody will require clarification of their status by testing for HBV DNA which if positive will exclude the patient from participation.

Patients with documented vaccination against Hepatitis B (primary and secondary immunization and booster) will be considered negative.

If at any time during the study patients test positive by the criteria described above (and this is not due to a previous vaccination), the patient should be withdrawn from further trial treatment and proceed to Follow-up.

A blood sample will also be collected and analyzed for Hepatitis C antibodies at screening, and

every 24 weeks during follow-up for a maximum of 2 years from the last scheduled visit in the Double-blind or Open-label Period. If the result is positive the viral load for Hepatitis C will be analyzed in another blood sample by a confirmatory assay. Both blood samples will be taken on the same day but will be stored and shipped ambient or frozen, respectively.

8.2.9 Biomarkers

Measurements of Rheumatoid Factor (RF) are used to characterize the disease activity and the immune status of the patient. Blood samples will be taken to determine whether the patient is RF seropositive or seronegative and to verify the quantitative proportion of IgM-, IgG-, and IgA-RF.

Other biomarkers which characterize disease type, immune status, and transcriptional profiles will include, but are not limited to the following, Rheumatoid factor (RF), Anti-Cyclic Citrullinated Peptide antibody (Anti-CCP) and B-Lymphocyte Stimulator (BLyS). Samples will be taken at visits indicated in the Flow Chart in Section 2 and may be assayed on arrival or stored at the central laboratory prior to analysis. Samples will be stored securely and may be kept for up to 15 years after the last patient completes the study or the sponsor may destroy the samples sooner.

The date of sampling will be documented.

8.2.10 Transcriptomics

Whole blood will be collected for transcriptomic analysis of mRNA at Baseline (Visit 2) as indicated in the Flow Chart in Section 2. The blood sample will be frozen prior to shipment to the central laboratory for further frozen storage and analysis of a subset of patients as indicated by the clinical response to ofatumumab. Samples will be stored securely and may be kept for up to 15 years after the last patient completes the study or the sponsor may destroy the samples sooner.

The date of sampling will be documented.

8.2.11 Pharmacogenetics

Information regarding pharmacogenetic research is included in Appendix 2. The IEC/IRB and, where required, the applicable regulatory agency must approve the PGx assessments before these can be conducted at the site. The approval(s) must be in writing and will clearly specify approval of the PGx assessments (i.e., approval of Appendix 2). In some cases, approval of the PGx assessments can occur after approval is obtained for the rest of the study. If so, then the written approval will clearly indicate approval of the PGx assessments is being deferred and in most cases, the study, except for PGx assessments, can be initiated. When PGx assessments will not be approved, then the approval for the rest of the study will clearly indicate this and therefore, PGx assessments will not be conducted.

The PGx sample may be collected at any visit during the trial.

8.2.12 ACR/DAS28: Laboratory Efficacy Assessments (CRP and ESR)

A blood sample will be taken at screening (Visit 1) to determine eligibility with respect to ESR/CRP. Furthermore, blood samples will be taken during the study for determinations of CRP and ESR. ESR is to be included in the assessments of DAS28 responses.

The dates of sampling will be recorded.

Blood samples for ESR will be measured immediately at trial site using the Westergren method. Blood samples for CRP will be sent to the central laboratory for analysis.

The date of sampling and results will be documented.

9 Adverse Events

9.1 Characterization

9.1.1 Definition of Adverse Events

An AE is any untoward medical occurrence in a patient administered a pharmaceutical product and which does not necessarily have a causal relationship with this treatment.

AEs include the following:

- All suspected Adverse Drug Reactions (ADR)
- All reactions from medication overdose, abuse, withdrawal, sensitivity, or toxicity
- Apparently unrelated illnesses, including the worsening of a pre-existing illness
- Injury or accidents. Note that if a medical condition is known to have caused the injury or accident, the medical condition and the accident should be reported as two separate AEs
- Abnormalities in physiological testing or physical examination findings that require clinical intervention or further investigation
- Laboratory abnormalities that are clinically significant (e.g. that require clinical intervention or further investigation) unless they are associated with an already reported clinical event
- Significant worsening of an abnormal laboratory value present at baseline

9.1.2 Pre-existing Conditions

In this study, a pre-existing condition (i.e., a disorder present before the AE reporting period started and noted on the medical history/physical examination form) should not be reported as an AE unless the condition worsens or episodes increase in frequency during the AE reporting period.

9.1.3 B cell depletion and hypogammaglobulinemia

B cell depletion and hypogammaglobulinemia with respect to IgG due to ofatumumab treatment should not be reported as an AE.

9.1.4 Study Disease

Signs and symptoms, which according to the investigator are expected and well known consequences of the RA, both in intensity, and frequency, should not be reported as AEs unless they meet any of the specified seriousness criteria.

9.1.5 Procedures

Diagnostic and therapeutic non-invasive and invasive procedures, such as surgery, should not be reported as AEs. A medical condition for which an unscheduled procedure was performed, should however be reported if it meets the definition of an AE. For example, an acute appendicitis should be reported as the AE and not the appendectomy.

9.1.6 Adverse Event Reporting

The investigator must report all directly observed AEs and all AEs spontaneously reported by the patient. A general type of question should be used similar to, "Do you have any health problems?" or, "Have you had any health problems since your last visit?"

All AEs that occur in patients during the AE reporting period must be reported, whether or not the event is treatment related.

The AE reporting period (for SAE reporting period, see Section 9.3.2) begins from first treatment (Visit 2/Day 0) until the patient terminates the trial. Any signs or symptoms occurring between Screening/Visit 1 and Visit 2 should be recorded as Medical History. During the Follow-Up Period when the patient's B-cells and IgG (if applicable) and IgG levels are followed, only AEs that meet one or more of the serious criteria (see Section 9.3.1) will be reported to sponsor. The Serious Adverse Event Form in the eCRF should be completed.

9.1.7 Pregnancy

Any pregnancy that occurs during study participation must be reported. To ensure patient safety, each pregnancy must be reported to sponsor within 2 weeks of learning of its occurrence. The pregnancy must be followed up to determine outcome (including premature termination) and status of mother and child. Pregnancy complications and elective terminations for medical reasons must be reported as an AE or SAE. Spontaneous abortions must be reported as an SAE.

Any SAE occurring in association with a pregnancy brought to the investigator's attention after the patient has completed the study and considered by the investigator as possibly related to the investigational product, must be promptly reported to sponsor.

In addition, the investigator must attempt to collect pregnancy information on any female partners of male study patients who become pregnant while the patient is enrolled in the study. Pregnancy information must be reported to sponsor as described above.

9.2 Reporting Instructions

9.2.1 Recording

All AEs are to be recorded. If the AE is judged by the investigator to meet the criteria for a SAE, the SAE form should be completed.

9.2.2 Diagnosis

Diagnosis should be recorded if available. If no diagnosis is available each sign and symptom should be recorded as individual AEs.

9.2.3 Intensity

The Investigator will use the adjectives MILD, MODERATE or SEVERE to describe the maximum intensity of the AE. For purposes of consistency, these intensity grades are defined as follows:

MILD	Does not interfere with patient's usual function (awareness of symptoms or signs, but easily tolerated); acceptable
MODERATE	Interferes to some extent with patient's usual function (enough discomfort to interfere with usual activity); disturbing
SEVERE	Interferes significantly with patient's usual function (incapacity to work or to do usual activities); unacceptable

Note the distinction between the gravity and the intensity of an AE. Severe is a clinical judgment of intensity; thus, a severe reaction is not necessarily a serious reaction. For example, a headache may be severe in intensity, but would not be classified as serious unless it met one of the criteria for serious events, Section 9.3.1.

The grade assigned by the investigator should be the most severe, which occurred during the AE period.

9.2.4 Relationship to Study Drug

The investigator must assess the AE as either Related (possible/probable) or Not Related. If relationship changes over time the last judgment by the investigator should be reported. Relatedness has to be assessed and reported from first time the AE is being reported.

9.2.5 Outcome

Outcome of the AE must be judged by investigator by the following terms:

- Recovered
- Recovered with sequelae
- Not recovered
- Death
- Unknown

Instructions for reporting changes in an ongoing AE during a patient's participation in the study are provided in the instructions that accompany the AE CRF.

9.3 Serious Adverse Events (SAE)

9.3.1 Definition of a SAE

Each AE is to be classified by the investigator as Serious or Non-Serious. This classification of the gravity of the event determines the reporting procedures to be followed.

An AE that meets one or more of the following criteria/outcomes is classified as Serious:

- Requires inpatient hospitalization or prolongation of existing hospitalization
- Results in persistent or significant disability/incapacity
- Is a congenital anomaly/birth defect
- Medically important
- Results in death
- Is life-threatening

The term “life-threatening” in the definition of “serious“ refers to an event in which the patient was at risk of death at the time of the event; it does not refer to an event which hypothetically might have caused death if it were more severe.

Elective surgery or other scheduled hospitalization periods that were planned before the patient was included in this study are not to be considered serious. However, the event must be reported on the AE form in the eCRF and commented upon.

Overnight stay at hospital due to prolonged infusion time will not be reported as an SAE.

Medical and scientific judgment must be exercised in deciding whether an AE is believed to be “medically important”. Medical important events may not be immediately life-threatening or result in death or hospitalization but may jeopardize the patient or may require intervention to prevent one of the other outcomes listed in the definition above.

9.3.2 Reporting of SAEs

SAEs related to study participation will be collected from the time of informed consent. All SAEs will be collected from the time of first treatment (Visit 2) until the patient’s last follow-up visit. However, if a SAE suspected by the investigator to be related to study medication occurs after the last follow-up visit, this must be reported at all times.

SAEs, pregnancies and liver function abnormalities meeting pre-defined criteria will be reported promptly to sponsor as described in the following table once the investigator determines that the event meets the protocol definition for that event.

Table 8 : Timelines for reporting SAEs, pregnancies and liver function abnormalities

	Initial Reports		Follow-up Information on a Previous Report	
**Type of Event**	**Time Frame**	**Documents**	**Time Frame**	**Documents**
All SAEs	24 hours	“SAE” data collection tool	24 hours	Updated “SAE” data collection tool
Pregnancy	2 Weeks	Pregnancy Form	2 Weeks	Updated Pregnancy Form
Liver chemistry abnormalities:				
ALT≥3xULN PLUS Bilirubin≥1.5xULN)	24 hours	Liver Chemistry Report Form	24 hours	Updated Liver Chemistry Report Form
ALT≥5xULN or ALT≥3xULN with hepatitis or rash or ≥4 weeks	24 hours	Liver Chemistry Report Form	24 hours	Updated Liver Chemistry Report Form
ALT≥3xULN and <5xULN and biliribin <1.5xULN	24 hours	Liver Chemistry Report Form	24 hours	Updated Liver Chemistry Report Form

The method of detecting, recording, evaluating and follow-up of AEs and SAEs plus procedures for completing and transmitting SAE reports to sponsor are provided in the study procedures manual. Procedures for post-study AEs/SAEs are provided in the study procedures manual.

9.3.3 Suspected Unexpected Serious Adverse Reactions (SUSARs)

The sponsor has a legal responsibility to notify, as appropriate and according to local regulations, both the local regulatory authority and other regulatory agencies about the safety of the product under clinical investigation. Prompt notification of SAEs by the Investigator to sponsor is essential so that legal obligations and ethical responsibilities towards the safety of patients are met.

Sponsor will ensure that all relevant information about SUSARs is recorded and reported as soon as possible to the competent regulatory authorities and/or to the Ethics Committee according to the applicable local regulatory requirements.

9.4 Follow-Up on Adverse Events

All non-serious AEs should be followed until they are resolved i.e. returned to baseline or until end of trial, whatever comes first.

Related severe AEs and AEs meeting one of the criteria for SAEs, still ongoing after ended trial participation, should be followed on a regular basis, according to the investigator’s clinical judgment, until the event has been resolved or until the investigator assesses it as chronic or stable.

9.5 Safety Monitoring

9.5.1 Safety Surveillance set up

A *Safety Review Team for ofatumumab has been* established. All safety data reported during the trial including serious adverse events, non-serious adverse events, laboratory data, infections, malignancies, will be evaluated.

9.5.2 Liver Chemistry Abnormalities

Liver chemistry threshold stopping criteria have been designed to assure patient safety. The liver event case report forms should be completed if protocol-specified liver chemistry patient stopping criteria are met. The liver imaging and/or liver biopsy case report forms should be completed if these tests are performed.

When patients meet one or more of the following liver chemistry threshold criteria, ofatumumab/placebo must be permanently withdrawn, additional testing performed, and the patient monitored until liver chemistries resolve, stabilize, or return to baseline (Visit 2) values. The patient must then proceed to the Follow-up Period:

- ALT ≥ 3xULN and bilirubin ≥1.5xULN (>35% direct).

- ALT ≥ 5xULN.

- ALT ≥ 3xULN if associated with the appearance or worsening of hepatitis symptoms or rash.

Patients with ALT ≥3xULN **and** <5xULN **and** bilirubin <1.5xULN, who do not exhibit hepatitis symptoms or rash, can continue ofatumumab/placebo and be monitored weekly for up to 4 weeks. At any point, if these patients meet the liver chemistry threshold stopping criteria (outlined above) or are unable to return for weekly liver chemistries, ofatumumab must be permanently withdrawn, additional testing performed, and the patient continue safety follow-up until liver chemistries resolve, stabilize, or return to baseline values (Visit 2). The patient must not receive additional ofatumumab courses.

Patients with ALT ≥3xULN and/or bilirubin ≥1.5xULN (>35% direct bilirubin; bilirubin fractionation required) must be immediately and permanently withdrawn from ofatumumab / placebo. Every attempt must be made to have the patient return to clinic (within 24 hours) for repeat liver chemistries and additional testing, and monitored closely (with specialist or hepatology consultation recommended). This event must be reported to sponsor within 24 hours of learning of its occurrence. Patients must be monitored twice weekly until liver chemistries (ALT, AST, alkaline phosphatase, bilirubin) resolve, stabilize or return to within baseline (Visit 2) values. Upon completion of the safety follow-up, the patient must then proceed to the Follow-up Period.

Patients with ALT ≥5xULN or ALT ≥3xULN with hepatitis or rash or if increase persists ≥4 weeks

must be immediately withdrawn from ofatumumab/placebo. Every attempt must be made to have the patient return to clinic within 24-72 hours for repeat liver chemistries and additional testing, and monitored weekly until liver chemistries (ALT, AST, alkaline phosphatase, bilirubin) resolve, stabilize or return to within baseline values. This event must be reported to sponsor within 24 hours of learning of its occurrence.

Patients with ALT ≥3xULN **and** <5xULN **and** bilirubin <1.5xULN can continue to receive ofatumumab/placebo, with every attempt made to have the patient return for repeat liver chemistries within one week, and be monitored weekly for up to 4 weeks. However, patients unable to be monitored for 4 weeks must be withdrawn from ofatumumab/placebo and monitored weekly until liver chemistries resolve, stabilize or return to within baseline values. These patients should not receive additional treatment courses. This event must be reported to sponsor within 24 hours of learning of its occurrence.

If, after 4 weeks of monitoring, ALT <3xULN and bilirubin <1.5xULN, patients should be monitored twice monthly until liver chemistries normalize or return to within baseline values. Note that ofatumumab/placebo must be withdrawn if bilirubin >1.5xULN or there are signs/symptoms of hepatitis or hypersensitivity or elevations in ALT ≥3xULN and <5xULN persist for more than 4 weeks.

In all the above situations, every attempt must be made to obtain the following:

- Viral hepatitis serology including:

- Hepatitis A IgM antibody;
- Hepatitis B surface antigen and Hepatitis B Core Antibody (IgM);
- Hepatitis C RNA;
- Cytomegalovirus IgM antibody;
- Epstein-Barr viral capsid antigen IgM antibody (or if unavailable, obtain heterophile antibody or monospot testing);
- Hepatitis E IgM antibody (if patient resides outside the USA or Canada, or has traveled outside USA or Canada in past 3 months).

- Creatinine Phosphokinase (CPK) and lactate dehydrogenase (LDH).

- Fractionate bilirubin, if bilirubin > 1.5xULN.

- Record the use of concomitant medication, paracetamol (acetaminophen), herbal remedies, other over the counter medications, putative hepatotoxins, or alcohol on the concomitant medication

report form.

The following are required for patients with ALT >3xULN and bilirubin >1.5xULN but are optional for other abnormal liver chemistries:

- Anti-nuclear antibody, anti-smooth muscle antibody, and Type 1 anti-liver kidney microsomal antibodies.

- Liver imaging (ultrasound, magnetic resonance, or computerized tomography) to evaluate liver disease.

9.5.3 Infections and malignancies

As part of the ongoing program to evaluate the benefit/risk of ofatumumab, this trial will include enhanced safety monitoring during the trial with regard to serious infections, malignancy, and deaths. While serious infections and malignancies have been observed in RA patients, a reasonable possibility of a causal association between ofatumumab and these events has not been established. Cases of the opportunistic viral infection progressive multifocal leukoencephalopathy (PML) have been reported in patients with hematologic malignancies systemic lupus erythematosus and rheumatoid arthritis treated with another anti-CD20 antibody, rituximab. Additionally, cases of PML have occurred in patients who have not received rituximab. Most reports have been in patients with a compromised immune system, either due to medical conditions (lymphoma or blood cancers, HIV infection and congenital immunodeficiency syndromes and systemic lupus erythematosus) or medical treatments (cancer chemotherapy and immunosuppressive medications in organ transplant recipients)[*]. In the Hx-CD20-406 trial one case of PML was observed in a CLL patient who had received ofatumumab (1x300mg & 10x2000mg) previously treated with fludarabine and alemtuzumab with low T-lymphocyte CD4 counts.

In order to accommodate potential developments of PML, neurological examinations and assessments of JCV PCR will be performed throughout the duration of the trial (see Section 2). Once identified, signs and symptoms consistent with a diagnosis of PML will be reported promptly to sponsor. Signs and symptoms of PML include visual disturbances, ocular movements, ataxia, and changes in mental status such as disorientation or confusion. These symptoms are not an exhaustive list and the investigator should exercise judgment in deciding to report signs and symptoms to sponsor promptly. Refer to the Study Procedures Manual for further information and detailed guidance for completing and transmitting these and other SAE reports for patients who experience a serious infection, malignancy, death, or sign or symptom of PML. If a patient develops neurological signs or symptoms consistent with PML, study drug is to be discontinued and the patient referred to a neurologist for evaluation. At a minimum, blood JCV PCR and/or brain MRI will be performed and if either is positive perform Cerebrospinal Fluid (CSF) JCV PCR. If blood JCV PCR and brain MRI are negative, the investigator will contact sponsor for appropriate action to be taken with study

[*] Ref FDA Alert 12/2006

drug. If blood JCV PCR and/or brain MRI are positive, the patient should proceed to the Follow-Up Period. All such patients will be followed until resolution. Any patient with a diagnosis of PML will be withdrawn from ofatumumab / placebo.

The investigator will do the following when reporting a serious infection, malignancy, death, or sign/symptom consistent with PML.

- Promptly report the event, as with any other SAE, as per Section 9.3.2 of this protocol.
- Provide key source documentation for sponsor to assist with the safety evaluation process.

Examples of key source documents include but are not limited to: hospitalization records, discharge summaries, laboratory evaluations, biopsy results, culture/sensitivity results, death certificates, and autopsy reports.

If the patient has not otherwise been withdrawn from the study, then the investigator should contact sponsor to discuss the appropriate course of action regarding study continuation.

9.5.4 Critical Adverse Event

A Critical Adverse Event (CAE) is defined as follows:

- Occurrence of a treatment related Adverse Event (AE) graded by the investigator as severe at the day of infusion and preventing the infusion to be resumed.
- Second occurrence of a treatment related bronchospasm graded by the investigator as severe during one infusion.
- If the severity of an AE becomes severe for the third time during one infusion.
- All infections reported as serious
- The occurrence of treatment related neurological events consistent with PML.
- Any malignancy.

Treatment related is defined as an event where either the investigator or the sponsor judges the AE to be related to trial drug.

9.5.5 Stopping Rules

If the number of patients with a Critical Adverse Event (CAE) reaches the limits specified in Table 9, further patient enrollment will be paused pending advice from the Safety Review Team (see Section 9.5.1, and Section 18.2). In Table 9, N is the number of patients enrolled and N_{CAE} is the number of patients with a CAE. The error rate states the probability of pausing with an acceptable risk of a CAE of 5% during the study. The detection rate states the probability of pausing with an unacceptable risk of 10%.

Table 9 : Limits of CAE with error and detection rates

N_{CAE}	N	Probability of pausing with acceptable CAE risk of 5% (Error rate).	Probability of pausing with unacceptable CAE risk of 10% (Detection rate)
4	30	6.1%	35%
7	60	7.4%	49%
10	100	8.6%	64%
14	150	9.1%	75%
18	200	9.4%	82%

For example, if 4 patients or more among the first 30 enrolled experience a CAE during the study, patient enrollment should be paused. Enrollment can only be continued following the decision from the Safety Review Team. In this manner, patients are divided into enrollment groups as indicated in N in Table 9, and N_{CAE}'s are the lower limit of discontinuation for the first group, the first and second combined, the three first groups combined, and so forth.

The Safety Review Team must evaluate the nature and the character of the CAE, the degree to which the adverse events are to be expected and the importance with regard to patient risk. The Safety Review Team shall carefully evaluate any CAE and recommend whether changes to dosing should be performed or other modifications to the planned conduct of the study. Thus it is possible to have reached the per protocol pre-defined maximum number of CAEs without having to stop the trial in case the Safety Review Team judges that the events do not constitute a safety signal that warrants modification to the planned conduct of the study.

10 Statistics

This section presents the principal features of the statistical analysis of this trial. Further details will be given in a separate Reporting and Analysis Plan (RAP), which will be finalized before breaking the blind.

The analyses and presentations will be performed for the Intent-to-treat dataset unless specified otherwise.

The significance level is set to 5%. All confidence intervals will be two-sided.

All summary statistics of continuous variables will include: n, mean, median, standard deviation, minimum and maximum. All summaries presenting frequencies and incidences will include n, % and N, where N is the total number of patients with recorded values in the corresponding group.

All data listings will include all randomized patients.

In the event the sponsor decides to suspend or discontinue the intravenous route of administration development program for RA and this trial is terminated early such that there are insufficient subjects to achieve 90% power, no statistical testing will be performed and only limited efficacy data will be summarized. Safety data will be reported as planned. Further details will be included in the RAP.

10.1 Analysis Populations

10.1.1 Intent-to-treat (ITT)

The Intent-to-treat (ITT) dataset, also known as the Full Analysis Set, comprises all randomized patients who have been exposed to study drug irrespective of their compliance to the planned course of treatment. This is the primary analysis dataset and will be used for evaluation of all endpoints.

10.1.2 Per Protocol (PP)

ACR20, the primary endpoint, and the secondary endpoints ACR50 and ACR70 will be analyzed for the Per Protocol dataset in addition to the ITT analyses. The PP population will not be analysed if this population comprises more than 90% or less than 50% of the ITT population. A patient will be excluded from the PP dataset on the basis of:

- Major violation of entry criteria (will be defined explicitly in the RAP).
- Use of prohibited concomitant therapy (see Section 7.4.2).
- Unblinding of treatment allocation.
- Any other major protocol violation assessed to severely affect the primary efficacy assessment for that patient.

10.1.3 Safety

The safety dataset is identical to the ITT dataset. However unlike in the ITT population, patients are analyzed according to their actual treatment in case this differs from their randomized treatment. All safety analyses will be based on the safety dataset.

All decisions regarding definition of analysis datasets will be made prior to unblinding.

10.2 Statistical Analysis of Primary Endpoint

10.2.1 ACR20

The primary endpoint is ACR20 at week 24. The ACR score is based on improvement from baseline in tender (TJC) and swollen joint counts (SJC). A patient has achieved ACR20 if the patient experiences ≥20% improvement from baseline in:

- Tender Joint Count (TJC) and Swollen Joint Count (SJC)
- **and** ≥20% improvement from baseline in 3 out of 5 of the following assessment;
- patient pain assessment on a 100 mm VAS scale
- patient global assessment on a 100 mm VAS scale
- physician global assessment on a 100 mm VAS scale
- patient self-assessed disability (HAQ-DI)
- C-reactive protein (CRP) determined in mg/L

The Cochran Mantel Haenszel (CMH) test will be used for comparing the ACR20 responder rates of ofatumumab+MTX versus MTX stratifying by rheumatoid factor seropositivity /-negativity, and region. Ideally, the analysis would be stratified by center (site) but since many centers will be used, some will recruit only few patients, and only strata with at least one patient on each treatment contributes to the CMH test statistic. Therefore, the analysis will be stratified by region instead. Further details about the pooling strategy will be defined in the RAP prior to unblinding. The Mantel Haenszel estimate of the ofatumumab+MTX versus MTX ratio of response rates with associated 95% confidence interval and p-value will be computed.

An exploratory logistic regression analysis investigating the effect of possible covariates will be conducted (see RAP for further details).

10.2.2 Handling of missing values and use of rescue medication

Patients that failed to complete the Double-blind phase of the study or who required rescue therapy (according to Section 7.4.1) will be considered as non-responders.

If CRP is missing at week 24 for an evaluation of the ACR response (ACR20, ACR50, ACR70 & ACRn) at 24 weeks, erythrocyte sedimentation rate (ESR) will be used instead of CRP for that ACR response assessment. For any other missing individual components or in case both the CRP and ESR measurements are missing, the last non-missing observation prior to week 24 will be carried forward (LOCF) for each component.

A supportive analysis will be conducted where drop outs prior to week 24 with withdrawal reasons that are not related to treatment (e.g. not for lack of efficacy) will use the last non-missing value for each component.

For patients who receive an intra-articular injection of corticosteroid in a single joint, the joint will count as tender and swollen in the ACR and DAS28 assessments for the 12 weeks following the time of injection.

10.3 Statistical Analysis of Secondary Endpoints

The secondary endpoints are considered to be supportive of the primary endpoint and therefore no statistical adjustments for multiplicity are planned.

10.3.1 ACR50 and ACR70

ACR50 and ACR70 are defined similarly to ACR20, requiring 50% and 70% improvements respectively. ACR50 and ACR70 at week 24 will be analyzed in the same way as ACR20 at week 24 (See Section 10.2.1 and Section 10.2.2).

10.3.2 ACRn

ACRn is the largest integer n, for which a patient meets the ACR criteria requiring an improvement of n%. ACRn at week 24 will be rank transformed because of known problems with occasional outlying values. The ACRn ranks will be analyzed using Analysis of Covariance (ANCOVA) adjusting for covariates that include rheumatoid factor seropositivity /-negativity and region. The significance of the treatment effect will be evaluated based on the p-value for the F-test of no treatment difference in this model.

Each of the individual components of the ACR will be analyzed at week 24 using ANCOVA adjusting for covariates that include baseline value, rheumatoid factor seropositivity and region. The model adjusted mean at Week 24 will be presented for each treatment group. In addition, the treatment difference between ofatumumab and placebo and associated 95% confidence interval and p-value will be presented.

In assumptions of normality do not hold for the analyses of the individual components, transformation of data or non-parametric approaches will be considered.

10.3.3 DAS28

Disease activity score (DAS28) will be calculated based on the following parameters:

Tender joint count (TJC), swollen joint count (SJC), C-reactive protein (CRP in mg/L) and patient global assessment (PGA in mm) using the formula:

$$\mathrm{DAS28} = 0.56\sqrt{\mathrm{TJC28}} + 0.28\sqrt{\mathrm{SJC28}} + 0.36\ln(\mathrm{CRP}+1) + 0.014\times\mathrm{PGA} + 0.96$$

The joint counts will be based on 28 joints for DAS28. The 28 joints constitute the following and is to be assessed for both left and right side: Shoulder, elbow, wrist, metacarpophalangeal (first (thumb), second, third, fourth, fifth), proximal interphalangeal (thumb (interphalangeal), index, middle, ring, little) and knee.

The DAS28 score will also be calculated using the Erythrocyte Sedimentation Rate (ESR in mm/hr):

$$DAS28 = 0.56\sqrt{TJC28} + 0.28\sqrt{SJC28} + 0.7\ln(ESR) + 0.014PGA$$

Both DAS28 scores will be subject to the same statistical analysis described below. But the main conclusion will be based on the DAS28 score calculated using CRP. Note that at the sites the DAS28 score based on ESR will be used.

DAS28 will be analyzed at week 24 using ANCOVA adjusting for covariates that include baseline DAS28, rheumatoid factor seropositivity and region. The model adjusted mean at Week 24 will be presented for each treatment group. In addition, the treatment difference between ofatumumab and placebo and associated 95% confidence interval and p-value will be presented.

10.3.4 EULAR

The DAS28 is a clinical index of RA disease activity that combines information from swollen joints, tender joints, the acute phase response and general health. The DAS28-based European League Against Rheumatism (EULAR) response criteria were developed to measure individual response in clinical trials. The EULAR response criteria classify individual patients as non-, moderate, or good responders, dependent on the extent of change and the level of disease activity reached. The EUL AR response criteria are based on the DAS28 score as follows:

Table 10: EULAR Response Conversion Table

DAS28	Improvement in DAS28 from baseline (Visit 2)		
	>1.2	>0.6 and ≤1.2	≤0.6
≤3.2	Good	Moderate	None
> 3.2 and ≤ 5.1	Moderate	Moderate	None
>5.1	Moderate	None	None

The EULAR response will be calculated based on the DAS28 score based on CRP and the DAS28 score based on ESR. Both EULAR scores will be subject to statistical analysis as described below but the statistical analysis on EULAR score based on CRP will be considered as the main analysis.

Patients with a moderate or good EULAR response as opposed to no response at week 24 will be analyzed as the binary ACR20/50/70 outcomes (See Section 10.2.1).

Patients that withdraw prior to 24 weeks or who require rescue therapy (according to Section 7.4.1) should be considered as non-responders.

10.3.5 Clinical Remission

Patients achieving clinical remission are defined as those with a low disease activity at week 24. This is defined as patients achieving a DAS28 score < 2.6 at week 24 (40). The DAS28 score used will be that derived using CRP.

Clinical remission will be analysed using the Cochran Mantel Haenszel (CMH) test as described in Section 10.2.1.

10.3.6 HAQ-DI Responders

HAQ-DI responders are defined as those patients achieving a change from baseline Health Assessment Questionnaire Disability Index (HAQ-DI) score at week 24 of ≥ 0.22 (41).

HAQ-DI response will be analysed using the Cochran Mantel Haenszel (CMH) test as described in Section 10.2.1.

10.4 Safety

10.4.1 Adverse Events

Adverse events will be categorized using the preferred terms and system organ class assigned by the Medical Dictionary for Regulatory Activities (MedDRA). The proportion of patients reporting each adverse event will be tabulated for each treatment group. The following summaries will be produced:

- All AEs

- AEs related to study drug
- AEs leading to withdrawal
- Serious AEs
- AEs occurring during the infusion
- Serious/severe infections
- Malignancies

10.4.2 Vital Signs

Summary statistics for the vital signs at each visit and also the change from baseline will be tabulated by treatment group

10.4.3 Laboratory Safety Data

Summary statistics for the laboratory measurements at each visit and also the change from baseline will be tabulated by treatment group. Additionally the number and percent of patients with values of clinical concern will be summarized by treatment group. Clinical concern criteria will be specified in the analysis plan

10.4.4 ECG

ECG assessments will be summarized by treatment and week.

10.4.5 Immunoglobulin levels

IgA, IgG and IgM levels will be summarized by treatment and week. Incidences of values below lower normal reference limit will be reported by week.

10.4.6 Flow cytometry

$CD19^+$, $CD3^+$, $CD4^+$, and $CD8^+$ profiles will be presented graphically. Time to re-population (return to normal or baseline level) will be summarized.

10.4.7 Host Immune Response

Changes from baseline in HAHA titer will be summarized by descriptive statistics.

10.5 Patient reported outcomes

10.5.1 HAQ-DI

HAQ-DI disability scores and pain scores will be analyzed as the DAS scores.

10.5.2 SF-36

SF-36 domain scores will be analyzed as the DAS scores.

10.5.3 FACIT-F

FACIT-F scores will be analyzed as the DAS scores.

10.6 Pharmacokinetics

Individual curves of serum concentration of ofatumumab will be presented for all patients including all available data. Further non-compartmental or compartmental PK modeling may be done. The analysis will be based on the ITT dataset.

10.7 Biomarkers / Transcriptomics

Biomarkers will be summarized by descriptive statistics. Exploratory analyses will be conducted to examine the relationship to clinical response and need of re-treatment.

10.8 Handling of Missing Data, data recorded after possible treatment with rescue therapy or Outliers

Unless described otherwise, missing values in the primary and secondary endpoints will be imputed using last observation carried forward (LOCF). In order to address the issue of rescue therapy prior to week 24 for secondary endpoints (efficacy related including patient reported outcome) in general two analyses will be performed:

1. Excluding data recorded after administration of rescue therapy
2. Including all recorded data regardless of the use of rescue therapy

The former one is considered the primary one, in line with the main analysis for the primary endpoint.

No imputation of missing data is planned for safety endpoints and pharmacokinetic endpoints.

10.9 Subgroups and Center Effects

The handling of strata, including centers is described in the analysis sections for each endpoint.

Countries and – by implication - centers will be combined in order to account both for regional differences and differences due to rheumatoid factor seropositivity in the stratification. Stratification for rheumatoid factor seropositivity together with stratification for center or country is not feasible since many strata would be too small or empty. The regions will be defined and described in the statistical analysis plan prior to generation of the randomization schedule.

All primary and key secondary endpoints will be presented separately by rheumatoid factor seropositivity.

10.10 Analysis of Open-Label data

Since the Open-Label part is uncontrolled no formal statistical tests will be used. Descriptive statistics will include

- ACR responses and DAS scores over time
- Duration of ACR20 response
- Use of rescue medication

10.11 Determination of Sample Size

The planned sample size is based on the power of a test of the null hypothesis that the proportion of ACR20 responders is the same with ofatumumab+MTX as with MTX alone under the alternative hypothesis that the ACR20 response rate is 45% with ofatumumab+MTX group and 25% with MTX. In a two-arm trial (1:1), 2x118=236 patients are needed in total in order to achieve 90% power with a significance level of 5%. This is based on a Chi-square test comparing two binomial proportions.

11 Ethics

11.1 Independent Ethics Committee (IEC) or Institutional Review Board (IRB)

The protocol, any amendments, the consent form, and the patient information must be approved by the health authorities according to local regulations and by appropriately constituted IECs or IRBs, before study initiation.

11.2 Patient Information and Informed Consent

The principal Investigator or his/her designee must obtain the written Informed Consent from each patient or the patient's acceptable authorized representative before any study related procedures are performed. In this context, wash-out of RA medications is also considered a study related procedure. Each patient must receive full patient information before giving consent. The patient information must contain full and adequate verbal and written information regarding the objective and procedures of the study and the possible risks involved.

Before signing the Informed Consent the patient must be given sufficient time to consider their possible participation. Furthermore, each patient must be informed about their right to withdraw from the study at any time.

Each patient must sign and date the Informed Consent form; the patient receives a copy of the signed form and the original is retained in the Investigator Site File. The Informed Consent forms must be signed and dated both by the patient and by the investigator or designee providing the information to the patient.

11.3 Ethical and Risk/Benefit Considerations

The following ethical considerations have been considered:

Is this trial necessary?

Rheumatoid arthritis (RA) is a systemic inflammatory disease which affects 0.8-1.0% of all populations. For unknown reasons the immune system attacks the synovium (tissue lining the joint capsule), causing local inflammation. This inflammatory response results in the destruction of ligaments, cartilage and bone within the joint but also tendons and muscles that support the joint are affected. Unless the inflammatory processes are halted or controlled, the disease leads to substantial disability.

Current treatment approaches include non-steroidal anti-inflammatory drugs (NSAID) for the relief of inflammation and pain, and disease-modifying antirheumatic drugs (DMARDs) for slowing the progression of the disease. When systemic RA treatment has failed, intra-articular injections of corticosteroids can temporarily ease the symptoms of RA. Advances in treatment include the

introduction of biologic DMARDs, offering a more effective and targeted therapy. None of the abovementioned treatments are curative and they are also associated with toxicity.

The overall objective in the treatment of RA is to maintain the quality of life of the patient by means of pain relief, reduction of inflammation and prevention of joint destruction and -deformities.

Therefore there is still an unmet need for new effective long-term treatments of active chronic RA.

What is the risk/benefit for the participating patients?

A phase I/II trial showed that ofatumumab has an acceptable safety profile and is well-tolerated. Most side effects occurred on days of infusion and comprised fatigue, rigors, pyrexia, dyspnoea, pharyngeal pain, rash, pruritus, urticaria, headache, flushing, hypotension and increased sweating. To prevent or relieve possible side effects the patient will receive pre-medication.

Ofatumumab has a theoretical advantage compared to other biologic DMARDs in being an antibody of fully human origin and therefore may have a lower potential to cause allergic reactions compared to antibodies of animal origin.

It is anticipated that the number of B-cells will deplete after treatment with ofatumumab. The number of B-cells will be monitored during and after the trial until the level of B-cells has normalized.

A phase I/II trial indicates clinical efficacy of ofatumumab in RA patients which the patients participating in this trial will benefit from. Patients originally randomized to receive placebo will be offered ofatumumab in the open-label extension of the trial.

In relation to the assessment of the disease, a chest X-ray will be taken at the first visit. The dose of radiation from a chest X-ray is very small (0.25mRad). Knowing that all people receive approximately 100 mRad (400 times that of a chest x-ray) annually from cosmic rays and trace radioactive minerals in surroundings, the increased risk of cancer by one chest X-ray is considered insignificant.

Is it ethical to perform a placebo-controlled study?

The use of placebo is considered reasonable for the individual patient, based on the following:

- Patients enrolled in this trial may have no access to acceptable, proven therapies
- All patients will receive background treatment of RA throughout the trial.
- At request of either the patient or the investigator the patient may be withdrawn from the trial at any time.
- Breakthrough pain management and intra-articular corticosteroid injections are allowed.

Considering the above and the fact that patients have failed other RA treatments the risk of not achieving a clinically relevant response in the placebo-controlled period of the trial is considered acceptable.

12 Monitoring and Quality Assurance (QA)

12.1 Compliance with Good Clinical Practice

This Trial Protocol is designed to comply with the Guideline produced by the International Conference on Harmonization (ICH) on the topic Good Clinical Practice (GCP) and published by the European Medicines Agency “Note for Guidance on Good Clinical Practice” (CPMP/ICH/135/95) (Approval 17 July 1996) as well as other relevant guidelines issued by ICH, primarily the efficacy guidelines.

12.2 Monitoring

Monitoring visits to the trial site will be made periodically during the trial, to ensure that all aspects of the protocol are followed. Source documents will be reviewed for verification of agreement with data on the eCRF.

To ensure compliance with GCP and all applicable regulatory requirements, GSK may also conduct a quality assurance audit of the site records, and the regulatory agencies may conduct a regulatory inspection at any time during or after completion of the study. In the event of an audit or inspection, the investigator (and institution) must agree to grant the auditor(s) and inspector(s) direct access to all relevant documents and to allocate their time and the time of their staff to discuss any findings/relevant issues

It is important that the Investigator and his/her relevant personnel are available during the monitoring visits and possible audits and that sufficient time is devoted to the process.

12.3 Source Data Verification

Source Documents are original documents, data and records (e.g. hospital records, clinical and office charts, laboratory notes, memoranda, x-rays, patient records kept at the pharmacy, recorded data from automated instruments etc.). Source Data is considered all information in original records and certified copies of clinical findings, observations, or other activities in the study. Source Data are contained in Source Documents (original records or certified copies).

The location of source documents will be registered on a form specifying where source data can be located, e.g. medical records, eCRF, lab reports, etc.

The following items must as a minimum be available for Source Data Verification (SDV) in source documents other than the eCRF:

- Date of conducting Informed Consent
- Date of birth and sex

- Statement that the patient is participating in clinical trial GEN411/OFA110634 with the study drug ofatumumab
- Data for evaluation of eligibility criteria
- Relevant medical history and diagnosis
- Patient ID Number
- Administration of trial drug
- All study visit dates
- Adverse Events or absence of Adverse Events
- Concomitant Medication including changes
- Date and reason for withdrawal from trial and / or trial treatment

12.4 Study and Site Closure

Upon completion or termination of the study, the GSK monitor will conduct site closure activities with the investigator or site staff (as appropriate), in accordance with applicable regulations, GCP, and GSK Standard Operating Procedures.

GSK reserves the right to temporarily suspend or terminate the study at any time for reasons including (but not limited to) safety issues, ethical issues, or severe non-compliance. If GSK determines that such action is required, GSK will discuss the reasons for taking such action with the investigator or head of the medical institution (where applicable). When feasible, GSK will provide advance notice to the investigator or head of the medical institution of the impending action.

If a study is suspended or terminated for **safety reasons**, GSK will promptly inform all investigators, heads of the medical institutions (where applicable),and/or institutions conducting the study. GSK will also promptly inform the relevant regulatory authorities of the suspension/termination along with the reasons for such action. Where required by applicable regulations, the investigator or head of the medical institution must inform the IRB/IEC promptly and provide the reason(s) for the suspension/termination.

13 Data Handling and Record Keeping

13.1 Data Management

Data Management will identify and implement the most effective data acquisition and management strategy for this clinical trial protocol and deliver datasets which support the protocol objectives. For this study patient data will be entered into electronic Case Report Forms (eCRFs), transmitted electronically to sponsor and combined with data provided from other sources (e.g. electronic diary data, laboratory data, and VAS scale gradings on paper) in a validated data system.

Trained study personnel will be responsible for entering data on the observations, measurements and assessments specified in the protocol into the eCRF according to procedures described in the study procedures manual.

Clinical data management will be performed in accordance with applicable sponsor standards and data cleaning procedures with the objective of removing errors and inconsistencies in the data which would otherwise impact on the analysis and reporting objectives, or the credibility of the Clinical Study Report. Adverse events and concomitant medications terms will be coded using MedDRA and GSKDrug, a validated medication dictionary provided by GSK. The database containing the eCRFs will be retained by sponsor, while the investigator will retain a copy. In all cases, patient initials will not be collected or transmitted to sponsor

The completed eCRFs are the sole property of the sponsor and should not be made available in any form to third parties, except for authorized audits performed for sponsor and for representatives of appropriate Health/Regulatory Authorities, without written permission from sponsor.

13.2 Archiving of Trial Documents at Site

Following closure of the study, the investigator or head of the medical institution (where applicable) must maintain all site study records (except for those required by local regulations to be maintained elsewhere) in a safe and secure location. The records must be easily accessible when needed (e.g. for sponsor audit or regulatory inspection) and must be available for review in conjunction with assessment of the facility, supporting systems, and relevant site staff.

Where permitted by local laws/regulations or institutional policy, some or all of the records may be maintained in a format other than hard copy (e.g. microfiche, scanned, electronic); however, caution must be exercised before such action is taken. The investigator must ensure that all reproductions are legible and are a true and accurate copy of the original. In addition, they must meet accessibility and retrieval standards, including regeneration of a hard copy, if required. The investigator must also ensure that an acceptable back-up of the reproductions exists and there is an acceptable quality control procedure in place for creating the reproductions.

The sponsor will inform the investigator of the time period for retaining the site records in order to

comply with all applicable regulatory requirements. The minimum retention time will meet the strictest standard applicable to a particular site, as dictated by local laws/regulations, sponsor standard operating procedures, and/or institutional requirements.

The investigator must notify sponsor of any changes in the archival arrangements, including, but not limited to archival of records at an off-site facility or transfer of ownership of the records in the vent that the investigator is no longer associated with the site.

14 Reporting and Communication of Results

14.1 Use of Information

All unpublished information relating to this trial and/or to the trial drug is considered confidential by the sponsor and shall remain the sole property of the sponsor.

The investigator must accept that sponsor may use the information from this clinical trial in connection with the development of the product, and therefore may disclose it as required to other investigators, to government licensing authorities, to regulatory agencies of other government, the stock exchange market, and commercial partners.

14.2 Provision of Study Results to Investigators, Posting to the Clinical Trials Register and Publication

Where required by applicable regulatory requirements, an investigator signatory will be identified for the approval of the clinical study report. The investigator will be provided reasonable access to statistical tables, figures, and relevant reports and will have the opportunity to review the complete study results at a GSK site or other mutually-agreeable location.

GSK will also provide the investigator with the full summary of the study results. The investigator is encouraged to share the summary results with the study subjects, as appropriate.

GSK will provide the investigator with the randomization codes for their site only after completion of the full statistical analysis.

The results summary will be posted to the Clinical Study Register at the time of the first regulatory approval or within 12 months of any decision to terminate development. In addition, a manuscript will be submitted to a peer-reviewed journal for publication within 12 months of the first approval or within 12 months of any decision to terminate development. When manuscript publication in a peer-reviewed journal is not feasible, further study information will be posted to the GSK Clinical Study Register to supplement the results summary.

15 Insurance/Liability/Indemnity

The patients in the present study are covered by the product and general liability insurance of GSK in the event of study related injury or death, in accordance with applicable law and with the CHMP Note for Guidance on Good Clinical Practices (CPMP/ICH/135/95) of 17 July 1996.

16 Changes to the Final Protocol

Any variation in procedure from that specified in the Final Trial Protocol may lead to the results of the trial being questioned and in some cases rejected. Any proposed protocol change must therefore be discussed with and approved by sponsor and submitted for the Ethics Committee and Health Authority approval or notification. Any protocol change should be documented in a Protocol Amendment.

17 Premature Termination of the Trial

If sponsor or International Coordinating Investigator discovers conditions arising during the trial, which indicate that the clinical investigation should be halted, the trial can be terminated after appropriate consultation between sponsor and International Coordinating Investigator. The Regulatory Authorities and Independent Ethics Committees/Institutional Review Board will be notified in writing. The reason will be stated.

Conditions that may warrant termination of the trial include, but are not limited to the following:

- The discovery of an unexpected and significant or unacceptable risk to the patients enrolled in the trial.
- The discovery of lack of efficacy.
- Failure of the Investigators to enter patients at an acceptable rate in the trial as a whole.
- A decision on the part of sponsor to suspend or discontinue development of the drug or formulation of drug in this indication.

In the event the trial is prematurely terminated, all subjects in Double-blind or Open-label Periods will enter the Follow-Up Period at the next scheduled study visit (see Section 6.5 for details on Follow–up activities)

17.1 Premature Termination of a Trial Site

Furthermore, sponsor can decide to prematurely terminate single sites. Conditions that may warrant termination include, but are not limited to the following:

Insufficient adherence to protocol requirements.
Failure to enter patients at an acceptable rate.

18 List of abbreviations and definitions of terms

18.1 List of abbreviations

ACR	American Collegue of Rheumatology
ADCC	Antibody Dependent Cell-mediated Cytotoxicity
ADR	Adverse Drug Reaction
AE	Adverse Event
ALP	Alkaline Phosphatase
ALT/ALAT	Alanine Amino Transferase
ALT/SGPT	Alanine Amino Transferase /Serum glutamate pyruvate transaminase
ANCOVA	Analysis of Covariance
AST/SGOT	Aspartate Aminotransferase/Glutamate-oxaloacetate Transaminase
AUC	Area Under Curve
BLyS	B-Lymphocyte Stimulatory protein
BUN	Blood Urea Nitrogen
CAE	Critical Adverse Events
CCP	Cyclic Citrullinated Peptide
CDC	Complement Dependent Cytotoxicity
CHMP	Committee for Medicinal Products for Human Use
CLL	Chronic Lymphocytic Leukemia
CMH	Cochran Mantel Haenszel test
CPK	Creatinine Phosphokinase
CPMP	Committee for Propriety Medicinal Products
CRA	Clinical Research Associate
CRP	C - Reactive Protein
CT	Computerized Tomography
CTC	Common Terminology Criteria
CTCAE	Common Terminology Criteria Adverse Events
CTL4A-Ig	Cytotoxic T-lymphocyte-associated antigen 4
DAS28	Disease Activity Score (based on 28 joints)

DMARD	Disease-modifying antirheumatic drugs
DNA	DeoxyriboNucleic Acid
ECG	Electrocardiogram
eCRF	Electronic Case Report Form
EDTA	EthyleneDiamine Tetraacetic Acid
ESR	Erythrocyte Sedimentation Rate
EudraCT	European Clinical Trials Database
EULAR	European League Against Rheumatism
FACIT	Functional Assessment of Chronic Illness Therapy
FACS	Flow-Activated Cell Sorter
FDA	Food and Drug Administration
FL	Follicular lymphoma
FU	Follow up
GCP	Good Clinical Practice
GGT	Gamma Glutaryl-Transferase
GSK	GlaxoSmithKline
HACA	Human Anti-Chimeric Antibodies
HAHA	Human Anti Human Antibodies
HAQ-DI	Health Assessment Questionnaire Disability Index
HB	Hepatitis B
HbsAg	Hepatitis B Surface Antigen
HCG	Human Chorionic Gonadotrophine
HCQ	Hydroxychloroquine
HIV	Human Immunodeficiency Virus
i.a.	Intra-articular
i.m.	Intra-muscular
i.v.	Intravenous
ICH	International Conference on Harmonization
IEC	Independent Ethics Committee
IgA	Immunoglobulin A
IgG	Immunoglobulin G
IgM	Immunoglobulin M

IL	Interleukin
IMP	Investigational Medicinal Product
IRB	Institutional Review Board
ITT	Intention To Treat
IVRS	Interactive Voice Response System
JCV	JC Virus
LDH	Lactate Dehydrogenase
LOCF	Last Observation Carried Forward
mAb	Monoclonal Antibody
MedDRA	Medical Dictionary for Regulatory Activities
mRNA	Messenger Ribonucleic Acid
MTD	Maximum Tolerated Dose
MTX	Methotrexate
NCI	National Cancer Institute
NSAID	Non-Steroidal Anti-Inflammatory Drugs
PCR	Polymerase Chain Reaction
PGA	Patient Global Assessment
PGx	Pharmacogenetics
PK	Pharmacokinetics
PML	Progressive Multifocal Leukoencephalopathy
PP	Per Protocol
PR	Partial Remission
QA	Quality Assurance
QC	Quality Control
RA	Rheumatoid Arthritis
RBC	Red blood cell
RF	Rheumatoid Factor
SAE	Serious Adverse Event
SAP	Statistical Analysis Plan
SCID	Severe Combined Immunodeficiency
SDV	Source Data Verification
SF	Short form

SJC	Swollen Joint Count
SNP	Single Nucleotide Polymorphisms
SSZ	Sulphasalazine
SUSAR	Suspected Unexpected Serious Adverse Reaction
TB	Tuberculosis
TGF-α	Transforming Growth Factor Alpha
TJC	Tender Joint Count
TNF-α	Tumor Necrosis Factor Alpha
ULN	Upper Limit of Normal
VAS	Visual analogue scale
WBC	White Blood Cell
WHO	World Health Organization

18.2 Definition of Terms

HuMax-CD20

This was the patented trademark for this IMP. It has now been designated an INN, which is ofatumumab.

International Coordinating Investigator

The International Coordinating Investigator is responsible for approval of the Clinical Trial Protocol and Report on behalf of all investigators.

Safety Review Team

A Safety Review Team for ofatumumab has been established. The Safety Review Team will include representatives from Clinical Development, Regulatory Affairs and Medical and Safety Departments

National Coordinating Investigator

One National Coordinating Investigator may be appointed for each country. The National Coordinating Investigators will be responsible for national issues relating to the study.

Principal Investigators

The Principal Investigator at site is responsible for all aspects of study conduct at his/her site. This includes ensuring that all personnel involved in the trial are fully informed of all relevant aspects of the trial, including detailed knowledge of and training in all procedures to be followed.

Sponsor

GSK is the sponsor for this trial.

19 References

(1) Scott DL, Symmons DP, Coulton BL, Popert AJ. Long-term outcome of treating rheumatoid arthritis: results after 20 years. Lancet 1987; 1(8542):1108-1111.

(2) van der Heijde DM. Joint erosions and patients with early rheumatoid arthritis. Br J Rheumatol 1995; 34 Suppl 2:74-78.

(3) Lard LR, Visser H, Speyer I, vander Horst-Bruinsma IE, Zwinderman AH, Breedveld FC et al. Early versus delayed treatment in patients with recent-onset rheumatoid arthritis: comparison of two cohorts who received different treatment strategies. Am J Med 2001; 111(6):446-451.

(4) McInnes IB. Rheumatoid arthritis. From bench to bedside. Rheum Dis Clin North Am 2001; 27(2):373-387.

(5) Petersen J, Ingemann-Hansen T, Halkjaer-Kristensen JS. Spontaneous and induced immunoglobulin secretion by synovial fluid B lymphocytes in rheumatoid arthritis. Ann Rheum Dis 1984; 43(2):140-145.

(6) Wang H, Marsters SA, Baker T, Chan B, Lee WP, Fu L et al. TACI-ligand interactions are required for T cell activation and collagen-induced arthritis in mice. Nat Immunol 2001; 2(7):632-637.

(7) Tan SM, Xu D, Roschke V, Perry JW, Arkfeld DG, Ehresmann GR et al. Local production of B lymphocyte stimulator protein and APRIL in arthritic joints of patients with inflammatory arthritis. Arthritis Rheum 2003; 48(4):982-992.

(8) Youinou P. B cell conducts the lymphocyte orchestra. J Autoimmun 2007; 28(2-3):143-151.

(9) Toubi E, Kessel A, Slobodin G, Boulman N, Pavlotzky E, Zisman D et al. Changes in macrophage function after rituximab treatment in patients with rheumatoid arthritis. Ann Rheum Dis 2007; 66(6):818-820.

(10) Edwards JC, Cambridge G, Abrahams VM. Do self-perpetuating B lymphocytes drive human autoimmune disease? Immunology 1999; 97(2):188-196.

(11) Edwards JC, Szczepanski L, Szechinski J, Filipowicz-Sosnowska A, Emery P, Close DR et al. Efficacy of B-cell-targeted therapy with rituximab in patients with rheumatoid arthritis. N Engl J Med 2004; 350(25):2572-2581.

(12) Kremer JM, Genant HK, Moreland LW, Russell AS, Emery P, Abud-Mendoza C et al. Effects of abatacept in patients with methotrexate-resistant active rheumatoid arthritis: a randomized trial. Ann Intern Med 2006; 144(12):865-876.

(13) Finckh A, Liang MH, van Herckenrode CM, de Pablo P. Long-term impact of early treatment on radiographic progression in rheumatoid arthritis: A meta-analysis. Arthritis Rheum 2006; 55(6):864-872.

(14) Felson DT, Anderson JJ, Meenan RF. The comparative efficacy and toxicity of second-line drugs in rheumatoid arthritis. Results of two metaanalyses. Arthritis Rheum 1990; 33(10):1449-1461.

(15) Morgan SL, Baggott JE, Vaughn WH, Austin JS, Veitch TA, Lee JY et al. Supplementation with folic acid during methotrexate therapy for rheumatoid arthritis. A double-blind, placebo-controlled trial. Ann Intern Med 1994; 121(11):833-841.

(16) Ortendahl M, Schettler JD, Fries JF. Factors influencing length of time taking methotrexate in rheumatoid arthritis. J Rheumatol 2000; 27(5):1139-1147.

(17) Jobanputra P, Wilson J, Douglas K, Burls A. A survey of British rheumatologists' DMARD preferences for rheumatoid arthritis. Rheumatology (Oxford) 2004; 43(2):206-210.

(18) Emery P, Fleischmann R, Filipowicz-Sosnowska A, Schechtman J, Szczepanski L, Kavanaugh A et al. The efficacy and safety of rituximab in patients with active rheumatoid arthritis despite methotrexate treatment: results of a phase IIB randomized, double-blind, placebo-controlled, dose-ranging trial. Arthritis Rheum 2006; 54(5):1390-1400.

(19) Edwards JC, Szczepanski L, Szechinski J, Filipowicz-Sosnowska A, Emery P, Close DR et al. Efficacy of B-cell-targeted therapy with rituximab in patients with rheumatoid arthritis. N Engl J Med 2004; 350(25):2572-2581.

(20) Finckh A, Ciurea A, Brulhart L, Kyburz D, Moller B, Dehler S et al. B cell depletion may be more effective than switching to an alternative anti-tumor necrosis factor agent in rheumatoid arthritis patients with inadequate response to anti-tumor necrosis factor agents. Arthritis Rheum 2007; 56(5):1417-1423.

(21) Higashida J, Wun T, Schmidt S, Naguwa SM, Tuscano JM. Safety and efficacy of rituximab in patients with rheumatoid arthritis refractory to disease modifying antirheumatic drugs and anti-tumor necrosis factor-alpha treatment. J Rheumatol 2005; 32(11):2109-2115.

(22) Westhovens R, Cole JC, Li T, Martin M, Maclean R, Lin P et al. Improved health-related quality of life for rheumatoid arthritis patients treated with abatacept who have inadequate response to anti-TNF therapy in a double-blind, placebo-controlled, multicentre randomized clinical trial. Rheumatology (Oxford) 2006; 45(10):1238-1246.

(23) Weinblatt M, Combe B, Covucci A, Aranda R, Becker JC, Keystone E. Safety of the selective costimulation modulator abatacept in rheumatoid arthritis patients receiving background biologic and nonbiologic disease-modifying antirheumatic drugs: A one-year randomized, placebo-controlled study. Arthritis Rheum 2006; 54(9):2807-2816.

(24) Leandro MJ, Edwards JC, Cambridge G. Clinical outcome in 22 patients with rheumatoid arthritis treated with B lymphocyte depletion. Ann Rheum Dis 2002; 61(10):883-888.

(25) Ng CM, Bruno R, Combs D, Davies B. Population pharmacokinetics of rituximab (anti-CD20 monoclonal antibody) in rheumatoid arthritis patients during a phase II clinical trial. J Clin Pharmacol 2005; 45(7):792-801.

(26) Cambridge G, Stohl W, Leandro MJ, Migone TS, Hilbert DM, Edwards JC. Circulating levels of B lymphocyte stimulator in patients with rheumatoid arthritis following rituximab treatment: relationships with B cell depletion, circulating antibodies, and clinical relapse. Arthritis Rheum 2006; 54(3):723-732.

(27) Leandro MJ, Cambridge G, Ehrenstein MR, Edwards JC. Reconstitution of peripheral blood B cells after depletion with rituximab in patients with rheumatoid arthritis. Arthritis Rheum 2006; 54(2):613-620.

(28) Brown AK, Quinn MA, Karim Z, Conaghan PG, Peterfy CG, Hensor E et al. Presence of significant synovitis in rheumatoid arthritis patients with disease-modifying antirheumatic drug-induced clinical remission: evidence from an imaging study may explain structural progression. Arthritis Rheum 2006; 54(12):3761-3773.

(29) Popa C, Leandro MJ, Cambridge G, Edwards JC. Repeated B lymphocyte depletion with rituximab in rheumatoid arthritis over 7 yrs. Rheumatology (Oxford) 2007; 46(4):626-630.

(30) Uniform requirements for manuscripts submitted to biomedical journals. International Committee of Medical Journal Editors. N Engl J Med 1997; 336(4):309-315.

(31) Macrophage function changes following rituximab treatment in patients with rheumatoid arthritis. Ann Rheum Dis. 2006 Dec 5; [Epub ahead of print]

(32) O'Dell JR, Leff R, Paulsen G, et al. Treatment of rheumatoid arthritis with methotrexate and hydroxychloroquine, methotrexate and sulfasalazine, or a combination of the three medications: results of a two-year, randomised double-blind, placebo-controlled trial. Arthritis Rheum 2002;46(5):1164-1170

(33) Emery P. et al. Sustained efficacy of repeat treatment courses of rituximab in rheumatoid arthritis patients with an inadequate response to disease-modifying anti-rheumatic drugs [abstract] Annals of the Rheumatic Diseases 2007; 66 (Suppl II): SAT0007

(34) van Vollenhoven R. et al, Long term safety data from extended follow up and repeat use of rituximab in rheumatoid arthritis [abstract] Annals of the Rheumatic Diseases 2007; 66 (Suppl II): OP0119)

(35) Koen Vos, Rogier M. Thurlings, Carla A. Wijbrandts, Dirkjan van Schaardenburg, Daniëlle M. Gerlag, Paul P. Tak: Early effects of rituximab on the synovial cell infiltrate in patients with rheumatoid arthritis. Arthritis Rheum 2007: 56 (3): 772-778

(36) P. Emery, D.E. Furst, G. Ferraccioli, J. Udell, R.F. Van Vollenhoven, K. Rowe, S. Agarwal, T. Shaw: Long term efficacy and safety of a repeat treatment course of rituximab in RA patients with an inadequate response to disease-modifying anti-rheumatic drugs [abstract] Annals Rheum Dis 2006; 65 (Suppl. 1): OP0017

(37) Keystone E et al. Repeated Treatment Courses of Rituximab Produce Sustained Efficacy in Patients with Rheumatoid Arthritis and an inadequate Response or Intolerance to One or More TNF Inhibitors. Arthr & Rheum 2007. 56; 9: S794

(38) Thurlings et al. Arthr & Rheum 2007. 56; 9: S1290

(39) Cragg MS, Morgan SM, Chan HTC, Morgan P, Filatov PB, Johnson PWM, French RR, Glennie MJ: Complement-mediated lysis by anti-CD20 mAb correlates with segregation into lipid rafts. Blood 2003, 101:1045-52

(40) Fransen J, Creemers MCW , Van Riel PLCM. Remission in rheumatoid arthritis: agreement of the disease activity score (DAS28) with the ARA preliminary remission criteria. Rheumatology. 2004; 43:1252-1255

(41) Wells GA, Tugwell P, Kraag GR, Baker PRA, Groh J, Redelmeier DA :Minimum important difference between patients with rheumatoid arthritis: the patient's perspective. J Rheumatol 1993, 20:557-560.

Appendices

Appendix 1: Rheumatoid Arthritis Functional Class

- **I**: Completely able to perform usual activities of daily living (self-care, vocational, and avocational)
- **II**: Able to perform usual self-care and vocational activities, but limited in avocational activities
- **III**: Able to perform usual self-care activities, but limited in vocational and avocational activities
- **IV**: Limited in ability to perform usual self-care, vocational, and avocational activities

NB. If **IV**, do not include the patient in the study.

Usual self-care activities include dressing, feeding, bathing, grooming and toileting. Avocational (recreational and/or leisure) and vocational (work, school, homemaking) activities are patient-desired and age and sex specific.

Appendix 2: Pharmacogenetic assessment

Pharmacogenetic Research

Pharmacogenetics - Background

Pharmacogenetics (PGx) is the study of variability in drug response due to hereditary factors in different populations. There is increasing evidence that an individual's genetic composition (i.e., genotype) may impact the pharmacokinetics (absorption, distribution, metabolism, elimination), pharmacodynamics (relationship between concentrations and pharmacologic effects or the time course of pharmacologic effects) and/or clinical outcome (in terms of efficacy and/or safety and tolerability). Some reported examples of PGx analysis include:

Drug	Disease	Gene	Outcome
Abacavir	HIV [Hetherington, 2002; Mallal, 2002]	HLA (human leukocyte antigen)	Caucasian males with HLA B57 variant were at increased risk for experiencing hypersensitivity to abacavir
Tranilast	Restenosis prevention following coronary bypass [Roses, 2002]	UGT1A1	Drug induced hyperbilirubinemia explained by high proportion of affected patients having 7/7 TA repeat genotype, consistent with clinically benign Gilbert’s Syndrome
ABT-761	Asthma [Drazen, 1999]	ALOX5	ALOX5 Sp1 promoter genotype (x,x) associated with reduced response to 5-lipoxygenase inhibitor ABT-761

A key component to successful PGx research is the collection of samples during the conduct of clinical studies.

Collection of whole blood samples, even when no a priory hypothesis has been identified, may enable PGx analysis to be conducted if at any time it appears that there is a potential unexpected or unexplained variation in handling or response to ofatumumab.

Pharmacogenetic Research Objectives

The objective of the PGx research (if there is a potential unexpected or unexplained variation) is to investigate a possible genetic relationship to handling or response to ofatumumab. If at any time it appears there is potential variability in response in this clinical study or in a series of clinical studies with ofatumumab that may be attributable to genetic variations of patients, the following objectives may be investigated:

- Relationship between genetic variants and the pharmacokinetics of the investigational product.
- Relationship between genetic variants and safety and/or tolerability of the investigational product.
- Relationship between genetic variants and efficacy of the investigational product.

Study Population

Any patient who has given informed consent to participate in the clinical study, has met all the entry criteria for the clinical study, and receives investigational product may take part in the PGx research. Any patient who has received an allogeneic bone marrow transplant must be excluded from the PGx research.

Patient participation in the PGx research is voluntary, and refusal to participate will not indicate withdrawal from the clinical study. Refusal to participate will involve no penalty or loss of benefits to which the patient would otherwise be entitled.

Study Assessments and Procedures

In addition to any blood samples taken for the clinical study, a whole blood sample (~10ml) will be collected for the PGx research using a tube containing EDTA. The PGx sample is labeled (or coded) with a study specific number that can be traced or linked back to the patient by the investigator or site staff. Coded samples do not carry personal identifiers (such as name or social security number). The blood sample will be taken on a single occasion unless a duplicate sample is required due to inability to utilize the original sample. It is recommended that the blood sample be taken at the first opportunity after a patient has been randomized and provided informed consent for PGx research, but may be taken at any time while the patient is participating in the clinical study.

If deoxyribonucleic acid (DNA) is extracted from the blood sample, the DNA may be subjected to sample quality control analysis. This analysis will involve the genotyping of several genetic markers to confirm the integrity of individual samples. If inconsistencies are noted in the analysis, then those samples may be destroyed.

The need to conduct PGx analysis analyses may be identified after a study (or set of studies) of ofatumumab has been completed and the study data reviewed.

In some cases, the samples may not be studied. e.g., no questions are raised about how people respond to ofatumumab.

Samples will be stored securely and may be kept for up to 15 years after the last patient completes the study, or the sponsor may destroy the samples sooner. The Sponsor or those working with the sponsor (for example, other researchers) will use samples collected from the study for the purpose stated in this protocol and in the informed consent form.

Patients can request their sample to be destroyed at any time.

Patient Withdrawal from Study

If a patient who has consented to participate in PGx research and has a sample taken for PGx research withdraws from the clinical study for any reason other than lost to follow-up, the patient will be given the following options:

1. The sample is retained for PGx research
2. Any PGx sample is destroyed.

If a patient withdraws consent from the PGx research or requests sample destruction for any reason, the investigator must complete the appropriate documentation to request sample destruction within the timeframe specified by the sponsor and maintain the documentation in the site study records. In either case, the sponsor will only use study information collected/generated up to that point.

Screen and Baseline Failures

If a blood sample for PGx research has been collected and it is determined that the patient does not meet the entry criteria for participation in the clinical study, then the investigator must complete the appropriate documentation to request sample destruction within the timeframe specified by the sponsor and maintain the documentation in the site study records.

Pharmacogenetics Analyses

Generally the sponsor will utilize two approaches to explore genetic variation in drug response.

1. Specific sections of DNA may be selected from areas of the genome (e.g., candidate genes) known to encode the drug target, drug metabolizing enzymes, areas associated with mechanisms underlying adverse events, and those linked to study disease and, thus, linked to drug response. The candidate genes that may be investigated in this study are the following: The CD20 molecule which represents the target of ofatumumab on B-lymphocytes; and receptors and regulatory proteins that may have a role in the antibody-dependent cell-mediated cytotoxicity and complement-dependent cytotoxicity elicited by ofatumumab including but not restricted to: 1) the V158F single nucleotide polymorphism in the FCGR3A gene which has been associated with increased efficacy to Rituximab [Cartron, 2003; Weng, 2003] and is a receptor on effector immune cells to which Ofatumumab ofatumumab binds; and 2) polymorphisms and/or copy number variation in complement subunits (e.g. C1Q, C1S, C2, C4, Factor H) and complement inhibitors (e.g. CD55, CD59). In addition, continuing research may identify other enzymes, transporters, proteins, or receptors that may be involved in response to ofatumumab. The genes that may code for these proteins may also be studied.

2. By evaluating large numbers of polymorphic markers (e.g., single nucleotide polymorphisms or SNPs) throughout the genome, sets of markers may be identified that correspond to differential drug response.

Hardy-Weinberg Equilibrium testing

The genotypic frequencies of each polymorphism will be evaluated for conformity to those expected under normal conditions by employing Hardy-Weinberg Equilibrium testing.

Comparison of Demographic and Baseline (Visit 2) Characteristics by Genotype

Differences in baseline clinical characteristics and potential contributing covariates may be summarized and compared among genotype (or haplotype) subgroups.

Evaluation of Genotypic Effects

Analyses may be carried out to evaluate the degree of association between patient genotype (or haplotype) and selected parameters (e.g., pharmacokinetics, efficacy and safety). Where such genotypic tests are inappropriate (for example, where the number of marker genotypes is too large and/or the frequency of individual genotypes too small), allelic tests may be conducted. Allelic tests evaluate whether the frequency of each marker allele is the same in responders and non-responders.

Evaluation of Treatment by Genotype and Gene-Gene Interaction

In addition to evaluating the main effects of the genotypes (haplotypes or alleles) on the selected parameters, the possibility of a treatment group by genotype (haplotype or allele) interaction will also be explored. If appropriate, the joint effects of multiple markers (gene-gene interactions) may also be evaluated.

Linkage Disequilibrium

For pairs of polymorphisms, the degree to which alleles from the two sites are correlated (linkage disequilibrium) may also be evaluated. If the genotypes at two polymorphic sites within a gene are shown to be statistically associated with a response to the investigational product, the degree of linkage disequilibrium will the aid interpretation in that it will indicate the extent to which the two sites are exerting independent effects.

Multiple Comparisons and Multiplicity

Adjustment to observed p-values may be made to limit erroneous conclusions due to multiple tests when multiple markers are evaluated (especially in the case of a genome scan for association).

Power and Sample Size Considerations

The ability to detect differential drug response among genotypes at a polymorphic site depends on the total number of patients genotyped and the frequency distribution of the different genotypes. Consequently, genotyping analyses are plausible for those polymorphic sites where the number of patients comprising the genotypic groups is sufficiently large; however, these frequencies will not be known until sufficient samples have been collected and genotyping is complete.

Estimates of sample sizes required to demonstrate genotype effects vary considerably, depending on the assumptions made about allele frequency, genetic effect size, and mechanism of inheritance [Cardon, 2000]. In the work by Palmer and Cookson [Palmer, 2001], which assumed a genotype relative risk of 1.5, it was estimated that more than 300 cases and 600 controls would be needed to conduct a genetic association analysis. In contrast, McCarthy and Hilfiker [McCarthy, 2000] showed that with a genotype relative risk of 2.16 and a relatively commonly occurring genotype, only 30 cases and 30 controls would be needed to demonstrate an association.

Published PGx examples include abacavir hypersensitivity reaction [Hetherington, 2002; Mallal, 2002] and tranilast induced hyperbilirubinemia [Roses, 2002] where genetic markers have been found to significantly associate with a hypersensitivity reaction (abacavir) and hyperbilirubinemia (tranilast). These examples show that small sample sizes typically encountered in Phase I and Phase II studies may be sufficient to identify clinically relevant genetic associations.

Informed Consent

Patients who do not wish to participate in the PGx research may still participate in the clinical study. PGx informed consent must be obtained prior to any blood being taken for PGx research.

Provision of Study Results and Confidentiality of SubjectPatient's PGx Data

The sponsor may summarize the cumulative PGx research results in the clinical study report. In general, the sponsor does not inform the investigator, patient or anyone else (e.g., family members, study investigators, primary care physicians, insurers, or employers) of the PGx research results because the information generated from PGx studies is preliminary in nature, and the significance and scientific validity of the results are undetermined at such an early stage of research, under any circumstance unless required by law.

References

Cardon LR, Idury RM, Harris TJR, Witte JS, Elston RC. Testing drug response in the presence of genetic information: sampling issues for clinical trials. Pharmacogenetics. 2000; 10:503-10.

Cartron G, Dacheux L, Salles G, Solal-Celigny P, Bardos P, Colombat P, Watier H. Therapeutic activity of humanized anti-CD20 monocolonal antibody and polymorphism in IgG Fc receptor FcgRIIIa gene. Blood 2003; 99: 754-758.

Drazen JM, Yandava CN, Dube L, Szcerback N, Hippensteel R, Pillari A, Israel E, Schork N, Silverman ES, Katz DA, Drajesk J. Pharmacogenetic association between ALOX5 promoter genotype and the response to anti-asthma treatment. Nature Genet. 1999; 22:168-70.

Hetherington S, Hughes AR, Mosteller M, Shortino D, Baker KL, Spreen W, Lai E, Davies K, Handley A, Dow DJ, Fling ME, Stocum M, Bowman C, Thurmond LM, Roses AD. Genetic variations in HLA-B region and hypersensitivity reactions to abacavir. Lancet. 2002; 359:1121-2.

Mallal S, Nolan D, Witt C, Masel G, Martin AM, Moore C, Sayer D, Castley A, Mamotte C, Maxwell D, James I. Association between presence of HLA-B*5701, HLA-DR7, and HLA-DQ3 and hypersensitivity to HIV-1 reverse-transcriptase inhibitor abacavir. Lancet. 2002; 359:727-32.

McCarthy JJ, Hilfiker R. The use of single-nucleotide polymorphism maps in pharmacogenomics. Nat Biotechnol. 2000; 18:505-8.

Palmer LJ, Cookson WO. Using single nucleotide polymorphisms as a means to understanding the pathophysiology of asthma. Respir Res. 2001; 2:102-12.

Roses AD. Genome-based pharmacogenetics and the pharmaceutical industry. Nat Rev Drug Discov. 2002; 1:541-9.

Weng W-K and Levy R. Two immunoglobulin G fragment C receptor polymorphism independently predict response to rituximab in patients with follicular lymphoma. J. Clin. Oncol. 2003; 21: 3940-3947.

Amendment to Clinical Trial Protocol GEN411/OFA110634, version 1

Title: A double-blind, randomized, placebo controlled, parallel group, multi-center, phase III trial of ofatumumab investigating clinical efficacy in adult patients with active rheumatoid arthritis who had an inadequate response to TNF-α antagonist therapy

Substantial Amendment: ☒

Non-substantial Amendment: ☐ Rationale:

Urgent Amendment: ☐

Protocol Amendment No.: 5

Internal approval date: 22 October 2010

Reasons for Protocol Amendment

1. **Termination of study (section 1.7 -figure 1, 6.1 , 6.5, 6.6, 10 and 17)**
   The primary purpose of this amendment is to update the protocol related to the Sponsor's decision to stop clinical development of the intravenous delivery of ofatumumab in autoimmune indications, including rheumatoid arthritis, as announced in a press release issued on Sept 16th 2010 by GSK and Genmab. Since a regulatory filing with the intravenous route of administration in rheumatoid arthritis will no longer be pursued, the Sponsor has decided to prematurely terminate study GEN411/OFA110634 in accordance with Section 17 of the protocol. After approval of the amendment, no patients will receive retreatment with ofatumumab. At the next scheduled study visit patients in Double-blind and Open-label periods will proceed into the planned Follow-up period where they will be monitored every 12 weeks until B-cells and circulating IgG levels have returned to normal or baseline levels or for a maximum of 2 years from the last scheduled visit in the Double-blind and Open-label Period, whichever occurs earlier.

2. **During Safety Follow-up IgG levels will be monitored instead of IgM (section 6.1, 6.5, 8, and 9.16)**
   As the risk of serious infections is associated with hypogammaglobulinemia (low IgG levels) rather than low circulating IgM levels, it is more appropriate to monitor patients until their IgG levels instead of IgM levels return to normal or baseline values in the Follow-up period.

3. **Additional text and changes to sections have been made to be more in alignment with GSK protocol wording & SOPs (section 5.3.3, 6.11, 7.1.1, 7.1.2, 7.1.3, 12.2, 12.4 and 14.2)**
   Although GSK was operationalizing this study, the previous Sponsor of this trial was the partner company Genmab, whose protocol template had been utilized, upon transfer of sponsorship of this study from Genmab to GSK based on the amendment of the ofatumaumb co-development agreement signed on July 1st 2010, additional mandatory wording and changes to some sections based on GSK protocol template and SOPs have been implemented with this amendment.

4. **Additional Safety Follow-up assessments (Table 2, and section 8.2.2 and 8.2.8)**
   To ensure compliance with GSK SOPs and the ongoing safety of patients throughout the Follow-up period additional pregnancy, Hepatitis B & C and liver function testings were included to the Follow-up period at specific timepoints.

5. **Administrative changes (section 3.2.5, 5.2. 5.3.1, 6.1, 6.4.2 , 8.2.8 and 9.5.3)**
   In addition to amending typographical errors, some sections have been updated to ensure accuracy of information.

Amended text

Amended text is struck through with a single line and new text is written in italics

1.7 Trial Design

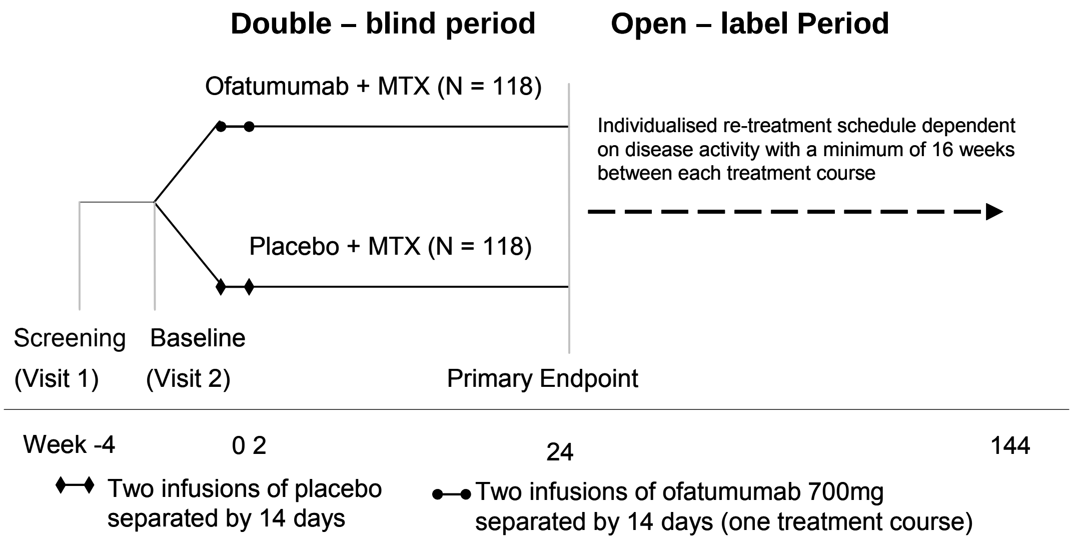

Figure 1: Trial Design
In the event the trial is prematurely terminated, all subjects in Double-blind or Open-label Periods will enter the Follow-Up Period at the next scheduled study visit.

2 Flow Chart

Screening[1]	Double-Blind Period	Open-Label Period[2]								Follow-Up	
**LABORATORY ASSESSMENT[17]** (Samples to be drawn prior to infusion unless stated otherwise)											
Period	28 days	24 weeks								120 weeks	Individualized[18]
Visit Number	1	2	3	4	5	6	7	8	9 / early withdrawal	10+	FU+
Day/Week[19]	≤4w	0d	14d	4w	8w	12w	16w	20w	24w	Every 8 w	Every 12 w
Visit Window (days)	≤28 prior to v2	+1	±2	±3	±3	±3	±3	±3	±3	±14	±14
Biochemistry/hematology	X	X	X	X	X	X	X	X	X	X	*X[20]*
Urine test	X	X	X	X	X	X	X	X	X	X	
Pregnancy Test[21]	X	X	X	X	X	X	X	X	X	X[22]	*X[23]*
IgA, IgG, IgM	X	X				X			X	X	X[24]
Flow Cytometry		X	X	X		X			X	X	X[25]
Plasma/white cell JCV PCR	X					X			X	X[28]	X
ESR[26]	X	X		X	X	X	X	X	X	X	
CRP	X	X		X	X	X	X	X	X	X	
HAHA	X	X	X						X	X[28]	
PK		X[27]	X[27]	X		X		X	X		
Hepatitis B	X								X	X[28]	*X[29]*
Hepatitis C[30]	X										*X[31]*
Rheumatoid Factor (RF)	X [32]	X									
Transcriptomics		X									
Anti-Cyclic Citrullinated Peptide (anti-CCP)		X									
Other biomarkers (incl. BLyS)		X		X		X			X		
Pharmacogenetics (PGx)[33]		X									

[17] All analysis will be performed by a central laboratory unless stated otherwise. Details of paremeters can be found in Section 8
[18] Patients will be followed until B-cells and circulating *IgG* ~~immunoglobulins~~ have returned to normal or baseline levels, *or a maximum of 2 years from the last scheduled visit in the Double-blind or Open-label Period, whichever occurs earlier*~~: it is anticipated that the follow-up period will be in the order of a maximum of 2 years period~~
[19] Visit dates are relative to baseline date (Visit 2)
[20] *During Follow-up biochemistry/hematology tests are ONLY to be performed at week 12 and 24 from the last scheduled visit in the Double-blind or Open-label Period..*
[21] Pregnancy tests~~ing~~ at screening, end of Double-Blind Period/early withdrawal and at end of Open-Label Period are based on serum-HCG. All other urine-pregnancy tests are done locally, if positive a confirmatory serum-HCG should be done
[22] In the Open-label period monthly urine pregnancy testing should be performed. If a visit is not scheduled, home urine testing must be performed
[23] *A urine pregnancy test is ONLY to be performed at week 12 and 24 from the last scheduled visit in the Double-blind or Open-label Period.*
[24] All patients will be followed with regards to IgG~~M~~ levels. ~~If patients are withdrawn due to low levels of IgG this will be measured as well~~
[25] CD19+ only
[26] ESR is measured locally utilizing the Westergren method
[27] PK- sampling should be done prior to infusion and immediately after the end of infusion
[28] Should only be taken every 24weeks, i.e. at week 48, 72, 96, 120 and 144
[29] *During Follow-up Hep B test is to be performed every 24 weeks for a maximum of 2 years from the last scheduled visit in the Double-blind or Open-label Period.*
[30] If Hepatitis C antibody positive, a Hepatitis C RIBA® immunoblot assay should be reflexively performed on the same sample to confirm the results (See Section 5.2)
[31] *During Follow-up Hep C test is to be performed every 24 weeks f or a maximum of 2 years from the last scheduled visit in the Double-blind or Open-label Period.*
[32] Only rheumatoid factor (RF) seropositivity / - negativity is measured at screening. RF quantitative will be measured at all visits indicated in the Flow Chart
[33] The PGx sample may be collected at any visit during the trial. The PGx sample is not mandatory

3.2.5 Summary of known and potential risks to human subjects

The following reactions have been observed in patients exposed to ofatumumab and should be regarded as expected infusion-related adverse reactions associated with administration of this antibody: pruritus, dyspnoea, throat irritation, rigors/chills, headache, flushing, nausea, hypotension, urticaria, fatigue, fever, and rash. However, experience to date shows that these infusion-related reactions can be prevented or reduced in frequency and severity when patients are administered pre-medication prior to ofatumumab infusion as described in section 3.3.4. Profound and prolonged depletion of peripheral $CD20^{+}$ B lymphocytes has been observed during treatment with ofatumumab, as expected with the administration of an anti-CD20 monoclonal antibody. To date, an increased risk of *serious* infectious complications associated with ~~this type of B cell depletion~~ *ofatumumab* has not been observed.

Further information can be found in the Investigator's Brochure.

5.2 Exclusion Criteria

16) Serologic evidence of Hepatitis B (HB) infection based on the results of testing for HBsAg, anti-HBc and anti-HBs *antibodies* as follows:

- Patients positive for HBsAg are excluded
- Patients negative for HBsAg but positive for both anti-HBc and anti-HBs antibodies are eligible to participate
- Patients negative for HBsAg and anti-HBc antibody but positive for anti-HBs antibody are eligible to participate
- Patients negative for HBsAg and anti-HBs antibody but positive for anti-HBc antibody will require clarification of their status by testing for HB*V* DNA which if positive will exclude the patient from participation.

5.3.1 Withdrawal from treatment

A patient should be withdrawn from ofatumumab/placebo if at any time:

- It is the wish of the patient (or their legally acceptable representative) for any reason
- The investigator judges it necessary due to medical reasons
- The patient becomes pregnant
- The patient receives prohibited therapy as listed en section 7.4.2
- An adjustment in concomitant MTX is initiated except for decreases in dose due to toxicity
- If the patient at any time during the trial (including the Follow-up Period) enters another

interventional clinical study

- The patient has an abnormal ECG finding judged by the investigator to be clinically relevant
- The patient has an abnormal liver chemistry as defined in section 9.5.2
- The patient becomes positive for hepatitis B infection[1]
- The patient develops symptoms of PML
- If, after the second treatment course and subsequent re-treatments with ofatumumab in the Open-label Period, if the patient has not at least once achieved a
  - moderate EULAR response compared to Visit 2 values (for definition of a EULAR response please refer to section 10.3.4) **and/or**
  - 20% improvement in both swollen and tender joint counts (compared to baseline value from Double-blind Period, i.e. Visit 2) *within 24 weeks since the last ofatumumab re-treatment course*

  ~~within 24 weeks since the last ofatumumab re-treatment course~~

Double-blind Period

If the patient is withdrawn from ofatumumab/placebo during the Double-blind Period the patient should continue protocol assessments until completion of the Double-blind Period at 24 weeks. Thereafter, the patient should proceed to the Follow-up Period as outlined in Section 6.5. Due to safety reasons the patient should only be offered the non-biological DMARDs which ~~is~~ *are* described in 7.4.1

5.3.3 Withdrawal from Safety Follow-up

A patient should be withdrawn from the Follow-up if at any time:

- *It is the wish of the patient (or their legally acceptable representative) for any reason*
- *The investigator judges it necessary due to medical reasons*
- *The patient initiates treatment with other B-cell suppressive treatment (e.g. other anti-CD20 antibodies, cyclophosphamide, azathioprine etc.)*

- *The patient enters another interventional clinical trial and/ or receives treatment with any non-marketed drug substance or experimental therapy.*

6.1 Overall Design

Second and subsequent re-treatment courses:

After the first re-treatment course, patients will be eligible to receive further ofatumumab re-treatment courses during the 120-week Open-label Period if she/he:

- Achieves an efficacy response to ofatumumab at least once within 24 weeks from the last ofatumumab re-treatment course, defined as at least a moderate EULAR response **and/or** ≥20% improvement in both swollen and tender joint counts (compared to baseline value from Double-blind Period, i.e., Visit 2).

  PLUS

Following this response, subsequently shows ~~an~~ *a* worsening in disease activity at any time since the last ofatumumab re-treatment course defined as:

- DAS28 ≥ 3.2 **PLUS** a ≥0.6 increase in DAS28 compared to lowest DAS28 after the last re-treatment course **and/or** a ≥20% increase in both tender and swollen joint counts compared to lowest counts since the previous treatment course

Note, in the Open-label Period the interval between initiation of a new treatment course for patients should be at least 16 weeks (16 weeks is counted from infusion A from the preceding infusion course) irrespective of progression in disease activity. The last treatment course should be planned to occur no later than the scheduled visit at week 120 after baseline (Visit 2), i.e. Infusion A of the last treatment course must be given no later than at week 120.

Patients who do not obtain at least a moderate EULAR response **or** ≥20% improvement in both swollen and tender joint counts (compared to baseline values from Double-blind Period, i.e., Visit 2) within 24 weeks from the first day of infusion in the previous treatment course in the Open-label Period will be withdrawn from further ofatumumab treatment and proceed to the Follow-up Period.

In addition, any patient with a circulating IgG level <lower limit of normal (as assessed by the central laboratory) at any time after the second treatment course will be withdrawn from further ofatumumab treatment and enter the Follow-Up Period. In the Follow-up Period the patient will be followed until the number of B-cells, and circulating *IgG* ~~and IgM~~ have returned to normal (according to the central laboratory) or baseline (i.e. Visit 2) levels *or for a maximum of 2 years from the last scheduled visit in the Double-Blind or Open-label Period, whichever occurs earlier.*

Patients who have completed the Open-label Period or who do not qualify for re-treatment will be followed (i.e., in the Follow-up Period) until the number of B-cells and circulating *IgG*~~M~~ have returned to normal (according to the central laboratory) or baseline (i.e. Visit 2) levels ~~It is anticipated that the Follow-up Period will be in the order of a 2 years period~~ *or for a maximum of 2 years from the last scheduled visit in the Double-Blind or Open-label Period, whichever occurs earlier.*

*In the event the trial is prematurely terminated, all subjects in Double-blind or Open-label Period will enter the Follow-Up Period at the next scheduled study visit (see section 6.5 for details on Follow-up activities)***.**

6.4.2 Open-label Period (Visit 9 and forwards)

The interval between initiation of a new treatment course should be at least 16 weeks irrespective of progression in disease activity. Sixteen weeks ~~is~~ *are* counted from infusion A from the preceding infusion course. Assessments will be performed every 8 weeks (± 2 weeks) independent of disease activity. If disease activity progresses between scheduled visits, an unscheduled visit should be performed. Pain management in the form of i.a. corticosteroid injection, analgesics or NSAIDs can be given to patients. If re-treatment criteria are fulfilled, infusion visits (two visits separated by 14 days) should be planned as soon as possible, and no later than 14 days after worsening of disease activity, in parallel to the scheduled visits performed every 8 weeks (please refer to separate Flow Chart in section 2 for assessments to be done at infusion visits). An infusion visit and a scheduled visit could be combined if within the visit windows of the scheduled visit. The last treatment course should be planned to occur no later than the scheduled visit at week 120 after baseline (Visit 2).

6.5 Follow-up Period

Patients who have completed the Open-label Period *or are withdrawn from the Double-blind or Open-label Period of the trial (section 5.3and 17)* will be followed every 12 weeks until B-cells, and circulating Ig*G*~~M~~ have returned to normal (according to central laboratory) or baseline levels (i.e. Visit 2) *or for a maximum of 2 years from the last scheduled visit in the Double-blind or Open-label Period, whichever occurs earlier* ~~It is anticipated the Follow-up Period will be in the order of a 2 year period~~. Neurological examinations and plasma/white cell JCV PCR testing will also be performed during Follow-up. At these visits, patients will also be followed for SAEs and concomitant RA medication. Patients who have been withdrawn due to low levels of IgG will in addition to the above also be followed until the level of circulating IgG has returned to normal (according to central laboratory) or baseline level (i.e. Visit 2), or for a maximum of 2 years from the last scheduled visit in the Double-blind or Open-label period.

If the patient initiates treatment with other B-cell suppressive treatment (e.g., other anti-CD20 antibodies, cyclophosphamide, or azathioprine), at any time during the trial including the Follow-

Up Period *or enters another interventional trial while in the Follow-Up Period*, all patient related study activities according to this trial protocol should be terminated. Furthermore, the investigator should document the date of initiation of other B-cell suppressive treatment *or date of initiation of other trial* and complete the End of Study Conclusion Form.

6.6 Treatment after the End of the Study

The investigator is responsible for ensuring that consideration has been given to the post-study care of the patient's medical condition.

Note: GSK will not provide any post study treatment with ofatumumab, and no compassionate use of ofatumumab for autoimmune indications is currently available for rheumatoid arthritis patients

6.11 Emergency Unblinding procedure

Emergency unblinding of patients will take place by using the IVRS.

Unblinding is restricted to emergency situations and should only be used under circumstances where knowledge of the treatment is necessary for the ~~proper handling~~ *appropriate clinical management or welfare of the subject* ~~of the patient~~. *Whenever possible, the investigator must first discuss options with the GSK Medical Monitor or appropriate GSK study personnel* ***before*** *unblinding the subject's treatment assignment. If this is impractical, the investigator must notify GSK as soon as possible, but without revealing the treatment assignment of the unblinded subject, unless that information is important for the safety of subjects currently in the study.*

GSK's Global Clinical Safety and Pharmacovigilance (GCSP) staff may unblind the treatment assignment for any subject with an SAE. If the SAE requires that an expedited regulatory report be sent to one or more regulatory agencies, a copy of the report, identifying the subject's treatment assignment, may be sent to clinical investigators in accordance with local regulations and/or GSK policy.

All SAEs and Suspected Unexpected Serious Adverse Reactions (SUSARs) will be unblinded within safety in accordance with regulatory requirements.

If the treatment blind is broken, *the investigator will;*

1) Contact the CRA (preferably before unblinding)

2) Document date and reason for unblinding in the Patient's Medical Record

3) Store the confirmation of unblinding from IVRS in the Investigator Site File

7 Treatment

7.1 Investigational Medicinal Product (IMP)

7.1.1 fatumumab

Ofatumumab is a clear colorless liquid. Ofatumumab is a concentrate for solution intended for intravenous administration.

Ofatumumab is formulated at 20 mg/mL ~~adjusted to pH 6.5 (Table 1).~~ Ofatumumab is supplied in 6 mL clear glass vials. Each vial contains 5 mL of ofatumumab (20 mg/mL), i.e. a total of 100 mg ofatumumab.

~~**Table 1: Investigational Medicinal Product**~~

~~Ingredient~~	~~Quantity per mL~~	~~Function~~
~~Ofatumumab~~	~~20 mg~~	~~Active ingredient~~
~~Sodium Citrate USP/EP~~	~~8.549 mg~~	~~Buffering and stabilizing agent~~
~~Citric Acid USP/EP~~	~~0.195 mg~~	~~Buffering and stabilizing agent~~
~~Sodium Chloride USP/EP~~	~~5.844 mg~~	~~Isotonic agent~~
~~Water for injection USP/EP q.s. to~~	~~1 mL~~	~~Solvent~~

Ofatumumab/placebo will be filtered using an inline filter (0.2 µm) during infusion.

7.1.2 Packaging and Labeling of ofatumumab/placebo

Ofatumumab will be supplied to the site or pharmacy in cartons, each carton containing 10 vials. Labeling will be in accordance with all applicable regulatory requirements:

~~**Table 2: Labeling of Outer Carton and Vial**~~

~~Label on Outer Carton~~	~~Label on Vial~~
~~Study identifier and EudraCT number~~	~~Study identifier and EudraCT number~~
~~Ofatumumab, 20 mg/mL, 10 vials of 5 mL drug product~~	~~Ofatumumab, 20 mg/mL, 5 mL drug product~~
~~Concentrate for solution for infusion IV~~	~~Concentrate for solution for infusion IV~~
~~Store at 2-8 °C~~	~~Store at 2-8 °C~~
~~Packaging Number~~	~~Batch and Packaging Number~~
~~For clinical trial use only /<br>Caution: New drug – Limited by Federal law to investigational use~~	~~For clinical trial use only /<br>Caution: New drug – Limited by Federal law to investigational use~~
~~Sponsor details~~	~~Sponsor details~~
~~Expiry Date and ID #~~	

~~**Table 3: Labeling of infusion bag**~~

~~**Label on infusion bag**~~
~~Study identifier and EudraCT number~~
~~Ofatumumab / placebo in 0.9% NaCl, 1000 mL~~
~~Caution: Do always use in-line filter~~
~~Patient ID no.:~~
~~Preparation date and time:~~
~~Prepared by:~~
~~To be infused at room temperature within 24 hours of preparation~~
~~For clinical trial use only /<br>Caution: New drug – Limited by Federal law to investigational use~~
~~Investigator name:~~
~~Sponsor details~~

7.1.3 Storage of ofatumumab

Ofatumumab should be stored in a safe and secure place in a refrigerator at 2-8°C, *protected from light* and it must not be frozen.

After ofatumumab has been diluted in sterile, pyrogen free 0.9% NaCl it can be kept at room temperature and must be given to the patient within 24 hours. Exact time of dilution into 0.9% NaCl must be written on the label of the infusion bag.

Drug supplies must be kept in an appropriate restricted area, which may be accessed only by the pharmacist, or a duly designated person. A log to document the temperature with daily readings (working days only) must be kept.

If the temperature of the refrigerator is outside the limits of 2-8°C (35.6-46.4 °F) it should be noted in the temperature log. If the temperature is / has been ≤ 0 °C or ≥ 10°C (≤ 32 °F or ≥ 50 °F) for more than 8 hours the local CRA should be contacted. The following information should be available: the study number, amount of ofatumumab and data on the temperature in the refrigerator, and for how long the temperature was outside the temperature limits. If a break down of the refrigerator occurs, ofatumumab should be transferred to another temperature controlled refrigerator immediately.

Ofatumumab must not be utilized after the expiry date printed on the carton label.

8 Trial Assessments

An overview of visits is given in a Flow Chart in Section 2. The trial consists of a 24 week Double-blind Period followed by a 120 week Open-label Period. After the last visit in the 120 week Open-label Period, patients will be followed every 12 weeks (Follow-up Visits) until normalization of circulating Ig*G*~~M~~ and $CD19^+$ cells *for a maximum of 2 years from the last scheduled visit in the Double-blind or Open-label Period.*

8.2.2 Pregnancy Test

For women of childbearing potential pregnancy testing will be performed. Blood samples will be drawn at screening, end of Double-Blind Period/early withdrawal, and at end of Open-Label Period. Serum samples are shipped to the central laboratory for immediate analysis of Human Chorionic Gonadotrophine (HCG).
If the patient is an early withdrawal from treatment in the Double-blind Period and continues with the remaining visits serum-HCG testing is not applicable at the early withdrawal from treatment visit. Instead, the serum-HCG testing will be done at the end of the double-blind period, i.e. when the patients attend the last visit prior to progressing to the follow-up period.

All remaining pregnancy tests (*including a test at week 12 and 24 from the last scheduled visit in the Double-Blind or Open-label Period)* will be based on urine sampling and will be analyzed locally. A negative urine pregnancy test should be obtained and documented. If the urine pregnancy test is positive a serum-*HCG* pregnancy test should be obtained. Protocol assessments can only be re-commenced if results of the serum pregnancy test are negative.

In the Open-label Period monthly urine pregnancy testing should be performed for women of childbearing potential. If a visit is not scheduled, home urine testing must be performed.

Women are considered of childbearing potential unless they have been hysterectomized, or have undergone tubal ligation at least one year prior to Visit 1 (Screening), or have been postmenopausal for at least one year.

The date of sampling and outcome will be noted. If sampling for pregnancy test is not applicable, the reason for this will be documented.

8.2.8 Hepatitis B *and C*

Patients will be evaluated for serologic evidence of Hepatitis B (HB) infection based on the results of testing for HBsAg, anti-HBc and anti-HBs antibodies as follows:

- Patients positive for HBsAg are excluded
- Patients negative for HBsAg but positive for both anti-HBc and anti-HBs antibodies are eligible to participate.

- Patients negative for HBsAg and anti-HBc antibody but positive for anti-HBs antibody are eligible to participate.
- Patients negative for HBsAg and anti-HBs antibody but positive for anti-HBc antibody will require clarification of their status by testing for HB*V* DNA which if positive will exclude the patient from participation.

Patients with documented vaccination against Hepatitis B (primary and secondary immunization and booster) will be considered negative.

If at any time during the study patients test positive by the criteria described above (and this is not due to a previous vaccination), the patient should be withdrawn from further trial treatment and proceed to Follow-up.

A blood sample will also be collected and analyzed for Hepatitis C antibodies at screening, and every 24 weeks during follow-up for a maximum of 2 years from the last scheduled visit in the Double-blind or Open-label Period. If the result is positive the viral load for Hepatitis C will be analyzed in another blood sample by a confirmatory assay. Both blood samples will be taken on the same day but will be stored and shipped ambient or frozen, respectively.

9.1.6 Adverse Event Reporting

The investigator must report all directly observed AEs and all AEs spontaneously reported by the patient. A general type of question should be used similar to, “Do you have any health problems?” or, “Have you had any health problems since your last visit?”

All AEs that occur in patients during the AE reporting period must be reported, whether or not the event is treatment related.

The AE reporting period (for SAE reporting period, see Section 9.3.2) begins from first treatment (Visit 2/Day 0) until the patient terminates the trial. Any signs or symptoms occurring between Screening/Visit 1 and Visit 2 should be recorded as Medical History. During the Follow-Up Period when the patient’s B-cells and IgG (if applicable) and Ig*G*~~M~~ levels are followed, only AEs that meet one or more of the serious criteria (see Section 9.3.1) will be reported to sponsor. The Serious Adverse Event Form in the eCRF should be completed.

9.5.3 Infections and malignancies

As part of the ongoing program to evaluate the benefit/risk of ofatumumab, this trial will include enhanced safety monitoring during the trial with regard to serious infections, malignancy, and deaths. While serious infections and malignancies have been observed in RA patients, a reasonable possibility of a causal association between ofatumumab and these events has not been established. Cases of the opportunistic viral infection progressive multifocal leukoencephalopathy (PML) have been reported in patients with hematologic malignancies, ~~and~~ systemic lupus erythematosus *and rheumatoid arthritis* treated with another anti-CD20 antibody, rituximab. Additionally, cases of

PML have occurred in patients who have not received rituximab. Most reports have been in patients with a compromised immune system, either due to medical conditions (lymphoma or blood cancers, HIV infection and congenital immunodeficiency syndromes and systemic lupus erythematosus) or medical treatments (cancer chemotherapy and immunosuppressive medications in organ transplant recipients)[2]. In the Hx-CD20-406 trial one case of PML was observed in a CLL patient who had received ofatumumab (1x300mg & 10x2000mg) previously treated with fludarabine and alemtuzumab with low T-lymphocyte CD4 counts.

In order to accommodate potential developments of PML, neurological examinations and assessments of JCV PCR will be performed throughout the duration of the trial (see Section 0). Once identified, signs and symptoms consistent with a diagnosis of PML will be reported promptly to sponsor. Signs and symptoms of PML include visual disturbances, ocular movements, ataxia, and changes in mental status such as disorientation or confusion. These symptoms are not an exhaustive list and the investigator should exercise judgment in deciding to report signs and symptoms to sponsor promptly. Refer to the Study Procedures Manual for further information and detailed guidance for completing and transmitting these and other SAE reports for patients who experience a serious infection, malignancy, death, or sign or symptom of PML. If a patient develops neurological signs or symptoms consistent with PML, study drug is to be discontinued and the patient referred to a neurologist for evaluation. At a minimum, blood JCV PCR and/or *brain* MRI will be performed and if either is positive perform Cerebrospinal Fluid (CSF) JCV PCR. If blood JCV PCR and *brain* MRI are negative, the investigator will contact sponsor for appropriate action to be taken with study drug. If blood JCV PCR and/or *brain* MRI are positive, the patient should proceed to the Follow-Up Period. All such patients will be followed until resolution. Any patient with a diagnosis of PML will be withdrawn from ofatumumab / placebo.

The investigator will do the following when reporting a serious infection, malignancy, death, or sign/symptom consistent with PML.

- Promptly report the event, as with any other SAE, as per Section 9.3.2 of this protocol.
- Provide key source documentation for sponsor to assist with the safety evaluation process.

Examples of key source documents include but are not limited to: hospitalization records, discharge summaries, laboratory evaluations, biopsy results, culture/sensitivity results, death certificates, and autopsy reports.

If the patient has not otherwise been withdrawn from the study, then the investigator should contact sponsor to discuss the appropriate course of action regarding study continuation.

[2] Ref FDA Alert 12/2006

10 Statistics

This section presents the principal features of the statistical analysis of this trial. Further details will be given in a separate Reporting and Analysis Plan (RAP), which will be finalized before breaking the blind.

The analyses and presentations will be performed for the Intent-to-treat dataset unless specified otherwise.

The significance level is set to 5%. All confidence intervals will be two-sided.

All summary statistics of continuous variables will include: n, mean, median, standard deviation, minimum and maximum. All summaries presenting frequencies and incidences will include n, % and N, where N is the total number of patients with recorded values in the corresponding group.

All data listings will include all randomized patients.

In the event the sponsor decides to suspend or discontinue the intravenous route of administration development program for RA and this trial is terminated early such that there are insufficient subjects to achieve 90% power, no statistical testing will be performed and only limited efficacy data will be summarized. Safety data will be reported as planned. Further details will be included in the RAP.

12.2 Monitoring

Monitoring visits to the trial site will be made periodically during the trial, to ensure that all aspects of the protocol are followed. Source documents will be reviewed for verification of agreement with data on the eCRF. ~~The Investigator/institution guarantees direct access to source documents by sponsor and appropriate regulatory agencies.~~

To ensure compliance with GCP and all applicable regulatory requirements, GSK may also conduct a quality assurance audit of the site records, and the regulatory agencies may conduct a regulatory inspection at any time during or after completion of the study. In the event of an audit or inspection, the investigator (and institution) must agree to grant the auditor(s) and inspector(s) direct access to all relevant documents and to allocate their time and the time of their staff to discuss any findings/relevant issues

~~The trial site may also be audited (quality assurance) by sponsor as well as inspected by appropriate regulatory agencies.~~

It is important that the Investigator and his/her relevant personnel are available during the monitoring visits and possible audits and that sufficient time is devoted to the process.

12.4 *Study and Site Closure*

Upon completion or termination of the study, the GSK monitor will conduct site closure activities with the investigator or site staff (as appropriate), in accordance with applicable regulations, GCP, and GSK Standard Operating Procedures.

GSK reserves the right to temporarily suspend or terminate the study at any time for reasons including (but not limited to) safety issues, ethical issues, or severe non-compliance. If GSK determines that such action is required, GSK will discuss the reasons for taking such action with the investigator or head of the medical institution (where applicable). When feasible, GSK will provide advance notice to the investigator or head of the medical institution of the impending action.

If a study is suspended or terminated for ***safety reasons****, GSK will promptly inform all investigators, heads of the medical institutions (where applicable),and/or institutions conducting the study. GSK will also promptly inform the relevant regulatory authorities of the suspension/termination along with the reasons for such action. Where required by applicable regulations, the investigator or head of the medical institution must inform the IRB/IEC promptly and provide the reason(s) for the suspension/termination.*

~~14.2 Publication~~

~~Sponsor acknowledges the Investigator's right to publish the entire results of the trial, regardless of the outcome, in accordance with the latest Vancouver rules(30).~~

~~The International Coordinating Investigator will together with sponsor decide on the publication strategy and has the right to publish and present the results and methods as first author of multicenter publications. Co-authorship will be decided by sponsor and International Coordinating Investigator and will be limited to a number of persons, who have contributed substantially in the conduct of the trial. Sponsor will have representation in the list of authors.~~

~~Publications are subject to the following conditions:~~

- ~~No publication prior to the completion of the trial at all participating sites without written approval from sponsor.~~
- ~~All proposed publications and presentations, including any modifications or amendments shall be submitted to sponsor for its review at least 30 days before such presentation or publication is submitted to any third party.~~
- ~~Publications shall not disclose any Sponsor Confidential Information and Property (not including the trial results, which can be published as described elsewhere in this section).~~

14.2 *Provision of Study Results to Investigators, Posting to the Clinical Trials Register and Publication*

Where required by applicable regulatory requirements, an investigator signatory will be identified for the approval of the clinical study report. The investigator will be provided reasonable access to statistical tables, figures, and relevant reports and will have the opportunity to review the complete study results at a GSK site or other mutually-agreeable location.

GSK will also provide the investigator with the full summary of the study results. The investigator is encouraged to share the summary results with the study subjects, as appropriate.

GSK will provide the investigator with the randomization codes for their site only after completion of the full statistical analysis.

The results summary will be posted to the Clinical Study Register at the time of the first regulatory approval or within 12 months of any decision to terminate development. In addition, a manuscript will be submitted to a peer-reviewed journal for publication within 12 months of the first approval or within 12 months of any decision to terminate development. When manuscript publication in a peer-reviewed journal is not feasible, further study information will be posted to the GSK Clinical Study Register to supplement the results summary.

17 Premature Termination of the Trial

If sponsor or International Coordinating Investigator discovers conditions arising during the trial, which indicate that the clinical investigation should be halted, the trial can be terminated after appropriate consultation between sponsor and International Coordinating Investigator. The Regulatory Authorities and Independent Ethics Committees/Institutional Review Board will be notified in writing. The reason will be stated.

Conditions that may warrant termination of the trial include, but are not limited to the following:

- The discovery of an unexpected and significant or unacceptable risk to the patients enrolled in the trial.
- The discovery of lack of efficacy.
- Failure of the Investigators to enter patients at an acceptable rate in the trial as a whole.
- A decision on the part of sponsor to suspend or discontinue development of the *drug or formulation of drug* ~~drug~~ in this indication.

In the event the trial is prematurely terminated, all subjects in Double-blind or Open-label Periods will enter the Follow-Up Period at the next scheduled study visit (see section 6.5 for details on Follow–up activities)
