# Supplementary material for: Safety of Repeated Open-Label Treatment Courses of Intravenous Ofatumumab, a Human Anti-CD20 Monoclonal Antibody, in Rheumatoid Arthritis: Results from Three Clinical Trials
Source: PLoS One. 2016 Jun 23;11(6):e0157961. doi: 10.1371/journal.pone.0157961 (PMC4919033; doi:10.1371/journal.pone.0157961)
Supplement: S3 Protocol — (PDF) [file pone.0157961.s004.pdf]

CLINICAL TRIAL PROTOCOL

Long-term efficacy and safety of repeated ofatumumab treatment courses in RA patients who previously received ofatumumab or placebo in Trial Hx-CD20-403

An open-label, international, multi-center, phase II, extension trial investigating long-term efficacy and safety of repeated treatment courses of ofatumumab, a fully human monoclonal anti-CD20 antibody, in adult patients with active rheumatoid arthritis who previously received ofatumumab or placebo in Trial Hx-CD20-403

**Confidential**	Final: 03-Sep-2007<br>Trial ID: GEN413/OFA111752<br>Amendment 1: 19-Nov-2007<br>Amendment 2: 27-Jun-2008<br>Amendment 3: 03-Sep-2008<br>Amendment 4: 15-Oct- 2008<br>Amendment 5: 06-Feb-2009<br>Amendment 6: 05-Nov-2010

This Study Protocol is the property of GSK and is a confidential document. It is not to be copied or distributed to other parties without written approval from GSK.

Table of Contents

Table of Contents 2
List of Tables 5
List of Figures 5
List of Attachments 5
1 Protocol Summary 6
1.1 Objectives 6
1.2 Endpoints 6
1.3 Trial Population 8
1.4 Number of Patients 8
1.5 Trial Design 8
1.6 Investigational Product 9
2 Flow Chart 10
3 Background and Rationale 13
3.1 Disease & Treatment 13
3.1.1 Rheumatoid arthritis 13
3.1.2 Current Treatment of Rheumatoid Arthritis 14
3.2 Investigational Medicinal Product 15
3.2.1 Ofatumumab 15
3.2.2 Pre-clinical Pharmacology 15
3.2.3 Pre-clinical Safety 16
3.2.4 Previous Clinical Experience 17
3.2.5 Summary of known and potential risks to human subjects 19
3.2.6 Study rationale 19
3.2.7 Study Population 19
3.2.8 Dose 19
3.2.9 Dose Schedule and Re-treatment 21
3.2.10 Pre-medication 22
4 Objectives 23
4.1 Primary Objective 23
4.2 Secondary Objective 23
4.3 Primary Endpoint 23
4.4 Secondary Endpoints 23
5 Patient Selection and Withdrawal 25
5.1 Inclusion Criteria 25
5.2 Exclusion Criteria 26
5.3 Withdrawal Criteria 29
5.3.1 Withdrawal from Treatment 29
5.3.2 Withdrawal from Trial 30
5.3.3 Withdrawal from Safety Follow-up 30
6 Trial Design 31
6.1 Overall Design 31
6.2 Schedule of Events 33
6.2.1 Screening (Visit 1) & Baseline (Visit 2_A) 33
6.2.2 Treatment 35
6.2.3 Follow-up 35
6.4 Recruitment period 36
6.5 Number of Patients 36
6.6 Treatment Allocation 37
7 Treatment 38
7.1 Ofatumumab 38

7.1.1 Ofatumumab....38
7.1.2 Packaging and Labeling of Ofatumumab....38
7.1.3 Storage of Ofatumumab....38
7.1.4 Drug Accountability and Compliance Check....38
7.2 Dosage of Ofatumumab....39
7.3 Handling of Ofatumumab....39
7.3.1 Pre-Medication....39
7.3.2 Preparation of Ofatumumab....40
7.3.3 Treatment Schedule....40
7.3.4 Administration of Ofatumumab....41
7.4 Concomitant Therapy....43
7.4.1 Therapy Allowed during Trial....43
7.4.2 Prohibited therapy and procedures during the study....45
8 Trial Assessment....46
8.1 Clinical Assessments....46
8.2 Demographic....46
8.3 Medical History....46
8.4 Height and Body Weight....46
8.5 Physical Examination....46
8.6 Neurological Examination....46
8.7 Electrocardiogram....47
8.8 Vital Signs....47
8.9 Adverse Events....47
8.10 Concomitant Medication....47
8.11 Administration of IMP....47
8.12 Tuberculosis....47
8.13 Clinical Efficacy Assessments....48
8.14 Joint Assessments....48
8.15 Visual Analogue Scale (VAS) of Pain....49
8.16 Patient and Physician VAS of Global Disease Assessment....49
8.17 Patient Reported Outcomes....49
8.18 Disability Index of the Health Assessment questionnaire (HAQ)....49
8.19 Laboratory Assessments....50
8.20 Biochemistry, Hematology and Urine test....50
8.21 Pregnancy Test....50
8.22 IgA, IgG, IgM....51
8.23 Flow Cytometry....51
8.24 Plasma/white cell JCV PCR....51
8.25 Host Immune Response (HAHA)....51
8.26 Pharmacokinetics (PK)....51
8.27 Hepatitis B and C....52
8.28 Biomarkers....52
8.29 Transcriptomics....53
8.30 Pharmacogenetics....53
8.31 CRP and ESR....53
9 Adverse Events....54
9.1 Definition of Adverse Events....54
9.1.1 Pre-existing Conditions....54
9.1.2 B-cell depletion....54
9.1.3 Study Disease....54
9.1.4 Procedures....54
9.1.5 Adverse Event Reporting....55
9.1.6 Pregnancy....55
9.2 Reporting Instructions....55

9.2.1 Recording Instructions 55
9.2.2 Diagnosis 55
9.2.3 Intensity 56
9.2.4 Relationship to Study Drug 56
9.2.5 Outcome 56
9.3 Serious Adverse Event (SAE) 56
9.3.1 Definition of a SAE 56
9.3.2 Serious Adverse Event Reporting 57
9.3.3 Suspected Unexpected Serious Adverse Reactions (SUSARs) 58
9.4 Follow-Up on Adverse Events 58
9.5 Safety Monitoring 58
9.5.1 Safety Surveillance set up 58
9.5.2 Liver Chemistry Abnormalities 59
9.5.3 Infections and malignancies 60
9.5.4 Critical Adverse Event 62
9.5.5 Stopping Rules 62
10 Statistics 64
10.1 Analysis Populations 64
10.2 Statistical Analysis of Primary Endpoint 64
10.3 Statistical Analysis of Secondary Endpoints 65
10.3.1 DAS28 65
10.3.2 EULAR Response 65
10.3.3 Time to first re-treatment 66
10.3.4 ACR20, AC50 and ACR70 66
10.3.5 ACRn and ACR components 67
10.3.6 Host Immune Response 67
10.3.7 Clinical Safety Data 67
10.3.8 Laboratory Safety Data 67
10.4 Patient reported outcomes 68
10.4.1 HAQ 68
10.5 Biomarkers, immune status, and whole blood transcriptional profiles 68
10.6 Statistical Analysis of Secondary Endpoints (Pharmacokinetics) 68
10.6.1 Pharmacokinetic Data 68
10.7 Handling of Missing Data or Outliers 68
10.8 Subgroups and Center Effects 68
10.9 Interim Analyses 68
10.10 Determination of Sample Size 68
11 Ethics 69
11.1 Independent Ethics Committee (IEC) or Institutional Review Board (IRB) 69
11.2 Patient Information and Informed Consent 69
11.3 Ethical and Risk/Benefit Considerations 69
12 Compliance with Good Clinical Practice 71
12.1 Monitoring 71
12.2 Source Data Verification 71
12.3 Study and Site Closure 72
13 Data Handling and Record Keeping 73
13.1 Case Report Form (CRF) 73
13.2 Archiving of Trial Documents at Site 73
14 Reporting and Communication of Results 75
14.1 Use of Information 75
14.2 Provision of Study Results to Investigators, Posting to the Clinical Trials Register and Publication 75
15 Insurance/Liability/Indemnity 76
16 Changes to the Final Protocol 77

17 Premature Termination of the Trial........78
17.1 Premature Termination of a Trial Site........78
18 List of abbreviations and definitions of terms........79
18.1 List of abbreviations........79
18.2 Definition of Terms........82
19 References........83
Appendices........87
Appendix 1: Rheumatoid arthritis functional class........87
Appendix 2: Pharmacogenetic assessment........88

List of Tables

Table 1: Ofatumumab dosages........39
Table 2: Pre-medication requirements before each infusion of ofatumumab........40
Table 3: Ofatumumab infusion preparation........40
Table 4: Infusion rate........41
Table 5: Measurements and PK sampling during infusion........42
Table 6: Maximum doses for i.a. / i.m. corticosteroids........44
Table 7: Timelines for reporting SAEs, pregnancies and liver function abnormalities........57
Table 8: Limits of CAE with error and detection rates........63
Table 9: EULAR Response Conversion Table........66

List of Figures

Figure 1: Trial Schedule........9
Figure 2: Trial Schedule........32
Figure 3: Entrance to the GEN413 trial........34

1 Protocol Summary

Trial ID: GEN413/OFA111752

Title: An open-label, international, multi-center, phase II, extension trial investigating long-term efficacy and safety of repeated treatment courses of ofatumumab, a fully human monoclonal anti-CD20 antibody, in adult patients with active rheumatoid arthritis who previously received ofatumumab or placebo in Trial Hx-CD20-403.

Short Title: Long-term efficacy and safety of repeated ofatumumab treatment courses in RA patients who previously received ofatumumab or placebo in Trial Hx-CD20-403.

1.1 Objectives

Primary:
To evaluate the long-term effectiveness of repeated courses of ofatumumab in RA patients who previously received ofatumumab or placebo in Trial Hx-CD20-403

Secondary:

- To evaluate the long-term safety of repeated courses of ofatumumab in RA patients who previously received ofatumumab or placebo in Trial Hx-CD20-403
- To evaluate the impact on patient reported outcomes after repeated courses of ofatumumab
- To evaluate the effect on established and novel biomarkers of clinical response after repeated courses of ofatumumab
- To evaluate host immune response against ofatumumab after repeated courses of ofatumumab

1.2 Endpoints

Primary:
Time to treatment withdrawal, defined as the time from first infusion of ofatumumab until date of treatment withdrawal.

Secondary:

Key Secondary Endpoints:

- Minimum DAS28 (based on ESR) score over 16-24 weeks in the first and last scheduled treatment cycles (last scheduled treatment cycle refers to the last treatment cycle administered if the patient is not withdrawn). For withdrawals the last available DAS28 score will be carried forward
- EULAR response at 16, 20, or 24 weeks in the first and last scheduled treatment cycles (last scheduled treatment cycle refers to the last treatment cycle administered if the patient is not withdrawn). Patients who are withdrawn from trial treatment are considered non-responders
- Time to first re-treatment, defined as the time from first infusion of ofatumumab until date of infusion 1 of the first re-treatment course

Other Secondary Endpoints:

- ACR20, ACR50 and ACR70 at 16, 20 and 24 weeks in the first and last scheduled treatment cycle (last scheduled treatment cycle refers to the last treatment cycle administered if the patient is not withdrawn). Patients who are withdrawn from trial treatment are considered non-responders.
- Maximum ACRn over 16-24 weeks in the first and last scheduled treatment cycle (last scheduled treatment cycle refers to the last treatment cycle administered if the patient is not withdrawn). For withdrawals the last available ACRn score will be carried forward
- Biomarkers of disease activity, immune status, and whole blood transcriptional profiles
- Human anti-human antibodies
- Adverse events, clinical laboratory parameters, vital signs, ECG
- Tender joint count (TJC)
- Swollen joint count (SJC)
- Patient VAS of pain
- Patient VAS of global disease
- Physician VAS of global disease
- Health Assessment Questionnaire (HAQ)
- C-reactive protein (CRP) and Erythrocyte sedimentation rate (ESR)
- $CD19^+$ cells
- Rheumatoid Factor (RF)

1.3 Trial Population

Patients eligible for this study are adults with active RA who previously received ofatumumab or placebo in Trial Hx-CD20-403.

1.4 Number of Patients

A total of 264 patients participated in Trial Hx-CD20-403, hence the maximum number of patients participating in Trial GEN413 is 264.

1.5 Trial Design

This is a 3-year open-label, phase II trial. Patients who previously participated in Trial Hx-CD20-403 and who fulfill the eligibility criteria for this trial (GEN413) will be offered participation.

All patients will initiate at least one treatment course of ofatumumab. One treatment course consists of two infusions of ofatumumab 700 mg (Infusions 1 and 2) separated by 2 weeks. The remaining treatment courses will be given at individualized time intervals when a clinical response has been achieved following the previous treatment course and a subsequent worsening in disease activity has been observed. The interval between each treatment course will be at least 16 weeks from Infusion 1 in the previous treatment course irrespective of progression in disease activity. The last treatment course should be planned to be initiated no later than week 130 after baseline (Visit 2_A).

After each treatment course the patients will attend their next trial visit 8 weeks after Infusion 1, followed by trial visits every 4 weeks up to Week 24, and subsequently every 8 weeks until the next treatment course.
The patients can receive a maximum of 9 treatment cycles.

After completing the Treatment Period or after withdrawing from the Treatment Period prematurely patients will be followed every 12 weeks (Follow-up Period) until $CD19^+$ cells have returned to baseline or normal levels (according to central laboratory normal ranges). For the purpose of the GEN413 trial, B-cell baseline is defined as the B-cell value measured prior to the administration of trial medication at Visit 2 during the Hx-CD20-403 trial. For patients withdrawn from Treatment Period prematurely due to IgG levels falling below normal range, IgG levels will also be followed. In cases where $CD19^+$ cells have returned to baseline or normal levels and the IgG level is still low, the patients will continue to be followed for IgG levels until normalization.

The patients can attend unscheduled visits any time during the trial. An unscheduled visit will include one or more of the assessments listed for a Visit 7, however, the actual assessments done for the individual patient will be selected at the discretion of the investigator.

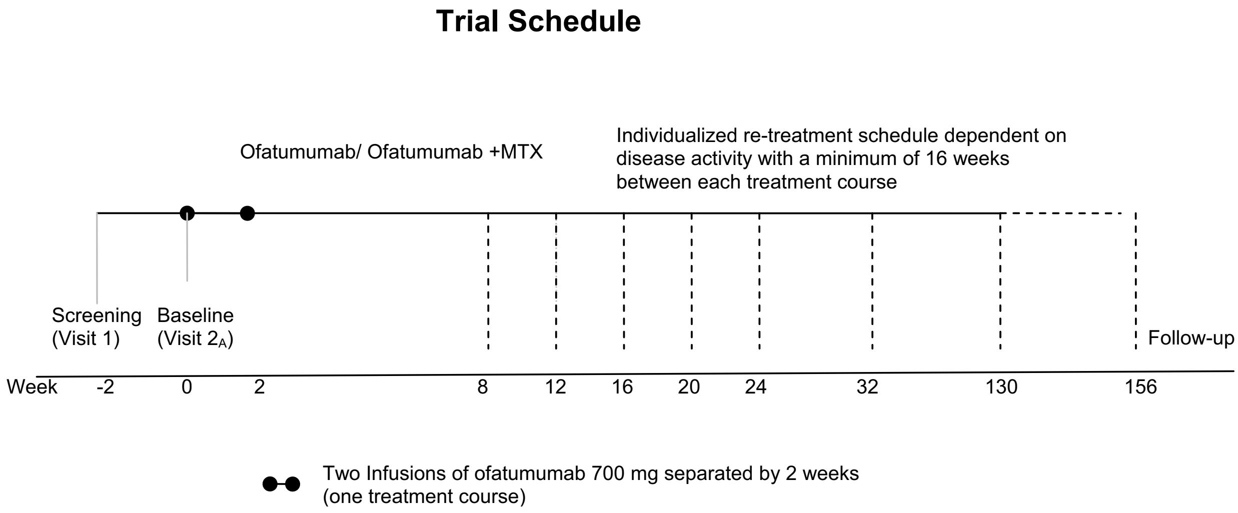

Figure 1: Trial Schedule

In the event the trial is prematurely terminated, all subjects in the Treatment Period will enter the Follow-Up Period at the next scheduled study visit.

Pain management as analgesics or NSAIDs will be allowed throughout the trial. Furthermore, one intra-articular or intra-muscular corticosteroid injection in one single joint or muscle every 6 month period will be permitted throughout the Treatment Period.

During the Follow-up Period there will be no restrictions with respect to pain management.

1.6 Investigational Product

Ofatumumab is a clear colorless liquid concentrate solution intended for intravenous administration. It is intended for administration after dilution into 1000 mL sterile, pyrogen free 0.9% NaCl. Ofatumumab will be filtered using an inline filter (0.2 µm) during infusion.

The initial infusion rate of the infusion will be 12 mL/hour for Infusion 1 and 25 mL/hour for Infusion 2 of each treatment course. During the infusion, the rate will be doubled every 30 minutes to a maximum of 400 mL/hour. Thereafter, the rate will be increased by 200 mL/hour every 30 minutes until 800 mL/hour is reached. The duration of Infusion 1 and Infusion 2 will be approximately 4 and 3½ hours, respectively, if this schedule is adhered to.

2 Flow Chart

Assessment (Assessments and samples should be done/drawn prior to infusion unless stated otherwise)										
**Period**	**Screening**	**Treatment Cycle A - I[1]**								**Follow-up**
Administration of ofatumumab		Infusion **1**	Infusion **2**							
Visit Number[2]	1	2_{A-I}	3_{A-I}	4_{A-I}	5_{A-I}	6_{A-I}	7_{A-I}	8_{A-I}	$9+_{A-I}$	FU+
Week	≤2w	Indivi-dualized	2w from Infusion 1	8w from Infusion 1	12w from Infusion 1	16w from Infusion 1	20w from Infusion 1	24w from Infusion 1	Every 8w	Every 12w
Visit Window (days)	≤14 prior to Visit 2_A	-	± 2	± 3	± 3	± 3	± 3	± 3	± 14	± 14
Informed Consent Form[3]	X									
Date of birth	X									
Eligibility Criteria	X	X[4]								
Medical History[5]	X									
Physical examination	X	X			X			X		
Weight		X[6]								
Neurological examination	X			X	X	X	X	X	X	X
ECG	X							X	X[7]	
Vital signs	X	X[8]	X[8]							X[9]

Table notes:

1) Each treatment cycle consists of minimum Visit 2 - 6, but may continue with additional visits until next treatment course becomes applicable. The patients can have maximum 9 treatment cycles (A - I).

2) If disease activity progresses between the scheduled visits an unscheduled visit should be performed.

3) Informed consent can be obtained outside the screening visit window i.e. may be prior to screening date. Informed consent should be obtained prior to any study related activity (including DMARD wash- out).

4) Swollen and tender joint counts should be re-assessed at baseline (Visit 2_A) to confirm patients meet requirements for inclusion criterion 4. Patients can not be allocated to treatment before inclusion criterion 4 is fulfilled. Where possible joint count reassessment should be performed at baseline (Visit 2_A); if this is not feasible it can be done ≤3 days prior to Visit 2_A.

5) Previous RA treatment will only be collected for the period between Visit 13 in Trial Hx-CD20-403 and Visit 1 in Trial GEN413.

6) Should only be measured prior to each treatment course.

7) Should only be assessed every 24 weeks within each treatment cycle.

8) Vital signs should be assessed prior to infusion, every hour during infusion and every hour during the observation period (see Table 7).

9) Only at the first Follow-Up Visit.

**Assessment (Assessments and samples should be done/drawn prior to infusion unless stated otherwise)**										
**Period**	**Screening**	**Treatment Cycle A - I**[1]								**Follow-up**
Administration of ofatumumab		Infusion **1**	Infusion **2**							
Visit Number[2]	1	2_{A-I}	3_{A-I}	4_{A-I}	5_{A-I}	6_{A-I}	7_{A-I}	8_{A-I}	$9+_{A-I}$	FU+
Week	≤2w	Indivi-dualized	2w from Infusion 1	8w from Infusion 1	12w from Infusion 1	16w from Infusion 1	20w from Infusion 1	24w from Infusion 1	Every 8w	Every 12w
Visit Window (days)	≤14 prior to Visit 2_A	-	± 2	± 3	± 3	± 3	± 3	± 3	± 14	± 14
Adverse Events[10]	X	X	X	X	X	X	X	X	X	X[11]
Concomitant Medication	X	X	X	X	X	X	X	X	X	X[12]
Tuberculosis[13]	X									
Efficacy Assessments<br>– Joint assessment[14]<br>– VAS Pain<br>– VAS Disease (patient)<br>– VAS Disease (physician)<br>– HAQ	X[15]	X		X	X	X	X	X	X	
Biochemistry/hematology	X	X	X	X	X	X	X	X	X	X[16]
Urine Test	X	X	X	X	X	X	X	X	X	

Table notes:

1) Each treatment cycle consists of minimum Visit 2 - 6, but may continue with additional visits until next treatment course becomes applicable. The patients can have maximum 9 treatment cycles (A - I).

2) If disease activity progresses between the scheduled visits an unscheduled visit should be performed.

10) SAEs are reported from time of informed consent. AEs occurring between Visit 1 and Visit 2_A should be recorded as Medical History.

11) Only Serious Adverse Events will be collected in the Follow-up Period.

12) Only concomitant RA medication will be collected.

13) X-ray obtained ≤12 weeks prior to screening as part of routine practice can replace the screening assessment. If clinically warranted the X-ray should be repeated at screening. The Mantoux test, or equivalent, is to be performed during the screening period. Patients who have documented BCG vaccination are exempt.

14) The joint assessment should be performed by an independent joint evaluator.

15) Only Joint assessments, Patient Pain assessment and patient global disease assessment.

16) During Follow-up biochemistry/hematology tests are ONLY to be performed at week 12 and 24 from the last scheduled visit in the Treatment Period.

**Assessment (Assessments and samples should be done/drawn prior to infusion unless stated otherwise)**										
**Period**	**Screening**	**Treatment Cycle A - I**[1]								**Follow-up**
Administration of ofatumumab		Infusion **1**	Infusion **2**							
Visit Number[2]	1	2_{A-I}	3_{A-I}	4_{A-I}	5_{A-I}	6_{A-I}	7_{A-I}	8_{A-I}	$9+_{A-I}$	FU+
Week	≤2w	Indivi-dualized	2w from Infusion 1	8w from Infusion 1	12w from Infusion 1	16w from Infusion 1	20w from Infusion 1	24w from Infusion 1	Every 8w	Every 12w
Visit Window (days)	≤14 prior to Visit 2_A	-	± 2	± 3	± 3	± 3	± 3	± 3	± 14	± 14
Pregnancy Test	X[17]	X	X	X	X	X	X	X	X	X[18]
IgA, IgG, IgM	X	X			X			X	X	X[19]
Flow Cytometry		X	X	X	X			X	X	X[20]
Plasma/white cell JCV PCR	X				X			X	X[21]	X
ESR[22]	X	X		X	X	X	X	X	X	
CRP	X	X		X	X	X	X	X	X	
HAHA	X	X						X	X[21]	
PK		X[23]	X[23]	X	X	X	X	X	X	
Hepatitis B and C[24]	X									X[25]
Biomarkers / transcriptomics		X		X	X	X		X	X	
Pharmacogenetics (PGx)		X[26]								

Table notes:

1) Each treatment cycle consists of minimum Visit 2 - 6, but may continue with additional visits until next treatment course becomes applicable. The patients can have maximum 9 treatment cycles (A - I).

2) If disease activity progresses between the scheduled visits an unscheduled visit should be performed.

17) Pregnancy testing based on serum-HCG will only be done at screening and monthly urine pregnancy testing performed at every clinic visit during the treatment cycle. All other urine-pregnancy tests are done locally; if positive a confirmatory serum-HCG should be done.

18) During Follow-up a urine pregnancy test is ONLY to be performed at week 12 and 24, from the last scheduled visit in Treatment Period..

19) During Follow-up immunoglobulins are to be performed every 12 weeks for a maximum of 2 years from the last scheduled visit in the Treatment Period.

20) $CD19^+$ only.

21) Should only be taken every 24 weeks within each treatment cycle.

22) ESR is measured locally.

23) PK-sampling should be done prior to infusion and immediately after the end of infusion.

24) If Hepatitis C antibody positive, a confirmatory Hepatitis C assay should be reflexively performed to confirm the results (see Section 8.27)

25) During Follow-up Hepatitis B &C tests are to be performed every 24 weeks for a maximum of 2 years from the last scheduled visit in the Treatment Period

26) Should only be taken at Visit 2_A.

3 Background and Rationale

3.1 Disease & Treatment

3.1.1 Rheumatoid arthritis

Rheumatoid arthritis (RA) is a chronic systemic inflammatory disease which affects 0.8-1.0% of all populations. The etiology of RA remains unknown.

RA primarily affects diarthrodial joints in a symmetrical distribution with an additive disease evolution over years (1). The hallmark of the disease is damage and disruption of joint integrity, starting early in the course of RA. In most cases, bone erosions progress leading to irreversible joint deformities. It is estimated that around one third of patients have radiographic evidence of bone erosions at the time of diagnosis, and this increases to almost 60% two years after diagnosis (2). Unless the inflammatory processes are halted or controlled, the disease leads to substantial disability, co-morbidities, and in many cases also premature death (3).

About 80% of RA patients present with rheumatoid factor (RF) (i.e., anti-IgG-Fc antibodies) in their serum, and this is associated with a poor prognosis (4). Along with more recent discoveries of other auto-antibodies, such as anti-cyclic citrullinated peptide (anti-CCP), the presence of autoantibodies strongly suggests that B-lymphocytes (B-cells) are involved in the pathogenesis of RA. Also, the observation, more than 20 years ago, of the presence of considerable numbers of cells of the B-cell lineage in the rheumatoid synovium, including RF-producing B-cells and plasma cells supports this notion (5). Previous research in animal models further demonstrated the critical role of B-cells in disease development as animals which lack functional B-cells or receive B-cell inhibitory signals are less prone to develop experimental arthritis (6).

Recent research established a clear role of cytokines, protein factors, and interaction of various cell populations including T-lymphocytes (T-cells), B-cells, and fibroblasts in the pathogenesis of the inflammatory lesion in the synovial membrane with consequent destruction of joint cartilage and bone. A recently discovered protein, B-lymphocyte stimulatory protein (BLys), is believed to contribute to B cell recruitment and activation in RA. Elevated concentrations of BLyS can be detected in the synovial fluid and serum of many patients with RA and in serum of patients with other inflammatory diseases (7).

An intimate relationship has also been established among dendritic cells, macrophages, B-cells, and T-cells within the RA synovium. In particular, dendritic cells and macrophages are clearly dependent on stimuli released by B lymphocytes, and this may explain why dendritic cell function may remain impaired beyond the recovery of B lymphocytes following B cell depletion (8;9). Cellular interactions such as antigen processing and presentation occur upstream of the pro-inflammatory cytokine response involving tumor necrosis factor-α (TNF-α), interleukin-1, and interleukin-6. In addition, production of IgM-RF and IgG-RF by plasma cells in the rheumatoid synovium may act as a self-perpetuating stimulus for local B-cell proliferation and differentiation (10).

Therefore, it appears increasingly evident that B-lymphocytes play a key pathogenetic role in the induction and maintenance of RA.

As B-lymphocytes are involved in various cellular interactions with other cells of the immune system in RA, B-cell depletion following ofatumumab treatment can be expected to affect disease activity within a few weeks after initiation of therapy. In addition, a prolonged beneficial effect can be anticipated with this treatment due to the upstream position of the B-cell system in relation to the classical pro-inflammatory cytokine pathways and to the slow recovery of this cell population as well. Remarkably, the clinical efficacy in RA achieved by depleting B-cells in the anti-TNF-α naïve RA patients occurs at a magnitude that is at least as high as that obtained with TNF-α blockade (11) or with down regulation of T-cell function with CTL4A-Ig (abatacept) (12).

3.1.2 Current Treatment of Rheumatoid Arthritis

Medications traditionally used in the treatment of RA belong to three categories: Non-steroidal anti-inflammatory drugs (NSAIDs), corticosteroids, and disease-modifying anti-rheumatic drugs (DMARDs). These are defined as medications that retard the radiographic progression of disease (joint destruction), Currently, DMARDs encompass both synthetic agents (small molecules) and the more potent biological agents.

Initiation of therapy with DMARDs within three months after the diagnosis of RA is crucial, because delayed therapy results in substantially more radiographic joint damage (13). Methotrexate is the synthetic DMARD which demonstrated in clinical trials to induce a long-term response and is therefore most often selected for initial therapy. Methotrexate has thus become the current standard of care in RA (14). Concomitant administration of folic acid significantly decreases methotrexate-induced toxic effects (15).

Methotrexate however is only effective (defined as obtaining an ACR20 response(ACR=American College of Rheumatology)) in about 50% of RA patients, and the effect tends to taper off four to six years after initial treatment by yet unknown mechanisms (16). Today, most patients not responding to methotrexate therapy will receive either therapy with new biologic agents (see below) or enter trials with combination therapy with other DMARDs (31), although the benefits of this combination therapy needs further evaluation (17).

Three biological products that inhibit the actions of TNF-α (infliximab, etanercept, and adalimumab) have been marketed for the treatment of RA in recent years. In clinical trials these agents induce ACR20 responses in about two thirds of patients and this efficacy was confirmed in several post-marketing studies.

Rituximab, a B-cell depleting chimeric anti-CD20 monoclonal antibody, was recently approved for the treatment of RA patients with an inadequate response to TNF-α blocking agents, as a statistically significant ACR20 response was achieved with this novel treatment compared to controls in clinical trials. Also in trials with anti-TNF-α naïve RA patients who have failed

methotrexate therapy, 2/3 of patients obtained an ACR20 response (18) lasting in some cases up to 2 years (19).

Recent data in RA patients relapsing after TNF-blocking treatments indicate that rituximab may be superior to alternative TNF-blocking treatments now available (20). However, rituximab is immunogenic and human anti-chimeric antibodies (HACA) have been observed in up to 25% of patients (21) thus a fully human anti-CD20 antibody is warranted.

Abatacept, a novel biologic treatment for RA based on a CTLA4-IgG construct (which acts by inhibiting co-stimulation, thus activation, of T-lymphocytes), was recently approved for treating RA patients with an inadequate response to MTX (12) and to TNF-α blocking agents (22). Antibodies binding to the entire abatacept molecule or to the CTLA-4 portion of abatacept have been seen in 1.7% of patients (see FDA prescribing information, March 2007). Moreover, abatacept treatment was also evaluated in combination with other DMARDs in a recent RA trial (22) and when used in combination with TNF-α blocking agents an increased risk of infection with the combination compared to the individual treatments was observed (23).

Thus, due to limitations of clinical efficacy and adverse reaction profile of synthetic and biologic DMARDs, alternative new therapies for RA are still needed.

3.2 Investigational Medicinal Product

3.2.1 Ofatumumab

Ofatumumab is a fully human IgG1κ lytic monoclonal antibody (mAb) that specifically binds to the human CD20 antigen whose expression is restricted to B lymphocytes from the pre-B to the plasmacytoid immunoblast stage only. The antibody is generated via transgenic mouse and hybridoma technology and produced in a recombinant murine cell line (NS0) using standard mammalian cell cultivation and purification techniques.

Ofatumumab recognizes an epitope (AA 143-164) on the human CD20 molecule which is different from the epitope recognized by the other anti-CD20 B-cell depleting agent rituximab (AA 165-172), and this property likely accounts for the higher efficiency of B-cell killing observed with ofatumumab in both in vitro and in vivo pre-clinical studies comparing these two agents.

Ofatumumab is currently being developed for the treatment of relapsed or refractory Non-Hodgkin B-cell follicular lymphoma (FL), for the treatment of relapsed or refractory chronic B-lymphocytic leukemia (CLL), and for the treatment of active rheumatoid arthritis (RA).

3.2.2 Pre-clinical Pharmacology

To characterize its features, ofatumumab has been tested in a series of in vitro and in vivo experiments, and in some instances compared to the reference antibody rituximab. A summary of data from the pharmacology and toxicology investigations, including important comparisons to rituximab, are provided below.

Ofatumumab represents a novel anti-CD20 mAb, which recognizes a CD20 epitope localized in the second extracellular loop distinct from the site recognized by rituximab. On human tissue sections ofatumumab demonstrated tissue reactivity that is consistent with its target antigen specificity.

Following the binding of ofatumumab to tumor B-cells clustering of CD20 into lipid rafts, as seen with rituximab, was observed. Strong differences in antibody cell-surface off-rates were detected between ofatumumab and rituximab. Ofatumumab dissociated much more slowly from surface CD20 than rituximab, corresponding to a half life of 180 minutes for ofatumumab and 90 minutes for rituximab. In this respect, both efficient clustering into rafts and a lower off-rate value for ofatumumab are important characteristics for an effective activation of complement and may explain the superiority of ofatumumab over rituximab in Complement Dependent Cytotoxicity (CDC) of B-cells.

Ofatumumab was able to induce complement-mediated lysis of freshly isolated human B-CLL tumor cells, and rituximab-resistant cells lines expressing low levels of CD20 and high levels of CD55/CD59, which is known to diminish the lytic effect of complement. Ofatumumab- and rituximab-mediated Antibody Dependent Cell-mediated Cytotoxicity (ADCC) were equivalent. Ofatumumab, as opposed to rituximab, did not induce cell death of tumor B-cell lines by apoptosis. Though ofatumumab mechanism of action is similar to that of rituximab, differences in the effector activity potentials exist between both mAbs.

In an in vivo study employing a SCID mouse tumor model, ofatumumab-treated animals showed a dose-dependent prolongation of survival that was superior to rituximab. A pilot pharmacokinetic study in cynomolgus monkeys demonstrated that depletion of CD20 expressing B lymphocytes occurred rapidly after antibody administration. Depletion of B cells with ofatumumab lasted four times longer than depletion observed after administration of a similar dose of rituximab. There were no adverse effects considered to be related to the ofatumumab treatment. In addition, a second pilot study in cynomolgus monkeys showed that a dose up to 150 mg/kg of ofatumumab did not induce any adverse effects and therefore, was considered a safe dose in these animals.

For further information see the current Investigator's Brochure.

3.2.3 Pre-clinical Safety

In a multiple dose toxicology study in cynomolgus monkeys, the toxicity of ofatumumab was investigated following four weekly doses by intravenous administration of 0, 20 or 100 mg/kg of ofatumumab. In the first set of animals, sacrificed two weeks after administration of the last dose, no changes in laboratory parameters, and no abnormal necropsy or histology findings (except for an expected germinal center atrophy after antibody treatment) were seen. Accordingly, analysis of CD20+ cells in peripheral blood and lymph node biopsies of all animals showed a rapid depletion of these cells after administration of ofatumumab. In the second set of animals sacrificed five months after administration of last dose, the B-cell counts in the peripheral blood in four out of six animals receiving 20 mg/kg of ofatumumab recovered to baseline levels at Days 133 to 149. Percentages of

CD20+ cells in the lymph nodes of all treated animals reached baseline levels at Days 112 to 182. In animals receiving 100 mg/kg of ofatumumab, four out of six animals showed full B-cell count recovery in the peripheral blood and lymph nodes at Days 112 to 182.

Subsequently, a multiple dose chronic toxicology study entitled "Multiple Dose Intravenous Toxicity Study in Cynomolgus Monkeys followed by a 6 Month Recovery Period" was initiated in 2005. The objectives of this study were to assess the toxicity of ofatumumab in cynomolgus monkeys following 13 intravenous administrations over a 7 month period (i.e. Days 1, 8, 15, 22, 29, 36, 43, 50, 78, 106, 134, 162 and 190) and then, during a 6 month recovery period, to monitor B-cell recovery in the blood and lymph nodes of designated animals. For this purpose, 21 male and 21 female cynomolgus monkeys were assigned to 3 dose groups in which ofatumumab was administered at dose levels of 0, 20 and 100 mg/kg/dose (Groups 1, 2 and 3, respectively). Two days after completion of the last dose administration, the Main study animals were sacrificed and sent for necropsy, and the remaining Recovery study animals were retained for an approximate 6 month recovery period. The chronic treatment of cynomolgus monkeys receiving 13 intravenous administrations of ofatumumab over a period of 7 months led to a depletion of B cells, which recovered to baseline levels in the majority of animals upon completion of a 6 month recovery period. No influence on cell numbers or immune system physiology, other than the target cell population, could be detected. In total 5 monkeys died or were humanely sacrificed due to deteriorating health during the course of the study. These deaths occurred in both treatment groups. Post-mortem investigation confirmed that the deaths or deterioration in health were attributable to infections and were not attributable to direct toxicity of the test item. The reasons for animal withdrawals can be characterized into those which had a probable C. jejuni infection and those which showed signs of hemolytic anemia. A review of hemolytic events indicated that the majority of treated animals were experiencing a slowly developing hemolytic anemia during both the dosing and the recovery periods, which were associated with a progressive, dose related increase in lactate dehydrogenase which was reversible. Direct Coomb's test results suggested that non-human primates probably developed a humoral response to ofatumumab which subsequently induced the formation of immune complexes capable of binding to the red cell surface. Preliminary results from the ongoing clinical studies indicate that ofatumumab does not induce hemolytic anemia in humans.

For further information see the current Investigator's Brochure.

3.2.4 Previous Clinical Experience

Ofatumumab has been administered to patients in eight phase I/II & III clinical trials, in B-cell Chronic Lymphocytic Leukemia (B-CLL), non-Hodgkin Follicular Lymphoma (FL), and Rheumatoid Arthritis (RA). Preliminary efficacy and safety data are available from one B-CLL and one FL trial. The remaining trials are still ongoing, and only limited safety data are available. Interim efficacy and safety data (24 weeks) are available for the rheumatoid arthritis study. For further information see the current Investigator's Brochure.

Follicular Lymphoma

A phase I/II trial investigating patients with relapsed or refractory FL grade I-II (Hx-CD20-001) has completed the treatment phase. It is an open-label, multi-center, and dose escalating trial testing doses in the ranges of 300 - 1000 mg (FL) administered as intravenous infusions once weekly for four weeks.

A phase III trial in patients with FL has been initiated (Hx-CD20-405). It is a double-blind, randomized, two-dose-arm trial testing doses in the range of 300 - 1000 mg, administered as intravenous infusions once weekly for eight weeks.

A phase II open-label, randomized, parallel group trial in 56 patients with previously untreated Follicular Lymphoma (Hx-CD20-409) has been initiated. Patients will receive an infusion of ofatumumab 3000 mg i.v. on day 0 in combination with CHOP on days 1-5. Thereafter, patients will receive either ofatumumab in doses of 500 mg or 1000 mg (1:1) at weeks 3, 6, 9, 12, and 15.

B-cell Chronic Lymphocytic Leukemia

For the phase I/II trials in patients with relapsed or refractory B-CLL (Hx-CD20-402), recruitment has been completed. It is an open-label, multi-center, and dose escalating trial testing doses in the ranges of 100 – 2000 mg administered as intravenous infusions once weekly for four weeks.

A phase III trial in patients with B-CLL (Hx-CD20-406) has been initiated. It is an open-label, multi-center trial testing doses in the range 300 – 2000 mg administered as intravenous infusions in a combination of weekly and monthly infusions.

A phase II two-arm trial (Hx-CD20-407) in which ofatumumab is investigated in previously untreated patients with B-CLL has been initiated. In this study ofatumumab is administered as 6 monthly infusions (1x300mg + 5x500mg) / (1x300mg + 5x1000mg) in combination with fludarabine and cyclophosphamide on days 1-3.

Rheumatoid Arthritis

A phase I/II trial investigating ofatumumab in patients with active RA (Hx-CD20-403) has been conducted. The first part (Part A) of the trial, which is the initial placebo-controlled dose-escalation part of the study, is completed. The second part (Part B), which has a placebo-controlled parallel group design, with randomization into one of four treatment arms, is of 48 weeks duration. The primary endpoint is ACR20 at week 24. This study is completed up to 24 weeks duration, with follow-up to 48 weeks ongoing. In Part A, 39 patients distributed across three cohorts were randomized (4:1 in each cohort) to receive two infusions of ofatumumab 300 mg (n=12), 700 mg (n=10), 1000 mg (n=10) or placebo (n=7) given two weeks apart. In Part B, 225 patients were randomized (1:1:1:1) to receive two infusions of ofatumumab 300 mg (n=58), 700 mg (n=57), 1000 mg (n=54) or placebo (n=56) two weeks apart. In total, 201 RA patients have received ofatumumab.

Results from Part A and interim results (to 24 weeks) from Part B are described in the current Investigator's Brochure.

3.2.5 Summary of known and potential risks to human subjects

The following reactions have been observed in patients exposed to ofatumumab and should be regarded as expected infusion-related adverse reactions associated with administration of this antibody: pruritus, dyspnea, throat irritation, rigors/chills, headache, flushing, nausea, hypotension, urticaria, fatigue, fever, and rash. However, experience to date shows that these infusion-related reactions can be prevented or reduced in frequency and severity when patients are administered pre-medication prior to ofatumumab infusion as described in Section 3.2.10. Profound and prolonged depletion of peripheral $CD20^{+}$ B lymphocytes has been observed during treatment with ofatumumab, as expected with the administration of an anti-CD20 monoclonal antibody. To date, an increased risk of serious infectious complications associated with ofatumumab has not been observed.

Further information can be found in the current Investigator's Brochure.

3.2.6 Study rationale

Data from the ongoing RA phase I/II trial, Hx-CD20-403, (i.e., final data from Part A and interim data from Part B) have shown ofatumumab to be generally well tolerated in patients with RA, with the majority of adverse events being reported on the day of ofatumumab infusion. In Part B significantly more patients achieved the primary endpoint, ACR20 response at Week 24, in the 300mg (41%), 700mg (49%) and 1000mg ofatumumab (46%) groups compared to placebo (15%). A similar benefit over placebo was demonstrated with other efficacy measures investigated in the trial including the Disease Activity Score with 28 joints (DAS28) and the EULAR response. Overall, these data support further investigation of the effectiveness and safety of ofatumumab in phase III clinical trials for rheumatoid arthritis.

3.2.7 Study Population

Patients eligible for this study are adults with active RA of at least six months' duration who have had an inadequate response to one or more DMARDs, and who have previously been exposed to ofatumumab or placebo in the Hx-CD20-403 trial.

Previous exposure to RA biologic cell-depleting therapies and to biological agents intended for RA treatment is allowed after a wash-out period.

3.2.8 Dose

Based on review of the efficacy and safety interim data from the phase I/II trial, Hx-CD20-403, a dose of 700mg ofatumumab (x2 infusions administered two weeks apart) for investigation in the phase III clinical program in subjects with rheumatoid arthritis has been selected. This is supported by the following data from study Hx-CD20-403:

- In the intention-to-treat study population, comprising 224 patients (55 on placebo, 58 on 300mg, 57 on 700mg and 54 on 1000mg), ACR20 response at week 24 was obtained by 41% (p=0.002), 49% (p<0.001) and 46% (p<0.001) of patients receiving 300mg, 700mg and 1000mg of ofatumumab compared to 15% in the placebo group. EULAR good/moderate response at week 24 was obtained by 72% in all dose groups compared to 40% in the placebo group. Median DAS28 changes at week 24 were -1.40, -1.44, -1.45 respectively with 300mg, 700mg, and 1000mg versus -0.50 with placebo.

- In the subgroup of patients receiving concomitant stable doses of methotrexate, comprising 178 patients, results across the three dose levels of ofatumumab studied showed that an ACR20 response was obtained by 42%, 56% and 50% of patients in the 300 mg, 700 mg and 1000 mg dose groups, respectively compared to 16% in the placebo group. Furthermore, for the EULAR and DAS28 efficacy outcomes all ofatumumab dose groups were superior to placebo with a numerically greater benefit demonstrated in the 700mg and 1000mg compared to the 300mg group in the subgroup of patients receiving concomitant methotrexate.

- Ofatumumab was generally well tolerated with a total of 81%, 84%, 83% and 57% of subjects in the 300mg, 700mg, 1000mg and placebo groups reporting at least one adverse event respectively. Approximately half of the adverse events noted in the ofatumumab group occurred on infusion days (51%) with the most frequently reported being mild or moderate (CTC AE grade 1-2 events), including throat irritation, dyspnea and rash. The proportion of patients who reported an AE on the day of 1st infusion was 28/58 (48%), 39/57 (68%) and 40/54 (74%) in the 300mg, 700mg and 1000mg groups respectively. The following AEs of grade 3 or greater (severe) were observed on the day of infusion as follows: first day: throat tightness (300mg); bronchospasm (2 events; both 700mg); fatigue (700mg); infusion related reaction (4 events; 1 in 700mg; 3 in 1000mg); hypersensitivity (1000mg); rash (1000mg) and on the second infusion day: IDDM (300mg); blood potassium increased (1000mg). Serious Adverse Events were reported in 3 (5%) subjects in the placebo group, 4 (7%), 5 (9%), 8 (15%) in the 300mg, 700mg and 1000mg groups respectively. Although there was a higher frequency of infusion-related adverse events reported in the 700mg and 1000mg ofatumumab groups compared to the 300mg group, following the modifications to the premedication regimen the majority were mild to moderate in severity and did not preclude treatment with ofatumumab.

- There is a demonstrated correlation between exposure (AUC, Cmax and Cmin) and ACR20 response such that responders had a significantly higher AUC, Cmin and Cmax than non-responders. This therefore indicates that the 700mg ofatumumab dose, by providing greater exposure may have the potential to provide improved efficacy compared to a 300mg dose.

Thus in summary, these data show 700 mg ofatumumab to be an effective and generally well tolerated dose and support selection of 700 mg ofatumumab (x2 infusions 2 weeks apart) for investigation in phase III rheumatoid arthritis trials and this extension study.

3.2.9 Dose Schedule and Re-treatment

Dose Schedule

Two i.v. infusions separated by 2 weeks has been chosen based on the following considerations:

Few studies have addressed the dosing schedule of rituximab. In an early paper by Leandro et al. (24), five cohorts of patients were studied. The first cohort of five patients received only one infusion and did not achieve ACR response whereas in the remaining 4 cohorts of patients receiving at least 2 infusions (a total of 17 patients), ACR responses were obtained. All subsequent studies with rituximab have used 2 infusions 2 weeks apart. A 2-compartment model based on such studies, used to analyze the population pharmacokinetic profiles, fits reasonably well with the PK data (25). Both the half life and Cmax increase considerably comparing the first and second dose. Likewise, the clearance is markedly reduced comparing the first and second dose. Therefore, 2 doses are selected to ensure maximal depletion of B cells in lymphoid tissues and bone marrow from which B cells are recruited following B cell depletion (26;27).

Similar data have been obtained with ofatumumab in rheumatoid arthritis (see Investigator's brochure and in the current Investigator's Brochure).

Re-treatment

The clinical efficacy of the 2-infusions cycle of rituximab therapy in rheumatoid arthritis patients who respond to the first cycle ("responders") usually lasts on average between 6 and 15 months in patients achieving an ACR20 (26) response, and in most cases B cells have returned to baseline levels before loss of clinical efficacy (27) is observed. Current scientific evidence on the pathophysiology of RA shows that a prolonged and tight control of inflammation is needed to secure clinical benefit and minimize joint structural damage (28), and this is why new biologic therapies such as TNF-α blocking agents and abatacept are indeed administered on a continuous basis. Furthermore, recent studies demonstrated long-term efficacy (29) without significant changes in the safety profile following re-treatment with rituximab when compared to single cycle therapy (29-31). As at the time of loss of response following the first B-cell depleting cycle B-lymphocytes levels usually remain below baseline levels (using conventional Flow-activated cell sorter (FACS) analyses) a rational approach to control the inflammatory and joint-destructive process in RA patients who respond to B-cell depleting therapy is to retreat them based on deterioration of their clinical status as, from present knowledge, this is not expected to alter the safety profile.

Moreover, synovial biopsy studies of patients treated with one cycle of rituximab demonstrated that in the majority only partial depletion of synovial B cells is achieved (32). Findings from an open-label study of patients with rheumatoid arthritis who were allowed re-treatment with rituximab if tender and swollen joint counts had improved ≥ 20%, and not necessarily meeting an ACR20 response, indicated that re-treatment may yield further depletion of synovial B-cells. In this study, the proportion of patients achieving ACR20 responses with the second course increased compared to the first course; thus the ACR20 response comparing the first and second treatment courses in the

same 99 patients increased from 59% to 73%, the ACR50 from 27% to 37% and the ACR70 from 9% to 19%, all of these differences reaching statistical significance (33). Re-treatment with an anti-CD20 depleting antibody may therefore achieve a clinical response in patients who did not meet an ACR20 response after the first treatment.

The phase I/II study of ofatumumab in RA patients (Hx-CD20-403) demonstrated minimal reduction of IgG and IgM following one treatment course. Therefore, assessments of IgG and IgM status to decide if patients can progress to the first re-treatment in the open-label period of this trial have been omitted. Patients who are IgM deficient and/or IgA deficient do not have an increased risk of infections as opposed to patients with low IgG (hypogammaglobulinemia or agammaglobulinemia). Therefore, the decision for second and subsequent re-treatment courses is based on IgG levels rather than IgM and IgA levels. Consequently, to assure the patients' safety, a minimum threshold level for IgG equivalent to the lower limit of normal has been set.

3.2.10 Pre-medication

In Part A of the ofatumumab phase I/II trial (Hx-CD20-403), patients were initially pre-medicated only with paracetamol (acetaminophen) and anti-histamines prior to the 2 infusions of ofatumumab 2 weeks apart. With this treatment regimen occurrence of grade >3 infusion-related AEs were observed, thus, pre-treatment with corticosteroids was subsequently implemented after issuing amendments to the protocol. In these amendments the dose of i.v. prednisolone (or similar) was increased up to 100 mg administered 1-2 hours prior to the ofatumumab infusions. An additional dose of prednisolone (100 mg orally the day before the first infusion and 30 mg the day before the second infusion) was also implemented. By these amendments, the incidences of grade 3 infusion-related AEs according to National Cancer Institute Common Terminology Criteria Adverse Events version 3.0 (NCI CTCAE) on the day of the first infusion was reduced from 14% to 6%; however, it was noted that pre-treatment with steroids the day before the infusions had little impact while a major reduction in the incidence of infusion-related grade 3 AEs occurred when the IMP was diluted in1000 mL rather than 500 mL saline, allowing for infusion of less drug substance at the initiation of the infusion. Following the increased dilution , the incidence of grade 3 infusion-related AEs among 169 patients was reduced from 6% to 3.5%. Hence, the major reduction of infusion-related grade 3 AEs was obtained by increasing the volume of the infusion to 1000 mL, thus, treatment with steroids the day before the infusions in this study is not required.

4 Objectives

4.1 Primary Objective

To evaluate the long-term effectiveness of repeated courses of ofatumumab in RA patients who previously received ofatumumab or placebo in Trial Hx-CD20-403.

4.2 Secondary Objective

- To evaluate the long-term safety of repeated courses of ofatumumab in RA patients who previously received ofatumumab or placebo in Trial Hx-CD20-403
- To evaluate the impact on patient reported outcomes after repeated courses of ofatumumab
- To evaluate the effect on established and novel biomarkers of clinical response after repeated courses of ofatumumab
- To evaluate host immune response against ofatumumab after repeated courses of ofatumumab.

4.3 Primary Endpoint

Time to treatment withdrawal, defined as the time from first infusion of ofatumumab until date of treatment withdrawal.

4.4 Secondary Endpoints

Key Secondary Endpoints:

- Minimum DAS28 (based on ESR) score over 16-24 weeks in the first and last scheduled treatment cycles (last scheduled treatment cycle refers to the last treatment cycle administered if the patient is not withdrawn). For withdrawals the last available DAS28 score will be carried forward
- EULAR response at 16, 20, or 24 weeks in the first and last scheduled treatment cycles (last scheduled treatment cycle refers to the last treatment cycle administered if the patient is not withdrawn). Patients who are withdrawn from trial treatment are considered non-responders
- Time to first re-treatment, defined as the time from first infusion of ofatumumab until date of infusion 1 of the first re-treatment course

Other Secondary Endpoints:

- ACR20, ACR50 and ACR70 at16, 20 and 24 weeks in the first and last scheduled treatment cycle (last scheduled treatment cycle refers to the last treatment cycle administered if the patient is not withdrawn). Patients who are withdrawn from trial treatment are considered non-responders.
- Maximum ACRn over 16-24 weeks in the first and last scheduled treatment cycle (last scheduled treatment cycle refers to the last treatment cycle administered if the patient is not withdrawn). For withdrawals the last available ACRn score will be carried forward

- Biomarkers of disease activity, immune status, and whole blood transcriptional profiles
- Human anti-human antibodies
- Adverse events, clinical laboratory parameters, vital signs, ECG
- Tender joint count (TJC)
- Swollen joint count (SJC)
- Patient VAS of pain
- Patient VAS of global disease
- Physician VAS of global disease
- Health Assessment Questionnaire (HAQ)
- C-reactive protein (CRP) and Erythrocyte sedimentation rate (ESR)
- $CD19^+$ cells
- Rheumatoid Factor (RF)

5 Patient Selection and Withdrawal

To be eligible the patient has to meet all inclusion criteria and must not meet any of the exclusion criteria.

5.1 Inclusion Criteria

1) Previously received ofatumumab or placebo in Trial Hx-CD20-403

2) Applicable only to patients on methotrexate therapy at time of screening:
   - Treatment with a stable dose of methotrexate (7.5 – 25 mg/week, p.o., i.m., and/or s.c.) ≥ 4 weeks prior to visit 2_A and
   - Treatment with methotrexate ≥ 12 weeks prior to Visit 2_A, with possible interruption of treatment of maximum two weeks in total, in the period 5-12 weeks from baseline.

3) Applicable only to patients on oral corticosteroids therapy at time of screening:
   - Treatment with a stable dose of oral corticosteroids (≤ 10 mg/day prednisolone or equivalent) ≥ 4 weeks prior to visit 2_A

4) Active disease at the time of screening as defined as:
   - ≥ 3 swollen joints (of 28 joints assessed) and
   - ≥ 3 tender joints (of 28 joints assessed) and
   - DAS28≥3.2 (based on ESR)

   Note:

   To accommodate for fluctuations in joint swelling, tenderness and ESR the following will be allowed:

   a) Visit 1:
   The joint swelling count, tenderness count, and ESR may be repeated once during the 14 day period from the screening visit to the baseline visit (Visit 2_A), to meet requirements for inclusion criterion 4. All three assessments should be reassessed regardless of which of the three assessments fails inclusion criterion 4. If one of the three criteria after the reassessment still do not fulfill inclusion criterion 4, Visit 1 may be repeated several times within the recruitment period.

   b) Visit 2_A:
   The swollen and tender joints should be reassessed at baseline (Visit 2_A). Where possible, joint count reassessment should be performed at the baseline visit (Visit 2_A); if this is not possible it can be performed ≤ 3 days prior to Visit 2_A. If the patient does not have ≥ 3 swollen and tender joints at Visit 2_A, this visit may be repeated once within the following 4 week period.

If joint counts at the repeated Visit 2 $_A$ (if applicable) are still not meeting eligibility criterion no. 4, then Visit 1 and Visit 2 $_A$ may be repeated several times within the recruitment period.

5) Following receipt of verbal and written information about the study, the patient must provide signed informed consent before any study related activity is carried out.

5.2 Exclusion Criteria

1) Use of DMARDs other than methotrexate < 4 weeks prior to Visit 2 $_A$.
Specifically for leflunomide treatment: use of leflunomide < 12 weeks prior to Visit 2 $_A$, unless the patient has completed peroral cholestyramine treatment for washout, according to locally accepted clinical practices.

2) Exposure to other cell depleting therapy, including investigational compounds (e.g. anti-CD11a, anti-CD19, anti-CD20, anti-CD22, anti-BLyS/BAFF, anti-CD3, anti-CD4, anti-CD5, CAMPATH) < 6 months prior to Visit 2 $_A$.

3) Exposure to etanercept or anakinra < 4 weeks, infliximab or adalimumab < 8 weeks, or abatacept < 12 weeks prior to Visit 2 $_A$

4) Received any of the following treatments < 4 weeks prior to Visit 2 $_A$:
    - Anti-cancer therapy (e.g. alkylating agents, anti-metabolites, purine analogues, monoclonal antibodies)
    - Oral corticosteroids > 10 mg prednisolone per day or equivalent
    - Intra-articular, i.m. or i.v. corticosteorids
    - Live/attenuated vaccinations
    - Cyclosporine
    - Azathioprine
    - Penicillamine
    - Mycophenolate Mufetil

5) Exposure to cyclophosphamide, nitrogen mustard, chlorambucil or other alkylating agents < 5 years prior to screening

6) Exposure to gold therapy <12 weeks prior to Visit 2 $_A$

7) Exposure to i.v. immunogammaglobulins < 24 weeks prior to Visit 2 $_A$

8) Active autoimmune disease (other than RA and RA-associated secondary diseases) requiring immunosuppressive therapy

9) Diagnosis of fibromyalgia or other chronic pain syndrome requiring daily narcotic treatment

10) History of infected joint prosthesis within five years before Visit 1 and infected native joints within one year before Visit 1

11) Past or current malignancy, except for:
    - Cervical carcinoma Stage 1B or less
    - Non-invasive basal cell and squamous cell skin carcinoma
    - Malignant melanoma with a complete response of a duration of > 10 years
    - Other cancer diagnoses with a complete response of a duration of > 5 years

12) Chronic or ongoing active infectious disease requiring systemic treatment such as, but not limited to, chronic renal infection, chronic chest infection with bronchiectasis, tuberculosis and active hepatitis B and C.

    Note i: Subjects with a screening chest X-ray suggestive of TB without documentation of adequate TB treatment (see Section 8.12) will be excluded; ii) Screening for latent TB infection using intradermal injection of tuberculin (e.g. the Mantoux test or equivalent) should be conducted according to local guidelines (see Section 8.12). Subjects with a positive skin tuberculin test should be excluded if the investigator judges the patient to be at risk of latent TB infection.

13) Clinically significant cardiac disease including unstable angina, acute myocardial infarction within six months from Visit 1, congestive heart failure, and arrhythmia requiring therapy, with the exception of extra systoles or minor conduction abnormalities

14) Significant concurrent, uncontrolled medical condition including, but not limited to, renal, hepatic, hematological, gastrointestinal, endocrine, pulmonary, neurological, cerebral or psychiatric disease

15) History of significant cerebrovascular disease

16) Known or suspected HIV positive

17) A circulating IgG level <lower limit of normal (as assessed by the central laboratory) at screening

18) Screening laboratory values:
    -Hemoglobin < 6.2 mmol/L (9.9 g/dL)
    - Neutrophils < 2 x 10^9/ L
    - Platelets < 100 x 10^9/ L

-CD4 (or CD4/CD8 ratio) < LLN
- S-ALAT > 3 times the upper limit of normal
- S-ALP > two times the upper limit of normal
-S-AST > 1.5 x ULN
- S-creatinine > 133 µmol/L (1.5 mg/dL)

19) Positive serology for hepatitis B (HB) defined as:
    - Positive test for HBsAg and/or
    - Positive test for anti-HBc and anti-HBs

    Patients with documented vaccination against Hepatitis B (primary and secondary immunization and booster) will be considered negative.

20) Positive plasma or white cell JC virus (JCV) PCR (either compartment)

21) Known hypersensitivity to components of the investigational medicinal product

22) Patients who have received treatment with any non-marketed drug substance within 4 weeks prior to Visit 1 (screening)

23) Current participation in any other interventional clinical study, except for study Hx-CD20-403.

24) Patients known or suspected of not being able to comply with a study protocol (e.g. due to alcoholism, drug dependency or psychological disorder)

25) Breast feeding women or women with a positive pregnancy test at Visit 1 (screening)

26) Women of childbearing potential not willing to use adequate contraception during study and one year after last dose of ofatumumab. Adequate contraception is defined as oral hormonal birth control, implants of levonorgestrel, estrogenic vaginal ring, percutaneous contraceptive patches, intrauterine device, and injection of prolonged gestagen. For patients in the USA the use of a double barrier method is also considered adequate (condom or occlusive cap plus spermicidal agent).

27) RA functional class IV.

28) Positive test for Hepatitis C:

    Note: i) Subjects who are positive for Hepatitis C antibody **and** have a positive or indeterminable result of the confirmative Hepatitis C analysis are excluded from participation in the trial. ii) Subjects who are positive for Hepatitis C antibody but negative in the Hepatitis C confirmatory assay are eligible to participate.

5.3 Withdrawal Criteria

Patients will not be substituted if withdrawn from the trial.

5.3.1 Withdrawal from Treatment

A patient should be withdrawn from further treatment with study drug if at any time:

- It is the wish of the patient (or their legally acceptable representative) for any reason
- The investigator judges it necessary due to medical reasons
- The patient becomes pregnant
- The patient receives prohibited therapy or procedures during the Treatment Period (see Section 7.4.2)
- If the patient at any time during the trial enters another interventional clinical study
- The patient has an abnormal ECG finding judged by the investigator to be clinically relevant
- An adjustment in concomitant MTX is initiated except for decreases in dose and/or MTX withdrawal due to toxicity
- An adjustment in concomitant corticosteroids is initiated except for decreases in dose and/or corticosteroid withdrawal
- If the patient has abnormal liver chemistry finding as defined in Section 9.5.2
- Positive hepatitis B (patients with documented vaccination against Hepatitis B (primary and secondary immunization and booster) will be considered negative)
- The patient develops symptoms of Progressive Multifocal Leukoencephalopathy (PML)
- The patient does not respond to the treatment by obtaining a EULAR response or a 20% reduction in swollen and tender joint counts within 24 weeks after a treatment course
- Levels of IgG < LLN (according to central laboratory normal range) at any time during the trial.

If the patient is withdrawn from further ofatumumab treatment, the patient should proceed to the Follow-up Period (Section 6.2.3). The first follow-up visit should take place 12 weeks (±2 weeks) after the latest visit attended in the Treatment Period.

If a patient does not return for a scheduled visit, every effort should be made to contact the patient. In all circumstances, every effort should be made to document patient outcome.

All study drug related adverse events should be followed until resolved or until the Investigator assesses them as chronic or stable.

The Investigator, in consultation with the patient, will decide the future course of treatment.

5.3.2 Withdrawal from Trial

A patient should be withdrawn from the study at any time if:

- It is the wish of the patient (or their legally acceptable representative) for any reason
- The investigator judges it necessary due to medical reasons.
- The patient initiates treatment with other B-cell suppressive treatment (e.g., other anti-CD20 antibodies, cyclophosphamide, or azathioprine)

5.3.3 Withdrawal from Safety Follow-up

A patient should be withdrawn from the Follow-up if at any time:

- It is the wish of the patient (or their legally acceptable representative) for any reason
- The investigator judges it necessary due to medical reasons
- The patient initiates treatment with other B-cell suppressive treatment (e.g. other anti-CD20 antibodies, cyclophosphamide, azathioprine etc.)
- The patient enters another interventional clinical trial and/ or receives treatment with any non-marketed drug substance or experimental therapy.

6 Trial Design

6.1 Overall Design

This is a 3-year open-label, phase II, extension trial. Patients who previously participated in Trial Hx-CD20-403 and who fulfill the eligibility criteria for this trial (GEN413) will be offered participation.

All patients will initiate at least one treatment course of ofatumumab. One treatment course consists of two infusions of ofatumumab 700 mg (Infusions 1 and 2) separated by 2 weeks. The remaining treatment courses will be given at individualized time intervals when a clinical response has been achieved following the previous treatment course and a subsequent worsening in disease activity has been observed. The interval between each treatment course will be at least 16 weeks from Infusion 1 in the previous treatment course irrespective of progression in disease activity. The last treatment course should be planned to be initiated no later than week 130 after baseline (Visit 2_A). The patients can receive a maximum of 9 treatment cycles.

After completing the Treatment Period or after withdrawing from the Treatment Period prematurely patients will be followed every 12 weeks (Follow-up Period) until $CD19^+$ cells have returned to baseline or normal levels (according to central laboratory normal ranges). The B-cell baseline refers to the B-cell value measured prior to the administration of trial medication at Visit 2 during the Hx-CD20-403 trial. For patients withdrawn from treatment period prematurely due to IgG levels falling below normal range, IgG levels will also be followed. In cases where the CD19+ cells have returned to baseline or normal levels and the IgG level is still low, the patients will continue to be followed for IgG levels until normalization.

The patients can attend unscheduled visits any time during the trial. An unscheduled visit will include one or more of the assessments listed for a Visit 7, however, the actual assessments done for the individual patient will be selected at the discretion of the investigator.

Patients on methotrexate therapy at time of screening will continue receiving stable doses of methotrexate (7.5 - 25 mg/week, p.o., i.m., and/or s.c.) throughout the trial. Adjustments in concomitant MTX are not allowed except for decreases in dose and/or MTX withdrawal due to toxicity.

In addition continued use of oral corticosteroids $\leq$ 10 mg/day prednisolone or equivalent will be allowed if the dosage was stable for at least four weeks prior to screening. Adjustments in concomitant corticosteroid are not allowed except for decreases in dose and/or corticosteroid withdrawal.

Pain management in the form of analgesics and NSAIDs will also be allowed throughout the trial. Furthermore, one intra-articular/intra-muscular corticosteroid injection in one single joint or muscle is allowed once every 6 months throughout the Treatment Period.

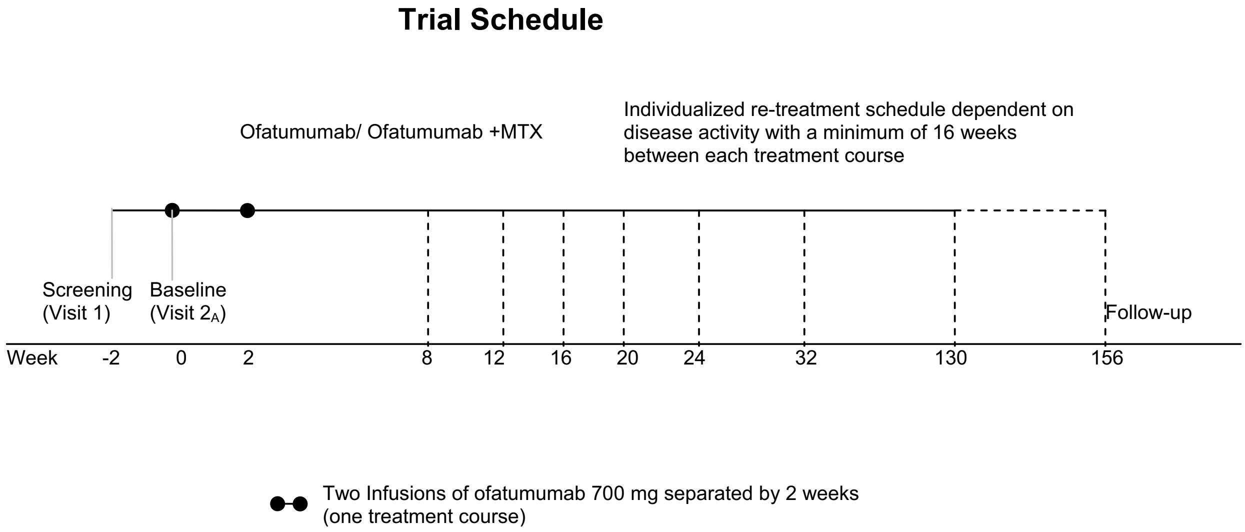

Figure 2: Trial Schedule

First treatment course

Patients will receive ofatumumab 700 mg at Visits 2 and 3 (Infusion 1 and 2).

Second and subsequent treatment courses

After the first treatment course, a patient will be eligible to receive further ofatumumab treatment courses, with last treatment course initiated no later than Week 130, when **all** of the **three** following criteria have been met:

1. The patient has achieved an efficacy response to ofatumumab at least once within 24 weeks from the last ofatumumab re-treatment course, defined as

   - at least a moderate EULAR response (compared to baseline value, i.e., Visit 2_A)

   OR

   - ≥20% improvement in both swollen <u>and</u> tender joint counts (compared to baseline value, i.e., Visit 2_A)

2. Following this response the patient subsequently shows a worsening in disease activity at anytime since the last ofatumumab treatment course defined as:

   - A sufficiently high disease activity defined as DAS28 ≥ 3.2

   AND

   - An increase in disease activity defined as:

     - a ≥0.6 increase in DAS28 compared to lowest DAS28 attained in the current treatment cycle

     And/Or

- a ≥20% worsening in both tender and swollen joint counts compared to lowest counts attained in the current treatment cycle.

3. The patient has adequate levels of IgG defined as IgG ≥ LLN (Lower Limit of Normal reference range).

Note: A moderate EULAR response is only considered to be valid if obtained at least at 6 weeks after Infusion 2 to allow washout of any positive effects of corticosteroid treatment from the pre-infusion treatment. Similarly the response is only valid if obtained at least 4 weeks after an i.m/i.a injection of corticosteroid.

Patients who do not obtain at least a moderate EULAR response or ≥20% improvement in both swollen and tender joint counts (compared to baseline values, i.e. Visit 2_A) within 24 weeks will be withdrawn from further ofatumumab treatment and proceed to the Follow-up Period.

When re-treatment criteria are fulfilled a treatment course should be planned as soon as possible, and no later than 28 days after re-treatment criteria are fulfilled

6.2 Schedule of Events

For more information about the visits please refer to the Flow Chart included in Section 2.

6.2.1 Screening (Visit 1) & Baseline (Visit 2_A)

In the Hx-CD20-403 protocol it states that the patients' B-cells should be followed until normalized (CD19+ cells equal to or above lower limit of normal or CD19+ cells are at the same or above the level measured at baseline). However since: 1) the GEN413 trial is considered as an extension trial of the Hx-CD20-403 trial and 2) as these patients' B-cells will also be monitored in the GEN413 trial, we will allow patients from Hx-CD20-403 to enter the GEN413 trial irrespective of their present B-cell status.

Furthermore, for safety reasons an exclusion criterion has been incorporated in to the GEN413 trial to ensure that only patients with normal IgG levels can be enrolled.

It may take several months (or even years) before those patients who received active treatment during the Hx-CD20-403 trial to eventually reach normalized B-cell values (CD19+ cells equal to or above lower limit of normal level or CD19+ cells are at the same or above the level measured at baseline). For the purpose of the GEN413 trial, B-cell baseline is defined as the B-cell value measured prior to the administration of trial medication at Visit 2 during the Hx-CD20-403 trial. Although the baseline B-cell levels for those patients who received active treatment may remain decreased, the Investigator may judge that an additional ofatumumab treatment is beneficial. Patients in the Hx-CD20-403 trial have an opportunity to receive additional treatments with ofatumumab by entering the open-label extension trial GEN413. Thus, patients from trial Hx-CD20-403 with suppressed B-cells will be allowed to be screened for GEN413 trial eligibility whilst remaining in B-cell follow-up for the Hx-CD20-403 trial. Patients that fulfill the screening criteria for GEN413 will proceed directly in to GEN413 and be immediately withdrawn from Hx-

CD20-403 follow-up. Those patients that fail the screening in GEN413 will, however, continue with B-cell follow-up in Hx-CD20-403 until B-cell normalization. If a patient fails screening for GEN413 due to inclusion criteria 4, they can be re-screened in accordance with the GEN413 protocol.

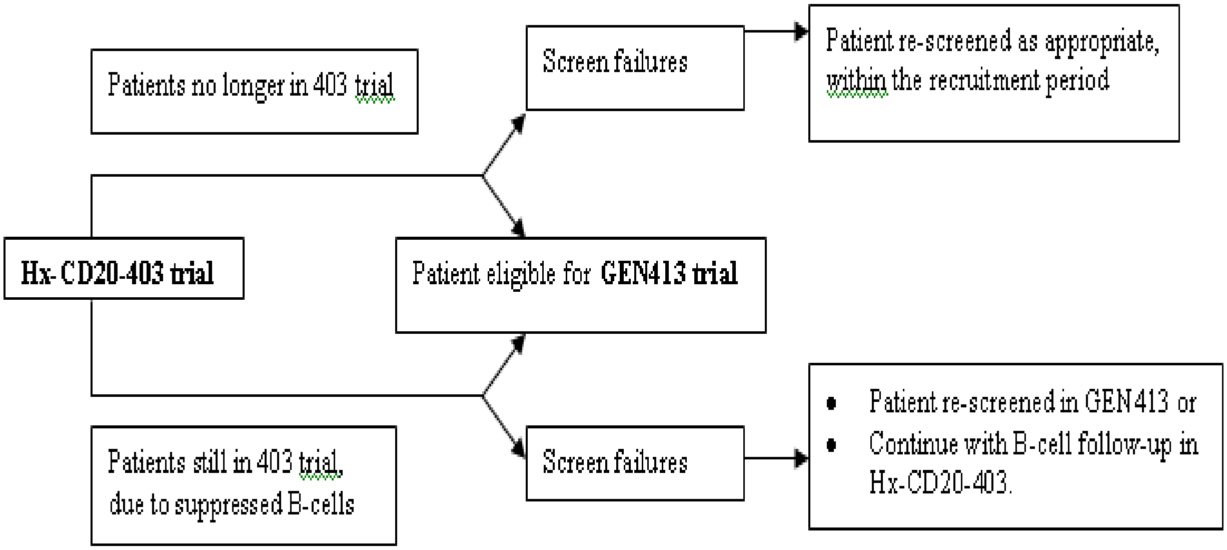

Figure 3: Entrance to the GEN413 trial

When an investigator identifies a patient suitable for screening and the patient has provided informed consent, the screening visit (Visit 1) can be initiated. All screening examinations must be performed within 2 weeks prior to the baseline visit (Visit 2_A). The only exception is the chest X-ray which, if already obtained as part of routine treatment ≤12 weeks prior to the screening visit, is not required. To accommodate for fluctuations in joint swelling, tenderness and ESR the below flexibility is allowed to meet requirements for inclusion criterion 4:

a) Visit 1:
The joint swelling count, joint tenderness count, and ESR may be repeated once during the 14 day period from the screening visit to the baseline visit (Visit 2_A), to meet requirements for inclusion criterion 4. All three assessments should be reassessed regardless of which of the three assessments that fails inclusion criterion 4. If one of the three criteria after the reassessment still do not fulfill inclusion criterion 4, Visit 1 may be repeated several times within the recruitment period.

b) Visit 2_A:
The swollen and tender joints should be reassessed at baseline (Visit 2_A). Where possible,

joint count reassessment should be performed at the baseline visit (Visit 2 $_A$); if this is not possible it can be performed ≤ 3 days prior to Visit 2 $_A$. If the patient does not have ≥ 3 swollen and tender joints at Visit 2 $_A$, this visit may be repeated once within the following 4 week period.
If joint counts at the repeated Visit 2 $_A$ (if applicable) are still not meeting eligibility criterion no. 4, then Visit 1 and Visit 2 $_A$ may be repeated several times within the recruitment period.

No other assessment can be repeated to meet the eligibility criteria.

Patients exposed to other B-cell depleting therapy (e.g. rituximab and ocrelizumab), abatacept or anti-TNF therapies have to undergo a wash-out prior to baseline (Visit 2 $_A$) and this can be initiated ahead of the screening visit (specified in Section 5.2). However, patient informed consent must be obtained prior to commencing the wash-out, if wash-out was initiated due to the planning of entering this trial.

For patients failing screening, the reason for not entering the study should be provided in the Screening and Allocation Log.

A patient can only be considered for re-screening in-line with inclusion criterion 4 (re-screening of joint re-assessments). Patients who fail screening for any other reason can not be re-screened, and hence can not enter GEN413. Informed consent is not required for a re-screen.

Each patient from the screening phase who is willing to participate and is found eligible according to the inclusion and exclusion criteria will enter the treatment phase and receive a Patient Number that will follow the patient throughout the trial.

The Investigator must maintain an Identification List of all allocated patients at the site containing data to identify the patient's existence and for traceability purposes. This list will contain full name, date of birth, and other contact details such as national ID number or hospital number.

6.2.2 Treatment

The investigator must have evaluated patient's eligibility and signed the Screening Evaluation Form before any patient receives trial treatment.

The investigator must confirm the patient's eligibility in the CRF before the patient can be enrolled and receive trial treatment.

The initial and following treatment courses must be administered as explained in Section 6.1

6.2.3 Follow-up

Patients who have completed the Treatment Period or are withdrawn from treatment or withdrawn from the trial (Section 5.3 and Section 17) will be followed every 12 weeks until B-cells have returned to normal (according to central laboratory) or baseline levels,or for a maximum of 2 years

from the last scheduled visit in the treatment period. The B-cell baseline refers to the B-cell value measured prior to the administration of trial medication at Visit 2 during trial Hx-CD20-403. For patients withdrawn from treatment period prematurely due to IgG levels falling below normal range, IgG levels will also be followed until IgG levels have returned to normal (according to central laboratory) or baseline levels,or for a maximum of 2 years from the last scheduled visit in the treatment period.

Furthermore neurological examinations and plasma/white cell JCV PCR testing will be done and the patients will be followed for SAEs and concomitant RA medication.

If the patient initiates treatment with other B-cell suppressive treatment (e.g., other anti-CD20 antibodies, cyclophosphamide, or azathioprine), at any time during the trial including the Follow-up Period, or enters another interventional trial while in the Follow-Up Period , all patient related study activities according to this trial protocol should be terminated. Furthermore, the investigator should document the date of initiation of other B-cell suppressive treatment or date of initiation of other trial and complete the End of Study Conclusion Form.

End of study is defined as date of the last patient attending the last visit, hence this will be when the last patient's B-cells have returned to baseline or normal levels (according to central laboratory) or for a maximum of 2 years from the last scheduled visit in the treatment period whichever occurs earlier.

6.3 Treatment after the End of the Study

The investigator is responsible for ensuring that consideration has been given to the post-study care of the patient's medical condition.

Note: GSK wil not provide any post study treatment with ofatumumab, and no compassionate use of ofatumumab for autoimmune indications is currently available for rheumatoid arthritis patients

6.4 Recruitment period

First Patient In: Jan 2008

Last Patient In expected: Jul 2009

The recruitment period is approximately 18 months

6.5 Number of Patients

A total of 264 patients participated in trial Hx-CD20-403, hence the maximum number of patients participating in trial GEN413 is 264.

See Section 10.10.

6.6 Treatment Allocation

Since this is an open-label single arm trial all the patients will initiate minimum 1 and maximum 9 treatment courses of ofatumumab, depending on the patient's response.

The Patient Numbers from Trial Hx-CD20-403 will be re-used in this Trial with a smaller modification. The first three digits (403) in the Patient Numbers in the Hx-CD20-403 trial will be replaced with the digits 413 to reflect the GEN413 trial. The last three digits will be unchanged; hence data in the two studies can be linked.

7 Treatment

7.1 Ofatumumab

7.1.1 Ofatumumab

Ofatumumab is a clear colorless liquid. Ofatumumab is a concentrate for solution intended for intravenous administration.

Ofatumumab is formulated at 20 mg/mL. Ofatumumab is supplied in 6 mL clear glass vials. Each vial contains 5 mL of ofatumumab (20 mg/mL), i.e. a total of 100 mg ofatumumab

Ofatumumab will be filtered using an inline filter (0.2 μm) during infusion.

7.1.2 Packaging and Labeling of Ofatumumab

Ofatumumab will be supplied to the sites/pharmacies in cartons, each carton containing 10 vials. Labeling will be in accordance with local law and trial requirements.

7.1.3 Storage of Ofatumumab

Ofatumumab should be stored in a safe and secure place in a refrigerator at 2-8°C, protected from light and it must not be frozen.

After ofatumumab has been diluted in sterile, pyrogen free, 0.9% NaCl it can be kept at room temperature and must be given to the patient within 24 hours. Exact time of dilution into 0.9% NaCl must be written on the label of the infusion bag.

Drug supplies must be kept in an appropriate restricted area, which may be accessed only by relevant site personnel/ pharmacist or a duly designated person. A log to document the temperature with daily readings must be kept.

If the temperature of the refrigerator is outside the limits of 2-8°C (35.6-46.4 °F) it should be noted in the temperature log. If the temperature is/ has been ≤ 0 °C or ≥ 10°C (≤ 32 °F or ≥ 50 °F) for more than 8 hours the local CRA should be contacted. The following information should be available: the study number, amount of ofatumumab and data on the temperature in the refrigerator, and for how long the temperature was outside the temperature limits. If a break down of the refrigerator occurs, ofatumumab should be transferred to another temperature controlled refrigerator immediately.

Ofatumumab must not be utilized after the expiry date printed on the carton label.

7.1.4 Drug Accountability and Compliance Check

The investigator must ensure that a designated person receives ofatumumab shipments from sponsor and that all such deliveries are:

- Recorded
- Handled and stored safely and properly
- Only dispensed to study patients according to the protocol
- Returned to sponsor if unused.

The investigator or designee must keep drug inventory and accountability logs. The inventory will include details of ofatumumab received and dispensed to the patient, batch and ID numbers. All unused vials must be kept and returned to sponsor after the reconciliation of delivery records with accountability logs by the CRA. After accountability of empty used vials has been performed by the CRA, these can be destroyed at site. Accountability must be made of any drug deliberately or accidentally destroyed. Discrepancies between the amount of received and dispensed drug must be reconciled.

Disposal of hazardous material, e.g., syringes, needles, etc. must conform to applicable local laws and regulations.

7.2 Dosage of Ofatumumab

Each patient will initiate two infusions of ofatumumab (Infusion 1 and 2) separated by 2 weeks (one treatment course) and further re-treatment courses will be administered according to Section 6.1 and will not exceed the number of re-treatment courses outlined in Table 1.

Table 1: Ofatumumab dosages

1st treatment course	2nd treatment course	3rd treatment course	4th treatment course	5th treatment course	6th treatment course	7th treatment course	8th treatment course	9th treatment course
2 x 700 mg	2 x 700 mg	2 x 700 mg	2 x 700 mg	2 x 700 mg	2 x 700 mg	2 x 700 mg	2 x 700 mg	2 x 700 mg

7.3 Handling of Ofatumumab

7.3.1 Pre-Medication

Before each infusion, all patients must receive the following pre-medication 30 min - 2 hours prior to start of treatment with ofatumumab. Data regarding pre-medication should be recorded in the CRF.

Table 2: Pre-medication requirements before each infusion of ofatumumab

Time prior to infusion	Medication	Dose	Administration
30 min – 2 h	Paracetamol (acetaminophen) or equivalent	1 g[a)]	p.o.
30 min – 2 h (i.v.)<br>alternatively<br>1 h – 2 h (p.o)	Antihistamine (cetirizine or equivalent)	10 mg[a)]	i.v.<br>alternatively<br>p.o.
30 min – 2 h	Glucocorticoid (prednisolone or equivalent)[b)]	100 mg	i.v.

Table notes:
a) If an equivalent drug is used dose range should be according to locally accepted practice
b) Prednisolone 100 mg corresponds to methylprednisolone 80 mg and hydrocortisone 400 mg

7.3.2 Preparation of Ofatumumab

Preparation of infusion bags should be done on the day of planned infusion. The infusion will be prepared as a 1000 mL dilution of ofatumumab in sterile, pyrogen free, 0.9% NaCl. Seven vials equivalent to 35 mL are required to prepare the solution for infusion. 35 mL of the sterile, pyrogen free, 0.9% NaCl should be removed from the infusion bag prior to mixing with ofatumumab. A detailed description with instructions for the preparation of ofatumumab will be supplied to each pharmacy/centre.

Table 3: Ofatumumab infusion preparation

Dose ofatumumab	Number of vials	Volume of ofatumumab
700 mg	7	35 mL

Preparation of ofatumumab infusion bags should be done on the day of planned infusion. The prepared infusion bag should be kept at room temperature for approximately one hour prior to infusion. Infusion should start no later than 12 hours after completed preparation to ensure completed infusion no later than 24 hour after preparation (due to expiry of the diluted ofatumumab).

7.3.3 Treatment Schedule

Ofatumumab will be administered as an intravenous (i.v.) infusion. The initial rate of the infusion will be 12 mL/hour for Infusion 1 and 25 mL/hour for Infusion 2 during each treatment course. During infusion, the rate will be doubled every 30 minutes to a maximum of 400 mL/hour. Thereafter, the rate will be increased by 200 mL/hour every 30 minutes until 800 mL/hour is reached. Duration of the infusion will be approximately 4 and 3½ hours for Infusion 1 and 2, respectively, if this schedule is adhered to.

Table 4: Infusion rate

Infusion 1 of each treatment course		Infusion 2 of each treatment course	
Time	mL/hour	Time	mL/hour
0 – 30 minutes	12	0 – 30 minutes	25
31 – 60 minutes	25	31 – 60 minutes	50
61 – 90 minutes	50	61 – 90 minutes	100
91 – 120 minutes	100	91 – 120 minutes	200
121- 150 minutes	200	121- 150 minutes	400
151 – 180 minutes	400	151 – 180 minutes	600
181 – 210 minutes	600	+ 180 minutes	800
+ 210 minutes	800		

The infusion rate may be adjusted if any study drug related AEs occur. A detailed description with instructions for the administration of ofatumumab will be supplied to each center.

The actual administration time should be recorded including any pauses and changes in the infusion rates.

7.3.4 Administration of Ofatumumab

Ofatumumab must be administered by intravenous (i.v.) infusion through an in-line filter and through a patent intravenous catheter (i.v. cannula) into a vein in the arm by an infusion pump. Please note that the injection site cannot be used for blood sampling.

The patient should be carefully observed (including blood pressure, temperature, heart rate, and adverse events) during infusion and 2 hours following infusion 1 in each treatment course and 1 hour for infusion 2 in each treatment course. The study staff at the clinic must be prepared to intervene if an infusion reaction should occur. Special caution should be taken during the first infusion for each patient and during the infusion of a patient who did not tolerate a previous infusion well.

Following the infusion, the infusion line should be flushed with sterile, pyrogen free, 0.9% NaCl. It should be noted that all infusions must be completed or pre-maturely stopped within 24 hours from the preparation of ofatumumab. A detailed description with instructions for administration of ofatumumab will be supplied to each center.

7.3.4.1 Measurements during infusion

During infusion the patient should be monitored closely and appropriate measurements should be performed whenever judged necessary. As a minimum vital signs and PK sampling must be performed as per Table 5.

Table 5: Measurements and PK sampling during infusion

Time	Vital Signs: Blood pressure, temperature and heart rate	PK sampling
Before infusion	X	X
Every hour during infusion	X	
End of infusion	X	X[a)]
+1 hour	X	
+2 hours[b)]	X	
After end of infusion	X[c)]	

Table notes:

a) PK sampling at "end of infusion" is immediately after the infusion line has been flushed with sterile, pyrogen free 0.9% NaCl.

b) Only applicable after Infusion 1 in each treatment course. Otherwise the observation period is one hour.

c) If the patients remain two hours after infusion 1 and one hour after infusion 2 due to trial drug induced toxicity, vital signs will be measured every hour until the patient leaves the clinic.

The following data should be documented:

- Start and end time for observations / measurements
- Infusion rate including all pauses and changes in infusion rate
- Pre-medication and medication given during infusion including information of dose
- All vital signs during infusion
- Adverse events.

7.3.4.2 Handling of Infusion Related Reactions

Previously observed infusion related AEs are described in the current Investigator´s Brochure and Section 3.2.5. In case of adverse events during infusion the patients must be treated according to the investigator's judgment and best clinical practice. Guidance on the management of infusion related reactions may also be found in the IMP Administration Manual.

Infusion related Adverse Events may lead to a prolonged infusion time. Overnight stay at the hospital due to slow infusion rate shall not be considered a Serious Adverse Event (SAE).

Interruption, restart and increasing the rate of the infusion depending on the severity of the adverse event must be according to the description below and at the investigator's discretion. Increase of the infusion rate after an interruption must not exceed the schedule in Table 4 (i.e. not more than doubled rate and no earlier than every 30 minutes).

Please see description below:

Adverse Events Grade 1 and 2

If the investigator judges the AE to be related to the infusion, the infusion must be paused.
When the patient's condition is stable, the infusion can be restarted according to the judgment of the investigator.

Upon restart, the infusion rate must be half of the infusion rate applied before the pause. If, however, the infusion rate was 12 mL/hour before the pause, the infusion should be restarted at 12 mL/hour. Hereafter, the infusion rate may be increased at the investigator's discretion in the manner described in Section 7.3.3, Table 4 (i.e. not more than doubled and no earlier than every 30 minutes).

Adverse Events Grade ≥ 3

If the investigator judges the AE to be related to the infusion, the infusion must be paused. The patient should be observed for 2 hours. If the AE remains ≥ grade 3 the patient should be withdrawn from further treatment. If the AE decreases to < Grade 3 the investigator must judge if the infusion should be restarted.

Upon restart, the infusion rate must be 12 mL/hour (Infusion 1 in each treatment course) or 25 mL/hour (Infusion 2 in each treatment course), but may subsequently be increased according to the judgment of the investigator, in the manner described in Table 4 (i.e. not more than doubled and no earlier than every 30 minutes).

If the severity of the AE again increases to ≥ Grade 3, the described procedure can be repeated at the investigators judgment. Should the severity of the AE increases to ≥ Grade 3 for a third time the patient must be withdrawn from treatment.

7.4 Concomitant Therapy

7.4.1 Therapy Allowed during Trial

Patients may receive their current medication for non-RA conditions.

With respect to current RA medication, the following will be allowed under the restrictions described below.

Methotrexate and Folic Acid

Patients on methotrexate therapy at time of screening should continue receiving stable doses of methotrexate (7.5 - 25 mg/week, p.o., i.m., and/or s.c.) throughout the trial. This dose should be identical to the dose received for the last four weeks prior to screening. During the study, adjustments in concomitant MTX is not allowed except for decreases in dose and/or MTX withdrawal due to toxicity.

The use of folic acid (dose according to locally accepted practice) will be at the discretion of the investigator.

Oral Corticosteroids

Continued use of oral corticosteroids ≤ 10 mg/day prednisolone or equivalent is allowed if the dosage was stable for at least four weeks prior to screening. Adjustments in concomitant corticosteroid are not allowed except for decreases in dose and/or steroid withdrawal.

NSAIDs

Use of NSAIDs is permitted (e.g. diclofenac, ibuprofen, ketoprofen, naproxen) in daily doses up to the maximum recommended according to locally accepted clinical practices. However, the patients should be advised not to take any NSAIDs within 12 hours prior to attending a trial visit.

Analgesics

Regular use of codeine, opium alkaloid, paracetamol/acetaminophen, propoxyphene, and tramadol are permitted in daily doses up to the maximum recommended according to locally accepted clinical practices. If the patient is not regularly using any analgesics, he/she may take the analgesics mentioned above as breakthrough pain management. However, the patients should be advised not to take any rescue analgesics within 12 hours prior to attending a trial visit.

Intra-articular or Intra-muscular Corticosteroids

One intra-articular (i.a.) or intra-muscular (i.m.) injection of corticosteroid (80 mg methyl prednisolone) or equivalent (see Table 6) in one joint/muscle every 6 month is allowed during the trial. However, the injected joint should only be considered (and marked) as tender and swollen for a time period of 12 weeks after the time of injection.

Table 6 Maximum doses for i.a. / i.m. corticosteroids

Generic Name	Brand Name (may be country specific)	Maximum Allowed Dose
Triamcinolone hexa-acetonide	Lederspan	20 mg
Triamcinolone acetonide	Kenalog	40 mg
Methyl prednisolone	Depo-Medrol	80 mg
Dexamethasone	Decadron	8 mg
Betamethasone	Celestona	12 mg
	Diprospan	14 mg

Responses (defined as at least a moderate EULAR response or ≥20% improvement in swollen and ≥20% improvement in tender joint counts (compared to Visit 2_A values)) obtained in the period from time of an i.a. or i.m corticosteroid injection and 4 weeks forwards are considered related to this injection and will not be regarded as a response to ofatumumab (will not trigger re-treatment).

7.4.2 Prohibited therapy and procedures during the study

The following medications and therapies will not be allowed as concomitant therapy from 4 weeks prior to screening and until completion of the study:

- Non- biological DMARDs, except methotrexate
- Biological RA therapies
- I.a., i.m. and i.v. corticosteroids (apart from i.a and i.m.. corticosteroid injections, as described in Section 7.4.1, and apart from i.v. glucocorticoid given as pre-medication, see Section 7.3.1)
- Anti-cancer therapy, immuno-therapy, or chemo-therapy
- Any non-marketed drug substance or experimental therapy
- Narcotics other than specified under analgesics in Section 7.4.1.

Exposure to biologic DMARDs during the study will not be allowed. For drug product specific wash-out period prior to Visit 2 $_A$ please refer to Section 5.2.

8 Trial Assessment

An overview of visits is given in the Flow Chart in Section 2.

8.1 Clinical Assessments

All trial assessments should be done prior to blood sampling and infusion unless otherwise stated.

8.2 Demographic

The demographic data (date of birth, race and sex) for patients to be enrolled in GEN413 is already available since this was collected in Trial Hx-CD20-403. However in order to clearly allow identification of the patients, date of birth will be collected in this trial (GEN413) as well.

8.3 Medical History

Any relevant prior or current diseases will be obtained by the site staff. Furthermore, the following information regarding RA will be recorded:

- RA functional class (I, II, III or IV) – see Appendix 1
- Previous RA treatment(s) including reason for discontinuation – Previous RA treatment will only be collected for the period between Visit 13 in Trial Hx-CD20-403 and Visit 1 in Trial GEN413
- Date of diagnosis of RA was collected in the Hx-CD20-403 and hence will not be collected in GEN413
- AEs having onset in Trial Hx-CD20-403 should not be recorded as medical history.

8.4 Height and Body Weight

The patients' heights have already been measured in Trial Hx-CD20-403 and hence will not be measured in Trial GEN413. Body weight (without overcoat and shoes) will be measured and recorded rounded to nearest kilogram.

8.5 Physical Examination

A physical examination will be performed by the physician. It will include but is not limited to: General appearance and the following body systems: Lymph nodes, mouth and throat, lungs, cardiovascular system, abdomen, extremities, musculo-skeletal system, neurological system and skin. All abnormal findings judged by the investigator to be clinically relevant, should be reported as AEs.

8.6 Neurological Examination

A neurological examination to detect any signs or symptoms consistent with a diagnosis of PML will be conducted as part of the physical examinations required by protocol. A questionnaire will be provided and should be filled in as part of the neurological examination. If any question is answered 'Yes' the investigator should contact the GSK medical officer or designee to discuss appropriate

management of the patient (Section 9.5.3). Any findings at Visit 1 should be reported as medical history and findings after Visit 2 $_A$ should be reported as adverse events and the PML reporting procedures followed (Section 9.5.3).

8.7 Electrocardiogram

An electrocardiograms (ECG) taken according to normal clinical practice will be performed.

An overall interpretation of the ECG will be performed by the investigator, or the investigator may delegate this task to a cardiologist, if appropriate. The date of the ECG and the overall interpretation of the ECG will be recorded. The ECG recordings will be kept in the patient's record.

If an ECG taken during the trial is abnormal and judged by the investigator to be clinically relevant, the patient should be withdrawn from further trial treatment and proceed to follow up.

8.8 Vital Signs

Vital signs including temperature, blood pressure, and heart rate will be measured and recorded by the site staff. Further information about measurements during infusion is described in Section 7.3.4.1.

Wherever possible, body temperature should be measured using the same method (e.g. an ear thermometer) and the position (e.g. sitting or lying) for measuring blood pressure and heart rate should be consistent throughout the study for each individual patient.

8.9 Adverse Events

The reporting of adverse events is described in Section 9.

8.10 Concomitant Medication

All concomitant medication taken during the study will be recorded. Any medication other than the IMP is considered concomitant medication (including background DMARD (if applicable) and medication given as pre-medication prior to infusions of ofatumumab).

8.11 Administration of IMP

A description of the procedures for administration of IMP is described in Section 6 (Treatment) and in the IMP handling Manual.

8.12 Tuberculosis

A chest X-ray will be performed to evaluate for tuberculosis. Posterior-anterior projections and, if indicated, lateral projections will be taken. An existing chest X-ray with the same projections, taken within 12 weeks prior to Visit 1, may be used instead. I the X-ray is inconclusive and if clinically warranted the x-ray should be repeated at screening.

Reading of the chest X-ray will be done by a radiologist. A copy of the radiologist's evaluation reports should be kept in the patient's record. The date of the chest X-ray and the overall interpretation of evaluation will be recorded.

An intradermal injection of tuberculin (e.g. Mantoux test or equivalent) should be performed according to local accepted practice to evaluate for latent TB infection. This skin test should be done at any time between screening and baseline (before first treatment). Subjects with documented BCG vaccination are exempted. Subjects with a positive skin tuberculin test will be excluded if the investigator judges the patient to be at risk of latent TB infection.

8.13 Clinical Efficacy Assessments

The clinical efficacy assessments must be performed prior to blood sampling and the administration of pre-medication and IMP.

8.14 Joint Assessments

A total of 28 joints should be assessed. Joints to be assessed for both the left and the right sides of the patient are:

Shoulder, elbow, wrist, metacarpophalangeal (first (thumb), second, third, fourth, fifth), proximal interphalangeal (thumb (interphalangeal), index, middle, ring, little) and knee.

Tender Joint Count:
A total of 28 joints should be assessed. Joints are classified as either tender or not tender.

Swollen Joint Count:
A total of 28 joints should be assessed. Joints are classified as either swollen or not swollen.

Replaced or Fused Joints:
Replaced or fused joint will not be included in joint evaluations. The reason for absence of the evaluations of those joints must be recorded.

Independent Joint Evaluator:
One or more independent assessors, who have documented experience in performing joint assessments, will be designated at each trial site to perform joint assessments. Preferably the independent assessor will perform all joint assessments for the same patient throughout the trial.

The independent joint assessor should have no other contact with the patient during the trial, must not be the treating physician (investigator), should not discuss the patient's clinical status with the patient during the joint assessment nor with other site personnel, and will not be permitted to review the patient's medical records, the CRF, nor any of the previous joint assessments.

The Independent Joint assessor will be provided with a Joint Assessment Form where joints that have been injected with Intra-articular corticosteroid Injections and/or joints that have been replaced

and/or fused will be indicated/premarked by the study nurse, since these joints should not be evaluated.

The independent joint assessor will perform only the joint assessment, and will not be involved in any other assessments in this trial.

8.15 Visual Analogue Scale (VAS) of Pain

A horizontal visual analogue scale (VAS) of 10 cm will be used to report the patient's level of joint pain. The scale ranges from ‘no pain’ to ‘severe pain’. The patient should be instructed to place a vertical mark on the line to indicate the severity of the pain.

The distance from the “no pain” end to the vertical line drawn by the patient is his/her joint pain score. The site staff or designee will measure this distance with a standard ruler scored in cm starting from “no pain” to the line marked by the patient and record that measurement (in cm). Patients should be instructed not to place a mark outside of the end markers. If this occurs, the data will be considered non-evaluable.

8.16 Patient and Physician VAS of Global Disease Assessment

Both the patient and the physician will use the horizontal VAS for overall assessment of the disease. The scale ranges from ‘extremely well’ to ‘extremely poor’. The evaluator and patient must complete the global assessment independently from each other.

The evaluator performing the global assessment cannot be the independent joint assessor. The results of the joint assessment performed by the independent joint assessor will be available to the physician assessing the patient's global disease.

It will be anchored at the 0 cm with "extremely well" and anchored at 10 cm end with "extremely poor". Using a standard ruler the site staff will measure and document the distance in cm from the left end marker to the point at which the patient's mark intersects the horizontal line. Patients should be instructed not to place a mark outside of the end markers. If this occurs, the data will be considered non-evaluable.

8.17 Patient Reported Outcomes

Questionnaires will be handed out to the patient for each assessment. If a patient is unable or refuses to complete a whole questionnaire the reason for this should then be ascertained and recorded.

8.18 Disability Index of the Health Assessment questionnaire (HAQ)

The functional status of the patient will be assessed by means of the Disability Index of the Stanford Health Assessment questionnaire (HAQ). This 20-question instrument assesses the degree of difficulty a person has in accomplishing tasks in eight functional areas (dressing, arising, eating, walking, hygiene, reaching, gripping, and errands and chores). Responses in each functional area are scored from 0 indicating no difficulty, to 3, indicating inability to perform a task in that area.

The index is calculated by the addition of the scores, then dividing this score by the total number of components answered.

8.19 Laboratory Assessments

Unless stated otherwise all samples should be drawn prior to infusion.

All laboratory samples will be sent to a central laboratory for analysis, except for urine pregnancy testing and ESR, which will be performed locally. The central laboratory may utilize subcontractors.

A detailed description (Investigator Manual) of the procedures for sampling, handling, storage, and shipment of the laboratory samples and all material such as test tubes and labels will be provided by the central laboratory. The Investigator Manual and the result reports will include all reference ranges.

8.20 Biochemistry, Hematology and Urine test

Blood and urine samples will be shipped to the central laboratory for immediate analysis of the following parameters:

- Biochemistry: Serum electrolytes (sodium, potassium, chloride, bicarbonate, calcium), creatinine, liver enzymes (AST/SGOT and ALT/SGPT), alkaline phosphatase (ALP), total protein, albumin, total bilirubin, uric acid, gamma glutaryl-transferase (GGT), alkaline phosphatase (ALP), blood urea nitrogen (BUN), lactic dehydrogenase (LDH), creatinine phosphokinase (CPK)
- Hematology: Hemoglobin, red blood cell (RBC) count, hematocrit, platelet count, and white blood cells (WBC) with differential count
- Urine test: Protein, glucose, ketones, blood and WBC.

Date of sampling will be recorded.

8.21 Pregnancy Test

For women of childbearing potential pregnancy testing will be performed. Serum samples will be drawn at screening and urine pregnancy testing should be performed at every clinic visit during the treatment cycle. If a visit is not scheduled, home urine testing must be performed.

Serum samples are shipped to the central laboratory for immediate analysis of Human Chorionic Gonadotrophine (HCG).

Women are considered of childbearing potential unless they have been hysterectomized, or have undergone tubal ligation at least one year prior to screening, or have been postmenopausal for at least one year.

All remaining pregnancy tests (including tests during Follow-up at week 12 and 24 from the last scheduled visit in the Treatment Period) will be based on urine sampling and will be analyzed locally. A negative urine pregnancy test should be obtained and documented. If the urine pregnancy test is positive a serum-HCG pregnancy test should be obtained. Treatment with ofatumumab can only be re-commenced if results of the serum pregnancy test are negative.

.

The date of sampling and outcome will be noted. If sampling for pregnancy test is not applicable, the reason for this will be documented.

8.22 IgA, IgG, IgM

Serum samples for measurement of IgA, IgG and IgM will be drawn and shipped to the central laboratory.

The date of sampling will be documented.

8.23 Flow Cytometry

Blood samples will be drawn and shipped to the central laboratory for analysis of $CD19^+$, $CD3^+$, $CD4^+$, and $CD8^+$ cells.

Only $CD19^+$ should be analyzed during the Follow Up Period

The date of sampling will be documented.

8.24 Plasma/white cell JCV PCR

Samples will be drawn and shipped to the central laboratory for analysis of plasma/white cell JC virus (JCV).

The date of serum sampling will be documented.

8.25 Host Immune Response (HAHA)

Serum samples will be drawn and frozen prior to shipment to the central laboratory for further frozen storage and analysis of Human Anti-Human Antibodies (HAHA).

The date of serum sampling will be documented.

8.26 Pharmacokinetics (PK)

Plasma samples will be drawn at the Visits marked in the Flow Charts in Section 2, and frozen prior to shipment to the central laboratory for further frozen storage and analysis of concentrations of ofatumumab.

Blood samples will be drawn for analysis of ofatumumab concentration at each visit. At visits where infusions will take place a more extended PK sampling will be done (see Table 5).

The date and time of plasma sampling will be recorded.

8.27 Hepatitis B and C

Patients will be evaluated for serologic evidence of Hepatitis B (HB) infection based on the results of testing for HBsAg, anti-HBc and anti-HBs antibodies as follows:

- Patients positive for HBsAg are excluded
- Patients negative for HBsAg but positive for both anti-HBc and anti-HBs antibodies are eligible
- Patients negative for HBsAg and anti-HBc antibody but positive for anti-HBs antibody are eligible
- Patients negative for HBsAg and anti-HBs antibody but positive for anti-HBc antibody will require clarification of their status by testing for HBV DNA which if positive will exclude the patient

Patients that have documented vaccination against hepatitis B (primary and secondary immunization and booster) will be considered negative.

A blood sample will be collected and analyzed for Hepatitis C antibodies at screening, and every 24 weeks during follow-up for a maximum of 2 years from the last scheduled visit in the Treatment Period. If the result is positive the viral load for Hepatitis C will be analyzed in another blood sample by a confirmatory assay. Both blood samples will be taken on the same day but will be stored and shipped ambient or frozen, respectively.

8.28 Biomarkers

Biomarkers which characterize disease activity, immune status, and transcriptional profiles will include, but are not limited to, the following: Rheumatoid factor (RF), Anti-Cyclic Citrullinated Peptide antibody (Anti-CCP), B-Lymphocyte Stimulator (BLyS), and IL-6. Samples will be taken at visits indicated in the Flow Chart in Section 2 and may be assayed on arrival or stored at the central laboratory prior to analysis. Samples will be stored securely and may be kept for up to 15 years after the last patient completes the study or the sponsor or the sponsor may destroy the samples sooner.

The date of sampling will be documented.

8.29 Transcriptomics

Whole blood will be collected for transcriptomic analysis of mRNA at the visits indicated in the Flow Chart in Section 2. Samples will be frozen prior to shipment to the central laboratory for further frozen storage and analysis. Samples will be stored securely and may be kept for up to 15 years after the last patient completes the study or the sponsor or the sponsor may destroy the samples sooner.

The date of sampling will be documented.

8.30 Pharmacogenetics

Information regarding pharmacogenetic (PGx) research is included in Appendix 2. The IEC/IRB and, where required, the applicable regulatory agency must approve the PGx assessments before these can be conducted at the site. The approval(s) must be in writing and will clearly specify approval of the PGx assessments (i.e., approval of Appendix 2). In some cases, approval of the PGx assessments can occur after approval is obtained for the rest of the study. If so, then the written approval will clearly indicate approval of the PGx assessments is being deferred and in most cases, the study, except for PGx assessments, can be initiated. When PGx assessments will not be approved, then the approval for the rest of the study will clearly indicate this and therefore, PGx assessments will not be conducted.

8.31 CRP and ESR

A blood sample will be taken at screening (Visit 1) to determine eligibility with respect to ESR. Furthermore, blood samples will be taken during the study for determinations of CRP and ESR.

The dates of sampling will be recorded.

Blood samples for ESR will be measured immediately at trial site using a Becton Dickinson Seditainer. Blood samples for CRP will be sent to the central laboratory for analysis.

The date of sampling and results will be documented.

9 Adverse Events

9.1 Definition of Adverse Events

An Adverse Event (AE) is any untoward medical occurrence in a patient administered a pharmaceutical product and which does not necessarily have a causal relationship with this treatment.

Adverse events include the following:

- All suspected Adverse Drug Reactions (ADR)
- All reactions from medication overdose, abuse, withdrawal, sensitivity, or toxicity
- Apparently unrelated illnesses, including the worsening of a pre-existing illness
- Injury or accidents. Note that if a medical condition is known to have caused the injury or accident, the medical condition and the accident should be reported as two separate AEs
- Abnormalities in physiological testing or physical examination findings that require clinical intervention or further investigation
- Laboratory abnormalities that require clinical intervention or further investigation unless they are associated with an already reported clinical event.

9.1.1 Pre-existing Conditions

All Adverse Events that had onset in Trial Hx-CD20-403 and Serious Adverse Events that are related to IMP and occurred after end of trial Hx-CD20-403 will be related to Trial Hx-CD20-403 and should hence not be reported in Trial GEN413.

In this study, a pre-existing condition (i.e., a disorder present before the AE reporting period started and noted on the medical history/physical examination form) should not be reported as an AE unless the condition worsens or episodes increase in frequency during the AE reporting period.

9.1.2 B-cell depletion

B-cell depletion due to ofatumumab treatment should not be reported as an AE.

9.1.3 Study Disease

Signs and symptoms, which according to the investigator are expected and well known consequences of the RA, both in intensity, and frequency, should not be reported as AEs unless they meet any of the specified seriousness criteria.

9.1.4 Procedures

Diagnostic and therapeutic non-invasive and invasive procedures, such as surgery, should not be reported as AEs. A medical condition for which an unscheduled procedure was performed, should

however be reported if it meets the definition of an AE. For example, an acute appendicitis should be reported as the AE and not the appendectomy.

9.1.5 Adverse Event Reporting

The Investigator must report all directly observed AEs and all AEs spontaneously reported by the patient. A general type of question should be used similar to" Do you have any health problems? "or" Have you had any health problems since your last visit?"

All AEs that occur in patients during the AE reporting period must be reported, whether or not the event is treatment related.

The AE reporting period (for SAE reporting period, see Section 9.3.2) begins from first treatment cycle (Visit 2/Day 0) until the end of the treatment Period. Any signs or symptoms occurring between Screening/Visit 1 and Visit 2 should be recorded as Medical History. During the Follow-up Period where the patient's B-cells, IgA, IgG and IgM levels are followed, only AEs that meet one or more of the serious criteria (see Section 9.3.1) will be reported to sponsor. The Serious Adverse Event Form should be completed.

9.1.6 Pregnancy

Any pregnancy that occurs during study participation must be reported. To ensure patient safety, each pregnancy must be reported to sponsor within 2 weeks of learning of its occurrence. The pregnancy must be followed up to determine outcome (including premature termination) and status of mother and child. Pregnancy complications and elective terminations for medical reasons must be reported as an AE or SAE. Spontaneous abortions must be reported as an SAE.

Any SAE occurring in association with a pregnancy brought to the investigator's attention after the patient has completed the study and considered by the investigator as possibly related to the investigational product, must be promptly reported to sponsor.

In addition, the investigator must attempt to collect pregnancy information on any female partners of male study patients who become pregnant while the patient is enrolled in the study. Pregnancy information must be reported to sponsor as described above.

9.2 Reporting Instructions

9.2.1 Recording Instructions

All AEs are to be recorded. If the AE is judged by the investigator to meet the criteria for a SAE, the SAE form should be completed.

9.2.2 Diagnosis

Diagnosis should be recorded if available. If no diagnosis is available each sign and symptom should be recorded as individual AEs.

9.2.3 Intensity

The Investigator will use the NCI CTCAE version 3.0 to describe the maximum severity of the AE (see Attachment 2).

The grade assigned by the investigator should be the most severe, which occurred during the AE period.

9.2.4 Relationship to Study Drug

The Investigator must assess the AE as either Related (possible/probable) or Not Related. If relationship changes over time the last judgment by the investigator should be reported. Relatedness has to be assessed and reported from first time the AE is being reported.

9.2.5 Outcome

Outcome of the AE must be judged by investigator by the following terms:

- Recovered
- Recovered with sequelae
- Not recovered
- Death
- Unknown.

Instructions for reporting changes in an ongoing AE during a patient's participation in the study are provided in the instructions that accompany the AE case report forms.

9.3 Serious Adverse Event (SAE)

9.3.1 Definition of a SAE

Each AE is to be classified by the Investigator as Serious or Non-Serious. This classification of the gravity of the event determines the reporting procedures to be followed.

An AE that meets one or more of the following criteria/outcomes is classified as Serious:

- Requires inpatient hospitalization or prolongation of existing hospitalization
- Results in persistent or significant disability/incapacity
- Is a congenital anomaly/birth defect
- Medically important
- Results in death
- Is life-threatening.

The term "life-threatening" in the definition of "serious" refers to an event in which the patient was at risk of death at the time of the event; it does not refer to an event which hypothetically might have caused death if it were more severe.

Elective surgery or other scheduled hospitalization periods that were planned before the patient was included in this study are not to be considered serious. However, the event must be reported on the AE page in the CRF and commented upon.

Overnight stay at hospital due to prolonged infusion time will not be reported as an SAE

Medical and scientific judgment must be exercised in deciding whether an AE is believed to be “medically important”. Medical important events may not be immediately life-threatening or result in death or hospitalization but may jeopardize the patient or may require intervention to prevent one of the other outcomes listed in the definition above.

9.3.2 Serious Adverse Event Reporting

SAEs, pregnancies and liver function abnormalities meeting pre-defined criteria will be reported promptly to sponsor as described in the following table once the investigator determines that the event meets the protocol definition for that event.

Table 7: Timelines for reporting SAEs, pregnancies and liver function abnormalities

	**Initial Reports**		**Follow-up Information on a Previous Report**	
**Type of Event**	**Time Frame**	**Documents**	**Time Frame**	**Documents**
All SAEs	24 hours	“SAE” data collection tool	24 hours	Updated “SAE” data collection tool
Pregnancy	2 Weeks	Pregnancy Form	2 Weeks	Updated Pregnancy Form
Liver chemistry abnormalities:				
ALT≥3xULN **and** Bilirubin≥1.5xULN)	24 hours	Liver Chemistry Report Form	24 hours	Updated Liver Chemistry Report Form
ALT≥5xULN **or** ALT≥3xULN with hepatitis or rash **or** ≥4 weeks	24 hours	Liver Chemistry Report Form	24 hours	Updated Liver Chemistry Report Form
ALT≥3xULN **and** <5xULN **and** biliribin <1.5xULN	24 hours	Liver Chemistry Report Form	24 hours	Updated Liver Chemistry Report Form

The method of detecting, recording, evaluating and follow-up of AEs and SAEs plus procedures for completing and transmitting SAE reports to sponsor are provided in the GSK SAE completing instructions. Procedures for post-study AEs/SAEs are provided in the GSK SAE completing instructions.

In case of SAEs, pregnancies and liver function abnormalities the investigator must within 24 hours of awareness of the event:

- Notify the CRA
- Immediately forward a mail with the SAE report form attached to us.naps@gsk.com or fax the SAE report form to GSK Global Clinical Safety and Pharmacovigilance at Fax no. +1 919-483-5404.

The investigator should be aware of local reporting regulations to the IRB/IEC. GSK or a designee will either supply the investigator with the reports which should be passed on to the IRB/IEC or report directly to the IRB/IEC depending on local regulations.

Events reported on the SAE report form occurring during the patient's participation in the trial must also be recorded on the AE page in the CRF

Any suspected trial drug related SAE, occurring at any time after the patient has terminated trial participation, should be reported to GSK Global Clinical Safety and Pharmacovigilance (email: us.naps@gsk.com or Fax no. +1 919-483-5404).

9.3.3 Suspected Unexpected Serious Adverse Reactions (SUSARs)

The sponsor has a legal responsibility to notify, as appropriate and according to local regulations, both the local regulatory authority and other regulatory agencies about the safety of the product under clinical investigation. Prompt notification of SAEs by the Investigator to the sponsor is essential so that legal obligations and ethical responsibilities towards the safety of patients are met.

The sponsor will ensure that all relevant information about SUSARs is recorded and reported as soon as possible to the competent regulatory authorities and/or to the Ethics Committee according to the applicable local regulatory requirements.

9.4 Follow-Up on Adverse Events

All AEs should be followed until they are resolved or the patient's participation in the trial ends whichever comes first. Grade 3 AEs and AEs meeting one of the serious criteria, judged related to trial drug and still ongoing after ended trial participation should be followed on a regular basis, according to the investigator’s clinical judgment, until the event has been resolved or until the investigator assesses it as chronic or stable.

9.5 Safety Monitoring

9.5.1 Safety Surveillance set up

An internal Joint Safety Committee for the trial will be established. All safety data reported during the trial including serious adverse events, non-serious adverse events and laboratory data will be evaluated.

9.5.2 Liver Chemistry Abnormalities

Liver chemistry threshold stopping criteria have been designed to assure patient safety. The liver event case report forms should be completed if protocol-specified liver chemistry patient stopping criteria are met. The liver imaging and/or liver biopsy case report forms should be completed if these tests are performed.

When patients meet one or more of the following liver chemistry threshold criteria, ofatumumab must be permanently withdrawn, additional testing performed, and the patient monitored until liver chemistries resolve, stabilize, or return to baseline (Visit 2 $_A$) values. The patient must then proceed to the Follow-up Period:

- ALT ≥ 3xULN and bilirubin ≥1.5xULN (>35% direct).
- ALT ≥ 5xULN
- ALT ≥ 3xULN if associated with the appearance or worsening of hepatitis symptoms or rash.

Patients with ALT ≥3xULN **and** <5xULN **and** bilirubin <1.5xULN, who do not exhibit hepatitis symptoms or rash, can continue ofatumumab≥ and be monitored weekly for up to 4 weeks. At any point, if these patients meet the liver chemistry threshold stopping criteria (outlined above) or are unable to return for weekly liver chemistries, ofatumumab must be permanently withdrawn, additional testing performed, and the patient continue safety follow-up until liver chemistries resolve, stabilize, or return to baseline values (Visit 2 $_A$). The patient must not receive additional ofatumumab courses.

Patients with ALT ≥3xULN **and** bilirubin ≥1.5xULN (>35% direct bilirubin; bilirubin fractionation required) must be immediately and permanently withdrawn from ofatumumab. Every attempt must be made to have the patient return to clinic (within 24 hours) for repeat liver chemistries and additional testing, and monitored closely (with specialist or hepatology consultation recommended). This event must be reported to sponsor within 24 hours of learning of its occurrence. Patients must be monitored twice weekly until liver chemistries (ALT, AST, alkaline phosphatase, bilirubin) resolve, stabilize or return to within baseline (Visit 2 $_A$) values. Upon completion of the safety follow-up, the patient must then proceed to Follow-up Period.

Patients with ALT≥5xULN **or** ALT ≥ 3x ULN with hepatitis or rash or if increase persists >4 weeks must be immediately and permanently withdrawn from ofatumumab. Every attempt must be made to have the subject return to clinic within 24-72 hours for repeat liver chemistries and additional testing, and monitored weekly until liver chemistries (ALT, AST, alkaline phosphatase, bilirubin) resolve, stabilize, or return to within baseline values. This event must be reported to the Sponsor within 24 hours of learning of its occurrence.

If, after 4 weeks of monitoring, ALT <3xULN **and** bilirubin <1.5xULN, patients should be monitored twice monthly until liver chemistries normalize or return to within baseline values. Note that ofatumumab must be withdrawn if bilirubin >1.5xULN **or** there are signs/symptoms of

hepatitis or hypersensitivity or elevations in ALT ≥3xULN **and** <5xULN persist for more than 4 weeks.

In all the above situations, every attempt must be made to obtain the following:

- Viral hepatitis serology including:
  - Hepatitis A IgM antibody;
  - Hepatitis B surface antigen and Hepatitis B Core Antibody (IgM);
  - Hepatitis C RNA;
  - Cytomegalovirus IgM antibody;
  - Epstein-Barr viral capsid antigen IgM antibody (or if unavailable, obtain heterophile antibody or monospot testing);
  - Hepatitis E IgM antibody (if patient resides outside the USA or Canada, or has traveled outside USA or Canada in past 3 months).
- Creatinine Phosphokinase (CPK) and lactate dehydrogenase (LDH)
- Fractionate bilirubin, if bilirubin > 1.5xULN.
- Record the use of concomitant medication, paracetamol (acetaminophen), herbal remedies, other over the counter medications, putative hepatotoxins, or alcohol on the concomitant medication report form.

The following are required for patients with ALT >3xULN and bilirubin >1.5xULN but are optional for other abnormal liver chemistries:

- Anti-nuclear antibody, anti-smooth muscle antibody, and Type 1 anti-liver kidney microsomal antibodies.
- Liver imaging (ultrasound, magnetic resonance, or computerized tomography (CT)) to evaluate liver disease.

9.5.3 Infections and malignancies

As part of the ongoing program to evaluate the benefit/risk of ofatumumab, this trial will include enhanced safety monitoring during the trial with regard to serious infections, malignancy, and deaths.

Cases of the opportunistic viral infection progressive multifocal leukoencephalopathy (PML) have been reported in patients with hematologic malignancies systemic lupus erythematosus and rheumatoid arthritis treated with another anti-CD20 antibody, rituximab. Additionally, cases of PML have occurred in patients who have not received rituximab. Most reports have been in patients with a compromised immune system, either due to medical conditions (lymphoma or blood cancers, HIV infection and congenital immunodeficiency syndromes and systemic lupus erythematosus) or

medical treatments (cancer chemotherapy and immunosuppressive medications in organ transplant recipients)*. In the Hx-CD20-406 trial one case of PML was observed in a CLL patient who had received ofatumumab (1x300mg & 10x2000mg) previously treated with fludarabine and alemtuzumab with low T-lymphocyte CD4 counts.

In order to accommodate potential developments of PML, neurological examinations:

Neurological Symptoms Questions

		YES	NO
1.	Does the subject report any new weakness?	☐	☐
2.	Does the subject report any new difficulty with coordination or walking?		
3.	Does the subject report any new signs of confusion, impaired memory or attention?		
4.	Does the subject appear apathetic compared to previous contacts?		
5.	Does the subject report any new visual disturbances?		
6.	Has the subject had any new trouble speaking, either slurring speech, difficulty getting out words, difficulty understanding words, or difficulty comprehending spoken language.		
7.	Does the subject have any other new neurological symptoms, including but not limited to:<br>New onset seizure<br>New sensory loss<br>New emotional liability		

If any of the above are answered "Yes" at any visit, the investigator will contact the Sponsor medical officer or designee and the patient will be referred to a neurologist and assessments of plasma/white cell JCV PCR will be performed throughout the duration of the trial. Once identified, signs and symptoms consistent with a diagnosis of PML will be reported promptly to sponsor. Neurological symptoms should not trigger an SAE unless the sign/ symptoms is consistent with an SAE. Signs and symptoms of PML include visual disturbances, ocular movements, ataxia, and changes in mental status such as disorientation or confusion. These symptoms are not an exhaustive list and the investigator should exercise judgment in deciding to report signs and symptoms to sponsor promptly.

If a patient develops neurological signs or symptoms consistent with PML, study drug is to be discontinued and the patient referred to a neurologist for evaluation. At a minimum, blood JCV PCR and/or brain MRI will be performed and if either is positive perform Cerebrospinal Fluid (CSF) JCV PCR. If blood JCV PCR and brain MRI are negative, the investigator will contact sponsor for appropriate action to be taken with study drug. All such patients will be followed until resolution. Any patient with a diagnosis of PML will be withdrawn from ofatumumab.

* Ref FDA Alert 12/2006

The investigator will do the following when reporting a serious infection, malignancy, death, or sign/symptom consistent with PML.

- Promptly report the event, as with any other SAE, as per Section of this protocol.
- Provide key source documentation for sponsor to assist with the safety evaluation process.

Examples of key source documents include but are not limited to: hospitalization records, discharge summaries, laboratory evaluations, biopsy results, culture/sensitivity results, death certificates, and autopsy reports.

If the patient has not otherwise been withdrawn from the study, then the investigator should contact sponsor to discuss the appropriate course of action regarding study continuation.

9.5.4 Critical Adverse Event

A Critical Adverse Event (CAE) is defined as follows:

- Occurrence of a treatment related Adverse Event (AE) graded by the investigator as grade 3 at the day of infusion and preventing the infusion to be resumed.
- Second occurrence of a treatment related bronchospasm graded by the investigator as grade 3 during one infusion.
- If the severity of an AE becomes grade 3 for the third time during one infusion.
- All infections reported as serious
- The occurrence of treatment related neurological events consistent with PML.
- Any malignancy.

Treatment related is defined as an event where either the investigator or the sponsor judges the AE to be related to trial drug.

9.5.5 Stopping Rules

If the number of patients with a Critical Adverse Event (CAE) reaches the limits specified in Table 8, further patient enrollment will be paused pending advice from the Joint Safety Committee (see Section 18.2). In Table 9, N is the number of patients enrolled and NCAE is the number of patients with a CAE. The error rate states the probability of pausing with an acceptable risk of a CAE of 5% during the study. The detection rate states the probability of pausing with an unacceptable risk of 10%.

Table 8 Limits of CAE with error and detection rates

N_{CAE}	N	Probability of pausing with acceptable CAE risk of 5% (Error rate)	Probability of pausing with unacceptable CAE risk of 10% (Detection rate)
6	50	3.8%	38%
10	100	5.3%	60%
17	200	6.6%	83%

For example, if 6 patients or more among the first 50 enrolled experience a CAE during the study, patient enrollment should be paused. Enrollment can only be continued following the decision from the Joint Safety Committee. In this manner, patients are divided into enrollment groups as indicated in Table 9, and NCAE's are the lower limit of discontinuation for the first group, the first and second combined, the three first groups combined, and so forth.

The Joint Safety Committee must evaluate the nature and the character of the CAE, the degree to which the adverse events are to be expected and the importance with regard to patient risk. The Joint Safety Committee shall carefully evaluate any CAE and recommend whether changes to dosing should be performed or other modifications to the planned conduct of the study. Thus it is possible to have reached the per protocol pre-defined maximum number of CAEs without having to stop the trial in case the Joint Safety Committee judges that the events do not constitute a safety signal that warrants modification to the planned conduct of the study.

10 Statistics

This section presents the principal features of the statistical analysis of this trial. Further details will be given in a separate Reporting and Analysis plan (RAP), which will be finalized before breaking the analysis population classification.

The analyses and presentations will be performed for the full analysis population unless specified otherwise.

The significance level is set to 5%. All confidence intervals will be two-sided.

All summary statistics of continuous variables will include: n, mean, median, standard deviation, minimum and maximum. All summaries presenting frequencies and incidences will include n, % and N, where N is the total number of patients with recorded values.

All data listings will include all treated patients.

In the event the sponsor decides to suspend or discontinue the intravenous route of administration development program for RA and this trial is terminated early, the primary analysis would not be performed because time to treatment withdrawal would not be a meaningful endpoint. In addition, only reduced analyses of secondary endpoints would be performed, but safety data would be reported as planned. Further details will be included in the RAP as appropriate.

10.1 Analysis Populations

The full analysis population comprises all patients who have been exposed to study drug irrespective of their compliance to the planned course of treatment. This is the primary analysis population and will be used for evaluation of all endpoints.

For the efficacy analyses, the per protocol (PP) analysis population includes patients that have not deviated from the protocol in such a manner that the assessment of efficacy endpoints may be biased. A patient may be excluded from the PP population due to:

- Use of disallowed concomitant medication
- Major violation of inclusion and/or exclusion criteria
- Any other major protocol violation which may affect the assessment of efficacy endpoints.

10.2 Statistical Analysis of Primary Endpoint

The primary objective is to evaluate the long-term effectiveness of repeated courses of ofatumumab in RA patients who previously received ofatumumab or placebo. The primary endpoint is time to treatment withdrawal, defined as the time from first infusion of ofatumumab until date of treatment

withdrawal. This is the most direct measure of the clinical effectiveness of the treatment; As long as the patient continues to respond to the treatment and is not experiencing any unacceptable side effects, the treatment is continued. The criteria for treatment withdrawal are given in Section 5.3.1.

Time to treatment withdrawal will be analyzed using the Kaplan-Meier method, estimating the proportion of patients who have not had treatment withdrawn over time. All premature discontinuations will count as treatment withdrawals. Patients not adhering to Section 7.4.2 with respect to disallowed concomitant medication will be considered as not responding to the treatment and the start date of the disallowed medication will be considered as a withdrawal date.

Supporting analyses will be considered in which discontinuations for reasons clearly unrelated to treatment are censored at the time of discontinuation.

10.3 Statistical Analysis of Secondary Endpoints

10.3.1 DAS28

Disease activity score (DAS) will be calculated by the site staff based on the following parameters:

Tender joint count (TJC), swollen joint count (SJC), Erythrocyte Sedimentation Rate (ESR) in mm/hour and patient global assessment (PGA) in mm using the formula:

$$\text{DAS28} = 0.555\sqrt{\text{TJC28}} + 0.284\sqrt{\text{SJC28}} + 0.7\ln\text{ESR} + 0.0142\times\text{PGA}$$

The joint counts will be based on 28 joints for DAS28.

The minimum DAS28 score over week 16-24 in the first and last treatment cycle as well as the change from baseline to the minimum will be summarized by descriptive statistics.

For withdrawals the last available DAS score will be carried forward (LOCF).

A sensitivity analysis will be conducted using the maximum DAS28 score over week 16-24 in the last available treatment cycle.

Individual profiles of DAS28 score over time will be presented with indications of re-treatment time points.

10.3.2 EULAR Response

The EULAR response is based on the DAS score which is a clinical index of RA disease activity that combines information from swollen joints, tender joints, the acute phase response and general health (see Section 9.3.3). The DAS-based European League Against Rheumatism (EULAR) response criteria were developed to measure individual response in clinical trials. The EULAR response criteria classify individual patients as non-, moderate, or good responders, dependent on the extent of change and the level of disease activity reached.

The EULAR response criteria are based on the DAS28 score as follows:

Table 9: EULAR Response Conversion Table

DAS28	Improvement in DAS28 from baseline		
	>1.2	>0.6 and ≤1.2	≤0.6
≤3.2	Good	Moderate	None
> 3.2 and ≤ 5.1	Moderate	Moderate	None
>5.1	Moderate	None	None

EULAR response at week 16, 20 or 24 in the first and last treatment cycle will be summarized by descriptive statistics. A patient will be considered a responder if good or moderate EULAR response is obtained at either week 16, 20 or 24.

Patients that withdraw or who receives prohibited therapy (according to Section 7.4.2) should be considered as non-responders in line with the ACR20/50/70 endpoints.

Estimates of the proportions with good or moderate EULAR response will be provided with 95% confidence intervals.

10.3.3 Time to first re-treatment

Time to first re-treatment, defined as the time from first infusion of ofatumumab until date of Infusion 1 of the first re-treatment course, will be analyzed using the Kaplan-Meier method. The median time to first re-treatment will be estimated with 95% confidence interval

Further statistical modeling of all the re-treatments will be attempted, characterizing the variation within and between patients in re-treatment intervals.

10.3.4 ACR20, AC50 and ACR70

The ACR score is based on improvement from baseline in tender (TJC) and swollen joint counts (SJC). A patient has achieved ACR20, ACR50 or ACR70 if the patient experiences ≥20%, ≥50% or ≥70% improvement from baseline respectively in:

- Tender Joint Count (TJC) and Swollen Joint Count (SJC)

and improvement from baseline in 3 out of 5 of the following assessment;

- patient pain assessment on a 10 cm VAS scale
- patient global assessment on a 10 cm VAS scale
- physician global assessment on a 10 cm VAS scale
- patient self-assessed disability (HAQ)
- C-reactive protein (CRP in mg/L)

Estimates and corresponding 95% CI will be calculated for ACR20, ACR50 and ACR70.

Patients that withdraw or who require rescue therapy (according to Section 7.4.2) will be considered as non-responders.

Missing CRP values used in the derivation of ACR20 will be replaced by a measure of erythrocyte sedimentation rate (ESR). For any other missing individual components or in case both the CRP and ESR measurements are missing, the last non-missing observation prior to week 16 will be carried forward (LOCF) for each component.

Patients who receive an intra-articular injection of corticosteroid in a single joint, for the following 12 weeks after the time of injection the joint will count as tender and swollen in the TJC, SJC, DAS28, EULAR response and ACR responses.

ACR response at week 16, 20 or 24 in the first and last treatment cycle will be summarized. A patient will be considered a responder if an ACR response is obtained at either week 16, 20 or 24. Withdrawals are counted as non-responders.

10.3.5 ACRn and ACR components

ACRn is the largest integer n, for which a patient meets the ACR criteria requiring an improvement of n%. Maximum ACRn over week 16-24 in the first and last treatment cycle will be summarized by descriptive statistics. For withdrawals the last available observation will be carried forward and used in the analysis.

A sensitivity analysis using the minimum ACRn over week 16-24 in the last available treatment cycle will be conducted

The individual components of the ACR will be presented using descriptive statistics.

10.3.6 Host Immune Response

Change in HAHA titer from baseline to end of trial will be listed and tabulated.

10.3.7 Clinical Safety Data

Abnormal findings in physical examination, body weight, ECG measurements and vital signs will be listed.

10.3.8 Laboratory Safety Data

Summary statistics for the laboratory measurements at each visit and also the change from baseline will be tabulated. Additionally the number and percent of patients with values of clinical concern will be summarized by treatment group. Clinical concern criteria will be specified in the analysis plan.

10.4 Patient reported outcomes

10.4.1 HAQ

HAQ disability scores and pain scores will be analyzed as the DAS scores.

10.5 Biomarkers, immune status, and whole blood transcriptional profiles

Biomarkers, immune status, and whole blood transcriptional profiles will be summarized by descriptive statistics.

10.6 Statistical Analysis of Secondary Endpoints (Pharmacokinetics)

10.6.1 Pharmacokinetic Data

Individual curves of serum concentration of ofatumumab will be presented for all patients including all available data. Further non-compartmental or compartmental PK modelling may be done. The analysis will be based on the full analysis population.

Further exploratory analyses will be performed where PK data is related to ACR20 values in order to investigate a possible PD/PK relationship.

10.7 Handling of Missing Data or Outliers

Unless otherwise mentioned no imputation of missing data will be done.

10.8 Subgroups and Center Effects

The primary endpoint and the secondary efficacy endpoints will also be presented by rheumatoid factor seropositivity and by country.

As explorative analyses, summary statistics will be made on number of treatment courses and average time to re-treatment.

10.9 Interim Analyses

No interim analyses are planned, however a cut of the database may be conducted for the purposes of the submission.

10.10 Determination of Sample Size

As this is an extension trial to Hx-CD20-403 all eligible patients from the Hx-CD20-403 trial will be included.

11 Ethics

11.1 Independent Ethics Committee (IEC) or Institutional Review Board (IRB)

The protocol, any amendments, the consent form, and the patient information must be approved by the health authorities according to local regulations and by appropriately constituted IECs or IRBs, before study initiation.

11.2 Patient Information and Informed Consent

The principal Investigator or his/her designee must obtain the written Informed Consent from each patient before any study related procedures are performed. Each patient must receive full patient information before giving consent. The patient information must contain full and adequate verbal and written information regarding the objective and procedures of the study and the possible risks involved.

Before signing the Informed Consent the patient must be given sufficient time to consider their possible participation. Further each patient must be informed about their right to withdraw from the study at any time.

Each patient must sign the Informed Consent form; the patient receives a copy of the signed form and the original is retained in the Investigator Site File. The Informed Consent forms must be signed and dated both by the patient and by the investigator providing the information to the patient.

11.3 Ethical and Risk/Benefit Considerations

The following ethical considerations have been considered:

Is this trial necessary?

Rheumatoid arthritis (RA) is a systemic inflammatory disease which affects 0.8-1.0% of all populations. For unknown reasons the immune system attacks the synovium (tissue lining the joint capsule), causing local inflammation. This inflammatory response results in the destruction of ligaments, cartilage and bone within the joint but also tendons and muscles that support the joint are affected. Unless the inflammatory processes are halted or controlled, the disease leads to substantial disability.

Current treatment approaches include non-steroidal anti-inflammatory drugs (NSAID) for the relief of inflammation and pain, and disease-modifying antirheumatic drugs (DMARDs) for slowing the progression of the disease. When systemic RA treatment has failed, intra-articular injections of corticosteroids can temporarily ease the symptoms of RA. Advances in treatment include the introduction of biologic DMARDs, offering a more effective and targeted therapy. None of the above mentioned treatments are curative and they are also associated with toxicity.

The overall objective in the treatment of RA is to maintain the quality of life of the patient by means of pain relief, reduction of inflammation and prevention of joint destruction and -deformities.

Therefore there is still an unmet need for new effective long-term treatments of active chronic RA.

What is the risk/benefit for the participating patients?

A phase I/II trial showed that ofatumumab has an acceptable safety profile and is well-tolerated. Most side effects occurred on the days of infusion and comprised fatigue, rigors, pyrexia, dyspnea, pharyngeal pain, rash, pruritus, urticaria, headache, flushing, hypotension and increased sweating. To prevent or relieve possible side effects the patient will receive pre-medication.

Ofatumumab has a theoretical advantage compared to other biologic DMARDs in being an antibody of fully human origin and therefore may have a lower potential to cause allergic reactions compared to antibodies of animal origin.

It is anticipated that the number of B-cells will deplete after treatment with ofatumumab. The number of B-cells will be monitored during and after the trial until the level of B-cells has normalized.

A phase I/II trial indicates clinical efficacy of ofatumumab in RA patients which the patients participating in this trial will benefit from. All patients will receive ofatumumab in this trial.

In relation to the assessment of the disease, a chest X-ray will be taken at the first visit. The dose of radiation from a chest X-ray is very small (0.25mRad). Knowing that all people receive approximately 100 mRad (400 times that of a chest x-ray) yearly from cosmic rays and trace radioactive minerals in surroundings, the increased risk of cancer by one chest X-ray is considered neglectable.

12 Compliance with Good Clinical Practice

This Trial Protocol is designed to comply with the Guideline produced by the International Conference on Harmonization (ICH) on the topic Good Clinical Practice (GCP) and published by the European Agency for the Evaluation of Medicinal Products as "Note for Guidance on Good Clinical Practice" (CPMP/ICH/135/95) (Approval 17 July 1996) as well as other relevant guidelines issued by ICH, primarily the efficacy guidelines.

12.1 Monitoring

Monitoring visits to the trial site will be made periodically during the trial, to ensure that:

- Data are authentic, accurate, and complete.
- Safety and rights of subjects are being protected.
- Study is conducted in accordance with the currently approved protocol and any other study agreements, GCP, and all applicable regulatory requirements.

Source documents will be reviewed for verification of agreement with data on Case Report Forms..

To ensure compliance with GCP and all applicable regulatory requirements, GSK may also conduct a quality assurance audit of the site records, and the regulatory agencies may conduct a regulatory inspection at any time during or after completion of the study. In the event of an audit or inspection, the investigator (and institution) must agree to grant the auditor(s) and inspector(s) direct access to all relevant documents and to allocate their time and the time of their staff to discuss any findings/relevant issues

It is important that the Investigator and their relevant personnel area available during the monitoring visits and possible audits and that sufficient time are devoted to the process.

12.2 Source Data Verification

Source Documents is original documents, data and records (e.g. hospital records, clinical and office charts, laboratory notes, memoranda, x-rays, subject files and, recorded data from automated instruments etc.). Source Data is considered all information in original records and certified copies of clinical findings, observations, or other activities in the study. Source Data are contained in Source Documents (original records or certified copies).

The location of source document will be registered on a form specifying where source data can be located e.g. medical record, CRF, lab reports etc.

The following items must be available for Source Data Verification (SDV) in source documents other than the CRF:

- Date of conducting Informed Consent
- Date of birth and sex

- Statement that the patient is participating in clinical trial GEN413 with the study drug ofatumumab
- Data for evaluation of eligibility criteria
- Relevant medical history and diagnosis
- Patient Identification Number
- Administration of trial drug
- All study visit dates
- Adverse Events or absence of Adverse Events
- Concomitant Medication including changes
- Date and reason for withdrawal from trial and / or trial treatment.

12.3 Study and Site Closure

Upon completion or termination of the study, the GSK monitor will conduct site closure activities with the investigator or site staff (as appropriate), in accordance with applicable regulations, GCP, and GSK Standard Operating Procedures.

GSK reserves the right to temporarily suspend or terminate the study at any time for reasons including (but not limited to) safety issues, ethical issues, or severe non-compliance. If GSK determines that such action is required, GSK will discuss the reasons for taking such action with the investigator or head of the medical institution (where applicable). When feasible, GSK will provide advance notice to the investigator or head of the medical institution of the impending action.

If a study is suspended or terminated for **safety reasons**, GSK will promptly inform all investigators, heads of the medical institutions (where applicable),and/or institutions conducting the study. GSK will also promptly inform the relevant regulatory authorities of the suspension/termination along with the reasons for such action. Where required by applicable regulations, the investigator or head of the medical institution must inform the IRB/IEC promptly and provide the reason(s) for the suspension/termination.

13 Data Handling and Record Keeping

13.1 Case Report Form (CRF)

The Sponsor or delegate will supply Case Report Forms (NCR paper) which should be completed for each randomized patient and signed by the Investigator. A separate CRF screening binder should be filled in for all screened patients. Eligible patients enrolled in the trial will have an additional CRF binder. For screening failures only the Screening Evaluation Form will be entered into the database.

The CRF books must be kept on file by the investigator and maintained in an up-to-date condition at all times. The investigator must sign and date all sections in the CRF books used and also any specific forms used, as indicated. Only medically qualified investigators can sign off data on clinical assessments/safety.

The original CRF for each patient will be checked against the patient's source documents at the study site by a CRA designated by the Sponsor. Instances of missing or unclear data will be discussed with the investigator for resolution. The original CRF will be taken by the CRA to the Sponsor or a designee and a copy will be left in the CRF.

Corrections of data should be made using one single line, leaving the corrected data clearly visible. The accurate data should be entered next to the inaccurate data. All changes should be initialed and dated. Correction fluids or erasers are not allowed.

Corrections to the patient data, after the original CRF pages have been retrieved by the Sponsor or a designee, will be made by the Sponsor or a designee issuing a Data Clarification Form (DCF) for each item. The DCF will be completed by investigator, the original forwarded to Sponsor or a designee and a copy to the site file/CRF at the involved site.

The completed original CRFs are the sole property of GSK and should not be made available in any form to third parties, except for a GSK representatives and representatives of appropriate Health/Regulatory Authorities, without written permission from GSK.

13.2 Archiving of Trial Documents at Site

The investigator at each investigational site must make arrangements to store the essential study documents (as defined in Essential Documents for the Conduct of a Clinical Trial (ICH E6, Guideline for Good Clinical Practice)) including the Investigator Site File, until GSK informs the investigator, in writing, that the documents are no longer to be retained. The minimum retention time will meet the strictest standard applicable to a particular site, as dictated by local laws/regulations, sponsor standard operating procedures, and/or institutional requirements.

In addition the investigator is responsible for archiving all relevant source documents so that the trial data can be compared against source data after completion of the study (e.g. in case of inspection from authorities).

The investigator is required to ensure the continued storage of the documents, even if the investigator, for example, leaves the clinic/practice or retires before the end of required storage period. The investigator must notify sponsor of any changes in the archival arrangements, including, but not limited to archival of records at an off-site facility or transfer of ownership of the records in the event that the investigator is no longer associated with the site.

GSK undertakes to store originally completed CRFs and separate copies of the above documents for the same period, except for source documents pertaining to the individual investigational site, which are kept by the investigator only.

Following closure of the study, the investigator or head of the medical institution (where applicable) must maintain all site study records (except for those required by local regulations to be maintained elsewhere) in a safe and secure location. The records must be easily accessible when needed (e.g. for sponsor audit or regulatory inspection) and must be available for review in conjunction with assessment of the facility, supporting systems, and relevant site staff.

Where permitted by local laws/regulations or institutional policy, some or all of the records may be maintained in a format other than hard copy (e.g. microfiche, scanned, electronic); however, caution must be exercised before such action is taken. The investigator must ensure that all reproductions are legible and are a true and accurate copy of the original. In addition, they must meet accessibility and retrieval standards, including regeneration of a hard copy, if required. The investigator must also ensure that an acceptable back-up of the reproductions exists and there is an acceptable quality control procedure in place for creating the reproductions.

14 Reporting and Communication of Results

14.1 Use of Information

All unpublished information relating to this trial and/or to the trial drug is considered confidential by the sponsor and shall remain the sole property of the sponsor (GSK).

The investigator must accept that GSK may use the information from this clinical trial in connection with the development of the product, and therefore, may disclose it as required to other investigators, to government licensing authorities, to regulatory agencies of other government, stock exchange market, and commercial partners.

14.2 Provision of Study Results to Investigators, Posting to the Clinical Trials Register and Publication

Where required by applicable regulatory requirements, an investigator signatory will be identified for the approval of the clinical study report. The investigator will be provided reasonable access to statistical tables, figures, and relevant reports and will have the opportunity to review the complete study results at a GSK site or other mutually-agreeable location.

GSK will also provide the investigator with the full summary of the study results. The investigator is encouraged to share the summary results with the study subjects, as appropriate.

GSK will provide the investigator with the randomization codes for their site only after completion of the full statistical analysis.

The results summary will be posted to the Clinical Study Register at the time of the first regulatory approval or within 12 months of any decision to terminate development. In addition, a manuscript will be submitted to a peer-reviewed journal for publication within 12 months of the first approval or within 12 months of any decision to terminate development. When manuscript publication in a peer-reviewed journal is not feasible, further study information will be posted to the GSK Clinical Study Register to supplement the results summary.

15 Insurance/Liability/Indemnity

The patients in the present study are covered by clinical trial insurance arranged by GSK, or by GSK itself in the event of study related injury or death, in accordance with applicable law and with the CPMP Note for Guidance on Good Clinical Practices (CPMP/ICH/135/95) of 17 July 1996.

16 Changes to the Final Protocol

Any variation in procedure from that specified in the Final Trial Protocol may lead to the results of the trial being questioned and in some cases rejected. Any proposed protocol change must therefore be discussed with and approved by GSK and submitted for the Ethics Committee and Health Authority approval or notification. Any protocol change should be documented in a Protocol Amendment.

17 Premature Termination of the Trial

If GSK International Coordinating Investigator, or the Joint Safety Committee discover conditions arising during the trial, which indicate that the clinical investigation should be halted, the trial can be temporarily suspended or terminated after appropriate consultation between GSK, the Joint Safety Committee, and International Coordinating Investigator. The Regulatory Authorities and Independent Ethics Committees/Institutional Review Board will be notified in writing. The reason will be stated.

Conditions that may warrant termination of the trial include, but are not limited to the following:

- The discovery of an unexpected and significant or unacceptable risk to the patients enrolled in the trial
- The discovery of lack of efficacy
- Failure of the Investigators to enter patients at an acceptable rate in the trial as a whole
- A decision on the part of GSK to suspend or discontinue development of the drug or formulation of drug in this indication.

In the event the trial is prematurely terminated, all subjects in the Treatment Period will enter the Follow-Up Period at the next scheduled study visit (see Section 6.2.3 for details on Follow–up activities)

17.1 Premature Termination of a Trial Site

Further GSK can decide to prematurely terminate single sites. Conditions that may warrant termination include, but are not limited to the following:

- Insufficient adherence to protocol requirements
- Failure to enter patients at an acceptable rate.

18 List of abbreviations and definitions of terms

18.1 List of abbreviations

ACR	American Collegue of Rheumatology
ADCC	Antibody Dependent Cell-mediated Cytotoxicity
ADR	Adverse Drug Reaction
AE	Adverse Event
ALP	Alkaline Phosphatase
ALT/ALAT	Alanine Amino Transferase
ALT/SGPT	Alanine Amino Transferase /Serum glutamate pyruvate Transaminase
ANCOVA	Analysis of Covariance
AST/SGOT	Aspartate Aminotransferase/Glutamate-oxaloacetate Transaminase
AUC	Area Under Curve
BLyS	B-Lymphocyte Stimulatory protein
BUN	Blood Urea Nitrogen
CAE	Critical Adverse Events
CCP	Cyclic Citrullinated Peptide
CDC	Complement Dependent Cytotoxicity
CHMP	Committee for Medicinal Products for Human Use
CLL	Chronic Lymphocytic Leukemia
CMH	Cochran Mantel Haenszel test
CPK	Creatinine Phosphokinase
CPMP	Committee for Propriety Medicinal Products
CRA	Clinical Research Associate
CRP	C - Reactive Protein
CT	Computerized Tomography
CTC	Common Terminology Criteria
CTCAE	Common Terminology Criteria Adverse Events
CTL4A-Ig	Cytotoxic T-lymphocyte-associated antigen 4
DAS28	Disease Activity Score (based on 28 joints)
DMARD	Disease-modifying antirheumatic drugs
DNA	DeoxyriboNucleic Acid

ECG	Electrocardiogram
eCRF	Electronic Case Report Form
EDTA	EthyleneDiamine Tetraacetic Acid
ESR	Erythrocyte Sedimentation Rate
EudraCT	European Clinical Trials Database
EULAR	European League Against Rheumatism
FACIT	Functional Assessment of Chronic Illness Therapy
FACS	Flow-activated Cell Sorter
FDA	Food and Drug Administration
FL	Follicular lymphoma
FU	Follow up
GCP	Good Clinical Practice
GGT	Gamma Glutaryl-Transferase
GSK	GlaxoSmithKline
HACA	Human Anti-Chimeric Antibodies
HAHA	Human Anti Human Antibodies
HAQ	Health Assessment Questionnaire
HB	Hepatitis B
HbsAg	Hepatitis B Surface Antigen
HCG	Human Chorionic Gonadotrophine
HCQ	Hydroxychloroquine
HIV	Human Immunodeficiency Virus
i.a.	Intra-articular
i.m.	Intra-muscular
i.v.	Intravenous
ICH	International Conference on Harmonization
IEC	Independent Ethics Committee
IgA	Immunoglobulin A
IgG	Immunoglobulin G
IgM	Immunoglobulin M
IL	Interleukin
IMP	Investigational Medicinal Product

IRB	Institutional Review Board
ITT	Intention To Treat
IVRS	Interactive Voice Response System
JCV	JC Virus
LDH	Lactate Dehydrogenase
LOCF	Last Observation Carried Forward
mAb	Monoclonal Antibody
MedDRA	Medical Dictionary for Regulatory Activities
mRNA	Messenger Ribonucleic Acid
MTD	Maximum Tolerated Dose
MTX	Methotrexate
NCI	National Cancer Institute
NSAID	Non-Steroidal Anti-Inflammatory Drugs
PCR	Polymerase Chain Reaction
PGA	Patient Global Assessment
PGx	Pharmacogenetics
PK	Pharmacokinetics
PML	Progressive Multifocal Leukoencephalopathy
PP	Per Protocol
PR	Partial Remission
QA	Quality Assurance
QC	Quality Control
RA	Rheumatoid Arthritis
RBC	Red blood cell
RF	Rheumatoid Factor
SAE	Serious Adverse Event
RAP	Reporting and Analysis Plan
SCID	Severe Combined Immunodeficiency
SDV	Source Data Verification
SF	Short form
SJC	Swollen Joint Count
SNP	Single Nucleotide Polymorphisms

SSZ	Sulphasalazine
SUSAR	Suspected Unexpected Serious Adverse Reaction
TGF-α	Transforming Growth Factor Alpha
TJC	Tender Joint Count
TNF-α	Tumor Necrosis Factor Alpha
ULN	Upper Limit of Normal
VAS	Visual analogue scale
WBC	White Blood Cell
WHO	World Health Organization

18.2 Definition of Terms

This was the patented trademark for this IMP. It has now been designated an INN, which is Ofatumumab.

International Coordinating Investigator

The International Coordinating Investigator is responsible for approval of the Clinical Trial Protocol and Report on behalf of all investigators.

National Coordinating Investigator

One National Coordinating Investigator may be appointed for each country. The National Coordinating Investigators will be responsible for national issues relating to the study.

Principal Investigators

The Principal Investigator at site is be responsible for all aspects of study conduct at his/her site. This includes ensuring that all personnel involved in the trial are fully informed of all relevant aspects of the trial, including detailed knowledge of and training in all procedures to be followed.

Sponsor

GSK is the sponsor for this trial.

Joint Safety Committee

A Joint Safety Committee for the trial will be established. The Joint Safety Committee will include representatives from Clinical Development, Statistics, Regulatory Affairs and Medical and Safety Departments at GSK.

19 References

(1) Scott DL, Symmons DP, Coulton BL, Popert AJ. Long-term outcome of treating rheumatoid arthritis: results after 20 years. Lancet 1987; 1(8542):1108-1111.

(2) van der Heijde DM. Joint erosions and patients with early rheumatoid arthritis. Br J Rheumatol 1995; 34 Suppl 2:74-78.

(3) Lard LR, Visser H, Speyer I, vander Horst-Bruinsma IE, Zwinderman AH, Breedveld FC et al. Early versus delayed treatment in patients with recent-onset rheumatoid arthritis: comparison of two cohorts who received different treatment strategies. Am J Med 2001; 111(6):446-451.

(4) McInnes IB. Rheumatoid arthritis. From bench to bedside. Rheum Dis Clin North Am 2001; 27(2):373-387.

(5) Petersen J, Ingemann-Hansen T, Halkjaer-Kristensen JS. Spontaneous and induced immunoglobulin secretion by synovial fluid B lymphocytes in rheumatoid arthritis. Ann Rheum Dis 1984; 43(2):140-145.

(6) Wang H, Marsters SA, Baker T, Chan B, Lee WP, Fu L et al. TACI-ligand interactions are required for T cell activation and collagen-induced arthritis in mice. Nat Immunol 2001; 2(7):632-637.

(7) Tan SM, Xu D, Roschke V, Perry JW, Arkfeld DG, Ehresmann GR et al. Local production of B lymphocyte stimulator protein and APRIL in arthritic joints of patients with inflammatory arthritis. Arthritis Rheum 2003; 48(4):982-992.

(8) Youinou P. B cell conducts the lymphocyte orchestra. J Autoimmun 2007; 28(2-3):143-151.

(9) Toubi E, Kessel A, Slobodin G, Boulman N, Pavlotzky E, Zisman D et al. Changes in macrophage function after rituximab treatment in patients with rheumatoid arthritis. Ann Rheum Dis 2007; 66(6):818-820.

(10) Edwards JC, Cambridge G, Abrahams VM. Do self-perpetuating B lymphocytes drive human autoimmune disease? Immunology 1999; 97(2):188-196.

(11) Edwards JC, Szczepanski L, Szechinski J, Filipowicz-Sosnowska A, Emery P, Close DR et al. Efficacy of B-cell-targeted therapy with rituximab in patients with rheumatoid arthritis. N Engl J Med 2004; 350(25):2572-2581.

(12) Kremer JM, Genant HK, Moreland LW, Russell AS, Emery P, Abud-Mendoza C et al. Effects of abatacept in patients with methotrexate-resistant active rheumatoid arthritis: a randomized trial. Ann Intern Med 2006; 144(12):865-876.

(13) Finckh A, Liang MH, van Herckenrode CM, de Pablo P. Long-term impact of early treatment on radiographic progression in rheumatoid arthritis: A meta-analysis. Arthritis Rheum 2006; 55(6):864-872.

(14) Felson DT, Anderson JJ, Meenan RF. The comparative efficacy and toxicity of second-line drugs in rheumatoid arthritis. Results of two metaanalyses. Arthritis Rheum 1990; 33(10):1449-1461.

(15) Morgan SL, Baggott JE, Vaughn WH, Austin JS, Veitch TA, Lee JY et al. Supplementation with folic acid during methotrexate therapy for rheumatoid arthritis. A double-blind, placebo-controlled trial. Ann Intern Med 1994; 121(11):833-841.

(16) Ortendahl M, Schettler JD, Fries JF. Factors influencing length of time taking methotrexate in rheumatoid arthritis. J Rheumatol 2000; 27(5):1139-1147.

(17) Jobanputra P, Wilson J, Douglas K, Burls A. A survey of British rheumatologists' DMARD preferences for rheumatoid arthritis. Rheumatology (Oxford) 2004; 43(2):206-210.

(18) Emery P, Fleischmann R, Filipowicz-Sosnowska A, Schechtman J, Szczepanski L, Kavanaugh A et al. The efficacy and safety of rituximab in patients with active rheumatoid arthritis despite methotrexate treatment: results of a phase IIB randomized, double-blind, placebo-controlled, dose-ranging trial. Arthritis Rheum 2006; 54(5):1390-1400.

(19) Edwards JC, Szczepanski L, Szechinski J, Filipowicz-Sosnowska A, Emery P, Close DR et al. Efficacy of B-cell-targeted therapy with rituximab in patients with rheumatoid arthritis. N Engl J Med 2004; 350(25):2572-2581.

(20) Finckh A, Ciurea A, Brulhart L, Kyburz D, Moller B, Dehler S et al. B cell depletion may be more effective than switching to an alternative anti-tumor necrosis factor agent in rheumatoid arthritis patients with inadequate response to anti-tumor necrosis factor agents. Arthritis Rheum 2007; 56(5):1417-1423.

(21) Higashida J, Wun T, Schmidt S, Naguwa SM, Tuscano JM. Safety and efficacy of rituximab in patients with rheumatoid arthritis refractory to disease modifying antirheumatic drugs and anti-tumor necrosis factor-alpha treatment. J Rheumatol 2005; 32(11):2109-2115.

(22) Westhovens R, Cole JC, Li T, Martin M, Maclean R, Lin P et al. Improved health-related quality of life for rheumatoid arthritis patients treated with abatacept who have inadequate response to anti-TNF therapy in a double-blind, placebo-controlled, multicentre randomized clinical trial. Rheumatology (Oxford) 2006; 45(10):1238-1246.

(23) Weinblatt M, Combe B, Covucci A, Aranda R, Becker JC, Keystone E. Safety of the selective costimulation modulator abatacept in rheumatoid arthritis patients receiving background biologic and nonbiologic disease-modifying antirheumatic drugs: A one-year randomized, placebo-controlled study. Arthritis Rheum 2006; 54(9):2807-2816.

(24) Leandro MJ, Edwards JC, Cambridge G. Clinical outcome in 22 patients with rheumatoid arthritis treated with B lymphocyte depletion. Ann Rheum Dis 2002; 61(10):883-888.

(25) Ng CM, Bruno R, Combs D, Davies B. Population pharmacokinetics of rituximab (anti-CD20 monoclonal antibody) in rheumatoid arthritis patients during a phase II clinical trial. J Clin Pharmacol 2005; 45(7):792-801.

(26) Cambridge G, Stohl W, Leandro MJ, Migone TS, Hilbert DM, Edwards JC. Circulating levels of B lymphocyte stimulator in patients with rheumatoid arthritis following rituximab treatment: relationships with B cell depletion, circulating antibodies, and clinical relapse. Arthritis Rheum 2006; 54(3):723-732.

(27) Leandro MJ, Cambridge G, Ehrenstein MR, Edwards JC. Reconstitution of peripheral blood B cells after depletion with rituximab in patients with rheumatoid arthritis. Arthritis Rheum 2006; 54(2):613-620.

(28) Brown AK, Quinn MA, Karim Z, Conaghan PG, Peterfy CG, Hensor E et al. Presence of significant synovitis in rheumatoid arthritis patients with disease-modifying antirheumatic drug-induced clinical remission: evidence from an imaging study may explain structural progression. Arthritis Rheum 2006; 54(12):3761-3773.

(29) Popa C, Leandro MJ, Cambridge G, Edwards JC. Repeated B lymphocyte depletion with rituximab in rheumatoid arthritis over 7 yrs. Rheumatology (Oxford) 2007; 46(4):626-630.

(30) Emery P, Furst DE, Ferraccioli G, Udell J, van Vollenhoven RF, Rowe K et al. Sustained efficacy of repeat treatment courses of rituximab in rheumatoid arthritis patients with an inadequate response to disease-modifying anti-rheumatic drugs. Ann Rheum Dis 66[Suppl II], 430. 2007.
Ref Type: Abstract

(31) van Vollenhoven R, Emery P, Bingham C, Keystone E, Greenwald M, Moreland LW et al. Long-term safety data from extended follow-up and repeat use of rituximab in rheumatoid arthritis. Ann Rheum Dis 66[Suppl II], 88. 2007.
Ref Type: Abstract

(32) Vos K, Thurlings RM, Wijbrandts CA, van Schaardenburg D, Gerlag DM, Tak PP. Early effects of rituximab on the synovial cell infiltrate in patients with rheumatoid arthritis. Arthritis Rheum 2007; 56(3):772-778.

(33) Emery P, Furst DE, Ferraccioli G, Udell J, van Vollenhoven RF, Rowe K et al. Long-term efficacy and safety of a repeat treatment course of rituximab in RA patients with an inadequate respons to disease-modifying anti-rheumatic drugs. Ann Rheum Dis 65[Suppl II], 58. 2006.
Ref Type: Abstract

(34) Uniform requirements for manuscripts submitted to biomedical journals. International Committee of Medical Journal Editors. N Engl J Med 1997; 336(4):309-315.

Appendices

Appendix 1: Rheumatoid arthritis functional class

I: Completely able to perform usual activities of daily living (self-care, vocational, and avocational)

II: Able to perform usual self-care and vocational activities, but limited in avocational activities

III: Able to perform usual self-care activities, but limited in vocational and avocational activities

IV: Limited in ability to perform usual self-care, vocational, and avocational activities

NB. If IV, do not include the patient in the study.

Usual self-care activities include dressing, feeding, bathing, grooming and toileting. Avocational (recreational and/or leisure) and vocational (work, school, homemaking) activities are patient-desired and age and sex specific.

Appendix 2: Pharmacogenetic assessment

Pharmacogenetic Research

Pharmacogenetics - Background

Pharmacogenetics (PGx) is the study of variability in drug response due to hereditary factors in different populations. There is increasing evidence that an individual's genetic composition (i.e., genotype) may impact the pharmacokinetics (absorption, distribution, metabolism, elimination), pharmacodynamics (relationship between concentrations and pharmacologic effects or the time course of pharmacologic effects) and/or clinical outcome (in terms of efficacy and/or safety and tolerability). Some reported examples of PGx analysis include:

Drug	Disease	Gene	Outcome
Abacavir	HIV [Hetherington, 2002; Mallal, 2002]	HLA (human leukocyte antigen)	Caucasian males with HLA B57 variant were at increased risk for experiencing hypersensitivity to abacavir
Tranilast	Restenosis prevention following coronary bypass [Roses, 2002]	UGT1A1	Drug induced hyperbilirubinemia explained by high proportion of affected patients having 7/7 TA repeat genotype, consistent with clinically benign Gilbert's Syndrome
ABT-761	Asthma [Drazen, 1999]	ALOX5	ALOX5 Sp1 promoter genotype (x,x) associated with reduced response to 5-lipoxygenase inhibitor ABT-761

A key component to successful PGx research is the collection of samples during the conduct of clinical studies.

Collection of whole blood samples, even when no a priory hypothesis has been identified, may enable PGx analysis to be conducted if at any time it appears that there is a potential unexpected or unexplained variation in handling or response to ofatumumab.

Pharmacogenetic Research Objectives

The objective of the PGx research (if there is a potential unexpected or unexplained variation) is to investigate a possible genetic relationship to handling or response to ofatumumab. If at any time it appears there is potential variability in response in this clinical study or in a series of clinical studies with ofatumumab that may be attributable to genetic variations of patients, the following objectives may be investigated:

- Relationship between genetic variants and the pharmacokinetics of investigational product.
- Relationship between genetic variants and safety and/or tolerability of investigational product.
- Relationship between genetic variants and efficacy of investigational product.

Study Population

Any patient who has given informed consent to participate in the clinical study, has met all the entry criteria for the clinical study, and receives investigational product may take part in the PGx research. Any patient who has received an allogeneic bone marrow transplant must be excluded from the PGx research.

Patient participation in the PGx research is voluntary and refusal to participate will not indicate withdrawal from the clinical study. Refusal to participate will involve no penalty or loss of benefits to which the patient would otherwise be entitled.

Study Assessments and Procedures

In addition to any blood samples take for the clinical study, a whole blood sample (~10ml) will be collected for the PGx research using a tube containing Ethylene Diamine Tetraacetic Acid (EDTA). The PGx sample is labeled (or coded) with a study specific number that can be traced or linked back to the patient by the investigator or site staff. Coded samples do not carry personal identifiers (such as name or social security number). The blood sample will be taken on a single occasion unless a duplicate sample is required due to inability to utilize the original sample. It is recommended that the blood sample be taken at the first opportunity after a patient has been randomized and provided informed consent for PGx research, but may be taken at any time while the patient is participating in the clinical study.

If deoxyribonucleic acid (DNA) is extracted from the blood sample, the DNA may be subjected to sample quality control analysis. This analysis will involve the genotyping of several genetic markers to confirm the integrity of individual samples. If inconsistencies are noted in the analysis, then those samples may be destroyed.

The need to conduct PGx analysis may be identified after a study (or set of studies) of ofatumumab has been completed and the study data reviewed.

In some cases, the samples may not be studied. e.g., no questions are raised about how people respond to Ofatumumab.

Samples will be stored securely and may be kept for up to 15 years after the last patient completes the study or the sponsor may destroy the samples sooner. The sponsor or those working with the sponsor (for example, other researchers) will use samples collected from the study for the purpose stated in this protocol and in the informed consent form.

Patients can request their sample to be destroyed at any time.

Patient Withdrawal from Study

If a patient who has consented to participate in PGx research and has a sample taken for PGx research withdraws from the clinical study for any reason other than lost to follow-up, the patient will be given the following options:

1. The sample is retained for PGx research
2. Any PGx sample is destroyed.

If a patient withdraws consent from the PGx research or requests sample destruction for any reason, the investigator must complete the appropriate documentation to request sample destruction within the timeframe specified by the sponsor and maintain the documentation in the site study records. In either case, the sponsor will only use study information collected/generated up to that point.

Screen Failures

If a blood sample for PGx research has been collected and it is determined that the patient does not meet the entry criteria for participation in the clinical study, then the investigator must complete the appropriate documentation to request sample destruction within the timeframe specified by the sponsor and maintain the documentation in the site study records.

Pharmacogenetics Analyses

Generally the sponsor will utilize two approaches to explore genetic variation in drug response.

1. Specific sections of DNA may be selected from areas of the genome (e.g., candidate genes) known to encode the drug target, drug metabolizing enzymes, areas associated with mechanisms underlying adverse events, and those linked to study disease and, thus, linked to drug response. The candidate genes that may be investigated in this study are the following: The CD20 molecule which represents the target of Ofatumumab on B-lymphocytes; and receptors and regulatory proteins that may have a role in the antibody-dependent cell-mediated cytotoxicity and complement-dependent cytotoxicity elicited by Ofatumumab including but not restricted to: 1) the V158F single nucleotide polymorphism in the FCGR3A gene which has been associated with increased efficacy to Rituximab [Cartron, 2003; Weng, 2003] and is a receptor on effector immune cells to which Ofatumumab binds; and 2) polymorphisms and/or copy number variation in complement subunits (e.g. C1Q, C1S, C2, C4, Factor H) and complement inhibitors (e.g. CD55, CD59). In addition, continuing research may identify other enzymes, transporters, proteins, or receptors that may be involved in response to Ofatumumab. The genes that may code for these proteins may also be studied.

2. By evaluating large numbers of polymorphic markers (e.g., single nucleotide polymorphisms (SNP)) throughout the genome, sets of markers may be identified that correspond to differential drug response.

Hardy-Weinberg Equilibrium testing

The genotypic frequencies of each polymorphism will be evaluated for conformity to those expected under normal conditions by employing Hardy-Weinberg Equilibrium testing.

Comparison of Demographic and Baseline Characteristics by Genotype

Differences in baseline clinical characteristics and potential contributing covariates may be summarized and compared among genotype (or haplotype) subgroups.

Evaluation of Genotypic Effects

Analyses may be carried out to evaluate the degree of association between patient genotype (or haplotype) and selected parameters (e.g., pharmacokinetics, efficacy and safety). Where such genotypic tests are inappropriate (for example, where the number of marker genotypes is too large and/or the frequency of individual genotypes too small), allelic tests may be conducted. Allelic tests evaluate whether the frequency of each marker allele is the same in responders and non-responders.

Evaluation of Treatment by Genotype and Gene-Gene Interaction

In addition to evaluating the main effects of the genotypes (haplotypes or alleles) on the selected parameters, the possibility of a treatment group by genotype (haplotype or allele) interaction will also be explored. If appropriate, the joint effects of multiple markers (gene-gene interactions) may also be evaluated.

Linkage Disequilibrium

For pairs of polymorphisms, the degree to which alleles from the two sites are correlated (linkage disequilibrium) may also be evaluated. If the genotypes at two polymorphic sites within a gene are shown to be statistically associated with a response to investigational product, the degree of linkage disequilibrium will aid interpretation in that it will indicate the extent to which the two sites are exerting independent effects.

Multiple Comparisons and Multiplicity

Adjustment to observed p-values may be made to limit erroneous conclusions due to multiple tests when multiple markers are evaluated (especially in the case of a genome scan for association).

Power and Sample Size Considerations

The ability to detect differential drug response among genotypes at a polymorphic site depends on the total number of patients genotyped and the frequency distribution of the different genotypes. Consequently, genotyping analyses are plausible for those polymorphic sites where the number of patients comprising the genotypic groups is sufficiently large; however, these frequencies will not be known until sufficient samples have been collected and genotyping is complete.

Estimates of sample sizes required to demonstrate genotype effects vary considerably, depending on the assumptions made about allele frequency, genetic effect size, and mechanism of inheritance [Cardon, 2000]. In the work by Palmer and Cookson [Palmer, 2001], which assumed a genotype relative risk of 1.5, it was estimated that more than 300 cases and 600 controls would be needed to conduct a genetic association analysis. In contrast, McCarthy and Hilfiker [McCarthy, 2000] showed that with a genotype relative risk of 2.16 and a relatively commonly occurring genotype, only 30 cases and 30 controls would be needed to demonstrate an association.

Published PGx examples include abacavir hypersensitivity reaction [Hetherington, 2002; Mallal, 2002] and tranilast induced hyperbilirubinemia [Roses, 2002] where genetic markers have been found to significantly associate with hypersensitivity reaction (abacavir) and hyperbilirubinemia (tranilast). These examples show that small sample sizes typically encountered in Phase I and Phase II studies may be sufficient to identify clinically relevant genetic associations.

Informed Consent

Patients who do not wish to participate in the PGx research may still participate in the clinical study. PGx informed consent must be obtained prior to any blood being taken for PGx research.

Provision of Study Results and Confidentiality of Patient's PGx Data

The sponsor may summarize the cumulative PGx research results in the clinical study report. In general, the sponsor does not inform the investigator, patient or anyone else (e.g., family members, study investigators, primary care physicians, insurers, or employers) of the PGx research results because the information generated from PGx studies is preliminary in nature, and the significance and scientific validity of the results are undetermined at such an early stage of research, under any circumstance unless required by law.

References

Cardon LR, Idury RM, Harris TJR, Witte JS, Elston RC. Testing drug response in the presence of genetic information: sampling issues for clinical trials. Pharmacogenetics. 2000; 10:503-10.

Cartron G, Dacheux L, Salles G, Solal-Celigny P, Bardos P, Colombat P, Watier H. Therapeutic activity of humanized anti-CD20 monocolonal antibody and polymorphism in IgG Fc receptor FcgRIIIa gene. Blood 2003; 99: 754-758.

Drazen JM, Yandava CN, Dube L, Szcerback N, Hippensteel R, Pillari A, Israel E, Schork N, Silverman ES, Katz DA, Drajesk J. Pharmacogenetic association between ALOX5 promoter genotype and the response to anti-asthma treatment. Nature Genet. 1999; 22:168-70.

Hetherington S, Hughes AR, Mosteller M, Shortino D, Baker KL, Spreen W, Lai E, Davies K, Handley A, Dow DJ, Fling ME, Stocum M, Bowman C, Thurmond LM, Roses AD. Genetic variations in HLA-B region and hypersensitivity reactions to abacavir. Lancet. 2002; 359:1121-2.

Mallal S, Nolan D, Witt C, Masel G, Martin AM, Moore C, Sayer D, Castley A, Mamotte C, Maxwell D, James I. Association between presence of HLA-B*5701, HLA-DR7, and HLA-DQ3 and hypersensitivity to HIV-1 reverse-transcriptase inhibitor abacavir. Lancet. 2002; 359:727-32.

McCarthy JJ, Hilfiker R. The use of single-nucleotide polymorphism maps in pharmacogenomics. Nat Biotechnol. 2000; 18:505-8.

Palmer LJ, Cookson WO. Using single nucleotide polymorphisms as a means to understanding the pathophysiology of asthma. Respir Res. 2001; 2:102-12.

Roses AD. Genome-based pharmacogenetics and the pharmaceutical industry. Nat Rev Drug Discov. 2002; 1:541-9.

Weng W-K and Levy R. Two immunoglobulin G fragment C receptor polymorphism independently predict response to rituximab in patients with follicular lymphoma. J. Clin. Oncol. 2003; 21: 3940-3947.

Attachment 2: NCI Common Terminology Criteria Adverse Events v. 3.0 (CTCAE) Published Date: August 9, 2006

Common Terminology Criteria for Adverse Events v3.0 (CTCAE)

Publish Date: August 9, 2006

Quick Reference

The NCI Common Terminology Criteria for Adverse Events v3.0 is a descriptive terminology which can be utilized for Adverse Event (AE) reporting. A grading (severity) scale is provided for each AE term.

Components and Organization

CATEGORY

A CATEGORY is a broad classification of AEs based on anatomy and/or pathophysiology. Within each CATEGORY, AEs are listed accompanied by their descriptions of severity (Grade).

Adverse Event Terms

An AE is any unfavorable and unintended sign (including an abnormal laboratory finding), symptom, or disease temporally associated with the use of a medical treatment or procedure that may or may *not* be considered related to the medical treatment or procedure. An AE is a term that is a unique representation of a specific event used for medical documentation and scientific analyses. Each AE term is mapped to a MedDRA term and code. AEs are listed alphabetically within CATEGORIES.

Short AE Name

The 'SHORT NAME' column is new and it is used to simplify documentation of AE names on Case Report Forms.

Supra-ordinate Terms

A supra-ordinate term is located within a CATEGORY and is a grouping term based on disease process, signs, symptoms, or diagnosis. A supra-ordinate term is followed by the word *'Select'* and is accompanied by specific AEs that are all related to the supra-ordinate term. Supra-ordinate terms provide clustering and consistent representation of Grade for related AEs. Supra-ordinate terms are not AEs, are not mapped to a MedDRA term and code, cannot be graded and cannot be used for reporting.

REMARK

A 'REMARK' is a clarification of an AE.

ALSO CONSIDER

An 'ALSO CONSIDER' indicates additional AEs that are to be graded if they are clinically significant.

NAVIGATION NOTE

A 'NAVIGATION NOTE' indicates the location of an AE term within the CTCAE document. It lists signs/symptoms alphabetically and the CTCAE term will appear in the same CATEGORY unless the 'NAVIGATION NOTE' states differently.

Grades

Grade refers to the severity of the AE. The CTCAE v3.0 displays Grades 1 through 5 with unique clinical descriptions of severity for each AE based on this general guideline:

Grade 1	Mild AE
Grade 2	Moderate AE
Grade 3	Severe AE
Grade 4	Life-threatening or disabling AE
Grade 5	Death related to AE

A Semi-colon indicates 'or' within the description of the grade.

An 'Em dash' (—) indicates a grade not available.

Not all Grades are appropriate for all AEs. Therefore, some AEs are listed with fewer than five options for Grade selection.

Grade 5

Grade 5 (Death) is not appropriate for some AEs and therefore is not an option.

The DEATH CATEGORY is new. Only one Supra-ordinate term is listed in this CATEGORY: 'Death not associated with CTCAE term – *Select*' with 4 AE options: Death NOS; Disease progression NOS; Multi-organ failure; Sudden death.

Important:

- Grade 5 is the only appropriate Grade
- This AE is to be used in the situation where a death
  1. cannot be reported using a CTCAE v3.0 term associated with Grade 5, or
  2. cannot be reported within a CTCAE CATEGORY as 'Other (Specify)'

Contents

ALLERGY/IMMUNOLOGY 1
AUDITORY/EAR 2
BLOOD/BONE MARROW 4
CARDIAC ARRHYTHMIA 5
CARDIAC GENERAL 7
COAGULATION 10
CONSTITUTIONAL SYMPTOMS 11
DEATH 13
DERMATOLOGY/SKIN 14
ENDOCRINE 17
GASTROINTESTINAL 19
GROWTH AND DEVELOPMENT 29
HEMORRHAGE/BLEEDING 30
HEPATOBILIARY/PANCREAS 34
INFECTION 35
LYMPHATICS 38
METABOLIC/LABORATORY 40
MUSCULOSKELETAL/SOFT TISSUE 43
NEUROLOGY 47
OCULAR/VISUAL 52
PAIN 55
PULMONARY/UPPER RESPIRATORY 56
RENAL/GENITOURINARY 60
SECONDARY MALIGNANCY 63
SEXUAL/REPRODUCTIVE FUNCTION 64
SURGERY/INTRA-OPERATIVE INJURY 66
SYNDROMES 68
VASCULAR 70

ALLERGY/IMMUNOLOGY

		Grade				
**Adverse Event**	**Short Name**	**1**	**2**	**3**	**4**	**5**
Allergic reaction/ hypersensitivity (including drug fever)	Allergic reaction	Transient flushing or rash; drug fever <38°C (<100.4°F)	Rash; flushing; urticaria; dyspnea; drug fever ≥38°C (≥100.4°F)	Symptomatic bronchospasm, with or without urticaria; parenteral medication(s) indicated; allergy-related edema/angioedema; hypotension	Anaphylaxis	Death
REMARK: Urticaria with manifestations of allergic or hypersensitivity reaction is graded as Allergic reaction/hypersensitivity (including drug fever).						
ALSO CONSIDER: Cytokine release syndrome/acute infusion reaction.						
Allergic rhinitis (including sneezing, nasal stuffiness, postnasal drip)	Rhinitis	Mild, intervention not indicated	Moderate, intervention indicated	—	—	—
REMARK: Rhinitis associated with obstruction or stenosis is graded as Obstruction/stenosis of airway – *Select* in the PULMONARY/UPPER RESPIRATORY CATEGORY.						
Autoimmune reaction	Autoimmune reaction	Asymptomatic and serologic or other evidence of autoimmune reaction, with normal organ function and intervention not indicated	Evidence of autoimmune reaction involving a non-essential organ or function (e.g., hypothyroidism)	Reversible autoimmune reaction involving function of a major organ or other adverse event (e.g., transient colitis or anemia)	Autoimmune reaction with life-threatening consequences	Death
ALSO CONSIDER: Colitis; Hemoglobin; Hemolysis (e.g., immune hemolytic anemia, drug-related hemolysis); Thyroid function, low (hypothyroidism).						
Serum sickness	Serum sickness	—	—	Present	—	Death
NAVIGATION NOTE: Splenic function is graded in the BLOOD/BONE MARROW CATEGORY.						
NAVIGATION NOTE: Urticaria as an isolated symptom is graded as Urticaria (hives, welts, wheals) in the DERMATOLOGY/SKIN CATEGORY.						
Vasculitis	Vasculitis	Mild, intervention not indicated	Symptomatic, non-steroidal medical intervention indicated	Steroids indicated	Ischemic changes; amputation indicated	Death
Allergy/Immunology – Other (Specify, __)	Allergy – Other (Specify)	Mild	Moderate	Severe	Life-threatening; disabling	Death

AUDITORY/EAR

		Grade				
Adverse Event	Short Name	1	2	3	4	5
NAVIGATION NOTE: Earache (otalgia) is graded as Pain – *Select* in the PAIN CATEGORY.						
Hearing: patients with/without baseline audiogram and enrolled in a monitoring program[1]	Hearing (monitoring program)	Threshold shift or loss of 15 – 25 dB relative to baseline, averaged at 2 or more contiguous test frequencies in at least one ear; or subjective change in the absence of a Grade 1 threshold shift	Threshold shift or loss of >25 – 90 dB, averaged at 2 contiguous test frequencies in at least one ear	Adult only: Threshold shift of >25 – 90 dB, averaged at 3 contiguous test frequencies in at least one ear<br>Pediatric: Hearing loss sufficient to indicate therapeutic intervention, including hearing aids (e.g., ≥20 dB bilateral HL in the speech frequencies; ≥30 dB unilateral HL; and requiring additional speech-language related services)	Adult only: Profound bilateral hearing loss (>90 dB)<br>Pediatric: Audiologic indication for cochlear implant and requiring additional speech-language related services	—
REMARK: Pediatric recommendations are identical to those for adults, unless specified. For children and adolescents (≤18 years of age) without a baseline test, pre-exposure/pre-treatment hearing should be considered to be <5 dB loss.						
Hearing: patients without baseline audiogram and not enrolled in a monitoring program[1]	Hearing (without monitoring program)	—	Hearing loss not requiring hearing aid or intervention (i.e., not interfering with ADL)	Hearing loss requiring hearing aid or intervention (i.e., interfering with ADL)	Profound bilateral hearing loss (>90 dB)	—
REMARK: Pediatric recommendations are identical to those for adults, unless specified. For children and adolescents (≤18 years of age) without a baseline test, pre-exposure/pre-treatment hearing should be considered to be <5 dB loss.						
Otitis, external ear (non-infectious)	Otitis, external	External otitis with erythema or dry desquamation	External otitis with moist desquamation, edema, enhanced cerumen or discharge; tympanic membrane perforation; tympanostomy	External otitis with mastoiditis; stenosis or osteomyelitis	Necrosis of soft tissue or bone	Death
ALSO CONSIDER: Hearing: patients with/without baseline audiogram and enrolled in a monitoring program[1]; Hearing: patients without baseline audiogram and not enrolled in a monitoring program[1].						
Otitis, middle ear (non-infectious)	Otitis, middle	Serous otitis	Serous otitis, medical intervention indicated	Otitis with discharge; mastoiditis	Necrosis of the canal soft tissue or bone	Death

AUDITORY/EAR

		Grade				
**Adverse Event**	**Short Name**	**1**	**2**	**3**	**4**	**5**
Tinnitus	Tinnitus	—	Tinnitus not interfering with ADL	Tinnitus interfering with ADL	Disabling	—
ALSO CONSIDER: Hearing: patients with/without baseline audiogram and enrolled in a monitoring program[1]; Hearing: patients without baseline audiogram and not enrolled in a monitoring program[1].						
Auditory/Ear – Other (Specify, __)	Auditory/Ear – Other (Specify)	Mild	Moderate	Severe	Life-threatening; disabling	Death

[1] Drug-induced ototoxicity should be distinguished from age-related threshold decrements or unrelated cochlear insult. When considering whether an adverse event has occurred, it is first necessary to classify the patient into one of two groups. (1) The patient is under standard treatment/enrolled in a clinical trial <2.5 years, and has a 15 dB or greater threshold shift averaged across two contiguous frequencies; or (2) The patient is under standard treatment/enrolled in a clinical trial >2.5 years, and the difference between the expected age-related and the observed threshold shifts is 15 dB or greater averaged across two contiguous frequencies. Consult standard references for appropriate age- and gender-specific hearing norms, e.g., Morrell, et al. Age- and gender-specific reference ranges for hearing level and longitudinal changes in hearing level. Journal of the Acoustical Society of America 100:1949-1967, 1996; or Shotland, et al. Recommendations for cancer prevention trials using potentially ototoxic test agents. Journal of Clinical Oncology 19:1658-1663, 2001.

In the absence of a baseline prior to initial treatment, subsequent audiograms should be referenced to an appropriate database of normals. ANSI. (1996)

American National Standard: Determination of occupational noise exposure and estimation of noise-induced hearing impairment, ANSI S 3.44-1996. (Standard S 3.44). New York: American National Standards Institute. The recommended ANSI S3.44 database is Annex B.

BLOOD/BONE MARROW

		Grade				
**Adverse Event**	**Short Name**	**1**	**2**	**3**	**4**	**5**
Bone marrow cellularity	Bone marrow cellularity	Mildly hypocellular or ≤25% reduction from normal cellularity for age	Moderately hypocellular or >25 – ≤50% reduction from normal cellularity for age	Severely hypocellular or >50 – ≤75% reduction cellularity from normal for age	—	Death
CD4 count	CD4 count	<LLN – 500/mm^3<br><LLN – 0.5 x 10^9 /L	<500 – 200/mm^3<br><0.5 – 0.2 x 10^9 /L	<200 – 50/mm^3<br><0.2 x 0.05 – 10^9 /L	<50/mm^3<br><0.05 x 10^9 /L	Death
Haptoglobin	Haptoglobin	<LLN	—	Absent	—	Death
Hemoglobin	Hemoglobin	<LLN – 10.0 g/dL<br><LLN – 6.2 mmol/L<br><LLN – 100 g/L	<10.0 – 8.0 g/dL<br><6.2 – 4.9 mmol/L<br><100 – 80g/L	<8.0 – 6.5 g/dL<br><4.9 – 4.0 mmol/L<br><80 – 65 g/L	<6.5 g/dL<br><4.0 mmol/L<br><65 g/L	Death
Hemolysis (e.g., immune hemolytic anemia, drug-related hemolysis)	Hemolysis	Laboratory evidence of hemolysis only (e.g., direct antiglobulin test [DAT, Coombs'] schistocytes)	Evidence of red cell destruction and ≥2 gm decrease in hemoglobin, no transfusion	Transfusion or medical intervention (e.g., steroids) indicated	Catastrophic consequences of hemolysis (e.g., renal failure, hypotension, bronchospasm, emergency splenectomy)	Death
ALSO CONSIDER: Haptoglobin; Hemoglobin.						
Iron overload	Iron overload	—	Asymptomatic iron overload, intervention not indicated	Iron overload, intervention indicated	Organ impairment (e.g., endocrinopathy, cardiopathy)	Death
Leukocytes (total WBC)	Leukocytes	<LLN – 3000/mm^3<br><LLN – 3.0 x 10^9 /L	<3000 – 2000/mm^3<br><3.0 – 2.0 x 10^9 /L	<2000 – 1000/mm^3<br><2.0 – 1.0 x 10^9 /L	<1000/mm^3<br><1.0 x 10^9 /L	Death
Lymphopenia	Lymphopenia	<LLN – 800/mm^3<br><LLN x 0.8 – 10^9 /L	<800 – 500/mm^3<br><0.8 – 0.5 x 10^9 /L	<500 – 200 mm^3<br><0.5 – 0.2 x 10^9 /L	<200/mm^3<br><0.2 x 10^9 /L	Death
Myelodysplasia	Myelodysplasia	—	—	Abnormal marrow cytogenetics (marrow blasts ≤5%)	RAEB or RAEB-T (marrow blasts >5%)	Death
Neutrophils/granulocytes (ANC/AGC)	Neutrophils	<LLN – 1500/mm^3<br><LLN – 1.5 x 10^9 /L	<1500 – 1000/mm^3<br><1.5 – 1.0 x 10^9 /L	<1000 – 500/mm^3<br><1.0 – 0.5 x 10^9 /L	<500/mm^3<br><0.5 x 10^9 /L	Death
Platelets	Platelets	<LLN – 75,000/mm^3<br><LLN – 75.0 x 10^9 /L	<75,000 – 50,000/mm^3<br><75.0 – 50.0 x 10^9 /L	<50,000 – 25,000/mm^3<br><50.0 – 25.0 x 10^9 /L	<25,000/mm^3<br><25.0 x 10^9 /L	Death
Splenic function	Splenic function	Incidental findings (e.g., Howell-Jolly bodies)	Prophylactic antibiotics indicated	—	Life-threatening consequences	Death
Blood/Bone Marrow – Other (Specify, __)	Blood – Other (Specify)	Mild	Moderate	Severe	Life-threatening; disabling	Death

CARDIAC ARRHYTHMIA						
		**Grade**				
**Adverse Event**	**Short Name**	**1**	**2**	**3**	**4**	**5**
Conduction abnormality/ atrioventricular heart block – *Select*:<br>– Asystole<br>– AV Block-First degree<br>– AV Block-Second degree Mobitz Type I (Wenckebach)<br>– AV Block-Second degree Mobitz Type II<br>– AV Block-Third degree (Complete AV block)<br>– Conduction abnormality NOS<br>– Sick Sinus Syndrome<br>– Stokes-Adams Syndrome<br>– Wolff-Parkinson-White Syndrome	Conduction abnormality – *Select*	Asymptomatic, intervention not indicated	Non-urgent medical intervention indicated	Incompletely controlled medically or controlled with device (e.g., pacemaker)	Life-threatening (e.g., arrhythmia associated with CHF, hypotension, syncope, shock)	Death
Palpitations	Palpitations	Present	Present with associated symptoms (e.g., lightheadedness, shortness of breath)	—	—	—
REMARK: Grade palpitations only in the absence of a documented arrhythmia.						
Prolonged QTc interval	Prolonged QTc	QTc >0.45 – 0.47 second	QTc >0.47 – 0.50 second; ≥0.06 second above baseline	QTc >0.50 second	QTc >0.50 second; life-threatening signs or symptoms (e.g., arrhythmia, CHF, hypotension, shock syncope); Torsade de pointes	Death
Supraventricular and nodal arrhythmia – *Select*:<br>– Atrial fibrillation<br>– Atrial flutter<br>– Atrial tachycardia/Paroxysmal Atrial Tachycardia<br>– Nodal/Junctional<br>– Sinus arrhythmia<br>– Sinus bradycardia<br>– Sinus tachycardia<br>– Supraventricular arrhythmia NOS<br>– Supraventricular extrasystoles (Premature Atrial Contractions; Premature Nodal/Junctional Contractions)<br>– Supraventricular tachycardia	Supraventricular arrhythmia – *Select*	Asymptomatic, intervention not indicated	Non-urgent medical intervention indicated	Symptomatic and incompletely controlled medically, or controlled with device (e.g., pacemaker)	Life-threatening (e.g., arrhythmia associated with CHF, hypotension, syncope, shock)	Death
NAVIGATION NOTE: Syncope is graded as Syncope (fainting) in the NEUROLOGY CATEGORY.						

CARDIAC ARRHYTHMIA						
		Grade				
**Adverse Event**	**Short Name**	**1**	**2**	**3**	**4**	**5**
Vasovagal episode	Vasovagal episode	—	Present without loss of consciousness	Present with loss of consciousness	Life-threatening consequences	Death
Ventricular arrhythmia – *Select:*<br>– Bigeminy<br>– Idioventricular rhythm<br>– PVCs<br>– Torsade de pointes<br>– Trigeminy<br>– Ventricular arrhythmia NOS<br>– Ventricular fibrillation<br>– Ventricular flutter<br>– Ventricular tachycardia	Ventricular arrhythmia – *Select*	Asymptomatic, no intervention indicated	Non-urgent medical intervention indicated	Symptomatic and incompletely controlled medically or controlled with device (e.g., defibrillator)	Life-threatening (e.g., arrhythmia associated with CHF, hypotension, syncope, shock)	Death
Cardiac Arrhythmia – Other (Specify, __)	Cardiac Arrhythmia – Other (Specify)	Mild	Moderate	Severe	Life-threatening; disabling	Death

CARDIAC GENERAL

		Grade				
**Adverse Event**	**Short Name**	**1**	**2**	**3**	**4**	**5**
NAVIGATION NOTE: Angina is graded as Cardiac ischemia/infarction in the CARDIAC GENERAL CATEGORY.						
Cardiac ischemia/infarction	Cardiac ischemia/infarction	Asymptomatic arterial narrowing without ischemia	Asymptomatic and testing suggesting ischemia; stable angina	Symptomatic and testing consistent with ischemia; unstable angina; intervention indicated	Acute myocardial infarction	Death
Cardiac troponin I (cTnI)	cTnI	—	—	Levels consistent with unstable angina as defined by the manufacturer	Levels consistent with myocardial infarction as defined by the manufacturer	Death
Cardiac troponin T (cTnT)	cTnT	0.03 – <0.05 ng/mL	0.05 – <0.1 ng/mL	0.1 – <0.2 ng/mL	0.2 ng/mL	Death
Cardiopulmonary arrest, cause unknown (non-fatal)	Cardiopulmonary arrest	—	—	—	Life-threatening	—
REMARK: Grade 4 (non-fatal) is the only appropriate grade. CTCAE provides three alternatives for reporting Death:<br>1. A CTCAE term associated with Grade 5.<br>2. A CTCAE 'Other (Specify, __)' within any CATEGORY.<br>3. Death not associated with CTCAE term – *Select* in the DEATH CATEGORY.						
NAVIGATION NOTE: Chest pain (non-cardiac and non-pleuritic) is graded as Pain – *Select* in the PAIN CATEGORY.						
NAVIGATION NOTE: CNS ischemia is graded as CNS cerebrovascular ischemia in the NEUROLOGY CATEGORY.						
Hypertension	Hypertension	Asymptomatic, transient (<24 hrs) increase by >20 mmHg (diastolic) or to >150/100 if previously WNL; intervention not indicated<br><br>Pediatric:<br>Asymptomatic, transient (<24 hrs) BP increase >ULN; intervention not indicated	Recurrent or persistent (≥24 hrs) or symptomatic increase by >20 mmHg (diastolic) or to >150/100 if previously WNL; monotherapy may be indicated<br><br>Pediatric:<br>Recurrent or persistent (≥24 hrs) BP >ULN; monotherapy may be indicated	Requiring more than one drug or more intensive therapy than previously<br><br>Pediatric:<br>Same as adult	Life-threatening consequences (e.g., hypertensive crisis)<br><br>Pediatric:<br>Same as adult	Death
REMARK: Use age and gender-appropriate normal values >95th percentile ULN for pediatric patients.						

		Grade				
**Adverse Event**	**Short Name**	**1**	**2**	**3**	**4**	**5**
Hypotension	Hypotension	Changes, intervention not indicated	Brief (<24 hrs) fluid replacement or other therapy; no physiologic consequences	Sustained (≥24 hrs) therapy, resolves without persisting physiologic consequences	Shock (e.g., acidemia; impairment of vital organ function)	Death
ALSO CONSIDER: Syncope (fainting).						
Left ventricular diastolic dysfunction	Left ventricular diastolic dysfunction	Asymptomatic diagnostic finding; intervention not indicated	Asymptomatic, intervention indicated	Symptomatic CHF responsive to intervention	Refractory CHF, poorly controlled; intervention such as ventricular assist device or heart transplant indicated	Death
Left ventricular systolic dysfunction	Left ventricular systolic dysfunction	Asymptomatic, resting ejection fraction (EF) <60 – 50%; shortening fraction (SF) <30 – 24%	Asymptomatic, resting EF <50 – 40%; SF <24 – 15%	Symptomatic CHF responsive to intervention; EF <40 – 20% SF <15%	Refractory CHF or poorly controlled; EF <20%; intervention such as ventricular assist device, ventricular reduction surgery, or heart transplant indicated	Death
NAVIGATION NOTE: Myocardial infarction is graded as Cardiac ischemia/infarction in the CARDIAC GENERAL CATEGORY.						
Myocarditis	Myocarditis	—	—	CHF responsive to intervention	Severe or refractory CHF	Death
Pericardial effusion (non-malignant)	Pericardial effusion	Asymptomatic effusion	—	Effusion with physiologic consequences	Life-threatening consequences (e.g., tamponade); emergency intervention indicated	Death
Pericarditis	Pericarditis	Asymptomatic, ECG or physical exam (rub) changes consistent with pericarditis	Symptomatic pericarditis (e.g., chest pain)	Pericarditis with physiologic consequences (e.g., pericardial constriction)	Life-threatening consequences; emergency intervention indicated	Death
NAVIGATION NOTE: Pleuritic pain is graded as Pain – *Select* in the PAIN CATEGORY.						
Pulmonary hypertension	Pulmonary hypertension	Asymptomatic without therapy	Asymptomatic, therapy indicated	Symptomatic hypertension, responsive to therapy	Symptomatic hypertension, poorly controlled	Death
Restrictive cardiomyopathy	Restrictive cardiomyopathy	Asymptomatic, therapy not indicated	Asymptomatic, therapy indicated	Symptomatic CHF responsive to intervention	Refractory CHF, poorly controlled; intervention such as ventricular assist device, or heart transplant indicated	Death

CARDIAC GENERAL

		Grade				
**Adverse Event**	**Short Name**	**1**	**2**	**3**	**4**	**5**
Right ventricular dysfunction (cor pulmonale)	Right ventricular dysfunction	Asymptomatic without therapy	Asymptomatic, therapy indicated	Symptomatic cor pulmonale, responsive to intervention	Symptomatic cor pulmonale poorly controlled; intervention such as ventricular assist device, or heart transplant indicated	Death
Valvular heart disease	Valvular heart disease	Asymptomatic valvular thickening with or without mild valvular regurgitation or stenosis; treatment other than endocarditis prophylaxis not indicated	Asymptomatic; moderate regurgitation or stenosis by imaging	Symptomatic; severe regurgitation or stenosis; symptoms controlled with medical therapy	Life-threatening; disabling; intervention (e.g., valve replacement, valvuloplasty) indicated	Death
Cardiac General – Other (Specify, __)	Cardiac General – Other (Specify)	Mild	Moderate	Severe	Life-threatening; disabling	Death

COAGULATION

		Grade				
**Adverse Event**	**Short Name**	**1**	**2**	**3**	**4**	**5**
DIC (disseminated intravascular coagulation)	DIC	—	Laboratory findings with no bleeding	Laboratory findings and bleeding	Laboratory findings, life-threatening or disabling consequences (e.g., CNS hemorrhage, organ damage, or hemodynamically significant blood loss)	Death
REMARK: DIC (disseminated intravascular coagulation) must have increased fibrin split products or D-dimer.						
ALSO CONSIDER: Platelets.						
Fibrinogen	Fibrinogen	<1.0 – 0.75 x LLN or <25% decrease from baseline	<0.75 – 0.5 x LLN or 25 – <50% decrease from baseline	<0.5 – 0.25 x LLN or 50 – <75% decrease from baseline	<0.25 x LLN or 75% decrease from baseline or absolute value <50 mg/dL	Death
REMARK: Use % decrease only when baseline is <LLN (local laboratory value).						
INR (International Normalized Ratio of prothrombin time)	INR	>1 – 1.5 x ULN	>1.5 – 2 x ULN	>2 x ULN	—	—
ALSO CONSIDER: Hemorrhage, CNS; Hemorrhage, GI – *Select*; Hemorrhage, GU – *Select*; Hemorrhage, pulmonary/upper respiratory – *Select*.						
PTT (Partial Thromboplastin Time)	PTT	>1 – 1.5 x ULN	>1.5 – 2 x ULN	>2 x ULN	—	—
ALSO CONSIDER: Hemorrhage, CNS; Hemorrhage, GI – *Select*; Hemorrhage, GU – *Select*; Hemorrhage, pulmonary/upper respiratory – *Select*.						
Thrombotic microangiopathy (e.g., thrombotic thrombocytopenic purpura [TTP] or hemolytic uremic syndrome [HUS])	Thrombotic microangiopathy	Evidence of RBC destruction (schistocytosis) without clinical consequences	—	Laboratory findings present with clinical consequences (e.g., renal insufficiency, petechiae)	Laboratory findings and life-threatening or disabling consequences, (e.g., CNS hemorrhage/ bleeding or thrombosis/ embolism or renal failure)	Death
REMARK: Must have microangiopathic changes on blood smear (e.g., schistocytes, helmet cells, red cell fragments).						
ALSO CONSIDER: Creatinine; Hemoglobin; Platelets.						
Coagulation – Other (Specify, __)	Coagulation – Other (Specify)	Mild	Moderate	Severe	Life-threatening; disabling	Death

		Grade				
**Adverse Event**	**Short Name**	**1**	**2**	**3**	**4**	**5**
Fatigue (asthenia, lethargy, malaise)	Fatigue	Mild fatigue over baseline	Moderate or causing difficulty performing some ADL	Severe fatigue interfering with ADL	Disabling	—
Fever (in the absence of neutropenia, where neutropenia is defined as ANC <1.0 x 10^9/L)	Fever	38.0 – 39.0°C (100.4 – 102.2°F)	>39.0 – 40.0°C (102.3 – 104.0°F)	>40.0°C (>104.0°F) for ≤24 hrs	>40.0°C (>104.0°F) for >24 hrs	Death
REMARK: The temperature measurements listed are oral or tympanic.						
ALSO CONSIDER: Allergic reaction/hypersensitivity (including drug fever).						
NAVIGATION NOTE: Hot flashes are graded as Hot flashes/flushes in the ENDOCRINE CATEGORY.						
Hypothermia	Hypothermia	—	35 – >32°C<br>95 – >89.6°F	32 – >28°C<br>89.6 – >82.4° F	≤28 °C<br>82.4°F or life-threatening consequences (e.g., coma, hypotension, pulmonary edema, acidemia, ventricular fibrillation)	Death
Insomnia	Insomnia	Occasional difficulty sleeping, not interfering with function	Difficulty sleeping, interfering with function but not interfering with ADL	Frequent difficulty sleeping, interfering with ADL	Disabling	—
REMARK: If pain or other symptoms interfere with sleep, do NOT grade as insomnia. Grade primary event(s) causing insomnia.						
Obesity[2]	Obesity	—	BMI 25 – 29.9 kg/m^2	BMI 30 – 39.99 kg/m^2	BMI ≥40 kg/m^2	—
REMARK: BMI = (weight [kg]) / (height [m])^2						
Odor (patient odor)	Patient odor	Mild odor	Pronounced odor	—	—	—
Rigors/chills	Rigors/chills	Mild	Moderate, narcotics indicated	Severe or prolonged, not responsive to narcotics	—	—

[2] NHLBI Obesity Task Force. "Clinical Guidelines on the Identification, Evaluation, and Treatment of Overweight and Obesity in Adults," *The Evidence Report,* Obes Res 6:51S-209S, 1998.

CONSTITUTIONAL SYMPTOMS

		Grade				
**Adverse Event**	**Short Name**	**1**	**2**	**3**	**4**	**5**
Sweating (diaphoresis)	Sweating	Mild and occasional	Frequent or drenching	—	—	—
ALSO CONSIDER: Hot flashes/flushes.						
Weight gain	Weight gain	5 – <10% of baseline	10 – <20% of baseline	≥20% of baseline	—	—
REMARK: Edema, depending on etiology, is graded in the CARDIAC GENERAL or LYMPHATICS CATEGORIES.						
ALSO CONSIDER: Ascites (non-malignant); Pleural effusion (non-malignant).						
Weight loss	Weight loss	5 to <10% from baseline; intervention not indicated	10 – <20% from baseline; nutritional support indicated	≥20% from baseline; tube feeding or TPN indicated	—	—
Constitutional Symptoms – Other (Specify, __)	Constitutional Symptoms – Other (Specify)	Mild	Moderate	Severe	Life-threatening; disabling	Death

DEATH						Page 1 of 1
		Grade				
**Adverse Event**	**Short Name**	**1**	**2**	**3**	**4**	**5**
Death not associated with CTCAE term – *Select*: – Death NOS – Disease progression NOS – Multi-organ failure – Sudden death	Death not associated with CTCAE term – *Select*	—	—	—	—	Death
REMARK: Grade 5 is the only appropriate grade. 'Death not associated with CTCAE term – *Select'* is to be used where a death: 1. Cannot be attributed to a CTCAE term associated with Grade 5. 2. Cannot be reported within any CATEGORY using a CTCAE 'Other (Specify, __)'.						

DERMATOLOGY/SKIN

		Grade				
**Adverse Event**	**Short Name**	**1**	**2**	**3**	**4**	**5**
Atrophy, skin	Atrophy, skin	Detectable	Marked	—	—	—
Atrophy, subcutaneous fat	Atrophy, subcutaneous fat	Detectable	Marked	—	—	—
ALSO CONSIDER: Induration/fibrosis (skin and subcutaneous tissue).						
Bruising (in absence of Grade 3 or 4 thrombocytopenia)	Bruising	Localized or in a dependent area	Generalized	—	—	—
Burn	Burn	Minimal symptoms; intervention not indicated	Medical intervention; minimal debridement indicated	Moderate to major debridement or reconstruction indicated	Life-threatening consequences	Death
REMARK: Burn refers to all burns including radiation, chemical, etc.						
Cheilitis	Cheilitis	Asymptomatic	Symptomatic, not interfering with ADL	Symptomatic, interfering with ADL	—	—
Dry skin	Dry skin	Asymptomatic	Symptomatic, not interfering with ADL	Interfering with ADL	—	—
Flushing	Flushing	Asymptomatic	Symptomatic	—	—	—
Hair loss/alopecia (scalp or body)	Alopecia	Thinning or patchy	Complete	—	—	—
Hyperpigmentation	Hyperpigmentation	Slight or localized	Marked or generalized	—	—	—
Hypopigmentation	Hypopigmentation	Slight or localized	Marked or generalized	—	—	—
Induration/fibrosis (skin and subcutaneous tissue)	Induration	Increased density on palpation	Moderate impairment of function not interfering with ADL; marked increase in density and firmness on palpation with or without minimal retraction	Dysfunction interfering with ADL; very marked density, retraction or fixation	—	—
ALSO CONSIDER: Fibrosis-cosmesis; Fibrosis-deep connective tissue.						
Injection site reaction/ extravasation changes	Injection site reaction	Pain; itching; erythema	Pain or swelling, with inflammation or phlebitis	Ulceration or necrosis that is severe; operative intervention indicated	—	—
ALSO CONSIDER: Allergic reaction/hypersensitivity (including drug fever); Ulceration.						

		Grade				
**Adverse Event**	**Short Name**	**1**	**2**	**3**	**4**	**5**
Nail changes	Nail changes	Discoloration; ridging (koilonychias); pitting	Partial or complete loss of nail(s); pain in nailbed(s)	Interfering with ADL	—	—
NAVIGATION NOTE: Petechiae is graded as Petechiae/purpura (hemorrhage/bleeding into skin or mucosa) in the HEMORRHAGE/BLEEDING CATEGORY.						
Photosensitivity	Photosensitivity	Painless erythema	Painful erythema	Erythema with desquamation	Life-threatening; disabling	Death
Pruritus/itching	Pruritus	Mild or localized	Intense or widespread	Intense or widespread and interfering with ADL	—	—
ALSO CONSIDER: Rash/desquamation.						
Rash/desquamation	Rash	Macular or papular eruption or erythema without associated symptoms	Macular or papular eruption or erythema with pruritus or other associated symptoms; localized desquamation or other lesions covering <50% of body surface area (BSA)	Severe, generalized erythroderma or macular, papular or vesicular eruption; desquamation covering ≥50% BSA	Generalized exfoliative, ulcerative, or bullous dermatitis	Death
REMARK: Rash/desquamation may be used for GVHD.						
Rash: acne/acneiform	Acne	Intervention not indicated	Intervention indicated	Associated with pain, disfigurement, ulceration, or desquamation	—	Death
Rash: dermatitis associated with radiation – *Select*: – Chemoradiation – Radiation	Dermatitis – *Select*	Faint erythema or dry desquamation	Moderate to brisk erythema; patchy moist desquamation, mostly confined to skin folds and creases; moderate edema	Moist desquamation other than skin folds and creases; bleeding induced by minor trauma or abrasion	Skin necrosis or ulceration of full thickness dermis; spontaneous bleeding from involved site	Death
Rash: erythema multiforme (e.g., Stevens-Johnson syndrome, toxic epidermal necrolysis)	Erythema multiforme	—	Scattered, but not generalized eruption	Severe (e.g., generalized rash or painful stomatitis); IV fluids, tube feedings, or TPN indicated	Life-threatening; disabling	Death
Rash: hand-foot skin reaction	Hand-foot	Minimal skin changes or dermatitis (e.g., erythema) without pain	Skin changes (e.g., peeling, blisters, bleeding, edema) or pain, not interfering with function	Ulcerative dermatitis or skin changes with pain interfering with function	—	—

DERMATOLOGY/SKIN

		Grade				
**Adverse Event**	**Short Name**	**1**	**2**	**3**	**4**	**5**
Skin breakdown/ decubitus ulcer	Decubitus	—	Local wound care; medical intervention indicated	Operative debridement or other invasive intervention indicated (e.g., hyperbaric oxygen)	Life-threatening consequences; major invasive intervention indicated (e.g., tissue reconstruction, flap, or grafting)	Death
REMARK: Skin breakdown/decubitus ulcer is to be used for loss of skin integrity or decubitus ulcer from pressure or as the result of operative or medical intervention.						
Striae	Striae	Mild	Cosmetically significant	—	—	—
Telangiectasia	Telangiectasia	Few	Moderate number	Many and confluent	—	—
Ulceration	Ulceration	—	Superficial ulceration <2 cm size; local wound care; medical intervention indicated	Ulceration ≥2 cm size; operative debridement, primary closure or other invasive intervention indicated (e.g., hyperbaric oxygen)	Life-threatening consequences; major invasive intervention indicated (e.g., complete resection, tissue reconstruction, flap, or grafting)	Death
Urticaria (hives, welts, wheals)	Urticaria	Intervention not indicated	Intervention indicated for <24 hrs	Intervention indicated for ≥24 hrs	—	—
ALSO CONSIDER: Allergic reaction/hypersensitivity (including drug fever).						
Wound complication, non-infectious	Wound complication, non-infectious	Incisional separation of ≤25% of wound, no deeper than superficial fascia	Incisional separation >25% of wound with local care; asymptomatic hernia	Symptomatic hernia without evidence of strangulation; fascial disruption/dehiscence without evisceration; primary wound closure or revision by operative intervention indicated; hospitalization or hyperbaric oxygen indicated	Symptomatic hernia with evidence of strangulation; fascial disruption with evisceration; major reconstruction flap, grafting, resection, or amputation indicated	Death
REMARK: Wound complication, non-infectious is to be used for separation of incision, hernia, dehiscence, evisceration, or second surgery for wound revision.						
Dermatology/Skin – Other (Specify, __)	Dermatology – Other (Specify)	Mild	Moderate	Severe	Life-threatening; disabling	Death

		Grade				
**Adverse Event**	**Short Name**	**1**	**2**	**3**	**4**	**5**
Adrenal insufficiency	Adrenal insufficiency	Asymptomatic, intervention not indicated	Symptomatic, intervention indicated	Hospitalization	Life-threatening; disabling	Death
REMARK: Adrenal insufficiency includes any of the following signs and symptoms: abdominal pain, anorexia, constipation, diarrhea, hypotension, pigmentation of mucous membranes, pigmentation of skin, salt craving, syncope (fainting), vitiligo, vomiting, weakness, weight loss. Adrenal insufficiency must be confirmed by laboratory studies (low cortisol frequently accompanied by low aldosterone).<br>ALSO CONSIDER: Potassium, serum-high (hyperkalemia); Thyroid function, low (hypothyroidism).						
Cushingoid appearance (e.g., moon face, buffalo hump, centripetal obesity, cutaneous striae)	Cushingoid	—	Present	—	—	—
ALSO CONSIDER: Glucose, serum-high (hyperglycemia); Potassium, serum-low (hypokalemia).						
Feminization of male	Feminization of male	—	—	Present	—	—
NAVIGATION NOTE: Gynecomastia is graded in the SEXUAL/REPRODUCTIVE FUNCTION CATEGORY.						
Hot flashes/flushes[3]	Hot flashes	Mild	Moderate	Interfering with ADL	—	—
Masculinization of female	Masculinization of female	—	—	Present	—	—
Neuroendocrine: ACTH deficiency	ACTH	Asymptomatic	Symptomatic, not interfering with ADL; intervention indicated	Symptoms interfering with ADL; hospitalization indicated	Life-threatening consequences (e.g., severe hypotension)	Death
Neuroendocrine: ADH secretion abnormality (e.g., SIADH or low ADH)	ADH	Asymptomatic	Symptomatic, not interfering with ADL; intervention indicated	Symptoms interfering with ADL	Life-threatening consequences	Death
Neuroendocrine: gonadotropin secretion abnormality	Gonadotropin	Asymptomatic	Symptomatic, not interfering with ADL; intervention indicated	Symptoms interfering with ADL; osteopenia; fracture; infertility	—	—
Neuroendocrine: growth hormone secretion abnormality	Growth hormone	Asymptomatic	Symptomatic, not interfering with ADL; intervention indicated	—	—	—
Neuroendocrine: prolactin hormone secretion abnormality	Prolactin	Asymptomatic	Symptomatic, not interfering with ADL; intervention indicated	Symptoms interfering with ADL; amenorrhea; galactorrhea	—	Death

[3] Sloan JA, Loprinzi CL, Novotny PJ, Barton DL, Lavasseur BI, Windschitl HJ, "Methodologic Lessons Learned from Hot Flash Studies," *J Clin Oncol* 2001 Dec 1;19(23):4280-90

ENDOCRINE

		Grade				
**Adverse Event**	**Short Name**	**1**	**2**	**3**	**4**	**5**
Pancreatic endocrine: glucose intolerance	Diabetes	Asymptomatic, intervention not indicated	Symptomatic; dietary modification or oral agent indicated	Symptoms interfering with ADL; insulin indicated	Life-threatening consequences (e.g., ketoacidosis, hyperosmolar non-ketotic coma)	Death
Parathyroid function, low (hypoparathyroidism)	Hypoparathyroidism	Asymptomatic, intervention not indicated	Symptomatic; intervention indicated	—	—	—
Thyroid function, high (hyperthyroidism, thyrotoxicosis)	Hyperthyroidism	Asymptomatic, intervention not indicated	Symptomatic, not interfering with ADL; thyroid suppression therapy indicated	Symptoms interfering with ADL; hospitalization indicated	Life-threatening consequences (e.g., thyroid storm)	Death
Thyroid function, low (hypothyroidism)	Hypothyroidism	Asymptomatic, intervention not indicated	Symptomatic, not interfering with ADL; thyroid replacement indicated	Symptoms interfering with ADL; hospitalization indicated	Life-threatening myxedema coma	Death
Endocrine – Other (Specify, __)	Endocrine – Other (Specify)	Mild	Moderate	Severe	Life-threatening; disabling	Death

GASTROINTESTINAL						
		Grade				
**Adverse Event**	**Short Name**	**1**	**2**	**3**	**4**	**5**
NAVIGATION NOTE: Abdominal pain or cramping is graded as Pain – *Select* in the PAIN CATEGORY.						
Anorexia	Anorexia	Loss of appetite without alteration in eating habits	Oral intake altered without significant weight loss or malnutrition; oral nutritional supplements indicated	Associated with significant weight loss or malnutrition (e.g., inadequate oral caloric and/or fluid intake); IV fluids, tube feedings or TPN indicated	Life-threatening consequences	Death
ALSO CONSIDER: Weight loss.						
Ascites (non-malignant)	Ascites	Asymptomatic	Symptomatic, medical intervention indicated	Symptomatic, invasive procedure indicated	Life-threatening consequences	Death
REMARK: Ascites (non-malignant) refers to documented non-malignant ascites or unknown etiology, but unlikely malignant, and includes chylous ascites.						
Colitis	Colitis	Asymptomatic, pathologic or radiographic findings only	Abdominal pain; mucus or blood in stool	Abdominal pain, fever, change in bowel habits with ileus; peritoneal signs	Life-threatening consequences (e.g., perforation, bleeding, ischemia, necrosis, toxic megacolon)	Death
ALSO CONSIDER: Hemorrhage, GI – *Select*.						
Constipation	Constipation	Occasional or intermittent symptoms; occasional use of stool softeners, laxatives, dietary modification, or enema	Persistent symptoms with regular use of laxatives or enemas indicated	Symptoms interfering with ADL; obstipation with manual evacuation indicated	Life-threatening consequences (e.g., obstruction, toxic megacolon)	Death
ALSO CONSIDER: Ileus, GI (functional obstruction of bowel, i.e., neuroconstipation); Obstruction, GI – *Select*.						
Dehydration	Dehydration	Increased oral fluids indicated; dry mucous membranes; diminished skin turgor	IV fluids indicated <24 hrs	IV fluids indicated ≥24 hrs	Life-threatening consequences (e.g., hemodynamic collapse)	Death
ALSO CONSIDER: Diarrhea; Hypotension; Vomiting.						
Dental: dentures or prosthesis	Dentures	Minimal discomfort, no restriction in activities	Discomfort preventing use in some activities (e.g., eating), but not others (e.g., speaking)	Unable to use dentures or prosthesis at any time	—	—

		Grade				
**Adverse Event**	**Short Name**	**1**	**2**	**3**	**4**	**5**
Dental: periodontal disease	Periodontal	Gingival recession or gingivitis; limited bleeding on probing; mild local bone loss	Moderate gingival recession or gingivitis; multiple sites of bleeding on probing; moderate bone loss	Spontaneous bleeding; severe bone loss with or without tooth loss; osteonecrosis of maxilla or mandible	—	—
REMARK: Severe periodontal disease leading to osteonecrosis is graded as Osteonecrosis (avascular necrosis) in the MUSCULOSKELETAL CATEGORY.						
Dental: teeth	Teeth	Surface stains; dental caries; restorable, without extractions	Less than full mouth extractions; tooth fracture or crown amputation or repair indicated	Full mouth extractions indicated	—	—
Dental: teeth development	Teeth development	Hypoplasia of tooth or enamel not interfering with function	Functional impairment correctable with oral surgery	Maldevelopment with functional impairment not surgically correctable	—	—
Diarrhea	Diarrhea	Increase of <4 stools per day over baseline; mild increase in ostomy output compared to baseline	Increase of 4 – 6 stools per day over baseline; IV fluids indicated <24hrs; moderate increase in ostomy output compared to baseline; not interfering with ADL	Increase of ≥7 stools per day over baseline; incontinence; IV fluids ≥24 hrs; hospitalization; severe increase in ostomy output compared to baseline; interfering with ADL	Life-threatening consequences (e.g., hemodynamic collapse)	Death
REMARK: Diarrhea includes diarrhea of small bowel or colonic origin, and/or ostomy diarrhea.<br>ALSO CONSIDER: Dehydration; Hypotension.						
Distension/bloating, abdominal	Distension	Asymptomatic	Symptomatic, but not interfering with GI function	Symptomatic, interfering with GI function	—	—
ALSO CONSIDER: Ascites (non-malignant); Ileus, GI (functional obstruction of bowel, i.e., neuroconstipation); Obstruction, GI – *Select*.						

		Grade				
**Adverse Event**	**Short Name**	**1**	**2**	**3**	**4**	**5**
Dry mouth/salivary gland (xerostomia)	Dry mouth	Symptomatic (dry or thick saliva) without significant dietary alteration; unstimulated saliva flow >0.2 ml/min	Symptomatic and significant oral intake alteration (e.g., copious water, other lubricants, diet limited to purees and/or soft, moist foods); unstimulated saliva 0.1 to 0.2 ml/min	Symptoms leading to inability to adequately aliment orally; IV fluids, tube feedings, or TPN indicated; unstimulated saliva <0.1 ml/min	—	—
REMARK: Dry mouth/salivary gland (xerostomia) includes descriptions of grade using both subjective and objective assessment parameters. Record this event consistently throughout a patient's participation on study. If salivary flow measurements are used for initial assessment, subsequent assessments must use salivary flow. ALSO CONSIDER: Salivary gland changes/saliva.						
Dysphagia (difficulty swallowing)	Dysphagia	Symptomatic, able to eat regular diet	Symptomatic and altered eating/swallowing (e.g., altered dietary habits, oral supplements); IV fluids indicated <24 hrs	Symptomatic and severely altered eating/swallowing (e.g., inadequate oral caloric or fluid intake); IV fluids, tube feedings, or TPN indicated ≥24 hrs	Life-threatening consequences (e.g., obstruction, perforation)	Death
REMARK: Dysphagia (difficulty swallowing) is to be used for swallowing difficulty from oral, pharyngeal, esophageal, or neurologic origin. Dysphagia requiring dilation is graded as Stricture/stenosis (including anastomotic), GI – *Select*. ALSO CONSIDER: Dehydration; Esophagitis.						
Enteritis (inflammation of the small bowel)	Enteritis	Asymptomatic, pathologic or radiographic findings only	Abdominal pain; mucus or blood in stool	Abdominal pain, fever, change in bowel habits with ileus; peritoneal signs	Life-threatening consequences (e.g., perforation, bleeding, ischemia, necrosis)	Death
ALSO CONSIDER: Hemorrhage, GI – *Select*; Typhlitis (cecal inflammation).						
Esophagitis	Esophagitis	Asymptomatic pathologic, radiographic, or endoscopic findings only	Symptomatic; altered eating/swallowing (e.g., altered dietary habits, oral supplements); IV fluids indicated <24 hrs	Symptomatic and severely altered eating/swallowing (e.g., inadequate oral caloric or fluid intake); IV fluids, tube feedings, or TPN indicated ≥24 hrs	Life-threatening consequences	Death
REMARK: Esophagitis includes reflux esophagitis. ALSO CONSIDER: Dysphagia (difficulty swallowing).						

		Grade				
**Adverse Event**	**Short Name**	**1**	**2**	**3**	**4**	**5**
Fistula, GI – *Select*:<br>– Abdomen NOS<br>– Anus<br>– Biliary tree<br>– Colon/cecum/appendix<br>– Duodenum<br>– Esophagus<br>– Gallbladder<br>– Ileum<br>– Jejunum<br>– Oral cavity<br>– Pancreas<br>– Pharynx<br>– Rectum<br>– Salivary gland<br>– Small bowel NOS<br>– Stomach	Fistula, GI – *Select*	Asymptomatic, radiographic findings only	Symptomatic; altered GI function (e.g., altered dietary habits, diarrhea, or GI fluid loss); IV fluids indicated <24 hrs	Symptomatic and severely altered GI function (e.g., altered dietary habits, diarrhea, or GI fluid loss); IV fluids, tube feedings, or TPN indicated ≥24 hrs	Life-threatening consequences	Death
REMARK: A fistula is defined as an abnormal communication between two body cavities, potential spaces, and/or the skin. The site indicated for a fistula should be the site from which the abnormal process is believed to have originated. For example, a tracheo-esophageal fistula arising in the context of a resected or irradiated esophageal cancer is graded as Fistula, GI – esophagus.						
Flatulence	Flatulence	Mild	Moderate	—	—	—
Gastritis (including bile reflux gastritis)	Gastritis	Asymptomatic radiographic or endoscopic findings only	Symptomatic; altered gastric function (e.g., inadequate oral caloric or fluid intake); IV fluids indicated <24 hrs	Symptomatic and severely altered gastric function (e.g., inadequate oral caloric or fluid intake); IV fluids, tube feedings, or TPN indicated ≥24 hrs	Life-threatening consequences; operative intervention requiring complete organ resection (e.g., gastrectomy)	Death
ALSO CONSIDER: Hemorrhage, GI – *Select*; Ulcer, GI – *Select*.						
NAVIGATION NOTE: Head and neck soft tissue necrosis is graded as Soft tissue necrosis – *Select* in the MUSCULOSKELETAL/SOFT TISSUE CATEGORY.						
Heartburn/dyspepsia	Heartburn	Mild	Moderate	Severe	—	—
Hemorrhoids	Hemorrhoids	Asymptomatic	Symptomatic; banding or medical intervention indicated	Interfering with ADL; interventional radiology, endoscopic, or operative intervention indicated	Life-threatening consequences	Death

		Grade				
**Adverse Event**	**Short Name**	**1**	**2**	**3**	**4**	**5**
Ileus, GI (functional obstruction of bowel, i.e., neuroconstipation)	Ileus	Asymptomatic, radiographic findings only	Symptomatic; altered GI function (e.g., altered dietary habits); IV fluids indicated <24 hrs	Symptomatic and severely altered GI function; IV fluids, tube feeding, or TPN indicated ≥24 hrs	Life-threatening consequences	Death
REMARK: Ileus, GI is to be used for altered upper or lower GI function (e.g., delayed gastric or colonic emptying).						
ALSO CONSIDER: Constipation; Nausea; Obstruction, GI – *Select*; Vomiting.						
Incontinence, anal	Incontinence, anal	Occasional use of pads required	Daily use of pads required	Interfering with ADL; operative intervention indicated	Permanent bowel diversion indicated	Death
REMARK: Incontinence, anal is to be used for loss of sphincter control as sequelae of operative or therapeutic intervention.						
Leak (including anastomotic), GI – *Select*: – Biliary tree – Esophagus – Large bowel – Leak NOS – Pancreas – Pharynx – Rectum – Small bowel – Stoma – Stomach	Leak, GI – *Select*	Asymptomatic radiographic findings only	Symptomatic; medical intervention indicated	Symptomatic and interfering with GI function; invasive or endoscopic intervention indicated	Life-threatening consequences	Death
REMARK: Leak (including anasomotic), GI – *Select* is to be used for clinical signs/symptoms or radiographic confirmation of anastomotic or conduit leak (e.g., biliary, esophageal, intestinal, pancreatic, pharyngeal, rectal), but without development of fistula.						
Malabsorption	Malabsorption	—	Altered diet; oral therapies indicated (e.g., enzymes, medications, dietary supplements)	Inability to aliment adequately via GI tract (i.e., TPN indicated)	Life-threatening consequences	Death

		Grade				
**Adverse Event**	**Short Name**	**1**	**2**	**3**	**4**	**5**
Mucositis/stomatitis (clinical exam) – *Select*:<br>– Anus<br>– Esophagus<br>– Large bowel<br>– Larynx<br>– Oral cavity<br>– Pharynx<br>– Rectum<br>– Small bowel<br>– Stomach<br>– Trachea	Mucositis (clinical exam) – *Select*	Erythema of the mucosa	Patchy ulcerations or pseudomembranes	Confluent ulcerations or pseudomembranes; bleeding with minor trauma	Tissue necrosis; significant spontaneous bleeding; life-threatening consequences	Death
REMARK: Mucositis/stomatitis (functional/symptomatic) may be used for mucositis of the upper aero-digestive tract caused by radiation, agents, or GVHD.						
Mucositis/stomatitis (functional/symptomatic) – *Select*:<br>– Anus<br>– Esophagus<br>– Large bowel<br>– Larynx<br>– Oral cavity<br>– Pharynx<br>– Rectum<br>– Small bowel<br>– Stomach<br>– Trachea	Mucositis (functional/ symptomatic) – *Select*	Upper aerodigestive tract sites: Minimal symptoms, normal diet; minimal respiratory symptoms but not interfering with function<br><br>Lower GI sites: Minimal discomfort, intervention not indicated	Upper aerodigestive tract sites: Symptomatic but can eat and swallow modified diet; respiratory symptoms interfering with function but not interfering with ADL<br><br>Lower GI sites: Symptomatic, medical intervention indicated but not interfering with ADL	Upper aerodigestive tract sites: Symptomatic and unable to adequately aliment or hydrate orally; respiratory symptoms interfering with ADL<br><br>Lower GI sites: Stool incontinence or other symptoms interfering with ADL	Symptoms associated with life-threatening consequences	Death
Nausea	Nausea	Loss of appetite without alteration in eating habits	Oral intake decreased without significant weight loss, dehydration or malnutrition; IV fluids indicated <24 hrs	Inadequate oral caloric or fluid intake; IV fluids, tube feedings, or TPN indicated ≥24 hrs	Life-threatening consequences	Death
ALSO CONSIDER: Anorexia; Vomiting.						

		Grade				
**Adverse Event**	**Short Name**	**1**	**2**	**3**	**4**	**5**
Necrosis, GI – *Select*:<br>– Anus<br>– Colon/cecum/appendix<br>– Duodenum<br>– Esophagus<br>– Gallbladder<br>– Hepatic<br>– Ileum<br>– Jejunum<br>– Oral<br>– Pancreas<br>– Peritoneal cavity<br>– Pharynx<br>– Rectum<br>– Small bowel NOS<br>– Stoma<br>– Stomach	Necrosis, GI – *Select*	—	—	Inability to aliment adequately by GI tract (e.g., requiring enteral or parenteral nutrition); interventional radiology, endoscopic, or operative intervention indicated	Life-threatening consequences; operative intervention requiring complete organ resection (e.g., total colectomy)	Death
ALSO CONSIDER: Visceral arterial ischemia (non-myocardial).						
Obstruction, GI – *Select*:<br>– Cecum<br>– Colon<br>– Duodenum<br>– Esophagus<br>– Gallbladder<br>– Ileum<br>– Jejunum<br>– Rectum<br>– Small bowel NOS<br>– Stoma<br>– Stomach	Obstruction, GI – *Select*	Asymptomatic radiographic findings only	Symptomatic; altered GI function (e.g., altered dietary habits, vomiting, diarrhea, or GI fluid loss); IV fluids indicated <24 hrs	Symptomatic and severely altered GI function (e.g., altered dietary habits, vomiting, diarrhea, or GI fluid loss); IV fluids, tube feedings, or TPN indicated ≥24 hrs; operative intervention indicated	Life-threatening consequences; operative intervention requiring complete organ resection (e.g., total colectomy)	Death
NAVIGATION NOTE: Operative injury is graded as Intra-operative injury – *Select Organ or Structure* in the SURGERY/INTRA-OPERATIVE INJURY CATEGORY.						
NAVIGATION NOTE: Pelvic pain is graded as Pain – *Select* in the PAIN CATEGORY.						

		Grade				
**Adverse Event**	**Short Name**	**1**	**2**	**3**	**4**	**5**
Perforation, GI – *Select*:<br>– Appendix<br>– Biliary tree<br>– Cecum<br>– Colon<br>– Duodenum<br>– Esophagus<br>– Gallbladder<br>– Ileum<br>– Jejunum<br>– Rectum<br>– Small bowel NOS<br>– Stomach	Perforation, GI – *Select*	Asymptomatic radiographic findings only	Medical intervention indicated; IV fluids indicated <24 hrs	IV fluids, tube feedings, or TPN indicated ≥24 hrs; operative intervention indicated	Life-threatening consequences	Death
Proctitis	Proctitis	Rectal discomfort, intervention not indicated	Symptoms not interfering with ADL; medical intervention indicated	Stool incontinence or other symptoms interfering with ADL; operative intervention indicated	Life-threatening consequences (e.g., perforation)	Death
Prolapse of stoma, GI	Prolapse of stoma, GI	Asymptomatic	Extraordinary local care or maintenance; minor revision indicated	Dysfunctional stoma; major revision indicated	Life-threatening consequences	Death
REMARK: Other stoma complications may be graded as Fistula, GI – *Select*; Leak (including anastomotic), GI – *Select*; Obstruction, GI – *Select;* Perforation, GI – *Select*; Stricture/stenosis (including anastomotic), GI – *Select*.						
NAVIGATION NOTE: Rectal or perirectal pain (proctalgia) is graded as Pain – *Select* in the PAIN CATEGORY.						
Salivary gland changes/saliva	Salivary gland changes	Slightly thickened saliva; slightly altered taste (e.g., metallic)	Thick, ropy, sticky saliva; markedly altered taste; alteration in diet indicated; secretion-induced symptoms not interfering with ADL	Acute salivary gland necrosis; severe secretion-induced symptoms interfering with ADL	Disabling	—
ALSO CONSIDER: Dry mouth/salivary gland (xerostomia); Mucositis/stomatitis (clinical exam) – *Select*; Mucositis/stomatitis (functional/symptomatic) – *Select*; Taste alteration (dysgeusia).						
NAVIGATION NOTE: Splenic function is graded in the BLOOD/BONE MARROW CATEGORY.						

		Grade				
**Adverse Event**	**Short Name**	**1**	**2**	**3**	**4**	**5**
Stricture/stenosis (including anastomotic), GI – *Select*:<br>– Anus<br>– Biliary tree<br>– Cecum<br>– Colon<br>– Duodenum<br>– Esophagus<br>– Ileum<br>– Jejunum<br>– Pancreas/pancreatic duct<br>– Pharynx<br>– Rectum<br>– Small bowel NOS<br>– Stoma<br>– Stomach	Stricture, GI – *Select*	Asymptomatic radiographic findings only	Symptomatic; altered GI function (e.g., altered dietary habits, vomiting, bleeding, diarrhea); IV fluids indicated <24 hrs	Symptomatic and severely altered GI function (e.g., altered dietary habits, diarrhea, or GI fluid loss); IV fluids, tube feedings, or TPN indicated ≥24 hrs; operative intervention indicated	Life-threatening consequences; operative intervention requiring complete organ resection (e.g., total colectomy)	Death
Taste alteration (dysgeusia)	Taste alteration	Altered taste but no change in diet	Altered taste with change in diet (e.g., oral supplements); noxious or unpleasant taste; loss of taste	—	—	—
Typhlitis (cecal inflammation)	Typhlitis	Asymptomatic, pathologic or radiographic findings only	Abdominal pain; mucus or blood in stool	Abdominal pain, fever, change in bowel habits with ileus; peritoneal signs	Life-threatening consequences (e.g., perforation, bleeding, ischemia, necrosis); operative intervention indicated	Death

ALSO CONSIDER: Colitis; Hemorrhage, GI – *Select* ; Ileus, GI (functional obstruction of bowel, i.e., neuroconstipation).

		Grade				
**Adverse Event**	**Short Name**	**1**	**2**	**3**	**4**	**5**
Ulcer, GI<br>– *Select*:<br>– Anus<br>– Cecum<br>– Colon<br>– Duodenum<br>– Esophagus<br>– Ileum<br>– Jejunum<br>– Rectum<br>– Small bowel NOS<br>– Stoma<br>– Stomach	Ulcer, GI – *Select*	Asymptomatic, radiographic or endoscopic findings only	Symptomatic; altered GI function (e.g., altered dietary habits, oral supplements); IV fluids indicated <24 hrs	Symptomatic and severely altered GI function (e.g., inadequate oral caloric or fluid intake); IV fluids, tube feedings, or TPN indicated ≥24 hrs	Life-threatening consequences	Death
ALSO CONSIDER: Hemorrhage, GI – *Select*.						
Vomiting	Vomiting	1 episode in 24 hrs	2 – 5 episodes in 24 hrs; IV fluids indicated <24 hrs	≥6 episodes in 24 hrs; IV fluids, or TPN indicated ≥24 hrs	Life-threatening consequences	Death
ALSO CONSIDER: Dehydration.						
Gastrointestinal – Other (Specify, __)	GI – Other (Specify)	Mild	Moderate	Severe	Life-threatening; disabling	Death

GROWTH AND DEVELOPMENT

		Grade				
**Adverse Event**	**Short Name**	**1**	**2**	**3**	**4**	**5**
Bone age (alteration in bone age)	Bone age	—	±2 SD (standard deviation) from normal	—	—	—
Bone growth: femoral head; slipped capital femoral epiphysis	Femoral head growth	Mild valgus/varus deformity	Moderate valgus/varus deformity, symptomatic, interfering with function but not interfering with ADL	Mild slipped capital femoral epiphysis; operative intervention (e.g., fixation) indicated; interfering with ADL	Disabling; severe slipped capital femoral epiphysis >60%; avascular necrosis	—
Bone growth: limb length discrepancy	Limb length	Mild length discrepancy <2 cm	Moderate length discrepancy 2 – 5 cm; shoe lift indicated	Severe length discrepancy >5 cm; operative intervention indicated; interfering with ADL	Disabling; epiphysiodesis	—
Bone growth: spine kyphosis/lordosis	Kyphosis/lordosis	Mild radiographic changes	Moderate accentuation; interfering with function but not interfering with ADL	Severe accentuation; operative intervention indicated; interfering with ADL	Disabling (e.g., cannot lift head)	—
Growth velocity (reduction in growth velocity)	Reduction in growth velocity	10 – 29% reduction in growth from the baseline growth curve	30 – 49% reduction in growth from the baseline growth curve	≥50% reduction in growth from the baseline growth curve	—	—
Puberty (delayed)	Delayed puberty	—	No breast development by age 13 yrs for females; no Tanner Stage 2 development by age 14.5 yrs for males	No sexual development by age 14 yrs for girls, age 16 yrs for boys; hormone replacement indicated	—	—
REMARK: Do not use testicular size for Tanner Stage in male cancer survivors.						
Puberty (precocious)	Precocious puberty	—	Physical signs of puberty <7 years for females, <9 years for males	—	—	—
Short stature	Short stature	Beyond two standard deviations of age and gender mean height	Altered ADL	—	—	—
REMARK: Short stature is secondary to growth hormone deficiency.<br>ALSO CONSIDER: Neuroendocrine: growth hormone secretion abnormality.						
Growth and Development – Other (Specify, __)	Growth and Development – Other (Specify)	Mild	Moderate	Severe	Life-threatening; disabling	Death

HEMORRHAGE/BLEEDING						
		Grade				
**Adverse Event**	**Short Name**	**1**	**2**	**3**	**4**	**5**
Hematoma	Hematoma	Minimal symptoms, invasive intervention not indicated	Minimally invasive evacuation or aspiration indicated	Transfusion, interventional radiology, or operative intervention indicated	Life-threatening consequences; major urgent intervention indicated	Death
REMARK: Hematoma refers to extravasation at wound or operative site or secondary to other intervention. Transfusion implies pRBC.						
ALSO CONSIDER: Fibrinogen; INR (International Normalized Ratio of prothrombin time); Platelets; PTT (Partial Thromboplastin Time).						
Hemorrhage/bleeding associated with surgery, intra-operative or postoperative	Hemorrhage with surgery	—	—	Requiring transfusion of 2 units non-autologous (10 cc/kg for pediatrics) pRBCs beyond protocol specification; postoperative interventional radiology, endoscopic, or operative intervention indicated	Life-threatening consequences	Death
REMARK: Postoperative period is defined as ≤72 hours after surgery. Verify protocol-specific acceptable guidelines regarding pRBC transfusion.						
ALSO CONSIDER: Fibrinogen; INR (International Normalized Ratio of prothrombin time); Platelets; PTT (Partial Thromboplastin Time).						
Hemorrhage, CNS	CNS hemorrhage	Asymptomatic, radiographic findings only	Medical intervention indicated	Ventriculostomy, ICP monitoring, intraventricular thrombolysis, or operative intervention indicated	Life-threatening consequences; neurologic deficit or disability	Death
ALSO CONSIDER: Fibrinogen; INR (International Normalized Ratio of prothrombin time); Platelets; PTT (Partial Thromboplastin Time).						

		Grade				
**Adverse Event**	**Short Name**	**1**	**2**	**3**	**4**	**5**
Hemorrhage, GI – *Select*:<br>– Abdomen NOS<br>– Anus<br>– Biliary tree<br>– Cecum/appendix<br>– Colon<br>– Duodenum<br>– Esophagus<br>– Ileum<br>– Jejunum<br>– Liver<br>– Lower GI NOS<br>– Oral cavity<br>– Pancreas<br>– Peritoneal cavity<br>– Rectum<br>– Stoma<br>– Stomach<br>– Upper GI NOS<br>– Varices (esophageal)<br>– Varices (rectal)	Hemorrhage, GI – *Select*	Mild, intervention (other than iron supplements) not indicated	Symptomatic and medical intervention or minor cauterization indicated	Transfusion, interventional radiology, endoscopic, or operative intervention indicated; radiation therapy (i.e., hemostasis of bleeding site)	Life-threatening consequences; major urgent intervention indicated	Death
REMARK: Transfusion implies pRBC.						
ALSO CONSIDER: Fibrinogen; INR (International Normalized Ratio of prothrombin time); Platelets; PTT (Partial Thromboplastin Time).						

		Grade				
**Adverse Event**	**Short Name**	**1**	**2**	**3**	**4**	**5**
Hemorrhage, GU – *Select*:<br>– Bladder<br>– Fallopian tube<br>– Kidney<br>– Ovary<br>– Prostate<br>– Retroperitoneum<br>– Spermatic cord<br>– Stoma<br>– Testes<br>– Ureter<br>– Urethra<br>– Urinary NOS<br>– Uterus<br>– Vagina<br>– Vas deferens	Hemorrhage, GU – *Select*	Minimal or microscopic bleeding; intervention not indicated	Gross bleeding, medical intervention, or urinary tract irrigation indicated	Transfusion, interventional radiology, endoscopic, or operative intervention indicated; radiation therapy (i.e., hemostasis of bleeding site)	Life-threatening consequences; major urgent intervention indicated	Death
REMARK: Transfusion implies pRBC.						
ALSO CONSIDER: Fibrinogen; INR (International Normalized Ratio of prothrombin time); Platelets; PTT (Partial Thromboplastin Time).						
Hemorrhage, pulmonary/ upper respiratory – *Select*:<br>– Bronchopulmonary NOS<br>– Bronchus<br>– Larynx<br>– Lung<br>– Mediastinum<br>– Nose<br>– Pharynx<br>– Pleura<br>– Respiratory tract NOS<br>– Stoma<br>– Trachea	Hemorrhage pulmonary – *Select*	Mild, intervention not indicated	Symptomatic and medical intervention indicated	Transfusion, interventional radiology, endoscopic, or operative intervention indicated; radiation therapy (i.e., hemostasis of bleeding site)	Life-threatening consequences; major urgent intervention indicated	Death
REMARK: Transfusion implies pRBC.						
ALSO CONSIDER: Fibrinogen; INR (International Normalized Ratio of prothrombin time); Platelets; PTT (Partial Thromboplastin Time).						
Petechiae/purpura (hemorrhage/bleeding into skin or mucosa)	Petechiae	Few petechiae	Moderate petechiae; purpura	Generalized petechiae or purpura	—	—
ALSO CONSIDER: Fibrinogen; INR (International Normalized Ratio of prothrombin time); Platelets; PTT (Partial Thromboplastin Time).						

		Grade				
**Adverse Event**	**Short Name**	**1**	**2**	**3**	**4**	**5**
NAVIGATION NOTE: Vitreous hemorrhage is graded in the OCULAR/VISUAL CATEGORY.						
Hemorrhage/Bleeding – Other (Specify, __)	Hemorrhage – Other (Specify)	Mild without transfusion	—	Transfusion indicated	Catastrophic bleeding, requiring major non-elective intervention	Death

HEPATOBILIARY/PANCREAS

		Grade				
**Adverse Event**	**Short Name**	**1**	**2**	**3**	**4**	**5**
NAVIGATION NOTE: Biliary tree damage is graded as Fistula, GI – *Select*; Leak (including anastomotic), GI – *Select*; Necrosis, GI – *Select*; Obstruction, GI – *Select*; Perforation, GI – *Select*; Stricture/stenosis (including anastomotic), GI – *Select* in the GASTROINTESTINAL CATEGORY.						
Cholecystitis	Cholecystitis	Asymptomatic, radiographic findings only	Symptomatic, medical intervention indicated	Interventional radiology, endoscopic, or operative intervention indicated	Life-threatening consequences (e.g., sepsis or perforation)	Death
ALSO CONSIDER: Infection (documented clinically or microbiologically) with Grade 3 or 4 neutrophils – *Select*; Infection with normal ANC or Grade 1 or 2 neutrophils – *Select*; Infection with unknown ANC – *Select*.						
Liver dysfunction/failure (clinical)	Liver dysfunction	—	Jaundice	Asterixis	Encephalopathy or coma	Death
REMARK: Jaundice is not an AE, but occurs when the liver is not working properly or when a bile duct is blocked. It is graded as a result of liver dysfunction/failure or elevated bilirubin.<br>ALSO CONSIDER: Bilirubin (hyperbilirubinemia).						
Pancreas, exocrine enzyme deficiency	Pancreas, exocrine enzyme deficiency	—	Increase in stool frequency, bulk, or odor; steatorrhea	Sequelae of absorption deficiency (e.g., weight loss)	Life-threatening consequences	Death
ALSO CONSIDER: Diarrhea.						
Pancreatitis	Pancreatitis	Asymptomatic, enzyme elevation and/or radiographic findings	Symptomatic, medical intervention indicated	Interventional radiology or operative intervention indicated	Life-threatening consequences (e.g., circulatory failure, hemorrhage, sepsis)	Death
ALSO CONSIDER: Amylase.						
NAVIGATION NOTE: Stricture (biliary tree, hepatic or pancreatic) is graded as Stricture/stenosis (including anastomotic), GI – *Select* in the GASTROINTESTINAL CATEGORY.						
Hepatobiliary/Pancreas – Other (Specify, __)	Hepatobiliary – Other (Specify)	Mild	Moderate	Severe	Life-threatening; disabling	Death

INFECTION

		Grade				
**Adverse Event**	**Short Name**	**1**	**2**	**3**	**4**	**5**
Colitis, infectious (e.g., Clostridium difficile)	Colitis, infectious	Asymptomatic, pathologic or radiographic findings only	Abdominal pain with mucus and/or blood in stool	IV antibiotics or TPN indicated	Life-threatening consequences (e.g., perforation, bleeding, ischemia, necrosis or toxic megacolon); operative resection or diversion indicated	Death
ALSO CONSIDER: Hemorrhage, GI – *Select*; Typhlitis (cecal inflammation).						
Febrile neutropenia (fever of unknown origin without clinically or microbiologically documented infection) (ANC <1.0 x 10^9/L, fever ≥38.5°C)	Febrile neutropenia	—	—	Present	Life-threatening consequences (e.g., septic shock, hypotension, acidosis, necrosis)	Death
ALSO CONSIDER: Neutrophils/granulocytes (ANC/AGC).						
Infection (documented clinically or microbiologically) with Grade 3 or 4 neutrophils (ANC <1.0 x 10^9/L) – *Select*<br>*'Select'* AEs appear at the end of the CATEGORY.	Infection (documented clinically) with Grade 3 or 4 ANC – *Select*	—	Localized, local intervention indicated	IV antibiotic, antifungal, or antiviral intervention indicated; interventional radiology or operative intervention indicated	Life-threatening consequences (e.g., septic shock, hypotension, acidosis, necrosis)	Death
REMARK: Fever with Grade 3 or 4 neutrophils in the absence of documented infection is graded as Febrile neutropenia (fever of unknown origin without clinically or microbiologically documented infection).<br>ALSO CONSIDER: Neutrophils/granulocytes (ANC/AGC).						
Infection with normal ANC or Grade 1 or 2 neutrophils – *Select*<br>'*Select*' AEs appear at the end of the CATEGORY.	Infection with normal ANC – *Select*	—	Localized, local intervention indicated	IV antibiotic, antifungal, or antiviral intervention indicated; interventional radiology or operative intervention indicated	Life-threatening consequences (e.g., septic shock, hypotension, acidosis, necrosis)	Death

		Grade				
**Adverse Event**	**Short Name**	**1**	**2**	**3**	**4**	**5**
Infection with unknown ANC – *Select*<br>*'Select'* AEs appear at the end of the CATEGORY.	Infection with unknown ANC – *Select*	—	Localized, local intervention indicated	IV antibiotic, antifungal, or antiviral intervention indicated; interventional radiology or operative intervention indicated	Life-threatening consequences (e.g., septic shock, hypotension, acidosis, necrosis)	Death
REMARK: Infection with unknown ANC – *Select* is to be used in the rare case when ANC is unknown.						
Opportunistic infection associated with ≥Grade 2 Lymphopenia	Opportunistic infection	—	Localized, local intervention indicated	IV antibiotic, antifungal, or antiviral intervention indicated; interventional radiology or operative intervention indicated	Life-threatening consequences (e.g., septic shock, hypotension, acidosis, necrosis)	Death
ALSO CONSIDER: Lymphopenia.						
Viral hepatitis	Viral hepatitis	Present; transaminases and liver function normal	Transaminases abnormal, liver function normal	Symptomatic liver dysfunction; fibrosis by biopsy; compensated cirrhosis	Decompensated liver function (e.g., ascites, coagulopathy, encephalopathy, coma)	Death
REMARK: Non-viral hepatitis is graded as Infection – *Select*.<br>ALSO CONSIDER: Albumin, serum-low (hypoalbuminemia); ALT, SGPT (serum glutamic pyruvic transaminase); AST, SGOT (serum glutamic oxaloacetic transaminase); Bilirubin (hyperbilirubinemia); Encephalopathy.						
Infection – Other (Specify, __)	Infection – Other (Specify)	Mild	Moderate	Severe	Life-threatening; disabling	Death

INFECTION – *SELECT*

AUDITORY/EAR
- External ear (otitis externa)
- Middle ear (otitis media)

CARDIOVASCULAR
- Artery
- Heart (endocarditis)
- Spleen
- Vein

DERMATOLOGY/SKIN
- Lip/perioral
- Peristomal
- Skin (cellulitis)
- Ungual (nails)

GASTROINTESTINAL
- Abdomen NOS
- Anal/perianal
- Appendix
- Cecum
- Colon
- Dental-tooth
- Duodenum
- Esophagus
- Ileum
- Jejunum
- Oral cavity-gums (gingivitis)
- Peritoneal cavity
- Rectum
- Salivary gland
- Small bowel NOS
- Stomach

GENERAL
- Blood
- Catheter-related
- Foreign body (e.g., graft, implant, prosthesis, stent)
- Wound

HEPATOBILIARY/PANCREAS
- Biliary tree
- Gallbladder (cholecystitis)
- Liver
- Pancreas

LYMPHATIC
- Lymphatic

MUSCULOSKELETAL
- Bone (osteomyelitis)
- Joint
- Muscle (infection myositis)
- Soft tissue NOS

NEUROLOGY
- Brain (encephalitis, infectious)
- Brain + Spinal cord (encephalomyelitis)
- Meninges (meningitis)
- Nerve-cranial
- Nerve-peripheral
- Spinal cord (myelitis)

OCULAR
- Conjunctiva
- Cornea
- Eye NOS
- Lens

PULMONARY/UPPER RESPIRATORY
- Bronchus
- Larynx
- Lung (pneumonia)
- Mediastinum NOS
- Mucosa
- Neck NOS
- Nose
- Paranasal
- Pharynx
- Pleura (empyema)
- Sinus
- Trachea
- Upper aerodigestive NOS
- Upper airway NOS

RENAL/GENITOURINARY
- Bladder (urinary)
- Kidney
- Prostate
- Ureter
- Urethra
- Urinary tract NOS

SEXUAL/REPRODUCTIVE FUNCTION
- Cervix
- Fallopian tube
- Pelvis NOS
- Penis
- Scrotum
- Uterus
- Vagina
- Vulva

LYMPHATICS

		Grade				
**Adverse Event**	**Short Name**	**1**	**2**	**3**	**4**	**5**
Chyle or lymph leakage	Chyle or lymph leakage	Asymptomatic, clinical or radiographic findings	Symptomatic, medical intervention indicated	Interventional radiology or operative intervention indicated	Life-threatening complications	Death
ALSO CONSIDER: Chylothorax.						
Dermal change lymphedema, phlebolymphedema	Dermal change	Trace thickening or faint discoloration	Marked discoloration; leathery skin texture; papillary formation	—	—	—
REMARK: Dermal change lymphedema, phlebolymphedema refers to changes due to venous stasis.<br>ALSO CONSIDER: Ulceration.						
Edema: head and neck	Edema: head and neck	Localized to dependent areas, no disability or functional impairment	Localized facial or neck edema with functional impairment	Generalized facial or neck edema with functional impairment (e.g., difficulty in turning neck or opening mouth compared to baseline)	Severe with ulceration or cerebral edema; tracheotomy or feeding tube indicated	Death
Edema: limb	Edema: limb	5 – 10% inter-limb discrepancy in volume or circumference at point of greatest visible difference; swelling or obscuration of anatomic architecture on close inspection; pitting edema	>10 – 30% inter-limb discrepancy in volume or circumference at point of greatest visible difference; readily apparent obscuration of anatomic architecture; obliteration of skin folds; readily apparent deviation from normal anatomic contour	>30% inter-limb discrepancy in volume; lymphorrhea; gross deviation from normal anatomic contour; interfering with ADL	Progression to malignancy (i.e., lymphangiosarcoma); amputation indicated; disabling	Death
Edema: trunk/genital	Edema: trunk/genital	Swelling or obscuration of anatomic architecture on close inspection; pitting edema	Readily apparent obscuration of anatomic architecture; obliteration of skin folds; readily apparent deviation from normal anatomic contour	Lymphorrhea; interfering with ADL; gross deviation from normal anatomic contour	Progression to malignancy (i.e., lymphangiosarcoma); disabling	Death
Edema: viscera	Edema: viscera	Asymptomatic; clinical or radiographic findings only	Symptomatic; medical intervention indicated	Symptomatic and unable to aliment adequately orally; interventional radiology or operative intervention indicated	Life-threatening consequences	Death

LYMPHATICS						
		**Grade**				
**Adverse Event**	**Short Name**	**1**	**2**	**3**	**4**	**5**
Lymphedema-related fibrosis	Lymphedema-related fibrosis	Minimal to moderate redundant soft tissue, unresponsive to elevation or compression, with moderately firm texture or spongy feel	Marked increase in density and firmness, with or without tethering	Very marked density and firmness with tethering affecting ≥40% of the edematous area	—	—
Lymphocele	Lymphocele	Asymptomatic, clinical or radiographic findings only	Symptomatic; medical intervention indicated	Symptomatic and interventional radiology or operative intervention indicated	—	—
Phlebolymphatic cording	Phlebolymphatic cording	Asymptomatic, clinical findings only	Symptomatic; medical intervention indicated	Symptomatic and leading to contracture or reduced range of motion	—	—
Lymphatics – Other (Specify, __)	Lymphatics – Other (Specify)	Mild	Moderate	Severe	Life-threatening; disabling	Death

METABOLIC/LABORATORY

		Grade				
**Adverse Event**	**Short Name**	**1**	**2**	**3**	**4**	**5**
Acidosis (metabolic or respiratory)	Acidosis	pH <normal, but ≥7.3	—	pH <7.3	pH <7.3 with life-threatening consequences	Death
Albumin, serum-low (hypoalbuminemia)	Hypoalbuminemia	<LLN – 3 g/dL<br><LLN – 30 g/L	<3 – 2 g/dL<br><30 – 20 g/L	<2 g/dL<br><20 g/L	—	Death
Alkaline phosphatase	Alkaline phosphatase	>ULN – 2.5 x ULN	>2.5 – 5.0 x ULN	>5.0 – 20.0 x ULN	>20.0 x ULN	—
Alkalosis (metabolic or respiratory)	Alkalosis	pH >normal, but ≤7.5	—	pH >7.5	pH >7.5 with life-threatening consequences	Death
ALT, SGPT (serum glutamic pyruvic transaminase)	ALT	>ULN – 2.5 x ULN	>2.5 – 5.0 x ULN	>5.0 – 20.0 x ULN	>20.0 x ULN	—
Amylase	Amylase	>ULN – 1.5 x ULN	>1.5 – 2.0 x ULN	>2.0 – 5.0 x ULN	>5.0 x ULN	—
AST, SGOT (serum glutamic oxaloacetic transaminase)	AST	>ULN – 2.5 x ULN	>2.5 – 5.0 x ULN	>5.0 – 20.0 x ULN	>20.0 x ULN	—
Bicarbonate, serum-low	Bicarbonate, serum-low	<LLN – 16 mmol/L	<16 – 11 mmol/L	<11 – 8 mmol/L	<8 mmol/L	Death
Bilirubin (hyperbilirubinemia)	Bilirubin	>ULN – 1.5 x ULN	>1.5 – 3.0 x ULN	>3.0 – 10.0 x ULN	>10.0 x ULN	—
REMARK: Jaundice is not an AE, but may be a manifestation of liver dysfunction/failure or elevated bilirubin. If jaundice is associated with elevated bilirubin, grade bilirubin.						
Calcium, serum-low (hypocalcemia)	Hypocalcemia	<LLN – 8.0 mg/dL<br><LLN – 2.0 mmol/L<br><br>Ionized calcium:<br><LLN – 1.0 mmol/L	<8.0 – 7.0 mg/dL<br><2.0 – 1.75 mmol/L<br><br>Ionized calcium:<br><1.0 – 0.9 mmol/L	<7.0 – 6.0 mg/dL<br><1.75 – 1.5 mmol/L<br><br>Ionized calcium:<br><0.9 – 0.8 mmol/L	<6.0 mg/dL<br><1.5 mmol/L<br><br>Ionized calcium:<br><0.8 mmol/L	Death
REMARK: Calcium can be falsely low if hypoalbuminemia is present. Serum albumin is <4.0 g/dL, hypocalcemia is reported after the following corrective calculation has been performed: Corrected Calcium (mg/dL) = Total Calcium (mg/dL) – 0.8 [Albumin (g/dL) – 4][4]. Alternatively, direct measurement of ionized calcium is the definitive method to diagnose metabolically relevant alterations in serum calcium.						

[4] Crit Rev Clin Lab Sci 1984;21(1):51-97

METABOLIC/LABORATORY

		Grade				
**Adverse Event**	**Short Name**	**1**	**2**	**3**	**4**	**5**
Calcium, serum-high (hypercalcemia)	Hypercalcemia	>ULN – 11.5 mg/dL<br>>ULN – 2.9 mmol/L<br><br>Ionized calcium:<br>>ULN – 1.5 mmol/L	>11.5 – 12.5 mg/dL<br>>2.9 – 3.1 mmol/L<br><br>Ionized calcium:<br>>1.5 – 1.6 mmol/L	>12.5 – 13.5 mg/dL<br>>3.1 – 3.4 mmol/L<br><br>Ionized calcium:<br>>1.6 – 1.8 mmol/L	>13.5 mg/dL<br>>3.4 mmol/L<br><br>Ionized calcium:<br>>1.8 mmol/L	Death
Cholesterol, serum-high (hypercholesteremia)	Cholesterol	>ULN – 300 mg/dL<br>>ULN – 7.75 mmol/L	>300 – 400 mg/dL<br>>7.75 – 10.34 mmol/L	>400 – 500 mg/dL<br>>10.34 – 12.92 mmol/L	>500 mg/dL<br>>12.92 mmol/L	Death
CPK (creatine phosphokinase)	CPK	>ULN – 2.5 x ULN	>2.5 x ULN – 5 x ULN	>5 x ULN – 10 x ULN	>10 x ULN	Death
Creatinine	Creatinine	>ULN – 1.5 x ULN	>1.5 – 3.0 x ULN	>3.0 – 6.0 x ULN	>6.0 x ULN	Death
REMARK: Adjust to age-appropriate levels for pediatric patients.<br>ALSO CONSIDER: Glomerular filtration rate.						
GGT (γ-Glutamyl transpeptidase)	GGT	>ULN – 2.5 x ULN	>2.5 – 5.0 x ULN	>5.0 – 20.0 x ULN	>20.0 x ULN	—
Glomerular filtration rate	GFR	<75 – 50% LLN	<50 – 25% LLN	<25% LLN, chronic dialysis not indicated	Chronic dialysis or renal transplant indicated	Death
ALSO CONSIDER: Creatinine.						
Glucose, serum-high (hyperglycemia)	Hyperglycemia	>ULN – 160 mg/dL<br>>ULN – 8.9 mmol/L	>160 – 250 mg/dL<br>>8.9 – 13.9 mmol/L	>250 – 500 mg/dL<br>>13.9 – 27.8 mmol/L	>500 mg/dL<br>>27.8 mmol/L or acidosis	Death
REMARK: Hyperglycemia, in general, is defined as fasting unless otherwise specified in protocol.						
Glucose, serum-low (hypoglycemia)	Hypoglycemia	<LLN – 55 mg/dL<br><LLN – 3.0 mmol/L	<55 – 40 mg/dL<br><3.0 – 2.2 mmol/L	<40 – 30 mg/dL<br><2.2 – 1.7 mmol/L	<30 mg/dL<br><1.7 mmol/L	Death
Hemoglobinuria	Hemoglobinuria	Present	—	—	—	Death
Lipase	Lipase	>ULN – 1.5 x ULN	>1.5 – 2.0 x ULN	>2.0 – 5.0 x ULN	>5.0 x ULN	—
Magnesium, serum-high (hypermagnesemia)	Hypermagnesemia	>ULN – 3.0 mg/dL<br>>ULN – 1.23 mmol/L	—	>3.0 – 8.0 mg/dL<br>>1.23 – 3.30 mmol/L	>8.0 mg/dL<br>>3.30 mmol/L	Death
Magnesium, serum-low (hypomagnesemia)	Hypomagnesemia	<LLN – 1.2 mg/dL<br><LLN – 0.5 mmol/L	<1.2 – 0.9 mg/dL<br><0.5 – 0.4 mmol/L	<0.9 – 0.7 mg/dL<br><0.4 – 0.3 mmol/L	<0.7 mg/dL<br><0.3 mmol/L	Death
Phosphate, serum-low (hypophosphatemia)	Hypophosphatemia	<LLN – 2.5 mg/dL<br><LLN – 0.8 mmol/L	<2.5 – 2.0 mg/dL<br><0.8 – 0.6 mmol/L	<2.0 – 1.0 mg/dL<br><0.6 – 0.3 mmol/L	<1.0 mg/dL<br><0.3 mmol/L	Death
Potassium, serum-high (hyperkalemia)	Hyperkalemia	>ULN – 5.5 mmol/L	>5.5 – 6.0 mmol/L	>6.0 – 7.0 mmol/L	>7.0 mmol/L	Death

METABOLIC/LABORATORY

		Grade				
**Adverse Event**	**Short Name**	**1**	**2**	**3**	**4**	**5**
Potassium, serum-low (hypokalemia)	Hypokalemia	<LLN – 3.0 mmol/L	—	<3.0 – 2.5 mmol/L	<2.5 mmol/L	Death
Proteinuria	Proteinuria	1+ or<br>0.15 – 1.0 g/24 hrs	2+ to 3+ or<br>>1.0 – 3.5 g/24 hrs	4+ or<br>>3.5 g/24 hrs	Nephrotic syndrome	Death
Sodium, serum-high (hypernatremia)	Hypernatremia	>ULN – 150 mmol/L	>150 – 155 mmol/L	>155 – 160 mmol/L	>160 mmol/L	Death
Sodium, serum-low (hyponatremia)	Hyponatremia	<LLN – 130 mmol/L	—	<130 – 120 mmol/L	<120 mmol/L	Death
Triglyceride, serum-high (hypertriglyceridemia)	Hypertriglyceridemia	>ULN – 2.5 x ULN	>2.5 – 5.0 x ULN	>5.0 – 10 x ULN	>10 x ULN	Death
Uric acid, serum-high (hyperuricemia)	Hyperuricemia	>ULN – 10 mg/dL<br>≤0.59 mmol/L without physiologic consequences	—	>ULN – 10 mg/dL<br>≤0.59 mmol/L with physiologic consequences	>10 mg/dL<br>>0.59 mmol/L	Death
ALSO CONSIDER: Creatinine; Potassium, serum-high (hyperkalemia); Renal failure; Tumor lysis syndrome.						
Metabolic/Laboratory – Other (Specify, __)	Metabolic/Lab – Other (Specify)	Mild	Moderate	Severe	Life-threatening; disabling	Death

MUSCULOSKELETAL/SOFT TISSUE

		Grade				
**Adverse Event**	**Short Name**	**1**	**2**	**3**	**4**	**5**
Arthritis (non-septic)	Arthritis	Mild pain with inflammation, erythema, or joint swelling, but not interfering with function	Moderate pain with inflammation, erythema, or joint swelling interfering with function, but not interfering with ADL	Severe pain with inflammation, erythema, or joint swelling and interfering with ADL	Disabling	Death
REMARK: Report only when the diagnosis of arthritis (e.g., inflammation of a joint or a state characterized by inflammation of joints) is made. Arthralgia (sign or symptom of pain in a joint, especially non-inflammatory in character) is graded as Pain – *Select* in the PAIN CATEGORY.						
Bone: spine-scoliosis	Scoliosis	≤20 degrees; clinically undetectable	>20 – 45 degrees; visible by forward flexion; interfering with function but not interfering with ADL	>45 degrees; scapular prominence in forward flexion; operative intervention indicated; interfering with ADL	Disabling (e.g., interfering with cardiopulmonary function)	Death
Cervical spine-range of motion	Cervical spine ROM	Mild restriction of rotation or flexion between 60 – 70 degrees	Rotation <60 degrees to right or left; <60 degrees of flexion	Ankylosed/fused over multiple segments with no C-spine rotation	—	—
REMARK: 60 – 65 degrees of rotation is required for reversing a car; 60 – 65 degrees of flexion is required to tie shoes.						
Exostosis	Exostosis	Asymptomatic	Involving multiple sites; pain or interfering with function	Excision indicated	Progression to malignancy (i.e., chondrosarcoma)	Death
Extremity-lower (gait/walking)	Gait/walking	Limp evident only to trained observer and able to walk ≥1 kilometer; cane indicated for walking	Noticeable limp, or limitation of limb function, but able to walk ≥0.1 kilometer (1 city block); quad cane indicated for walking	Severe limp with stride modified to maintain balance (widened base of support, marked reduction in step length); ambulation limited to walker; crutches indicated	Unable to walk	—
ALSO CONSIDER: Ataxia (incoordination); Muscle weakness, generalized or specific area (not due to neuropathy) – *Select*.						
Extremity-upper (function)	Extremity-upper (function)	Able to perform most household or work activities with affected limb	Able to perform most household or work activities with compensation from unaffected limb	Interfering with ADL	Disabling; no function of affected limb	—
Fibrosis-cosmesis	Fibrosis-cosmesis	Visible only on close examination	Readily apparent but not disfiguring	Significant disfigurement; operative intervention indicated if patient chooses	—	—

MUSCULOSKELETAL/SOFT TISSUE

		Grade				
**Adverse Event**	**Short Name**	**1**	**2**	**3**	**4**	**5**
Fibrosis-deep connective tissue	Fibrosis-deep connective tissue	Increased density, "spongy" feel	Increased density with firmness or tethering	Increased density with fixation of tissue; operative intervention indicated; interfering with ADL	Life-threatening; disabling; loss of limb; interfering with vital organ function	Death
ALSO CONSIDER: Induration/fibrosis (skin and subcutaneous tissue); Muscle weakness, generalized or specific area (not due to neuropathy) – *Select*; Neuropathy: motor; Neuropathy: sensory.						
Fracture	Fracture	Asymptomatic, radiographic findings only (e.g., asymptomatic rib fracture on plain x-ray, pelvic insufficiency fracture on MRI, etc.)	Symptomatic but non-displaced; immobilization indicated	Symptomatic and displaced or open wound with bone exposure; operative intervention indicated	Disabling; amputation indicated	Death
Joint-effusion	Joint-effusion	Asymptomatic, clinical or radiographic findings only	Symptomatic; interfering with function but not interfering with ADL	Symptomatic and interfering with ADL	Disabling	Death
ALSO CONSIDER: Arthritis (non-septic).						
Joint-function[5]	Joint-function	Stiffness interfering with athletic activity; ≤25% loss of range of motion (ROM)	Stiffness interfering with function but not interfering with ADL; >25 – 50% decrease in ROM	Stiffness interfering with ADL; >50 – 75% decrease in ROM	Fixed or non-functional joint (arthrodesis); >75% decrease in ROM	—
ALSO CONSIDER: Arthritis (non-septic).						
Local complication – device/prosthesis-related	Device/prosthesis	Asymptomatic	Symptomatic, but not interfering with ADL; local wound care; medical intervention indicated	Symptomatic, interfering with ADL; operative intervention indicated (e.g., hardware/device replacement or removal, reconstruction)	Life-threatening; disabling; loss of limb or organ	Death
Lumbar spine-range of motion	Lumbar spine ROM	Stiffness and difficulty bending to the floor to pick up a very light object but able to do activity	Some lumbar spine flexion but requires a reaching aid to pick up a very light object from the floor	Ankylosed/fused over multiple segments with no L-spine flexion (i.e., unable to reach to floor to pick up a very light	—	—

[5] Adapted from the *International SFTR Method of Measuring and Recording Joint Motion, International Standard Orthopedic Measurements (ISOM),* Jon J. Gerhardt and Otto A. Russee, Bern, Switzerland, Han Huber 9 Publisher), 1975.

		Grade				
**Adverse Event**	**Short Name**	**1**	**2**	**3**	**4**	**5**
				object)		
Muscle weakness, generalized or specific area (not due to neuropathy)<br>– *Select*:<br>– Extraocular<br>– Extremity-lower<br>– Extremity-upper<br>– Facial<br>– Left-sided<br>– Ocular<br>– Pelvic<br>– Right-sided<br>– Trunk<br>– Whole body/generalized	Muscle weakness – *Select*	Asymptomatic, weakness on physical exam	Symptomatic and interfering with function, but not interfering with ADL	Symptomatic and interfering with ADL	Life-threatening; disabling	Death
ALSO CONSIDER: Fatigue (asthenia, lethargy, malaise).						
Muscular/skeletal hypoplasia	Muscular/skeletal hypoplasia	Cosmetically and functionally insignificant hypoplasia	Deformity, hypoplasia, or asymmetry able to be remediated by prosthesis (e.g., shoe insert) or covered by clothing	Functionally significant deformity, hypoplasia, or asymmetry, unable to be remediated by prosthesis or covered by clothing	Disabling	—
Myositis (inflammation/damage of muscle)	Myositis	Mild pain, not interfering with function	Pain interfering with function, but not interfering with ADL	Pain interfering with ADL	Disabling	Death
REMARK: Myositis implies muscle damage (i.e., elevated CPK).<br>ALSO CONSIDER: CPK (creatine phosphokinase); Pain – *Select*.						
Osteonecrosis (avascular necrosis)	Osteonecrosis	Asymptomatic, radiographic findings only	Symptomatic and interfering with function, but not interfering with ADL; minimal bone removal indicated (i.e., minor sequestrectomy)	Symptomatic and interfering with ADL; operative intervention or hyperbaric oxygen indicated	Disabling	Death

MUSCULOSKELETAL/SOFT TISSUE

		Grade				
**Adverse Event**	**Short Name**	**1**	**2**	**3**	**4**	**5**
Osteoporosis[6]	Osteoporosis	Radiographic evidence of osteoporosis or Bone Mineral Density (BMD) t-score –1 to –2.5 (osteopenia) and no loss of height or therapy indicated	BMD t-score < –2.5; loss of height <2 cm; anti-osteoporotic therapy indicated	Fractures; loss of height ≥2 cm	Disabling	Death
Seroma	Seroma	Asymptomatic	Symptomatic; medical intervention or simple aspiration indicated	Symptomatic, interventional radiology or operative intervention indicated	—	—
Soft tissue necrosis – *Select*:<br>– Abdomen<br>– Extremity-lower<br>– Extremity-upper<br>– Head<br>– Neck<br>– Pelvic<br>– Thorax	Soft tissue necrosis – *Select*	—	Local wound care; medical intervention indicated	Operative debridement or other invasive intervention indicated (e.g., hyperbaric oxygen)	Life-threatening consequences; major invasive intervention indicated (e.g., tissue reconstruction, flap, or grafting)	Death
Trismus (difficulty, restriction or pain when opening mouth)	Trismus	Decreased range of motion without impaired eating	Decreased range of motion requiring small bites, soft foods or purees	Decreased range of motion with inability to adequately aliment or hydrate orally	—	—
NAVIGATION NOTE: Wound-infectious is graded as Infection – *Select* in the INFECTION CATEGORY.						
NAVIGATION NOTE: Wound non-infectious is graded as Wound complication, non-infectious in the DERMATOLOGY/SKIN CATEGORY.						
Musculoskeletal/Soft Tissue – Other (Specify, __)	Musculoskeletal – Other (Specify)	Mild	Moderate	Severe	Life-threatening; disabling	Death

[6] "Assessment of Fracture Risk and its Application to Screening for Postmenopausal Osteoporosis," Report of a *WHO Study Group Technical Report Series*, No. 843, 1994, v + 129 pages [C*, E, F, R, S], ISBN 92 4 120843 0, Sw.fr. 22.-/US $19.80; in developing countries: Sw.fr. 15.40, Order no. 1100843

NEUROLOGY

		Grade				
**Adverse Event**	**Short Name**	**1**	**2**	**3**	**4**	**5**
NAVIGATION NOTE: ADD (Attention Deficit Disorder) is graded as Cognitive disturbance.						
NAVIGATION NOTE: Aphasia, receptive and/or expressive, is graded as Speech impairment (e.g., dysphasia or aphasia).						
Apnea	Apnea	—	—	Present	Intubation indicated	Death
Arachnoiditis/ meningismus/radiculitis	Arachnoiditis	Symptomatic, not interfering with function; medical intervention indicated	Symptomatic (e.g., photophobia, nausea) interfering with function but not interfering with ADL	Symptomatic, interfering with ADL	Life-threatening; disabling (e.g., paraplegia)	Death
ALSO CONSIDER: Fever (in the absence of neutropenia, where neutropenia is defined as ANC <1.0 x 10^9/L); Infection (documented clinically or microbiologically) with Grade 3 or 4 neutrophils (ANC <1.0 x 109/L) – *Select*; Infection with normal ANC or Grade 1 or 2 neutrophils – *Select*; Infection with unknown ANC – *Select*; Pain – *Select*; Vomiting.						
Ataxia (incoordination)	Ataxia	Asymptomatic	Symptomatic, not interfering with ADL	Symptomatic, interfering with ADL; mechanical assistance indicated	Disabling	Death
REMARK: Ataxia (incoordination) refers to the consequence of medical or operative intervention.						
Brachial plexopathy	Brachial plexopathy	Asymptomatic	Symptomatic, not interfering with ADL	Symptomatic, interfering with ADL	Disabling	Death
CNS cerebrovascular ischemia	CNS ischemia	—	Asymptomatic, radiographic findings only	Transient ischemic event or attack (TIA) ≤24 hrs duration	Cerebral vascular accident (CVA, stroke), neurologic deficit >24 hrs	Death
NAVIGATION NOTE: CNS hemorrhage/bleeding is graded as Hemorrhage, CNS in the HEMORRHAGE/BLEEDING CATEGORY.						
CNS necrosis/cystic progression	CNS necrosis	Asymptomatic, radiographic findings only	Symptomatic, not interfering with ADL; medical intervention indicated	Symptomatic and interfering with ADL; hyperbaric oxygen indicated	Life-threatening; disabling; operative intervention indicated to prevent or treat CNS necrosis/cystic progression	Death
Cognitive disturbance	Cognitive disturbance	Mild cognitive disability; not interfering with work/school/life performance; specialized educational services/devices not indicated	Moderate cognitive disability; interfering with work/school/life performance but capable of independent living; specialized resources on part-time basis indicated	Severe cognitive disability; significant impairment of work/school/life performance	Unable to perform ADL; full-time specialized resources or institutionalization indicated	Death
REMARK: Cognitive disturbance may be used for Attention Deficit Disorder (ADD).						

		Grade				
**Adverse Event**	**Short Name**	**1**	**2**	**3**	**4**	**5**
Confusion	Confusion	Transient confusion, disorientation, or attention deficit	Confusion, disorientation, or attention deficit interfering with function, but not interfering with ADL	Confusion or delirium interfering with ADL	Harmful to others or self; hospitalization indicated	Death
REMARK: Attention Deficit Disorder (ADD) is graded as Cognitive disturbance.						
NAVIGATION NOTE: Cranial neuropathy is graded as Neuropathy-cranial – *Select*.						
Dizziness	Dizziness	With head movements or nystagmus only; not interfering with function	Interfering with function, but not interfering with ADL	Interfering with ADL	Disabling	—
REMARK: Dizziness includes disequilibrium, lightheadedness, and vertigo.<br>ALSO CONSIDER: Neuropathy: cranial – *Select*; Syncope (fainting).						
NAVIGATION NOTE: Dysphasia, receptive and/or expressive, is graded as Speech impairment (e.g., dysphasia or aphasia).						
Encephalopathy	Encephalopathy	—	Mild signs or symptoms; not interfering with ADL	Signs or symptoms interfering with ADL; hospitalization indicated	Life-threatening; disabling	Death
ALSO CONSIDER: Cognitive disturbance; Confusion; Dizziness; Memory impairment; Mental status; Mood alteration – *Select*; Psychosis (hallucinations/delusions); Somnolence/depressed level of consciousness.						
Extrapyramidal/ involuntary movement/ restlessness	Involuntary movement	Mild involuntary movements not interfering with function	Moderate involuntary movements interfering with function, but not interfering with ADL	Severe involuntary movements or torticollis interfering with ADL	Disabling	Death
NAVIGATION NOTE: Headache/neuropathic pain (e.g., jaw pain, neurologic pain, phantom limb pain, post-infectious neuralgia, or painful neuropathies) is graded as Pain – *Select* in the PAIN CATEGORY.						
Hydrocephalus	Hydrocephalus	Asymptomatic, radiographic findings only	Mild to moderate symptoms not interfering with ADL	Severe symptoms or neurological deficit interfering with ADL	Disabling	Death
Irritability (children <3 years of age)	Irritability	Mild; easily consolable	Moderate; requiring increased attention	Severe; inconsolable	—	—
Laryngeal nerve dysfunction	Laryngeal nerve	Asymptomatic, weakness on clinical examination/testing only	Symptomatic, but not interfering with ADL; intervention not indicated	Symptomatic, interfering with ADL; intervention indicated (e.g., thyroplasty, vocal cord injection)	Life-threatening; tracheostomy indicated	Death

		Grade				
**Adverse Event**	**Short Name**	**1**	**2**	**3**	**4**	**5**
Leak, cerebrospinal fluid (CSF)	CSF leak	Transient headache; postural care indicated	Symptomatic, not interfering with ADL; blood patch indicated	Symptomatic, interfering with ADL; operative intervention indicated	Life-threatening; disabling	Death
REMARK: Leak, cerebrospinal fluid (CSF) may be used for CSF leak associated with operation and persisting >72 hours.						
Leukoencephalopathy (radiographic findings)	Leukoencephalopathy	Mild increase in subarachnoid space (SAS); mild ventriculomegaly; small (+/- multiple) focal T2 hyperintensities, involving periventricular white matter or <1/3 of susceptible areas of cerebrum	Moderate increase in SAS; moderate ventriculomegaly; focal T2 hyperintensities extending into centrum ovale or involving 1/3 to 2/3 of susceptible areas of cerebrum	Severe increase in SAS; severe ventriculomegaly; near total white matter T2 hyperintensities or diffuse low attenuation (CT)	—	—
REMARK: Leukoencephalopathy is a diffuse white matter process, specifically NOT associated with necrosis. Leukoencephalopathy (radiographic findings) does not include lacunas, which are areas that become void of neural tissue.						
Memory impairment	Memory impairment	Memory impairment not interfering with function	Memory impairment interfering with function, but not interfering with ADL	Memory impairment interfering with ADL	Amnesia	—
Mental status[7]	Mental status	—	1 – 3 point below age and educational norm in Folstein Mini-Mental Status Exam (MMSE)	>3 point below age and educational norm in Folstein MMSE	—	—
Mood alteration – *Select*: – Agitation – Anxiety – Depression – Euphoria	Mood alteration – *Select*	Mild mood alteration not interfering with function	Moderate mood alteration interfering with function, but not interfering with ADL; medication indicated	Severe mood alteration interfering with ADL	Suicidal ideation; danger to self or others	Death
Myelitis	Myelitis	Asymptomatic, mild signs (e.g., Babinski's or Lhermitte's sign)	Weakness or sensory loss not interfering with ADL	Weakness or sensory loss interfering with ADL	Disabling	Death

[7] Folstein MF, Folstein, SE and McHugh PR (1975) "Mini-Mental State: A Practical Method for Grading the State of Patients for the Clinician," *Journal of Psychiatric Research*, 12: 189-198

		Grade				
**Adverse Event**	**Short Name**	**1**	**2**	**3**	**4**	**5**
NAVIGATION NOTE: Neuropathic pain is graded as Pain – *Select* in the PAIN CATEGORY.						
Neuropathy: cranial – *Select*:<br>– CN I Smell<br>– CN II Vision<br>– CN III Pupil, upper eyelid, extra ocular movements<br>– CN IV Downward, inward movement of eye<br>– CN V Motor-jaw muscles; Sensory-facial<br>– CN VI Lateral deviation of eye<br>– CN VII Motor-face; Sensory-taste<br>– CN VIII Hearing and balance<br>– CN IX Motor-pharynx; Sensory-ear, pharynx, tongue<br>– CN X Motor-palate; pharynx, larynx<br>– CN XI Motor-sternomastoid and trapezius<br>– CN XII Motor-tongue	Neuropathy: cranial – *Select*	Asymptomatic, detected on exam/testing only	Symptomatic, not interfering with ADL	Symptomatic, interfering with ADL	Life-threatening; disabling	Death
Neuropathy: motor	Neuropathy-motor	Asymptomatic, weakness on exam/testing only	Symptomatic weakness interfering with function, but not interfering with ADL	Weakness interfering with ADL; bracing or assistance to walk (e.g., cane or walker) indicated	Life-threatening; disabling (e.g., paralysis)	Death
REMARK: Cranial nerve motor neuropathy is graded as Neuropathy: cranial – *Select*.<br>ALSO CONSIDER: Laryngeal nerve dysfunction; Phrenic nerve dysfunction.						
Neuropathy: sensory	Neuropathy-sensory	Asymptomatic; loss of deep tendon reflexes or paresthesia (including tingling) but not interfering with function	Sensory alteration or paresthesia (including tingling), interfering with function, but not interfering with ADL	Sensory alteration or paresthesia interfering with ADL	Disabling	Death
REMARK: Cranial nerve sensory neuropathy is graded as Neuropathy: cranial – *Select*.						
Personality/behavioral	Personality	Change, but not adversely affecting patient or family	Change, adversely affecting patient or family	Mental health intervention indicated	Change harmful to others or self; hospitalization indicated	Death
Phrenic nerve dysfunction	Phrenic nerve	Asymptomatic weakness on exam/testing only	Symptomatic but not interfering with ADL; intervention not indicated	Significant dysfunction; intervention indicated (e.g., diaphragmatic plication)	Life-threatening respiratory compromise; mechanical ventilation indicated	Death
Psychosis (hallucinations/ delusions)	Psychosis	—	Transient episode	Interfering with ADL; medication, supervision	Harmful to others or self; life-threatening	Death

		Grade				
**Adverse Event**	**Short Name**	**1**	**2**	**3**	**4**	**5**
				or restraints indicated	consequences	
Pyramidal tract dysfunction (e.g., ↑ tone, hyperreflexia, positive Babinski, ↓ fine motor coordination)	Pyramidal tract dysfunction	Asymptomatic, abnormality on exam or testing only	Symptomatic; interfering with function but not interfering with ADL	Interfering with ADL	Disabling; paralysis	Death
Seizure	Seizure	—	One brief generalized seizure; seizure(s) well controlled by anticonvulsants or infrequent focal motor seizures not interfering with ADL	Seizures in which consciousness is altered; poorly controlled seizure disorder, with breakthrough generalized seizures despite medical intervention	Seizures of any kind which are prolonged, repetitive, or difficult to control (e.g., status epilepticus, intractable epilepsy)	Death
Somnolence/depressed level of consciousness	Somnolence	—	Somnolence or sedation interfering with function, but not interfering with ADL	Obtundation or stupor; difficult to arouse; interfering with ADL	Coma	Death
Speech impairment (e.g., dysphasia or aphasia)	Speech impairment	—	Awareness of receptive or expressive dysphasia, not impairing ability to communicate	Receptive or expressive dysphasia, impairing ability to communicate	Inability to communicate	—
REMARK: Speech impairment refers to a primary CNS process, not neuropathy or end organ dysfunction.<br>ALSO CONSIDER: Laryngeal nerve dysfunction; Voice changes/dysarthria (e.g., hoarseness, loss, or alteration in voice, laryngitis).						
Syncope (fainting)	Syncope (fainting)	—	—	Present	Life-threatening consequences	Death
ALSO CONSIDER: CNS cerebrovascular ischemia; Conduction abnormality/atrioventricular heart block – *Select*; Dizziness; Supraventricular and nodal arrhythmia – *Select*; Vasovagal episode; Ventricular arrhythmia – *Select*.						
NAVIGATION NOTE: Taste alteration (CN VII, IX) is graded as Taste alteration (dysgeusia) in the GASTROINTESTINAL CATEGORY.						
Tremor	Tremor	Mild and brief or intermittent but not interfering with function	Moderate tremor interfering with function, but not interfering with ADL	Severe tremor interfering with ADL	Disabling	—
Neurology – Other (Specify, __)	Neurology – Other (Specify)	Mild	Moderate	Severe	Life-threatening; disabling	Death

OCULAR/VISUAL

		Grade				
**Adverse Event**	**Short Name**	**1**	**2**	**3**	**4**	**5**
Cataract	Cataract	Asymptomatic, detected on exam only	Symptomatic, with moderate decrease in visual acuity (20/40 or better); decreased visual function correctable with glasses	Symptomatic with marked decrease in visual acuity (worse than 20/40); operative intervention indicated (e.g., cataract surgery)	—	—
Dry eye syndrome	Dry eye	Mild, intervention not indicated	Symptomatic, interfering with function but not interfering with ADL; medical intervention indicated	Symptomatic or decrease in visual acuity interfering with ADL; operative intervention indicated	—	—
Eyelid dysfunction	Eyelid dysfunction	Asymptomatic	Symptomatic, interfering with function but not ADL; requiring topical agents or epilation	Symptomatic; interfering with ADL; surgical intervention indicated	—	—
REMARK: Eyelid dysfunction includes canalicular stenosis, ectropion, entropion, erythema, madarosis, symblepharon, telangiectasis, thickening, and trichiasis.<br>ALSO CONSIDER: Neuropathy: cranial – *Select*.						
Glaucoma	Glaucoma	Elevated intraocular pressure (EIOP) with single topical agent for intervention; no visual field deficit	EIOP causing early visual field deficit (i.e., nasal step or arcuate deficit); multiple topical or oral agents indicated	EIOP causing marked visual field deficits (i.e., involving both superior and inferior visual fields); operative intervention indicated	EIOP resulting in blindness (20/200 or worse); enucleation indicated	—
Keratitis (corneal inflammation/corneal ulceration)	Keratitis	Abnormal ophthalmologic changes only; intervention not indicated	Symptomatic and interfering with function, but not interfering with ADL	Symptomatic and interfering with ADL; operative intervention indicated	Perforation or blindness (20/200 or worse)	—
NAVIGATION NOTE: Ocular muscle weakness is graded as Muscle weakness, generalized or specific area (not due to neuropathy) – *Select* in the MUSCULOSKELETAL/SOFT TISSUE CATEGORY.						
Night blindness (nyctalopia)	Nyctalopia	Symptomatic, not interfering with function	Symptomatic and interfering with function but not interfering with ADL	Symptomatic and interfering with ADL	Disabling	—

		Grade				
**Adverse Event**	**Short Name**	**1**	**2**	**3**	**4**	**5**
Nystagmus	Nystagmus	Asymptomatic	Symptomatic and interfering with function but not interfering with ADL	Symptomatic and interfering with ADL	Disabling	—
ALSO CONSIDER: Neuropathy: cranial – *Select*; Ophthalmoplegia/diplopia (double vision).						
Ocular surface disease	Ocular surface disease	Asymptomatic or minimally symptomatic but not interfering with function	Symptomatic, interfering with function but not interfering with ADL; topical antibiotics or other topical intervention indicated	Symptomatic, interfering with ADL; operative intervention indicated	—	—
REMARK: Ocular surface disease includes conjunctivitis, keratoconjunctivitis sicca, chemosis, keratinization, and palpebral conjunctival epithelial metaplasia.						
Ophthalmoplegia/ diplopia (double vision)	Diplopia	Intermittently symptomatic, intervention not indicated	Symptomatic and interfering with function but not interfering with ADL	Symptomatic and interfering with ADL; surgical intervention indicated	Disabling	—
ALSO CONSIDER: Neuropathy: cranial – *Select*.						
Optic disc edema	Optic disc edema	Asymptomatic	Decreased visual acuity (20/40 or better); visual field defect present	Decreased visual acuity (worse than 20/40); marked visual field defect but sparing the central 20 degrees	Blindness (20/200 or worse)	—
ALSO CONSIDER: Neuropathy: cranial – *Select*.						
Proptosis/enophthalmos	Proptosis/enophthalmos	Asymptomatic, intervention not indicated	Symptomatic and interfering with function, but not interfering with ADL	Symptomatic and interfering with ADL	—	—
Retinal detachment	Retinal detachment	Exudative; no central vision loss; intervention not indicated	Exudative and visual acuity 20/40 or better but intervention not indicated	Rhegmatogenous or exudative detachment; operative intervention indicated	Blindness (20/200 or worse)	—
Retinopathy	Retinopathy	Asymptomatic	Symptomatic with moderate decrease in visual acuity (20/40 or better)	Symptomatic with marked decrease in visual acuity (worse than 20/40)	Blindness (20/200 or worse)	—

OCULAR/VISUAL

		Grade				
**Adverse Event**	**Short Name**	**1**	**2**	**3**	**4**	**5**
Scleral necrosis/melt	Scleral necrosis	Asymptomatic or symptomatic but not interfering with function	Symptomatic, interfering with function but not interfering with ADL; moderate decrease in visual acuity (20/40 or better); medical intervention indicated	Symptomatic, interfering with ADL; marked decrease in visual acuity (worse than 20/40); operative intervention indicated	Blindness (20/200 or worse); painful eye with enucleation indicated	—
Uveitis	Uveitis	Asymptomatic	Anterior uveitis; medical intervention indicated	Posterior or pan-uveitis; operative intervention indicated	Blindness (20/200 or worse)	—
Vision-blurred vision	Blurred vision	Symptomatic not interfering with function	Symptomatic and interfering with function, but not interfering with ADL	Symptomatic and interfering with ADL	Disabling	—
Vision-flashing lights/floaters	Flashing lights	Symptomatic not interfering with function	Symptomatic and interfering with function, but not interfering with ADL	Symptomatic and interfering with ADL	Disabling	—
Vision-photophobia	Photophobia	Symptomatic not interfering with function	Symptomatic and interfering with function, but not interfering with ADL	Symptomatic and interfering with ADL	Disabling	—
Vitreous hemorrhage	Vitreous hemorrhage	Asymptomatic, clinical findings only	Symptomatic, interfering with function, but not interfering with ADL; intervention not indicated	Symptomatic, interfering with ADL; vitrectomy indicated	—	—
Watery eye (epiphora, tearing)	Watery eye	Symptomatic, intervention not indicated	Symptomatic, interfering with function but not interfering with ADL	Symptomatic, interfering with ADL	—	—
Ocular/Visual – Other (Specify, __)	Ocular – Other (Specify)	Symptomatic not interfering with function	Symptomatic and interfering with function, but not interfering with ADL	Symptomatic and interfering with ADL	Blindness (20/200 or worse)	Death

PAIN

		Grade				
**Adverse Event**	**Short Name**	**1**	**2**	**3**	**4**	**5**
Pain<br>– *Select*:<br>'*Select*' AEs appear at the end of the CATEGORY.	Pain – *Select*	Mild pain not interfering with function	Moderate pain; pain or analgesics interfering with function, but not interfering with ADL	Severe pain; pain or analgesics severely interfering with ADL	Disabling	—
Pain – Other (Specify, __)	Pain – Other (Specify)	Mild pain not interfering with function	Moderate pain; pain or analgesics interfering with function, but not interfering with ADL	Severe pain; pain or analgesics severely interfering with ADL	Disabling	—

PAIN – *SELECT*

AUDITORY/EAR
- External ear
- Middle ear

CARDIOVASCULAR
- Cardiac/heart
- Pericardium

DERMATOLOGY/SKIN
- Face
- Lip
- Oral-gums
- Scalp
- Skin

GASTROINTESTINAL
- Abdomen NOS
- Anus
- Dental/teeth/peridontal
- Esophagus
- Oral cavity
- Peritoneum
- Rectum
- Stomach

GENERAL
- Pain NOS
- Tumor pain

HEPATOBILIARY/PANCREAS
- Gallbladder
- Liver

LYMPHATIC
- Lymph node

MUSCULOSKELETAL
- Back
- Bone
- Buttock
- Extremity-limb
- Intestine
- Joint
- Muscle
- Neck
- Phantom (pain associated with missing limb)

NEUROLOGY
- Head/headache
- Neuralgia/peripheral nerve

OCULAR
- Eye

PULMONARY/UPPER RESPIRATORY
- Chest wall
- Chest/thorax NOS

PULMONARY/UPPER RESPIRATORY (*continued*)
- Larynx
- Pleura
- Sinus
- Throat/pharynx/larynx

RENAL/GENITOURINARY
- Bladder
- Kidney

SEXUAL/REPRODUCTIVE FUNCTION
- Breast
- Ovulatory
- Pelvis
- Penis
- Perineum
- Prostate
- Scrotum
- Testicle
- Urethra
- Uterus
- Vagina

PULMONARY/UPPER RESPIRATORY

		Grade				
**Adverse Event**	**Short Name**	**1**	**2**	**3**	**4**	**5**
Adult Respiratory Distress Syndrome (ARDS)	ARDS	—	—	Present, intubation not indicated	Present, intubation indicated	Death
ALSO CONSIDER: Dyspnea (shortness of breath); Hypoxia; Pneumonitis/pulmonary infiltrates.						
Aspiration	Aspiration	Asymptomatic ("silent aspiration"); endoscopy or radiographic (e.g., barium swallow) findings	Symptomatic (e.g., altered eating habits, coughing or choking episodes consistent with aspiration); medical intervention indicated (e.g., antibiotics, suction or oxygen)	Clinical or radiographic signs of pneumonia or pneumonitis; unable to aliment orally	Life-threatening (e.g., aspiration pneumonia or pneumonitis)	Death
ALSO CONSIDER: Infection (documented clinically or microbiologically) with Grade 3 or 4 neutrophils (ANC <1.0 x 10^9/L) – *Select;* Infection with normal ANC or Grade 1 or 2 neutrophils – *Select;* Infection with unknown ANC – *Select;* Laryngeal nerve dysfunction; Neuropathy: cranial – *Select*; Pneumonitis/pulmonary infiltrates.						
Atelectasis	Atelectasis	Asymptomatic	Symptomatic (e.g., dyspnea, cough), medical intervention indicated (e.g., bronchoscopic suctioning, chest physiotherapy, suctioning)	Operative (e.g., stent, laser) intervention indicated	Life-threatening respiratory compromise	Death
ALSO CONSIDER: Adult Respiratory Distress Syndrome (ARDS); Cough; Dyspnea (shortness of breath); Hypoxia; Infection (documented clinically or microbiologically) with Grade 3 or 4 neutrophils (ANC <1.0 x 109/L) – *Select*; Infection with normal ANC or Grade 1 or 2 neutrophils – *Select*; Infection with unknown ANC – *Select*; Obstruction/stenosis of airway – *Select*; Pneumonitis/pulmonary infiltrates; Pulmonary fibrosis (radiographic changes).						
Bronchospasm, wheezing	Bronchospasm	Asymptomatic	Symptomatic not interfering with function	Symptomatic interfering with function	Life-threatening	Death
ALSO CONSIDER: Allergic reaction/hypersensitivity (including drug fever); Dyspnea (shortness of breath).						
Carbon monoxide diffusion capacity (DL_{CO})	DL_{CO}	90 – 75% of predicted value	<75 – 50% of predicted value	<50 – 25% of predicted value	<25% of predicted value	Death
ALSO CONSIDER: Hypoxia; Pneumonitis/pulmonary infiltrates; Pulmonary fibrosis (radiographic changes).						
Chylothorax	Chylothorax	Asymptomatic	Symptomatic; thoracentesis or tube drainage indicated	Operative intervention indicated	Life-threatening (e.g., hemodynamic instability or ventilatory support indicated)	Death
Cough	Cough	Symptomatic, non-narcotic medication only indicated	Symptomatic and narcotic medication indicated	Symptomatic and significantly interfering with sleep or ADL	—	—

		Grade				
**Adverse Event**	**Short Name**	**1**	**2**	**3**	**4**	**5**
Dyspnea (shortness of breath)	Dyspnea	Dyspnea on exertion, but can walk 1 flight of stairs without stopping	Dyspnea on exertion but unable to walk 1 flight of stairs or 1 city block (0.1km) without stopping	Dyspnea with ADL	Dyspnea at rest; intubation/ventilator indicated	Death
ALSO CONSIDER: Hypoxia; Neuropathy: motor; Pneumonitis/pulmonary infiltrates; Pulmonary fibrosis (radiographic changes).						
Edema, larynx	Edema, larynx	Asymptomatic edema by exam only	Symptomatic edema, no respiratory distress	Stridor; respiratory distress; interfering with ADL	Life-threatening airway compromise; tracheotomy, intubation, or laryngectomy indicated	Death
ALSO CONSIDER: Allergic reaction/hypersensitivity (including drug fever).						
FEV_1	FEV_1	90 – 75% of predicted value	<75 – 50% of predicted value	<50 – 25% of predicted value	<25% of predicted	Death
Fistula, pulmonary/upper respiratory – *Select*: – Bronchus – Larynx – Lung – Oral cavity – Pharynx – Pleura – Trachea	Fistula, pulmonary – *Select*	Asymptomatic, radiographic findings only	Symptomatic, tube thoracostomy or medical management indicated; associated with altered respiratory function but not interfering with ADL	Symptomatic and associated with altered respiratory function interfering with ADL; or endoscopic (e.g., stent) or primary closure by operative intervention indicated	Life-threatening consequences; operative intervention with thoracoplasty, chronic open drainage or multiple thoracotomies indicated	Death
REMARK: A fistula is defined as an abnormal communication between two body cavities, potential spaces, and/or the skin. The site indicated for a fistula should be the site from which the abnormal process is believed to have arisen. For example, a tracheo-esophageal fistula arising in the context of a resected or irradiated esophageal cancer should be graded as Fistula, GI – esophagus in the GASTROINTESTINAL CATEGORY.						
NAVIGATION NOTE: Hemoptysis is graded as Hemorrhage, pulmonary/upper respiratory – *Select* in the HEMORRHAGE/BLEEDING CATEGORY.						
Hiccoughs (hiccups, singultus)	Hiccoughs	Symptomatic, intervention not indicated	Symptomatic, intervention indicated	Symptomatic, significantly interfering with sleep or ADL	—	—
Hypoxia	Hypoxia	—	Decreased O_2 saturation with exercise (e.g., pulse oximeter <88%); intermittent supplemental oxygen	Decreased O_2 saturation at rest; continuous oxygen indicated	Life-threatening; intubation or ventilation indicated	Death

		Grade				
**Adverse Event**	**Short Name**	**1**	**2**	**3**	**4**	**5**
Nasal cavity/paranasal sinus reactions	Nasal/paranasal reactions	Asymptomatic mucosal crusting, blood-tinged secretions	Symptomatic stenosis or edema/narrowing interfering with airflow	Stenosis with significant nasal obstruction; interfering with ADL	Necrosis of soft tissue or bone	Death
ALSO CONSIDER: Infection (documented clinically or microbiologically) with Grade 3 or 4 neutrophils (ANC <1.0 x 10^9/L) – *Select;* Infection with normal ANC or Grade 1 or 2 neutrophils – *Select;* Infection with unknown ANC – *Select*.						
Obstruction/stenosis of airway – *Select*: – Bronchus – Larynx – Pharynx – Trachea	Airway obstruction – *Select*	Asymptomatic obstruction or stenosis on exam, endoscopy, or radiograph	Symptomatic (e.g., noisy airway breathing), but causing no respiratory distress; medical management indicated (e.g., steroids)	Interfering with ADL; stridor or endoscopic intervention indicated (e.g., stent, laser)	Life-threatening airway compromise; tracheotomy or intubation indicated	Death
Pleural effusion (non-malignant)	Pleural effusion	Asymptomatic	Symptomatic, intervention such as diuretics or up to 2 therapeutic thoracenteses indicated	Symptomatic and supplemental oxygen, >2 therapeutic thoracenteses, tube drainage, or pleurodesis indicated	Life-threatening (e.g., causing hemodynamic instability or ventilatory support indicated)	Death
ALSO CONSIDER: Atelectasis; Cough; Dyspnea (shortness of breath); Hypoxia; Pneumonitis/pulmonary infiltrates; Pulmonary fibrosis (radiographic changes).						
NAVIGATION NOTE: Pleuritic pain is graded as Pain – *Select* in the PAIN CATEGORY.						
Pneumonitis/pulmonary infiltrates	Pneumonitis	Asymptomatic, radiographic findings only	Symptomatic, not interfering with ADL	Symptomatic, interfering with ADL; O_2 indicated	Life-threatening; ventilatory support indicated	Death
ALSO CONSIDER: Adult Respiratory Distress Syndrome (ARDS); Cough; Dyspnea (shortness of breath); Hypoxia; Infection (documented clinically or microbiologically) with Grade 3 or 4 neutrophils (ANC <1.0 x 10^9/L) – *Select;* Infection with normal ANC or Grade 1 or 2 neutrophils – *Select;* Infection with unknown ANC – *Select*; Pneumonitis/pulmonary infiltrates; Pulmonary fibrosis (radiographic changes).						
Pneumothorax	Pneumothorax	Asymptomatic, radiographic findings only	Symptomatic; intervention indicated (e.g., hospitalization for observation, tube placement without sclerosis)	Sclerosis and/or operative intervention indicated	Life-threatening, causing hemodynamic instability (e.g., tension pneumothorax); ventilatory support indicated	Death
Prolonged chest tube drainage or air leak after pulmonary resection	Chest tube drainage or leak	—	Sclerosis or additional tube thoracostomy indicated	Operative intervention indicated (e.g., thoracotomy with stapling or sealant application)	Life-threatening; debilitating; organ resection indicated	Death

		Grade				
**Adverse Event**	**Short Name**	**1**	**2**	**3**	**4**	**5**
Prolonged intubation after pulmonary resection (>24 hrs after surgery)	Prolonged intubation	—	Extubated within 24 – 72 hrs postoperatively	Extubated >72 hrs postoperatively, but before tracheostomy indicated	Tracheostomy indicated	Death
NAVIGATION NOTE: Pulmonary embolism is graded as Grade 4 either as Thrombosis/embolism (vascular access-related) or Thrombosis/thrombus/embolism in the VASCULAR CATEGORY.						
Pulmonary fibrosis (radiographic changes)	Pulmonary fibrosis	Minimal radiographic findings (or patchy or bi-basilar changes) with estimated radiographic proportion of total lung volume that is fibrotic of <25%	Patchy or bi-basilar changes with estimated radiographic proportion of total lung volume that is fibrotic of 25 – <50%	Dense or widespread infiltrates/consolidation with estimated radiographic proportion of total lung volume that is fibrotic of 50 – <75%	Estimated radiographic proportion of total lung volume that is fibrotic is ≥75%; honeycombing	Death
REMARK: Fibrosis is usually a "late effect" seen >3 months after radiation or combined modality therapy (including surgery). It is thought to represent scar/fibrotic lung tissue. It may be difficult to distinguish from pneumonitis that is generally seen within 3 months of radiation or combined modality therapy.<br>ALSO CONSIDER: Adult Respiratory Distress Syndrome (ARDS); Cough; Dyspnea (shortness of breath); Hypoxia; Infection (documented clinically or microbiologically) with Grade 3 or 4 neutrophils (ANC <1.0 x 10^9/L) – *Select;* Infection with normal ANC or Grade 1 or 2 neutrophils – *Select;* Infection with unknown ANC – *Select.*						
NAVIGATION NOTE: Recurrent laryngeal nerve dysfunction is graded as Laryngeal nerve dysfunction in the NEUROLOGY CATEGORY.						
Vital capacity	Vital capacity	90 – 75% of predicted value	<75 – 50% of predicted value	<50 – 25% of predicted value	<25% of predicted value	Death
Voice changes/dysarthria (e.g., hoarseness, loss or alteration in voice, laryngitis)	Voice changes	Mild or intermittent hoarseness or voice change, but fully understandable	Moderate or persistent voice changes, may require occasional repetition but understandable on telephone	Severe voice changes including predominantly whispered speech; may require frequent repetition or face-to-face contact for understandability; requires voice aid (e.g., electrolarynx) for ≤50% of communication	Disabling; non-understandable voice or aphonic; requires voice aid (e.g., electrolarynx) for >50% of communication or requires >50% written communication	Death
ALSO CONSIDER: Laryngeal nerve dysfunction; Speech impairment (e.g., dysphasia or aphasia).						
Pulmonary/Upper Respiratory – Other (Specify, __)	Pulmonary – Other (Specify)	Mild	Moderate	Severe	Life-threatening; disabling	Death

RENAL/GENITOURINARY

		Grade				
**Adverse Event**	**Short Name**	**1**	**2**	**3**	**4**	**5**
Bladder spasms	Bladder spasms	Symptomatic, intervention not indicated	Symptomatic, antispasmodics indicated	Narcotics indicated	Major surgical intervention indicated (e.g., cystectomy)	—
Cystitis	Cystitis	Asymptomatic	Frequency with dysuria; macroscopic hematuria	Transfusion; IV pain medications; bladder irrigation indicated	Catastrophic bleeding; major non-elective intervention indicated	Death
ALSO CONSIDER: Infection (documented clinically or microbiologically) with Grade 3 or 4 neutrophils (ANC <1.0 x 109/L) – *Select*; Infection with normal ANC or Grade 1 or 2 neutrophils – *Select*; Infection with unknown ANC – *Select*; Pain – *Select*.						
Fistula, GU – *Select*:<br>– Bladder<br>– Genital tract-female<br>– Kidney<br>– Ureter<br>– Urethra<br>– Uterus<br>– Vagina	Fistula, GU – *Select*	Asymptomatic, radiographic findings only	Symptomatic; noninvasive intervention indicated	Symptomatic interfering with ADL; invasive intervention indicated	Life-threatening consequences; operative intervention requiring partial or full organ resection; permanent urinary diversion	Death
REMARK: A fistula is defined as an abnormal communication between two body cavities, potential spaces, and/or the skin. The site indicated for a fistula should be the site from which the abnormal process is believed to have originated.						
Incontinence, urinary	Incontinence, urinary	Occasional (e.g., with coughing, sneezing, etc.), pads not indicated	Spontaneous, pads indicated	Interfering with ADL; intervention indicated (e.g., clamp, collagen injections)	Operative intervention indicated (e.g., cystectomy or permanent urinary diversion)	—
Leak (including anastomotic), GU – *Select*:<br>– Bladder<br>– Fallopian tube<br>– Kidney<br>– Spermatic cord<br>– Stoma<br>– Ureter<br>– Urethra<br>– Uterus<br>– Vagina<br>– Vas deferens	Leak, GU – *Select*	Asymptomatic, radiographic findings only	Symptomatic; medical intervention indicated	Symptomatic, interfering with GU function; invasive or endoscopic intervention indicated	Life-threatening	Death
REMARK: Leak (including anastomotic), GU – *Select* refers to clinical signs and symptoms or radiographic confirmation of anastomotic leak but without development of fistula.						

		Grade				
**Adverse Event**	**Short Name**	**1**	**2**	**3**	**4**	**5**
Obstruction, GU – *Select*:<br>– Bladder<br>– Fallopian tube<br>– Prostate<br>– Spermatic cord<br>– Stoma<br>– Testes<br>– Ureter<br>– Urethra<br>– Uterus<br>– Vagina<br>– Vas deferens	Obstruction, GU – *Select*	Asymptomatic, radiographic or endoscopic findings only	Symptomatic but no hydronephrosis, sepsis or renal dysfunction; dilation or endoscopic repair or stent placement indicated	Symptomatic and altered organ function (e.g., sepsis or hydronephrosis, or renal dysfunction); operative intervention indicated	Life-threatening consequences; organ failure or operative intervention requiring complete organ resection indicated	Death
NAVIGATION NOTE: Operative injury is graded as Intra-operative injury – *Select Organ or Structure* in the SURGERY/INTRA-OPERATIVE INJURY CATEGORY.						
Perforation, GU – *Select*:<br>– Bladder<br>– Fallopian tube<br>– Kidney<br>– Ovary<br>– Prostate<br>– Spermatic cord<br>– Stoma<br>– Testes<br>– Ureter<br>– Urethra<br>– Uterus<br>– Vagina<br>– Vas deferens	Perforation, GU – *Select*	Asymptomatic radiographic findings only	Symptomatic, associated with altered renal/GU function	Symptomatic, operative intervention indicated	Life-threatening consequences or organ failure; operative intervention requiring organ resection indicated	Death
Prolapse of stoma, GU	Prolapse stoma, GU	Asymptomatic; special intervention, extraordinary care not indicated	Extraordinary local care or maintenance; minor revision under local anesthesia indicated	Dysfunctional stoma; operative intervention or major stomal revision indicated	Life-threatening consequences	Death
REMARK: Other stoma complications may be graded as Fistula, GU – *Select*; Leak (including anastomotic), GU – *Select*; Obstruction, GU – *Select;* Perforation, GU – *Select*; Stricture/stenosis (including anastomotic), GU – *Select*.						
Renal failure	Renal failure	—	—	Chronic dialysis not indicated	Chronic dialysis or renal transplant indicated	Death
ALSO CONSIDER: Glomerular filtration rate.						

RENAL/GENITOURINARY

		Grade				
**Adverse Event**	**Short Name**	**1**	**2**	**3**	**4**	**5**
Stricture/stenosis (including anastomotic), GU – *Select*:<br>– Bladder<br>– Fallopian tube<br>– Prostate<br>– Spermatic cord<br>– Stoma<br>– Testes<br>– Ureter<br>– Urethra<br>– Uterus<br>– Vagina<br>– Vas deferens	Stricture, anastomotic, GU – *Select*	Asymptomatic, radiographic or endoscopic findings only	Symptomatic but no hydronephrosis, sepsis or renal dysfunction; dilation or endoscopic repair or stent placement indicated	Symptomatic and altered organ function (e.g., sepsis or hydronephrosis, or renal dysfunction); operative intervention indicated	Life-threatening consequences; organ failure or operative intervention requiring organ resection indicated	Death
ALSO CONSIDER: Obstruction, GU – *Select*.						
Urinary electrolyte wasting (e.g., Fanconi's syndrome, renal tubular acidosis)	Urinary electrolyte wasting	Asymptomatic, intervention not indicated	Mild, reversible and manageable with replacement	Irreversible, requiring continued replacement	—	—
ALSO CONSIDER: Acidosis (metabolic or respiratory); Bicarbonate, serum-low; Calcium, serum-low (hypocalcemia); Phosphate, serum-low (hypophosphatemia).						
Urinary frequency/urgency	Urinary frequency	Increase in frequency or nocturia up to 2 x normal; enuresis	Increase >2 x normal but <hourly	≥1 x/hr; urgency; catheter indicated	—	—
Urinary retention (including neurogenic bladder)	Urinary retention	Hesitancy or dribbling, no significant residual urine; retention occurring during the immediate postoperative period	Hesitancy requiring medication; or operative bladder atony requiring indwelling catheter beyond immediate postoperative period but for <6 weeks	More than daily catheterization indicated; urological intervention indicated (e.g., TURP, suprapubic tube, urethrotomy)	Life-threatening consequences; organ failure (e.g., bladder rupture); operative intervention requiring organ resection indicated	Death
REMARK: The etiology of retention (if known) is graded as Obstruction, GU – *Select*; Stricture/stenosis (including anastomotic), GU – *Select*.<br>ALSO CONSIDER: Obstruction, GU – *Select*; Stricture/stenosis (including anastomotic), GU – *Select*.						
Urine color change	Urine color change	Present	—	—	—	—
REMARK: Urine color refers to change that is not related to other dietary or physiologic cause (e.g., bilirubin, concentrated urine, and hematuria).						
Renal/Genitourinary – Other (Specify, __)	Renal – Other (Specify)	Mild	Moderate	Severe	Life-threatening; disabling	Death

SECONDARY MALIGNANCY

		Grade				
**Adverse Event**	**Short Name**	**1**	**2**	**3**	**4**	**5**
Secondary Malignancy – possibly related to cancer treatment (Specify, __)	Secondary Malignancy (possibly related to cancer treatment)	—	—	Non-life-threatening basal or squamous cell carcinoma of the skin	Solid tumor, leukemia or lymphoma	Death

REMARK: Secondary malignancy excludes metastasis from initial primary. Any malignancy possibly related to cancer treatment (including AML/MDS) should be reported via the routine reporting mechanisms outlined in each protocol. Important: Secondary Malignancy is an exception to NCI Expedited Adverse Event Reporting Guidelines. Secondary Malignancy is "Grade 4, present" but NCI does not require AdEERS Expedited Reporting for any (related or unrelated to treatment) Secondary Malignancy. A diagnosis of AML/MDS following treatment with an NCI-sponsored investigational agent is to be reported using the form available from the CTEP Web site at http://ctep.cancer.gov. Cancers not suspected of being treatment-related are not to be reported here.

		Grade				
**Adverse Event**	**Short Name**	**1**	**2**	**3**	**4**	**5**
Breast function/lactation	Breast function	Mammary abnormality, not functionally significant	Mammary abnormality, functionally significant	—	—	—
Breast nipple/areolar deformity	Nipple/areolar	Limited areolar asymmetry with no change in nipple/areolar projection	Asymmetry of nipple areolar complex with slight deviation in nipple projection	Marked deviation of nipple projection	—	—
Breast volume/hypoplasia	Breast	Minimal asymmetry; minimal hypoplasia	Asymmetry exists, ≤1/3 of the breast volume; moderate hypoplasia	Asymmetry exists, >1/3 of the breast volume; severe hypoplasia	—	—
REMARK: Breast volume is referenced with both arms straight overhead.						
NAVIGATION NOTE: Dysmenorrhea is graded as Pain – *Select* in the PAIN CATEGORY.						
NAVIGATION NOTE: Dyspareunia is graded as Pain – *Select* in the PAIN CATEGORY.						
NAVIGATION NOTE: Dysuria (painful urination) is graded as Pain – *Select* in the PAIN CATEGORY.						
Erectile dysfunction	Erectile dysfunction	Decrease in erectile function (frequency/rigidity of erections) but erectile aids not indicated	Decrease in erectile function (frequency/rigidity of erections), erectile aids indicated	Decrease in erectile function (frequency/rigidity of erections) but erectile aids not helpful; penile prosthesis indicated	—	—
Ejaculatory dysfunction	Ejaculatory dysfunction	Diminished ejaculation	Anejaculation or retrograde ejaculation	—	—	—
NAVIGATION NOTE: Feminization of male is graded in the ENDOCRINE CATEGORY.						
Gynecomastia	Gynecomastia	—	Asymptomatic breast enlargement	Symptomatic breast enlargement; intervention indicated	—	—
ALSO CONSIDER: Pain – *Select*.						
Infertility/sterility	Infertility/sterility	—	Male: oligospermia/low sperm count<br>Female: diminished fertility/ovulation	Male: sterile/azoospermia<br>Female: infertile/ anovulatory	—	—
Irregular menses (change from baseline)	Irregular menses	1 – 3 months without menses	>3 – 6 months without menses but continuing menstrual cycles	Persistent amenorrhea for >6 months	—	—

		Grade				
**Adverse Event**	**Short Name**	**1**	**2**	**3**	**4**	**5**
Libido	Libido	Decrease in interest but not affecting relationship; intervention not indicated	Decrease in interest and adversely affecting relationship; intervention indicated	—	—	—
NAVIGATION NOTE: Masculinization of female is graded in the ENDOCRINE CATEGORY.						
Orgasmic dysfunction	Orgasmic function	Transient decrease	Decrease in orgasmic response requiring intervention	Complete inability of orgasmic response; not responding to intervention	—	—
NAVIGATION NOTE: Pelvic pain is graded as Pain – *Select* in the PAIN CATEGORY.						
NAVIGATION NOTE: Ulcers of the labia or perineum are graded as Ulceration in DERMATOLOGY/SKIN CATEGORY.						
Vaginal discharge (non-infectious)	Vaginal discharge	Mild	Moderate to heavy; pad use indicated	—	—	—
Vaginal dryness	Vaginal dryness	Mild	Interfering with sexual function; dyspareunia; intervention indicated	—	—	—
ALSO CONSIDER: Pain – *Select*.						
Vaginal mucositis	Vaginal mucositis	Erythema of the mucosa; minimal symptoms	Patchy ulcerations; moderate symptoms or dyspareunia	Confluent ulcerations; bleeding with trauma; unable to tolerate vaginal exam, sexual intercourse or tampon placement	Tissue necrosis; significant spontaneous bleeding; life-threatening consequences	—
Vaginal stenosis/length	Vaginal stenosis	Vaginal narrowing and/or shortening not interfering with function	Vaginal narrowing and/or shortening interfering with function	Complete obliteration; not surgically correctable	—	—
Vaginitis (not due to infection)	Vaginitis	Mild, intervention not indicated	Moderate, intervention indicated	Severe, not relieved with treatment; ulceration, but operative intervention not indicated	Ulceration and operative intervention indicated	—
Sexual/Reproductive Function – Other (Specify, __)	Sexual – Other (Specify)	Mild	Moderate	Severe	Disabling	Death

SURGERY/INTRA-OPERATIVE INJURY

		Grade				
**Adverse Event**	**Short Name**	**1**	**2**	**3**	**4**	**5**
NAVIGATION NOTE: Intra-operative hemorrhage is graded as Hemorrhage/bleeding associated with surgery, intra-operative or postoperative in the HEMORRHAGE/BLEEDING CATEGORY.						
Intra-operative injury – *Select Organ or Structure*<br>*'Select'* AEs appear at the end of the CATEGORY.	Intraop injury – *Select*	Primary repair of injured organ/structure indicated	Partial resection of injured organ/structure indicated	Complete resection or reconstruction of injured organ/structure indicated	Life threatening consequences; disabling	—
REMARK: The *'Select'* AEs are defined as significant, unanticipated injuries that are recognized at the time of surgery. These AEs do not refer to additional surgical procedures that must be performed because of a change in the operative plan based on intra-operative findings. Any sequelae resulting from the intra-operative injury that result in an adverse outcome for the patient must also be recorded and graded under the relevant CTCAE Term.						
Intra-operative Injury – Other (Specify, __)	Intraop Injury – Other (Specify)	Primary repair of injured organ/structure indicated	Partial resection of injured organ/structure indicated	Complete resection or reconstruction of injured organ/structure indicated	Life threatening consequences; disabling	—
REMARK: Intra-operative Injury – Other (Specify, __) is to be used only to report an organ/structure not included in the *'Select'* AEs found at the end of the CATEGORY. Any sequelae resulting from the intra-operative injury that result in an adverse outcome for the patient must also be recorded and graded under the relevant CTCAE Term.						

SURGERY/INTRA-OPERATIVE INJURY – *SELECT*

AUDITORY/EAR
- Inner ear
- Middle ear
- Outer ear NOS
- Outer ear-Pinna

CARDIOVASCULAR
- Artery-aorta
- Artery-carotid
- Artery-cerebral
- Artery-extremity (lower)
- Artery-extremity (upper)
- Artery-hepatic
- Artery-major visceral artery
- Artery-pulmonary
- Artery NOS
- Heart
- Spleen
- Vein-extremity (lower)
- Vein-extremity (upper)
- Vein-hepatic
- Vein-inferior vena cava
- Vein-jugular
- Vein-major visceral vein
- Vein-portal vein
- Vein-pulmonary
- Vein-superior vena cava
- Vein NOS

DERMATOLOGY/SKIN
- Breast
- Nails
- Skin

ENDOCRINE
- Adrenal gland
- Parathyroid
- Pituitary

ENDOCRINE (*continued*)
- Thyroid

HEAD AND NECK
- Gingiva
- Larynx
- Lip/perioral area
- Face NOS
- Nasal cavity
- Nasopharynx
- Neck NOS
- Nose
- Oral cavity NOS
- Parotid gland
- Pharynx
- Salivary duct
- Salivary gland
- Sinus
- Teeth
- Tongue
- Upper aerodigestive NOS

GASTROINTESTINAL
- Abdomen NOS
- Anal sphincter
- Anus
- Appendix
- Cecum
- Colon
- Duodenum
- Esophagus
- Ileum
- Jejunum
- Oral
- Peritoneal cavity
- Rectum
- Small bowel NOS

GASTROINTESTINAL (*continued*)
- Stoma (GI)
- Stomach

HEPATOBILIARY/ PANCREAS
- Biliary tree-common bile duct
- Biliary tree-common hepatic duct
- Biliary tree-left hepatic duct
- Biliary tree-right hepatic duct
- Biliary tree NOS
- Gallbladder
- Liver
- Pancreas
- Pancreatic duct

MUSCULOSKELETAL
- Bone
- Cartilage
- Extremity-lower
- Extremity-upper
- Joint
- Ligament
- Muscle
- Soft tissue NOS
- Tendon

NEUROLOGY
- Brain
- Meninges
- Spinal cord

NERVES:
- Brachial plexus
- CN I (olfactory)
- CN II (optic)
- CN III (oculomotor)
- CN IV (trochlear)

NEUROLOGY (*continued*)

NERVES:
- CN V (trigeminal) motor
- CN V (trigeminal) sensory
- CN VI (abducens)
- CN VII (facial) motor-face
- CN VII (facial) sensory-taste
- CN VIII (vestibulocochlear)
- CN IX (glossopharyngeal) motor pharynx
- CN IX (glossopharyngeal) sensory ear-pharynx-tongue
- CN X (vagus)
- CN XI (spinal accessory)
- CN XII (hypoglossal)
- Cranial nerve or branch NOS
- Lingual
- Lung thoracic
- Peripheral motor NOS
- Peripheral sensory NOS
- Recurrent laryngeal
- Sacral plexus
- Sciatic
- Thoracodorsal

OCULAR
- Conjunctiva
- Cornea
- Eye NOS
- Lens
- Retina

PULMONARY/UPPER RESPIRATORY
- Bronchus
- Lung
- Mediastinum
- Pleura
- Thoracic duct
- Trachea
- Upper airway NOS

RENAL/GENITOURINARY
- Bladder
- Cervix
- Fallopian tube
- Kidney
- Ovary
- Pelvis NOS
- Penis
- Prostate
- Scrotum
- Testis
- Ureter
- Urethra
- Urinary conduit
- Urinary tract NOS
- Uterus
- Vagina
- Vulva

SYNDROMES

		Grade				
**Adverse Event**	**Short Name**	**1**	**2**	**3**	**4**	**5**
NAVIGATION NOTE: Acute vascular leak syndrome is graded in the VASCULAR CATEGORY.						
NAVIGATION NOTE: Adrenal insufficiency is graded in the ENDOCRINE CATEGORY.						
NAVIGATION NOTE: Adult Respiratory Distress Syndrome (ARDS) is graded in the PULMONARY/UPPER RESPIRATORY CATEGORY.						
Alcohol intolerance syndrome (antabuse-like syndrome)	Alcohol intolerance syndrome	—	—	Present	—	Death
REMARK: An antabuse-like syndrome occurs with some new anti-androgens (e.g., nilutamide) when patient also consumes alcohol.						
NAVIGATION NOTE: Autoimmune reaction is graded as Autoimmune reaction/hypersensitivity (including drug fever) in the ALLERGY/IMMUNOLOGY CATEGORY.						
Cytokine release syndrome/acute infusion reaction	Cytokine release syndrome	Mild reaction; infusion interruption not indicated; intervention not indicated	Requires therapy or infusion interruption but responds promptly to symptomatic treatment (e.g., antihistamines, NSAIDS, narcotics, IV fluids); prophylactic medications indicated for $\leq$24 hrs	Prolonged (i.e., not rapidly responsive to symptomatic medication and/or brief interruption of infusion); recurrence of symptoms following initial improvement; hospitalization indicated for other clinical sequelae (e.g., renal impairment, pulmonary infiltrates)	Life-threatening; pressor or ventilatory support indicated	Death
REMARK: Cytokine release syndromes/acute infusion reactions are different from Allergic/hypersensitive reactions, although some of the manifestations are common to both AEs. An acute infusion reaction may occur with an agent that causes cytokine release (e.g., monoclonal antibodies or other biological agents). Signs and symptoms usually develop during or shortly after drug infusion and generally resolve completely within 24 hrs of completion of infusion. Signs/symptoms may include: Allergic reaction/hypersensitivity (including drug fever); Arthralgia (joint pain); Bronchospasm; Cough; Dizziness; Dyspnea (shortness of breath); Fatigue (asthenia, lethargy, malaise); Headache; Hypertension; Hypotension; Myalgia (muscle pain); Nausea; Pruritis/itching; Rash/desquamation; Rigors/chills; Sweating (diaphoresis); Tachycardia; Tumor pain (onset or exacerbation of tumor pain due to treatment); Urticaria (hives, welts, wheals); Vomiting.						
ALSO CONSIDER: Allergic reaction/hypersensitivity (including drug fever); Bronchospasm, wheezing; Dyspnea (shortness of breath); Hypertension; Hypotension; Hypoxia; Prolonged QTc interval; Supraventricular and nodal arrhythmia – *Select*; Ventricular arrhythmia – *Select*.						
NAVIGATION NOTE: Disseminated intravascular coagulation (DIC) is graded in the COAGULATION CATEGORY.						
NAVIGATION NOTE: Fanconi's syndrome is graded as Urinary electrolyte wasting (e.g., Fanconi's syndrome, renal tubular acidosis) in the RENAL/GENITOURINARY CATEGORY.						
Flu-like syndrome	Flu-like syndrome	Symptoms present but not interfering with function	Moderate or causing difficulty performing some ADL	Severe symptoms interfering with ADL	Disabling	Death
REMARK: Flu-like syndrome represents a constellation of symptoms which may include cough with catarrhal symptoms, fever, headache, malaise, myalgia, prostration, and is to be used when the symptoms occur in a cluster consistent with one single pathophysiological process.						
NAVIGATION NOTE: Renal tubular acidosis is graded as Urinary electrolyte wasting (e.g., Fanconi's syndrome, renal tubular acidosis) in the RENAL/GENITOURINARY CATEGORY.						

		Grade				
**Adverse Event**	**Short Name**	**1**	**2**	**3**	**4**	**5**
"Retinoic acid syndrome"	"Retinoic acid syndrome"	Fluid retention; less than 3 kg of weight gain; intervention with fluid restriction and/or diuretics indicated	Mild to moderate signs/ symptoms; steroids indicated	Severe signs/symptoms; hospitalization indicated	Life-threatening; ventilatory support indicated	Death
REMARK: Patients with acute promyelocytic leukemia may experience a syndrome similar to "retinoic acid syndrome" in association with other agents such as arsenic trioxide. The syndrome is usually manifested by otherwise unexplained fever, weight gain, respiratory distress, pulmonary infiltrates and/or pleural effusion, with or without leukocytosis.						
ALSO CONSIDER: Acute vascular leak syndrome; Pleural effusion (non-malignant); Pneumonitis/pulmonary infiltrates.						
NAVIGATION NOTE: SIADH is graded as Neuroendocrine: ADH secretion abnormality (e.g., SIADH or low ADH) in the ENDOCRINE CATEGORY.						
NAVIGATION NOTE: Stevens-Johnson syndrome is graded as Rash: erythema multiforme (e.g., Stevens-Johnson syndrome, toxic epidermal necrolysis) in the DERMATOLOGY/SKIN CATEGORY.						
NAVIGATION NOTE: Thrombotic microangiopathy is graded as Thrombotic microangiopathy (e.g., thrombotic thrombocytopenic purpura [TTP] or hemolytic uremic syndrome [HUS]) in the COAGULATION CATEGORY.						
Tumor flare	Tumor flare	Mild pain not interfering with function	Moderate pain; pain or analgesics interfering with function, but not interfering with ADL	Severe pain; pain or analgesics interfering with function and interfering with ADL	Disabling	Death
REMARK: Tumor flare is characterized by a constellation of signs and symptoms in direct relation to initiation of therapy (e.g., anti-estrogens/androgens or additional hormones). The symptoms/signs include tumor pain, inflammation of visible tumor, hypercalcemia, diffuse bone pain, and other electrolyte disturbances.						
ALSO CONSIDER: Calcium, serum-high (hypercalcemia).						
Tumor lysis syndrome	Tumor lysis syndrome	—	—	Present	—	Death
ALSO CONSIDER: Creatinine; Potassium, serum-high (hyperkalemia).						
Syndromes – Other (Specify, __)	Syndromes – Other (Specify)	Mild	Moderate	Severe	Life-threatening; disabling	Death

VASCULAR

		Grade				
**Adverse Event**	**Short Name**	**1**	**2**	**3**	**4**	**5**
Acute vascular leak syndrome	Acute vascular leak syndrome	—	Symptomatic, fluid support not indicated	Respiratory compromise or fluids indicated	Life-threatening; pressor support or ventilatory support indicated	Death
Peripheral arterial ischemia	Peripheral arterial ischemia	—	Brief (<24 hrs) episode of ischemia managed non-surgically and without permanent deficit	Recurring or prolonged (≥24 hrs) and/or invasive intervention indicated	Life-threatening, disabling and/or associated with end organ damage (e.g., limb loss)	Death
Phlebitis (including superficial thrombosis)	Phlebitis	—	Present	—	—	—
ALSO CONSIDER: Injection site reaction/extravasation changes.						
Portal vein flow	Portal flow	—	Decreased portal vein flow	Reversal/retrograde portal vein flow	—	—
Thrombosis/embolism (vascular access-related)	Thrombosis/embolism (vascular access)	—	Deep vein thrombosis or cardiac thrombosis; intervention (e.g., anticoagulation, lysis, filter, invasive procedure) not indicated	Deep vein thrombosis or cardiac thrombosis; intervention (e.g., anticoagulation, lysis, filter, invasive procedure) indicated	Embolic event including pulmonary embolism or life-threatening thrombus	Death
Thrombosis/thrombus/ embolism	Thrombosis/thrombus/ embolism	—	Deep vein thrombosis or cardiac thrombosis; intervention (e.g., anticoagulation, lysis, filter, invasive procedure) not indicated	Deep vein thrombosis or cardiac thrombosis; intervention (e.g., anticoagulation, lysis, filter, invasive procedure) indicated	Embolic event including pulmonary embolism or life-threatening thrombus	Death
Vessel injury-artery – *Select*:<br>– Aorta<br>– Carotid<br>– Extremity-lower<br>– Extremity-upper<br>– Other NOS<br>– Visceral	Artery injury – *Select*	Asymptomatic diagnostic finding; intervention not indicated	Symptomatic (e.g., claudication); not interfering with ADL; repair or revision not indicated	Symptomatic interfering with ADL; repair or revision indicated	Life-threatening; disabling; evidence of end organ damage (e.g., stroke, MI, organ or limb loss)	Death
NAVIGATION NOTE: Vessel injury to an artery intra-operatively is graded as Intra-operative injury – *Select Organ or Structure* in the SURGERY/INTRA-OPERATIVE INJURY CATEGORY.						

		Grade				
**Adverse Event**	**Short Name**	**1**	**2**	**3**	**4**	**5**
Vessel injury-vein – *Select*:<br>– Extremity-lower<br>– Extremity-upper<br>– IVC<br>– Jugular<br>– Other NOS<br>– SVC<br>– Viscera	Vein injury – *Select*	Asymptomatic diagnostic finding; intervention not indicated	Symptomatic (e.g., claudication); not interfering with ADL; repair or revision not indicated	Symptomatic interfering with ADL; repair or revision indicated	Life-threatening; disabling; evidence of end organ damage	Death
NAVIGATION NOTE: Vessel injury to a vein intra-operatively is graded as Intra-operative injury – *Select Organ or Structure* in the SURGERY/INTRA-OPERATIVE INJURY CATEGORY.						
Visceral arterial ischemia (non-myocardial)	Visceral arterial ischemia	—	Brief (<24 hrs) episode of ischemia managed medically and without permanent deficit	Prolonged (≥24 hrs) or recurring symptoms and/or invasive intervention indicated	Life-threatening; disabling; evidence of end organ damage	Death
ALSO CONSIDER: CNS cerebrovascular ischemia.						
Vascular – Other (Specify, __)	Vascular – Other (Specify)	Mild	Moderate	Severe	Life-threatening; disabling	Death

Amendment to Clinical Trial Protocol GEN413/OFA111752, version 1

Title: An open-label, international, multi-center, phase II, extension trial investigating long-term efficacy and safety of repeated treatment courses of ofatumumab, a fully human monoclonal anti-CD20 antibody, in adult patients with active rheumatoid arthritis who previously received ofatumumab or placebo in Trial Hx-CD20-403

Substantial Amendment: ☒

Non-substantial Amendment: ☐ Rationale:

Urgent Amendment: ☐

Protocol Amendment No.: 6

Internal approval date: 5 November 2010

Reasons for Protocol Amendment

1. **Termination of study (section 1.7 -figure 1, 6.2.3, 6.3, 10 and 17)**
   The primary purpose of this amendment is to update the protocol related to the Sponsor's decision to stop clinical development of the intravenous delivery of ofatumumab in autoimmune indications, including rheumatoid arthritis, as announced in a press release issued on Sept 16th 2010 by GSK and Genmab. Since a regulatory filing with the intravenous route of administration in rheumatoid arthritis will no longer be pursued, the Sponsor has decided to prematurely terminate study GEN413/OFA111752 in accordance with Section 17 of the protocol. After approval of the amendment, no patients will receive retreatment with ofatumumab. At the next scheduled study visit patients in the Treatment period will proceed into the planned Follow-up period where they will be monitored every 12 weeks until B-cells and circulating IgG levels have returned to normal or baseline levels or for a maximum of 2 years from the last scheduled visit in the Treatment Period, whichever occurs earlier.

2. **Additional text and changes to sections have been made to be more in alignment with GSK protocol wording & SOPs (section 5.3.3, 7.1.1, 7.1.3, 12.1, 12.3 and 14.2)**
   Although GSK was operationalizing this study the previous Sponsor of this trial was the partner company Genmab, whose protocol template had been utilized at the time, upon transfer of sponsorship of this study from Genmab to GSK, based on an amendment of the ofatumaumb co-development agreement signed on July 1st 2010, additional mandatory

wording and changes to some sections based on GSK protocol template and SOPs has been implemented with this amendment.

3. **Additional Safety Follow-up assessments (Table 2, and section 8.21 and 8.27)**
   To ensure compliance with GSK SOPs , and consistency across the ofatumumab Rheumatoid Arthritis program , while maintaining the ongoing safety of patients throughout the Follow-up period additional pregnancy, Hepatitis B & C and liver function testings were included to the Follow-up period at specific time-points.

4. **Administrative changes (section 3.2.5, 7.3.4.2, 8.6 8.27, 9.3.2, 9.5.3, 10.8, 13.1, 17.1 , 18.1, 18.2 and attachment 1)**
   In addition to amending typographical errors, some sections have been updated to ensure accuracy of information.

Amended text

Amended text is struck through with a single line and new text is written in italics

1.7 Trial Design

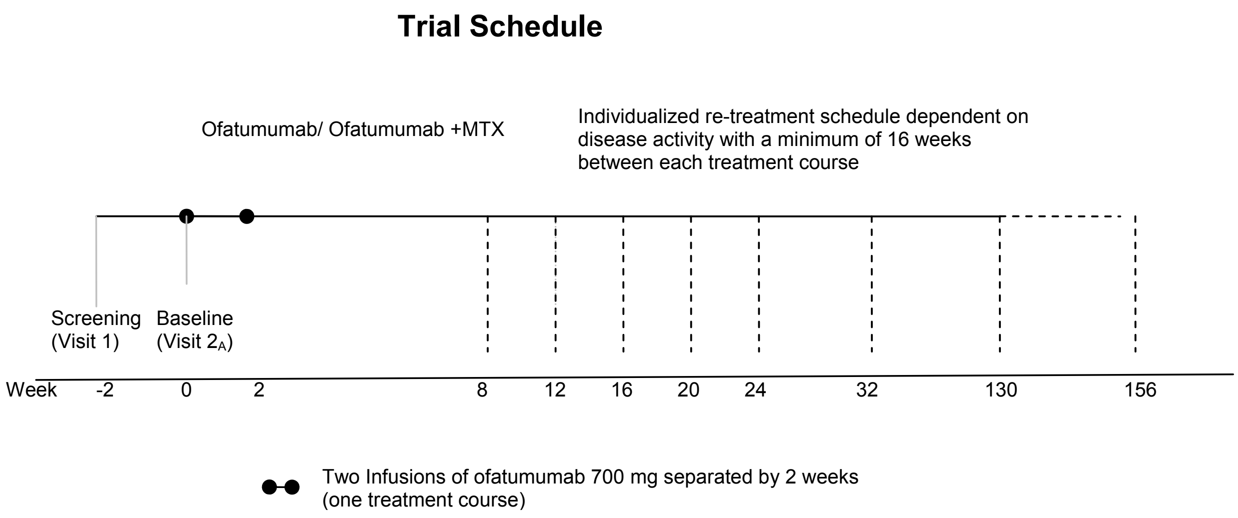

Figure 1: Trial Design

In the event the trial is prematurely terminated, all subjects in the Treatment Period will enter the Follow-Up Period at the next scheduled study visit.

2 Flow Chart

Assessment (Assessments and samples should be done/drawn prior to infusion unless stated otherwise)										
**Period**	**Screening**	**Treatment Cycle A - I[1]**								**Follow-up**
Administration of ofatumumab		Infusion **1**	Infusion **2**							
Visit Number[2]	1	2_{A-I}	3_{A-I}	4_{A-I}	5_{A-I}	6_{A-I}	7_{A-I}	8_{A-I}	$9+_{A-I}$	FU+
Week	≤2w	Indivi-dualized	2w from Infusion 1	8w from Infusion 1	12w from Infusion 1	16w from Infusion 1	20w from Infusion 1	24w from Infusion 1	Every 8w	Every 12w
Visit Window (days)	≤14 prior to Visit 2_A	-	± 2	± 3	± 3	± 3	± 3	± 3	± 14	± 14
Adverse Events[10]	X	X	X	X	X	X	X	X	X	X[11]
Concomitant Medication	X	X	X	X	X	X	X	X	X	X[12]
Tuberculosis[13]	X									
Efficacy Assessments<br>– Joint assessment[14]<br>– VAS Pain<br>– VAS Disease (patient)<br>– VAS Disease (physician)<br>– HAQ	X[15]	X		X	X	X	X	X	X	
Biochemistry/hematology	X	X	X	X	X	X	X	X	X	*X[16]*
Urine Test	X	X	X	X	X	X	X	X	X	

Table notes:

1) Each treatment cycle consists of minimum Visit 2 - 6, but may continue with additional visits until next treatment course becomes applicable. The patients can have maximum 9 treatment cycles (A - I).

2) If disease activity progresses between the scheduled visits an unscheduled visit should be performed.

10) SAEs are reported from time of informed consent. AEs occurring between Visit 1 and Visit 2_A should be recorded as Medical History.

11) Only Serious Adverse Events will be collected in the Follow-up Period.

12) Only concomitant RA medication will be collected.

13) X-ray obtained ≤12 weeks prior to screening as part of routine practice can replace the screening assessment. If clinically warranted the X-ray should be repeated at screening. The Mantoux test, or equivalent, is to be performed during the screening period. Patients who have documented BCG vaccination are exempt. ~~excempt~~.

14) The joint assessment should be performed by an independent joint evaluator.

15) Only Joint assessments, Patient Pain assessment and patient global disease assessment.

16) During Follow-up biochemistry/hematology tests are ONLY to be performed at week 12 and 24 from the last scheduled visit in the Treatment Period.

**Assessment (Assessments and samples should be done/drawn prior to infusion unless stated otherwise)**										
**Period**	**Screening**	**Treatment Cycle A - I**[1]								**Follow-up**
Administration of ofatumumab		Infusion **1**	Infusion **2**							
Visit Number[2]	1	2_{A-I}	3_{A-I}	4_{A-I}	5_{A-I}	6_{A-I}	7_{A-I}	8_{A-I}	9+$_{A-I}$	FU+
Week	≤2w	Indivi-dualized	2w from Infusion 1	8w from Infusion 1	12w from Infusion 1	16w from Infusion 1	20w from Infusion 1	24w from Infusion 1	Every 8w	Every 12w
Visit Window (days)	≤14 prior to Visit 2_A	-	± 2	± 3	± 3	± 3	± 3	± 3	± 14	± 14
Pregnancy Test	X[17]	X	X	X	X	X	X	X	X	X[18]
IgA, IgG, IgM	X	X			X			X	X	X[19]
Flow Cytometry		X	X	X	X			X	X	X[20]
Plasma/white cell JCV PCR	X				X			X	X[21]	X
ESR[22]	X	X		X	X	X	X	X	X	
CRP	X	X		X	X	X	X	X	X	
HAHA	X	X						X	X[21]	
PK		X[23]	X[23]	X	X	X	X	X	X	
Hepatitis B and C[24]	X									*X*[25]
Biomarkers / transcriptomics		X		X	X	X		X	X	
Pharmacogenetics (PGx)		X[26]								

Table notes:

1) Each treatment cycle consists of minimum Visit 2 - 6, but may continue with additional visits until next treatment course becomes applicable. The patients can have maximum 9 treatment cycles (A - I).

2) If disease activity progresses between the scheduled visits an unscheduled visit should be performed.

17) Pregnancy testing based on serum-HCG will only be done at screening *and monthly urine pregnancy testing performed at every clinic visit during the treatment cycle* ~~at the first Follow-up Visit~~. All other urine-pregnancy tests are done locally; if positive a confirmatory serum-HCG should be done.

18) During Follow-up a urine pregnancy test is ONLY to be performed at week 12 and 24, from the last scheduled visit in Treatment Period.

19) ~~IgG for patients with IgG levels below lower limit of normal~~ *During Follow-up immunoglobulins are to be performed every 12 weeks for a maximum of 2 years from the last scheduled visit in the Treatment Period..*

20) $CD19^+$ only.

21) Should only be taken every 24 weeks within each treatment cycle.

22) ESR is measured locally.

23) PK-sampling should be done prior to infusion and immediately after the end of infusion.

24) If Hepatitis C antibody positive, a confirmatory Hepatitis C assay should be reflexively performed to confirm the results (see Section 8.27)

25) During Follow-up Hepatitis B &C tests are to be performed every 24 weeks for a maximum of 2 years from the last scheduled visit in the Treatment Period

26) Should only be taken at Visit 2_A.

3.2.5 Summary of known and potential risks to human subjects

The following reactions have been observed in patients exposed to ofatumumab and should be regarded as expected infusion-related adverse reactions associated with administration of this antibody: pruritus, dyspnea, throat irritation, rigors/chills, headache, flushing, nausea, hypotension, urticaria, fatigue, fever, and rash. However, experience to date shows that these infusion-related reactions can be prevented or reduced in frequency and severity when patients are administered pre-medication prior to ofatumumab infusion as described in Section 3.2.10. Profound and prolonged depletion of peripheral $CD20^+$ B lymphocytes has been observed during treatment with ofatumumab, as expected with the administration of an anti-CD20 monoclonal antibody. To date, an increased risk of *serious* infectious complications associated with ~~this type of B-cell depletion~~ *ofatumumab* has not been observed.

Further information can be found in the current Investigator's Brochure.

5.3.3 Withdrawal from Safety Follow-up

A patient should be withdrawn from the Follow-up if at any time:

- *It is the wish of the patient (or their legally acceptable representative) for any reason*
- *The investigator judges it necessary due to medical reasons*
- *The patient initiates treatment with other B-cell suppressive treatment (e.g. other anti-CD20 antibodies, cyclophosphamide, azathioprine etc.)*
- *The patient enters another interventional clinical trial and/ or receives treatment with any non-marketed drug substance or experimental therapy.*

6.2.3 Follow-up

Patients who have completed the Treatment Period o*r are withdrawn from treatment or withdrawn from the trial (section 5.3 and 17)* will be followed every 12 weeks until B-cells have returned to normal (according to central laboratory) or baseline levels*, or for a maximum of 2 years from the last scheduled visit in the treatment period.* The B-cell baseline refers to the B-cell value measured prior to the administration of trial medication at Visit 2 during trial Hx-CD20-403. For patients withdrawn from treatment period prematurely due to IgG levels falling below normal range, IgG levels will also be followed *until IgG levels have returned to normal (according to central laboratory) or baseline levels, or for a maximum of 2 years from the last scheduled visit in the treatment period.*

Furthermore neurological examinations and plasma/white cell JCV PCR testing will be done and the patients will be followed for SAEs and concomitant RA medication.

If the patient initiates treatment with other B-cell suppressive treatment (e.g., other anti-CD20 antibodies, cyclophosphamide, or azathioprine), at any time during the trial including the Follow-up Period, *or enters another interventional trial while in the Follow-Up Period* , all patient related study

activities according to this trial protocol should be terminated. Furthermore, the investigator should document the date of initiation of other B-cell suppressive treatment *or date of initiation of other trial* and complete the End of Study Conclusion Form.

End of study is defined as date of the last patient attending the last visit, hence this will be when the last patient's B-cells have returned to baseline or normal levels (according to central laboratory) *or for a maximum of 2 years from the last scheduled visit in the treatment period whichever occurs earlier.*

6.3 Treatment after the End of the Study

The investigator is responsible for ensuring that consideration has been given to the post-study care of the patient's medical condition.

Note: GSK wil not provide any post study treatment with ofatumumab, and no compassionate use of ofatumumab for autoimmune indications is currently available for rheumatoid arthritis patients

7 Treatment

7.1 Investigational Medicinal Product (IMP)

7.1.1 Ofatumumab

Ofatumumab is a clear colorless liquid. Ofatumumab is a concentrate for solution intended for intravenous administration.

Ofatumumab is formulated at 20 mg/mL ~~adjusted to pH 6.5 (Table 1).~~ Ofatumumab is supplied in 6 mL clear glass vials. Each vial contains 5 mL of ofatumumab (20 mg/mL), i.e. a total of 100 mg ofatumumab.

~~**Table 1: Investigational Medicinal Product**~~

~~**Ingredient**~~	~~**Quantity per mL**~~	~~**Function**~~
~~Ofatumumab~~	~~20 mg~~	~~Active ingredient~~
~~Sodium Citrate USP/EP~~	~~8.549 mg~~	~~Buffering and stabilizing agent~~
~~Citric Acid USP/EP~~	~~0.195 mg~~	~~Buffering and stabilizing agent~~
~~Sodium Chloride USP/EP~~	~~5.844 mg~~	~~Isotonic agent~~
~~Water for injection USP/EP q.s. to~~	~~1 mL~~	~~Solvent~~

Ofatumumab/placebo will be filtered using an inline filter (0.2 μm) during infusion.

7.1.3 Storage of ofatumumab

Ofatumumab should be stored in a safe and secure place in a refrigerator at 2-8°C, *protected from light* and it must not be frozen.

After ofatumumab has been diluted in sterile, pyrogen free 0.9% NaCl it can be kept at room temperature and must be given to the patient within 24 hours. Exact time of dilution into 0.9% NaCl must be written on the label of the infusion bag.

Drug supplies must be kept in an appropriate restricted area, which may be accessed only by the pharmacist, or a duly designated person. A log to document the temperature with daily readings (working days only) must be kept.

If the temperature of the refrigerator is outside the limits of 2-8°C (35.6-46.4 °F) it should be noted in the temperature log. If the temperature is / has been ≤ 0 °C or ≥ 10°C (≤ 32 °F or ≥ 50 °F) for more than 8 hours the local CRA should be contacted. The following information should be available: the study number, amount of ofatumumab and data on the temperature in the refrigerator, and for how long the temperature was outside the temperature limits. If a break down of the refrigerator occurs, ofatumumab should be transferred to another temperature controlled refrigerator immediately.

Ofatumumab must not be utilized after the expiry date printed on the carton label.

7.3.4.2 Handling of Infusion Related Reactions

Previously observed infusion related AEs are described in the current Investigator´s Brochure and Section 3.2.5. In case of adverse events during infusion the patients must be treated according to the investigator's judgment and best clinical practice. Guidance on the management of infusion related reactions may also be found in the *IMP Administration Manual* ~~Study Manual~~.

8.6 Neurological Examination

A neurological examination to detect any signs or symptoms consistent with a diagnosis of PML will be conducted as part of the physical examinations required by protocol. A questionnaire will be provided and should be filled in as part of the neurological examination. If any question is answered 'Yes' the investigator should contact the *GSK* medical officer *or designee* ~~Genmab~~ to discuss appropriate management of the patient (Section9.5.3). Any findings at Visit 1 should be reported as medical history and findings after Visit 2 $_A$ should be reported as adverse events and the PML reporting procedures followed (Section9.5.3).

8.21 Pregnancy Test

For women of childbearing potential pregnancy testing will be performed. Serum samples will be drawn at screening ~~and at the patient's first Follow-up Visit~~. ~~For women of childbearing potential,~~ and ~~monthly~~ urine pregnancy testing should be performed *at every clinic visit during the* ~~during the first 24 weeks of the trial, and subsequently performed every 2nd month for the remaining trial weeks in each~~ treatment cycle. If a visit is not scheduled, home urine testing must be performed.

Serum samples are shipped to the central laboratory for immediate analysis of Human Chorionic Gonadotrophine (HCG).

Women are considered of childbearing potential unless they have been hysterectomized, or have undergone tubal ligation at least one year prior to screening, or have been postmenopausal for at least one year.

All remaining pregnancy tests *(including tests during Follow-up at week 12 and 24 from the last scheduled visit in the Treatment Period)* will be based on urine sampling and will be analyzed locally. A negative urine pregnancy test should be obtained and documented. If the urine pregnancy test is positive a serum-*HCG* pregnancy test should be obtained. Treatment with ofatumumab can only be re-commenced if results of the serum pregnancy test are negative.

.The date of sampling and outcome will be noted. If sampling for pregnancy test is not applicable, the reason for this will be documented.

8.27 Hepatitis B and C

Patients will be evaluated for serologic evidence of Hepatitis B (HB) infection based on the results of testing for HBsAg, anti-HBc and anti-HBs antibodies as follows:

- Patients positive for HBsAg are excluded
- Patients negative for HBsAg but positive for both anti-HBc and anti-HBs antibodies are eligible
- Patients negative for HBsAg and anti-HBc antibody but positive for anti-HBs antibody are eligible
- Patients negative for HBsAg and anti-HBs antibody but positive for anti-HBc antibody will require clarification of their status by testing for HB*V* DNA which if positive will exclude the patient

Patients that have documented vaccination against hepatitis B (primary and secondary immunization and booster) will be considered negative.

A blood sample will be collected and analyzed for Hepatitis C antibodies *at screening, and every 24 weeks during follow-up for a maximum of 2 years from the last scheduled visit in the Treatment Period*. If the result is positive the viral load for Hepatitis C will be analyzed in another blood sample by a confirmatory assay. Both blood samples will be taken on the same day but will be stored and shipped ambient or frozen, respectively.

9.3.2 Serious Adverse Event Reporting

The method of detecting, recording, evaluating and follow-up of AEs and SAEs plus procedures for completing and transmitting SAE reports to sponsor are provided in the *GSK SAE completing*

instructions ~~IMP Handling Manual~~. Procedures for post-study AEs/SAEs are provided in the *GSK SAE completing instructions* ~~IMP Handling Manual~~.

In case of SAEs, pregnancies and liver function abnormalities the investigator must within 24 hours of awareness of the event:

- Notify the CRA
- Immediately forward a mail with the SAE report form attached to us.naps@gsk.com or fax the SAE report form to GSK Global Clinical Safety and Pharmacovigilance at Fax no. +1 919-483-5404.

The investigator should be aware of local reporting regulations to the IRB/IEC. *GSK or a designee* ~~Genmab~~ will either supply the investigator with the reports which should be passed on to the IRB/IEC or report directly to the IRB/IEC depending on local regulations.

Events reported on the SAE report form occurring during the patient's participation in the trial must also be recorded on the AE page in the CRF

Any suspected trial drug related SAE, occurring at any time after the patient has terminated trial participation, should be reported to GSK Global Clinical Safety and Pharmacovigilance (email: us.naps@gsk.com or Fax no. +1 919-483-5404).

9.5.3 Infections and malignancies

As part of the ongoing program to evaluate the benefit/risk of ofatumumab, this trial will include enhanced safety monitoring during the trial with regard to serious infections, malignancy, and deaths.

Cases of the opportunistic viral infection progressive multifocal leukoencephalopathy (PML) have been reported in patients with hematologic malignancies ~~and~~ systemic lupus erythematosus *and rheumatoid arthritis* treated with another anti-CD20 antibody, rituximab. Additionally, cases of PML have occurred in patients who have not received rituximab. Most reports have been in patients with a compromised immune system, either due to medical conditions (lymphoma or blood cancers, HIV infection and congenital immunodeficiency syndromes and systemic lupus erythematosus) or medical treatments (cancer chemotherapy and immunosuppressive medications in organ transplant recipients)[*]. In the Hx-CD20-406 trial one case of PML was observed in a CLL patient who had received ofatumumab (1x300mg & 10x2000mg) previously treated with fludarabine and alemtuzumab with low T-lymphocyte CD4 counts.

[*] Ref FDA Alert 12/2006

In order to accommodate potential developments of PML, neurological examinations:

Neurological Symptoms Questions

		YES	NO
1.	Does the subject report any new weakness?	☐	☐
2.	Does the subject report any new difficulty with coordination or walking?		
3.	Does the subject report any new signs of confusion, impaired memory or attention?		
4.	Does the subject appear apathetic compared to previous contacts?		
5.	Does the subject report any new visual disturbances?		
6.	Has the subject had any new trouble speaking, either slurring speech, difficulty getting out words, difficulty understanding words, or difficulty comprehending spoken language.		
7.	Does the subject have any other new neurological symptoms, including but not limited to:<br>New onset seizure<br>New sensory loss<br>New emotional liability		

If any of the above are answered "Yes" at any visit, the investigator will contact the *Sponsor* medical officer *or designee* ~~at Genmab~~ and the patient will be referred to a neurologist and assessments of *plasma/white cell* JCV PCR will be performed throughout the duration of the trial. Once identified, signs and symptoms consistent with a diagnosis of PML will be reported promptly to sponsor. *Neurological symptoms should not trigger an SAE unless the sign/ symptoms is consistent with an SAE.* Signs and symptoms of PML include visual disturbances, ocular movements, ataxia, and changes in mental status such as disorientation or confusion. These symptoms are not an exhaustive list and the investigator should exercise judgment in deciding to report signs and symptoms to sponsor promptly.

~~Refer to the IMP Handling Manual for further information and detailed guidance for completing and transmitting these and other SAE reports for patients who experience a serious infection, malignancy, death, or sign or symptom of PML~~. If a patient develops neurological signs or symptoms consistent with PML, study drug is to be discontinued and the patient referred to a neurologist for evaluation. At a minimum, blood JCV PCR and/or *brain* MRI will be performed and if either is positive perform Cerebrospinal Fluid (CSF) JCV PCR. If blood JCV PCR and *brain* MRI are negative, the investigator will contact sponsor for appropriate action to be taken with study drug. All such patients will be followed until resolution. Any patient with a diagnosis of PML will be withdrawn from ofatumumab.

The investigator will do the following when reporting a serious infection, malignancy, death, or sign/symptom consistent with PML.

- Promptly report the event, as with any other SAE, as per Section of this protocol.
- Provide key source documentation for sponsor to assist with the safety evaluation process.

Examples of key source documents include but are not limited to: hospitalization records, discharge summaries, laboratory evaluations, biopsy results, culture/sensitivity results, death certificates, and autopsy reports.

If the patient has not otherwise been withdrawn from the study, then the investigator should contact sponsor to discuss the appropriate course of action regarding study continuation.

10 Statistics

This section presents the principal features of the statistical analysis of this trial. Further details will be given in a separate ~~statistical~~ *Reporting and Analysis* ~~Statistical Analysis~~ plan (*RAP* ~~SAP~~), which will be finalized before breaking the analysis population classification.

The analyses and presentations will be performed for the full analysis population unless specified otherwise.

The significance level is set to 5%. All confidence intervals will be two-sided.

All summary statistics of continuous variables will include: n, mean, median, standard deviation, minimum and maximum. All summaries presenting frequencies and incidences will include n, % and N, where N is the total number of patients with recorded values.

All data listings will include all treated patients.

In the event the sponsor decides to suspend or discontinue the intravenous route of administration development program for RA and this trial is terminated early, the primary analysis would not be performed because time to treatment withdrawal would not be a meaningful endpoint. In addition, only reduced analyses of secondary endpoints would be performed, but safety data would be reported as planned. Further details will be included in the RAP as appropriate.

10.8 Subgroups and Center Effects

The primary endpoint and the secondary efficacy endpoints will also be presented by rheum*atoid* factor seropositivity and by country.

12.1 Monitoring

Monitoring visits to the trial site will be made periodically during the trial, to ensure that:

- Data are authentic, accurate, and complete.
- Safety and rights of subjects are being protected.
- Study is conducted in accordance with the currently approved protocol and any other study agreements, GCP, and all applicable regulatory requirements.

Source documents will be reviewed for verification of agreement with data on Case Report Forms. ~~The Investigator/institution guarantees direct access to source documents by sponsor and appropriate regulatory agencies.~~.

To ensure compliance with GCP and all applicable regulatory requirements, GSK may also conduct a quality assurance audit of the site records, and the regulatory agencies may conduct a regulatory inspection at any time during or after completion of the study. In the event of an audit or inspection, the investigator (and institution) must agree to grant the auditor(s) and inspector(s) direct access to all relevant documents and to allocate their time and the time of their staff to discuss any findings/relevant issues

~~The trial site may also be audited (quality assurance) by sponsor as well as inspected by appropriate regulatory agencies.~~

It is important that the Investigator and their relevant personnel area available during the monitoring visits and possible audits and that sufficient time are devoted to the process.

12.3 *Study and Site Closure*

Upon completion or termination of the study, the GSK monitor will conduct site closure activities with the investigator or site staff (as appropriate), in accordance with applicable regulations, GCP, and GSK Standard Operating Procedures.

GSK reserves the right to temporarily suspend or terminate the study at any time for reasons including (but not limited to) safety issues, ethical issues, or severe non-compliance. If GSK determines that such action is required, GSK will discuss the reasons for taking such action with the investigator or head of the medical institution (where applicable). When feasible, GSK will provide advance notice to the investigator or head of the medical institution of the impending action.

If a study is suspended or terminated for ***safety reasons****, GSK will promptly inform all investigators, heads of the medical institutions (where applicable),and/or institutions conducting the study. GSK will also promptly inform the relevant regulatory authorities of the suspension/termination along with the reasons for such action. Where required by applicable regulations, the investigator or head of the medical institution must inform the IRB/IEC promptly and provide the reason(s) for the suspension/termination.*

13 Data Handling and Record Keeping

13.1 Case Report Form (CRF)

The Sponsor or delegate ~~Genmab~~ will supply Case Report Forms (NCR paper) which should be completed for each randomized patient and signed by the Investigator. A separate CRF screening binder should be filled in for all screened patients. Eligible patients enrolled in the trial will have an additional CRF binder. For screening failures only the Screening Evaluation Form will be entered into the database.

The CRF books must be kept on file by the investigator and maintained in an up-to-date condition at all times. The investigator must sign and date all sections in the CRF books used and also any specific forms used, as indicated. Only medically qualified investigators can sign off data on clinical assessments/safety.

The original CRF for each patient will be checked against the patient's source documents at the study site by a CRA designated by the *Sponsor* ~~Genmab~~. Instances of missing or unclear data will be discussed with the investigator for resolution. The original CRF will be taken by the CRA to *the Sponsor or a designee* ~~Genmab~~ and a copy will be left in the CRF.

Corrections of data should be made using one single line, leaving the corrected data clearly visible. The accurate data should be entered next to the inaccurate data. All changes should be initialed and dated. Correction fluids or erasers are not allowed.

Corrections to the patient data, after the original CRF pages have been retrieved by *the Sponsor or a designee* ~~Genmab~~, will be made *by the Sponsor or a designee* ~~Genmab~~ issuing a Data Clarification Form (DCF) for each item. The DCF will be completed by investigator, the original forwarded to *Sponsor or a designee* ~~Genmab~~ and a copy to the site file/CRF at the involved site.

The completed original CRFs are the sole property of GSK and should not be made available in any form to third parties, except for *a* ~~Genmab and~~ GSK representatives and representatives of appropriate Health/Regulatory Authorities, without written permission from GSK.

14.2 Publication

~~GSK acknowledges the Investigator's right to publish the entire results of the trial, regardless of the outcome, in accordance with the latest Vancouver rules (34).~~

~~The International Coordinating Investigator will together with GSK decide on the publication strategy and has the right to publish and present the results and methods as first author of multicenter publications. Co-authorship will be decided by GSK and International Coordinating Investigator and will be limited to a number of persons, who have contributed substantially in the conduct of the trial. GSK and Genmab will have representation in the list of authors.~~

~~Publications are subject to the following conditions:~~

- ~~No publication prior to the completion of the trial at all participating sites without written approval from GSK~~
- ~~All proposed publications and presentations, including any modifications or amendments shall be submitted to GSK for its review at least 30 days before such presentation or publication is submitted to any third party~~
- ~~Publications shall not disclose any Sponsor Confidential Information and Property (not including the trial results, which can be published as described elsewhere in this section).~~

14.2 *Provision of Study Results to Investigators, Posting to the Clinical Trials Register and Publication*

Where required by applicable regulatory requirements, an investigator signatory will be identified for the approval of the clinical study report. The investigator will be provided reasonable access to statistical tables, figures, and relevant reports and will have the opportunity to review the complete study results at a GSK site or other mutually-agreeable location.

GSK will also provide the investigator with the full summary of the study results. The investigator is encouraged to share the summary results with the study subjects, as appropriate.

GSK will provide the investigator with the randomization codes for their site only after completion of the full statistical analysis.

The results summary will be posted to the Clinical Study Register at the time of the first regulatory approval or within 12 months of any decision to terminate development. In addition, a manuscript will be submitted to a peer-reviewed journal for publication within 12 months of the first approval or within 12 months of any decision to terminate development. When manuscript publication in a peer-reviewed journal is not feasible, further study information will be posted to the GSK Clinical Study Register to supplement the results summary.

17 Premature Termination of the Trial

If GSK, ~~Genmab~~ International Coordinating Investigator, or the Joint Safety Committee discover conditions arising during the trial, which indicate that the clinical investigation should be halted, the trial can be temporarily suspended or terminated after appropriate consultation between GSK, ~~Genmab~~, the Joint Safety Committee, and International Coordinating Investigator. The Regulatory Authorities and Independent Ethics Committees/Institutional Review Board will be notified in writing. The reason will be stated

Conditions that may warrant termination of the trial include, but are not limited to the following:

- The discovery of an unexpected and significant or unacceptable risk to the patients enrolled in the trial.
- The discovery of lack of efficacy.
- Failure of the Investigators to enter patients at an acceptable rate in the trial as a whole.
- A decision on the part of GSK ~~in consultation with Genmab~~ to suspend or discontinue development of the drug *or formulation of drug* in this indication.

In the event the trial is prematurely terminated, all subjects in the Treatment Period will enter the Follow-Up Period at the next scheduled study visit (see section 6.2.3 for details on Follow–up activities)

17.1 Premature Termination of a Trial Site

Further GSK ~~and/or Genmab~~ can decide to prematurely terminate single sites. Conditions that may warrant termination include, but are not limited to the following:

- Insufficient adherence to protocol requirements
- Failure to enter patients at an acceptable rate.

18.1 List of abbreviations

ACR	American Collegue of Rheumatology
RAP ~~SAP~~	*Reporting and Analysis* ~~Statistical Analysis~~ Plan

18.2 Definition of Terms

Sponsor

GSK is the sponsor for this trial. ~~The trial is being conducted in collaboration with Genmab. GSK is responsible for SAE data handling and reporting while Genmab A/S is responsible for trial conduct, and therefore some of the sponsor obligations will be handled by Genmab.~~

Joint Safety Committee

A Joint Safety Committee for the trial will be established. The Joint Safety Committee will include representatives from Clinical Development, Statistics, Regulatory Affairs and Medical and Safety Departments at GSK. ~~and Genmab.~~
