# Supplementary material for: Safety of Repeated Open-Label Treatment Courses of Intravenous Ofatumumab, a Human Anti-CD20 Monoclonal Antibody, in Rheumatoid Arthritis: Results from Three Clinical Trials
Source: PLoS One. 2016 Jun 23;11(6):e0157961. doi: 10.1371/journal.pone.0157961 (PMC4919033; doi:10.1371/journal.pone.0157961)
Supplement: S1 Table — (DOCX) [file pone.0157961.s005.docx]

**S1 Table. OFA110635 - Safety of placebo and ofatumumab over the 24 weeks double-blind period (safety population).**

|  | **Patients with AEs, n (%)** | |
| --- | --- | --- |
|  | **Ofatumumab 700 mg (n=130)** | **Placebo (n=130)** |
| Any AE | 116 (89) | 71 (55) |
| Any SAE (fatal or non-fatal)* | 6 (5) | 4 (3) |
| Death | 1 (<1) | 0 |
| AE leading to discontinuation of IP or withdrawal from study | 12 (9) | 1 (<1) |
| **Most common AEs (>5% in either group)** | | |
| Rash | 27 (21) | 1 (<1) |
| Urticaria | 16 (12) | 1 (<1) |
| Urinary tract infection | 7 (5) | 9 (7) |
| Headache | 6 (5) | 8 (6) |
| Nasopharyngitis | 10 (8) | 3 (2) |
| Pruritis | 10 (8) | 2 (2) |
| Throat irritation | 10 (8) | 1 (<1) |
| Hypersensitivity | 8 (6) | 0 |
| **Infusion reactions** | | |
| Any AE on day of first infusion | 92 (71) | 12 (9) |
| Infusion-related reaction^†^ | 89 (68) | 8 (6) |
| Any AE on day of second infusion | 4 (3) | 4 (3) |
| Infusion-related reaction^‡^ | 1 (<1) | 0 |
| **Patients with an infection** | 42 (32) | 34 (26) |
| **Patients with a serious infection** | 1 (<1) | 2 (2) |
| No. of infections | 1 | 2 |

*SAEs were bacterial gastroenteritis, pneumonia, myocardial infarction, ischaemic stroke in the placebo group and angioedema, interstitial lung disease (fatal, unrelated to ofatumumab), synovitis, pulmonary embolism, diarrhoea and pneumonia, pericardial effusion in the ofatumumab group.

^†^Infusion-related reactions (events likely to represent clinical signs and symptoms characteristic of ofatumumab infusion reactions in patients with RA) were identified by a safety review team prior to unblinding.

AE, adverse event; IP, investigational product; SAE, serious adverse event.
