# Supplementary material for: Safety of Repeated Open-Label Treatment Courses of Intravenous Ofatumumab, a Human Anti-CD20 Monoclonal Antibody, in Rheumatoid Arthritis: Results from Three Clinical Trials
Source: PLoS One. 2016 Jun 23;11(6):e0157961. doi: 10.1371/journal.pone.0157961 (PMC4919033; doi:10.1371/journal.pone.0157961)
Supplement: S2 Table — (DOCX) [file pone.0157961.s006.docx]

**S2 Table. OFA110634 - Safety of placebo and ofatumumab over the 24 weeks double-blind period (safety population).**

|  | **Patients with AEs, n (%)** | |
| --- | --- | --- |
|  | **Ofatumumab 700 mg (n=86)** | **Placebo (n=83)** |
| Any AE | 72 (84) | 49 (59) |
| Any SAE (fatal or non-fatal)* | 11 (13) | 6 (7) |
| Death | 0 | 0 |
| AE leading to discontinuation of IP or withdrawal from study | 13 (15) | 4 (5) |
| **Most common AEs (>5% in either group)** | | |
| Rash | 24 (28) | 2 (2) |
| Pruritis | 8 (9) | 2 (2) |
| Urticaria | 8 (9) | 0 |
| Cough | 8 (9) | 1 (1) |
| Throat irritation | 7 (8) | 0 |
| Erythema | 5 (6) | 1 (1) |
| Nasopharyngitis | 1 (1) | 5 (6) |
| **Infusion reactions** | | |
| Any AE on day of first infusion | 59 (69) | 5 (6) |
| Infusion-related reaction^†^ | 59 (69) | 4 (5) |
| Any AE on day of second infusion | 1 (1) | 5 (6) |
| Infusion-related reaction^‡^ | 0 | 3 (4) |
| **Patients with an infection** | 16 (19) | 26 (31) |
| **Patients with a serious infection** | 0 | 1 (1) |
| No. of infections | 0 | 1 (1) |

*SAEs in the ofatumumab group were: anaphylactic reaction [3 patients], anaphylactic shock, hypersensitivity, cervical myelopathy and foot deformity. SAEs in the placebo group were:viral meningitis, arthralgia, cerebrovascular accident, JC virus test positive, pleurisy and diabetes mellitus inadequate control.

^†^Infusion-related reactions (events likely to represent clinical signs and symptoms characteristic of ofatumumab infusion reactions in patients with RA) were identified by a safety review team prior to unblinding..

AE, adverse event; IP, investigational product; SAE, serious adverse event.
