# Supplementary material for: Safety of Repeated Open-Label Treatment Courses of Intravenous Ofatumumab, a Human Anti-CD20 Monoclonal Antibody, in Rheumatoid Arthritis: Results from Three Clinical Trials
Source: PLoS One. 2016 Jun 23;11(6):e0157961. doi: 10.1371/journal.pone.0157961 (PMC4919033; doi:10.1371/journal.pone.0157961)
Supplement: S3 Table — (DOCX) [file pone.0157961.s007.docx]

S3 Table. Number (%) of patients who achieved Remission or Low Disease Activity based on DAS28 (using ESR) during the Double-Blind or Open-Label Periods, by Treatment Course (As Treated Population).

|  | **OFA110635 - Number (%) of Patients** | | | | | | | |
| --- | --- | --- | --- | --- | --- | --- | --- | --- |
|  | **Ofatumumab Treatment Course** | | | | | | | |
|  | **1** | **2** | **3** | **4** | **5** | **6** | **7** | **Overall** |
|  | **N=243** | **N=198** | **N=136** | **N=72** | **N=31** | **N=11** | **N=2** | **N=243** |
| **DAS28-ESR** | **n (%)** | **n (%)** | **n (%)** | **n (%)** | **n (%)** | **n (%)** | **n (%)** | **n (%)** |
| Remission^1^ | 38 (16) | 47 (24) | 40 (29) | 15 (21) | 13 (42) | 3 (27) | 0 (0) | 90 (37) |
| Low disease activity^2^ | 33 (14) | 33 (17) | 31 (23) | 20 (28) | 4 (13) | 1 (9) | 0 (0) | 83 (34) |
|  | **OFA110634 - Number (%) of Patients** | | | | | | | |
|  | **Ofatumumab Treatment Course** | | | | | | | |
|  | **1** | **2** | **3** | **4** | **5** | **6** | **N/A** | **Overall** |
|  | **N=148** | **N=93** | **N=63** | **N=30** | **N=13** | **N=6** |  | **N=148** |
| Remission^1^ | 15 (10) | 5 (5) | 8 (13) | 6 (20) | 2 (15) | 2 (33) |  | 25 (17) |
| Low disease activity^2^ | 7 (5) | 20 (22) | 8 (13) | 5 (17) | 2 (15) | 2 (33) |  | 29 (20) |
|  | **OFA111752 - Number (%) of Patients** | | | | | | | |
|  | **Ofatumumab Treatment Course** | | | | | | | |
|  | **1** | **2** | **3** | **4** | **5** | **6** | **7** | **Overall** |
|  | **N=91** | **N=71** | **N=53** | **N=20** | **N=8** | **N=4** | **N=2** | **N=92** |
| Remission^1^ | 6 (6.6%) | 12(16.9%) | 11(20.8%) | 4 (20%) | 3(37.5%) | 0 | 1 (50%) | 21(23) |
| Low disease activity^2^ | 13(14.3%) | 12(16.9%) | 5 (9.4%) | 5 (25%) | 1(12.5%) | 1 (25%) | 0 | 26(28) |

^1^Subjects who achieved a DAS28 score <2.6 at any time during the first 24 weeks within each treatment course.

^2^Subjects who achieved a DAS28 score ≥2.6 and <3.2 at any time during the first 24 weeks within each treatment course.
